# Fossil fuel emissions dominate Northern Hemisphere CO_2_ seasonal cycle trends under mitigation scenarios

**DOI:** 10.1038/s41467-026-75003-x

**Published:** 2026-07-01

**Authors:** Zhe Jin, Yue He, Yilong Wang, Kai Wang, Hao Xu, Yanchen Gui, Xiangjun Tian, Thomas Gasser, Shilong Piao

**Affiliations:** 1 Institute of Carbon Neutrality, Sino-French Institute for Earth System Science, College of Urban and Environmental Sciences, Peking University, Beijing, China; 2 State Key Laboratory of Tibetan Plateau Earth System, Environment and Resources (TPESER), Institute of Tibetan Plateau Research, Chinese Academy of Sciences, Beijing, China; 3 University of Chinese Academy of Sciences, Beijing, China; 4 International Institute for Applied Systems Analysis (IIASA), Laxenburg, Austria; 5 Laboratoire des Sciences du Climat et de l’Environnement (LSCE), IPSL, CEA/CNRS/UVSQ, Université Paris-Saclay, Gif-sur-Yvette, France

**Keywords:** Biogeochemistry, Climate change, Environmental sciences

## Abstract

Variations in the atmospheric CO_2_ seasonal cycle across the Northern Hemisphere have historically been dominated by terrestrial ecosystems, making ground-based observations a reliable proxy for terrestrial carbon dynamics. However, whether this dominance will persist in the future remains uncertain. Here we combine atmospheric transport modeling with factorial simulations to assess and attribute future changes in the CO_2_ seasonal cycle through 2100. We show that the dominant drivers of these changes shift fundamentally across scenarios. Under the high-emission scenario (SSP5-8.5), strengthening land sinks dominate and amplify CO_2_ seasonal variability, preserving ground-based observations as a reliable terrestrial proxy. In contrast, under the low-emission scenario (SSP1-2.6), CO_2_ seasonal amplitude declines widely, driven primarily by reduced fossil fuel emissions and their dampened seasonality. Consequently, established ground-based CO_2_ observations may no longer reliably track terrestrial carbon dynamics under mitigation pathways, underscoring the need for new approaches for monitoring and climate policy verification.

## Abstract (web summary)

Trends in the CO_2_ seasonal cycle have long been attributed to terrestrial ecosystem changes. Here, we show that under low-emission scenarios, historically negligible fossil fuel emissions become the dominant driver, reshaping this proxy relationship.

## Introduction

Atmospheric CO_2_ concentrations exhibit a pronounced seasonal cycle, characterized by regular sub-annual oscillations with maxima typically occurring in late spring and minima in late summer or early fall^1–3^. Long-term observations have documented significant trends in this seasonal cycle across different latitudes, including increases in seasonal cycle amplitude (SCA)^4–7^ and coherent shifts in the timing of spring zero-crossing date (SZC) and autumn zero-crossing date (AZC) (Supplementary Fig. 1)^8–10^. These changes primarily reflect an altered seasonality of the terrestrial carbon cycle in the Northern Hemisphere, where climate-driven enhancements in photosynthetic carbon assimilation outweigh concurrent increases in respiratory carbon losses^1,11,12^. Meanwhile, contributions from fossil fuel CO_2_ emissions and air-sea CO_2_ exchange have been minor^13,14^ over the past decades. This terrestrial dominance has established atmospheric CO_2_ measurements as reliable proxies of terrestrial carbon balance variations since the 1960s^2,15^.

However, this foundation may be fundamentally challenged under future climate and socioeconomic scenarios. Different pathways are expected to reshape the seasonal behaviors of terrestrial, oceanic, and anthropogenic CO_2_ fluxes through changes in climate conditions, biogeochemical processes, and emission policies^16–21^. Under ambitious climate mitigation scenarios, fossil fuel emissions are projected to decline substantially, potentially altering their seasonal shapes and contribution to atmospheric CO_2_ variability^16,22,23^. Simultaneously, terrestrial ecosystem responses may differ from historical trajectories as CO_2_ concentrations stabilize and climate forcing weakens^24,25^. Given the potential shifts in the seasonal shape of different flux components, the atmospheric CO_2_ seasonality can be altered, raising fundamental questions about future CO_2_ seasonal cycle attribution: will terrestrial ecosystems maintain their dominant influence, or could other flux components emerge as primary drivers and outweigh terrestrial ecosystems? As a result, the continued reliability of atmospheric CO_2_ measurements as proxies for terrestrial carbon dynamics needs to be quantitatively assessed, with significant implications for carbon monitoring and verification systems in the future.

In this study, we investigate the evolution of the Northern Hemisphere (0°−90°N) atmospheric CO_2_ seasonal cycle from the historical period (1979-2021) through 2100 under four CMIP6 scenarios (SSP1-2.6, SSP2-4.5, SSP3-7.0, and SSP5-8.5). We first validate our atmospheric transport simulations against historical records from 27 stations, with a detailed focus on two representative stations: Barrow, Alaska (BRW) and Mauna Loa, Hawaii (MLO). Subsequently, using a suite of factorial simulations, we isolate and quantify the distinct contributions of terrestrial ecosystems, fossil fuel emissions, and air-sea fluxes to projected changes in SCA, SZC, and AZC. Finally, we assess how the contributions of these drivers shift under different mitigation pathways, providing critical insights into the future utility of atmospheric CO_2_ seasonality for monitoring the carbon cycle.

## Results

### Trends in the seasonality of atmospheric CO_2_

**Fig. 1:**
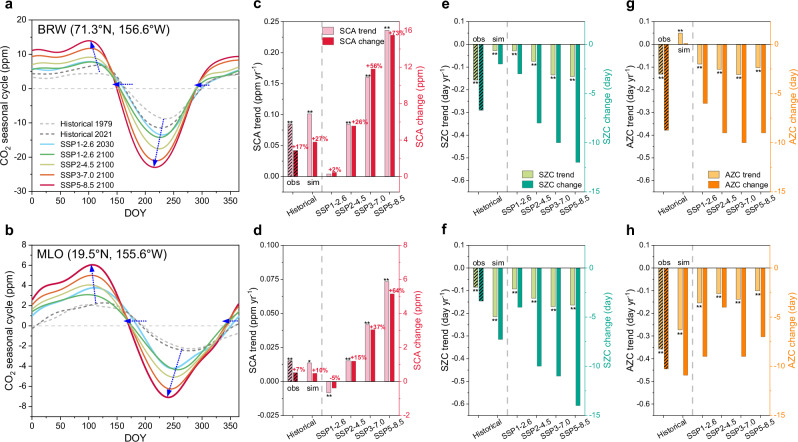
Variations in the seasonality of atmospheric CO_2_ under historical and future scenarios at the Barrow, Alaska (BRW; 71.3°N, 156.6°W) and Mauna Loa, Hawaii (MLO; 19.5°N, 155.6°W) stations. Detrended seasonal cycles of atmospheric CO_2_ simulated for the historical period (1979-2021) and future projections (2030-2100) under the SSP1-2.6, SSP2-4.5, SSP3-7.0, and SSP5-8.5 scenarios at BRW (**a**) and MLO (**b**). Given the negligible inter-scenario differences in CO_2_ seasonal cycles for 2030 (Supplementary Fig. 7), only the SSP1-2.6 result is shown as representative. Trends and changes in three CO_2_ seasonal parameters, including the seasonal cycle amplitude (SCA, ppm; **c**,** d**), the spring zero-crossing date (SZC, day of year, DOY; **e**,** f**), and the autumn zero-crossing date (AZC, DOY; **g**,** h**), are presented at BRW and MLO, respectively. In panels (**a**) and (**b**), the blue arrows indicate the direction of change in the peak, SZC, trough, and AZC of the CO_2_ seasonal cycle, from the historical state to 2100 under SSP1-2.6, then to 2100 under SSP2-4.5, SSP3-7.0, and SSP5-8.5 in sequence. For the historical period, trends and changes in observed (obs) and simulated (sim) CO_2_ seasonal cycle parameters are shown. Linear trends in SCA, SZC, and AZC were estimated from the 11-year running averages of their respective time series using the Theil-Sen method, and their significance was assessed with the Mann-Kendall non-parametric test. Significant trends (*p *< 0.05) are indicated by two dots, and marginally significant trends (*p* < 0.1) by a single dot, both placed above the bars. Values above the bars in (**c**) and (**d**) denote the percentage change in SCA over the period.

We employed the atmospheric chemistry transport model GEOS-Chem^26^ to simulate the three-dimensional variations of atmospheric CO_2_ for both the historical period and future scenarios (see Methods). CO_2_ concentrations were sampled at 27 northern ground-based monitoring stations for model validation (Supplementary Table 1; see Methods). Over the historical period, observational records across the Northern Hemisphere documented consistent increases in SCA and advances in SZC, with more variable patterns in AZC (Supplementary Fig. 2). Our transport modeling captured most of these observed trends in atmospheric CO_2_ seasonal shapes, particularly the latitudinal gradient with stronger SCA trends at high latitudes and the more pronounced SZC advances at mid-latitudes. However, simulations show limited performance for AZC trends, which are primarily driven by autumn terrestrial carbon dynamics represented by the ensemble mean of 14 dynamic global vegetation models (DGVMs) (see Methods). The DGVMs, however, face greater challenges in modeling autumn land ecosystem variability compared to spring, due to the poorly understood processes underlying autumnal phenological events^27,28^ (Supplementary Fig. 3). Given the limitation in the simulation of AZC, the corresponding trends should be interpreted with caution and are presented here as indicative rather than definitive. For the two stations with the longest records and large-scale representativeness in the Northern Hemisphere, i.e., Barrow, Alaska (BRW; 71.3°N, 156.6°W) and Mauna Loa, Hawaii (MLO; 19.5°N, 155.6°W), the observed historical trends are as follows: SCA trends of 0.085 ppm yr^−1^ (*p* < 0.05) at BRW and 0.015 ppm yr^−1^ (*p* < 0.05) at MLO, SZC trends of −0.16 day yr^−1^ and −0.09 day yr^−1^ (both *p* < 0.05; negative values indicate an advancement in SZC), and AZC trends of −0.13 day yr^−1^ and −0.36 day yr^−1^ (both *p* < 0.05; negative values indicate an advancement in AZC), respectively. Transport simulations showed good agreement with observed SCA and SZC trends at both stations, whereas the simulated AZC trend was consistent with observations only at MLO (Fig. 1, and Supplementary Fig. 4).

**Fig. 2:**
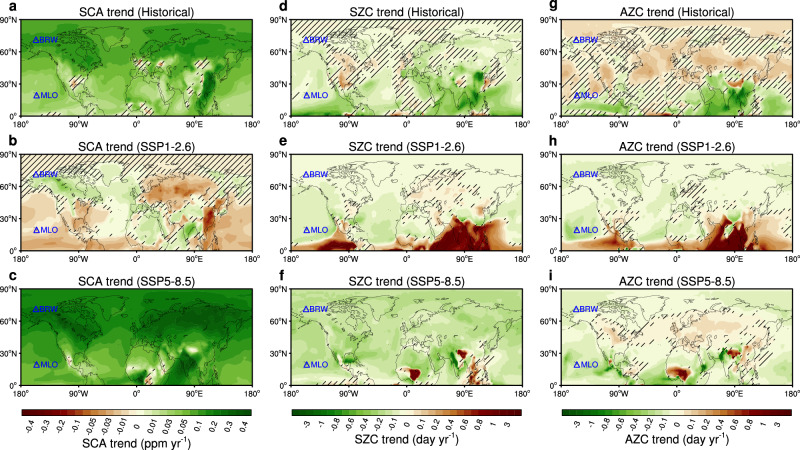
Spatial patterns of simulated trends in atmospheric CO_2_ seasonal parameters across the Northern Hemisphere under historical and future scenarios. Simulated trends in seasonal cycle amplitude (SCA; **a**–**c**), spring zero-crossing date (SZC; **d**–**f**), and autumn zero-crossing date (AZC; **g**–**i**) for the historical period (top row), SSP1-2.6 scenario (second row), and SSP5-8.5 scenario (bottom row). Hatched areas denote regions where trends are not statistically significant (*p* > 0.05). The locations of the Barrow, Alaska (BRW) and Mauna Loa, Hawaii (MLO) stations are indicated in each panel.

Future simulations encompassed four distinct CMIP6 socioeconomic trajectories: SSP1-2.6, SSP2-4.5, SSP3-7.0, and SSP5-8.5 (see Methods). Under the strongest mitigation scenario (SSP1-2.6), CO_2_ seasonal dynamics undergo a paradigmatic departure from historical patterns: BRW displays no significant trend in SCA (trend: 0.004 ppm yr^−1^; *p* > 0.1) and MLO shows a modest reduction in SCA (trend: − 0.008 ppm yr^−1^; *p* < 0.05), contrasting sharply with historical amplification trends (Fig. 1a–d). These contrasting changes reflect regional changes in CO_2_ seasonal dynamics across the boreal and northern temperate regions^9,15,29,30^. Spatially, this transformation manifests as a pronounced slowdown or reverse in SCA trends across the Northern Hemisphere (Fig. 2a, b). The boreal region, which showed the strongest historical amplification of SCA, displays contrasting responses under emission mitigation: the Canadian boreal sector maintains a modest SCA increase, while most Eurasian boreal areas exhibit significant SCA declines. These contradictory regional effects explain the statistically non-significant trend in SCA at BRW station and imply a reduced representativeness of the station over high latitudes. Furthermore, temperate regions generally show a reduction in SCA, which is consistent with the projected SCA decrease at MLO. The SCA trends increase progressively from SSP1-2.6 to SSP5-8.5 scenarios, coincident with fossil fuel emissions differences (Fig. 1a, b, and Supplementary Fig. 8). SCA maintains positive trends at the levels close to historical period under SSP2-4.5, while exhibiting significantly enlarged trends than historical levels under SSP3-7.0 (Fig. 1c, d, and Supplementary Fig. 9). The most pronounced amplification occurs under SSP5-8.5, with SCA trends reaching 0.232 ppm yr^−1^ at BRW and 0.074 ppm yr^−1^ at MLO (both *p* < 0.05) (Fig. 1c, d), manifesting a latitudinal gradient with faster increase at high latitudes and slower increase at low latitudes, and coherently show 2-3 times greater amplification than historically observed (Fig. 2c). The primary hotspots of enhanced SCA in mid-low latitudes shift from eastern China to India and Nigeria, regions projected to experience surges in fossil fuel emissions (Supplementary Fig. 10).

Across various future scenarios, the projected advancement of SZC becomes more pronounced with increasing dependence on fossil fuels. The trend of SZC advancement at BRW increases from 0.03 day yr^−1^ under SSP1-2.6 to 0.14 day yr^−1^ under SSP5-8.5; a parallel increase is also noted at MLO, where the trend increases from 0.09 to 0.16 day yr^−1^, with all trends being statistically significant (*p* < 0.05) (Fig. 1e, f). The SZC trends are more spatially uniform compared to SCA ones, and BRW and MLO are broadly representative of the boreal and northern temperate regions, respectively. Under the SSP1-2.6 scenario, SZC is slightly delayed in western Europe but slightly advanced elsewhere in the boreal and northern temperate zones (Fig. 2e). Under the SSP5-8.5 scenario, SZC advances across the most boreal and northern temperate regions, showing the greatest magnitude of advancement among all four scenarios, and exceeding that during the historical period (Fig. 2f). In contrast, the AZC trend in our simulation shows an opposite pattern across scenarios compared to that of SZC. From SSP1-2.6 to SSP5-8.5, AZC in eastern North America and central-western Europe may gradually shift from advancing to delaying (Fig. 2h, i). However, at both BRW and MLO, AZC is projected to continue to advance under all four scenarios. Under SSP1-2.6, AZC may advance at rates of approximately 0.09 day yr^−1^ at BRW and 0.15 day yr^−1^ at MLO (both *p* < 0.05), while under SSP5-8.5, the corresponding rates are 0.10 day yr^−1^ at both stations (both *p* < 0.05) (Fig. 1g, h). This indicates that, under high-emission scenarios, the AZC trends at BRW and MLO stations may become different from those across the broader boreal and northern temperate regions. The above results demonstrate that the contrasting responses of CO_2_ seasonal variations under SSP1-2.6 and SSP5-8.5 encompass the full range of potential future changes. Accordingly, our subsequent attribution analysis focuses primarily on these two scenarios.

### Attributions of trends in the seasonal amplitude of atmospheric CO_2_

**Fig. 3:**
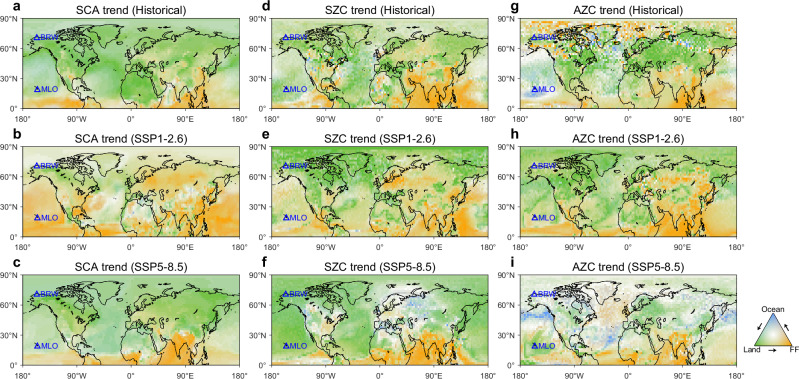
Relative contributions of net terrestrial CO_2_ fluxes, fossil fuel CO_2_ emissions, and air-sea CO_2_ fluxes to trends in atmospheric CO_2_ seasonal parameters across the Northern Hemisphere under historical and future scenarios. Relative contributions of three component fluxes, net terrestrial CO_2_ fluxes (Land, green), fossil fuel CO_2_ emissions (FF, orange) and air-sea CO_2_ fluxes (Ocean, blue), to trends in CO_2_ seasonal cycle amplitude (SCA; **a**–**c**), spring zero-crossing date (SZC; **d**–**f**), and autumn zero-crossing date (AZC; **g**–**i**) for the historical period (top row), SSP1-2.6 scenario (second row), and SSP5-8.5 scenario (bottom row). The ternary representation of the relative importance of the three component fluxes is based on the normalized absolute factorial trends associated with each flux (see Methods). The direction and spatial distribution of each factorial trend are presented separately in the corresponding maps (Supplementary Figs. 13, 23 and 24). The locations of the Barrow, Alaska (BRW) and Mauna Loa, Hawaii (MLO) stations are indicated in each panel.

The impacts of changes in net terrestrial CO_2_ fluxes, fossil fuel CO_2_ emissions, and air-sea CO_2_ fluxes on the atmospheric CO_2_ seasonal variability are assessed through the differences between our baseline simulations, where all variables are transient, and the corresponding factorial simulations, in which the relevant CO_2_ flux component is held constant throughout the simulation period using flux values from the beginning of the period (see Methods). The results show that terrestrial ecosystems or fossil fuel emissions are the primary drivers of changes in atmospheric CO_2_ seasonality in the Northern Hemisphere, with the contribution of oceans being comparatively marginal under both historical and future scenarios (Fig. 3, and Supplementary Fig. 11). Terrestrial carbon dynamics dominated the SCA trends in boreal and northern temperate regions during the historical period (Fig. 3a). However, under the SSP1-2.6 scenario, a remarkable shift in the drivers of the SCA trend is observed (Fig. 3b). In the boreal region, the contribution of terrestrial ecosystems declines substantially, and fossil fuel CO_2_ emissions become the dominant driver of the SCA trend, particularly in Eurasia. The boreal terrestrial ecosystems exhibit a modest enhancement in carbon uptake from May to August (Supplementary Fig. 12), contributing to a slight increase in SCA (Supplementary Fig. 13). By contrast, the decline in fossil fuel CO_2_ emissions exerts a much stronger influence, overwhelming the ecosystem-driven signal and propagating its impact into the broader European boreal region through atmospheric transport (Supplementary Figs. 10, 13). The mechanism underlying this shift involves changes in the seasonal profile of fossil fuel emissions. At Northern mid-high latitudes, fossil fuel emissions typically exhibit the seasonal pattern with higher emissions in winter and lower emissions in spring or summer^30–32^, which is almost synchronous with the seasonality of atmospheric CO_2_ (Supplementary Fig. 14). Consequently, the reduced fossil fuel emissions and dampened seasonality under SSP1-2.6 result in a substantial decrease in the atmospheric CO_2_ SCA (Supplementary Figs. 13). At the BRW station, the contribution of fossil fuel emissions to the variation in the CO_2_ seasonal cycle increases substantially, with the fossil fuel-driven decrease in SCA largely offsetting the increase caused by the terrestrial biosphere (Supplementary Figs. 15, 16), resulting in no significant long-term trend in SCA. This implies that it will become more challenging to apply the SCA trend at BRW to track boreal terrestrial ecosystem carbon dynamics under the SSP1-2.6 scenario.

Across the northern temperate regions, decreasing SCA trends under the SSP1-2.6 scenario are mostly driven by fossil fuel CO_2_ emissions (Fig. 3b). We confirmed that this conclusion is robust to the choice of emission seasonality (Supplementary Figs. 17–22) by running simulations using an alternative emission dataset with seasonality taken from historical MEIC inventory (see Methods). Specifically, declining fossil fuel emissions from eastern China and the eastern United States reduce the seasonal variability of these emissions, leading to a pronounced decrease in SCA (Supplementary Figs. 10, 13, 14). Terrestrial ecosystems contribute to an increase in SCA across the region, but this enhancement is generally weaker than the reduction driven by fossil fuel emissions and remains insignificant in many areas. At the site level, the decreasing trend in SCA at the MLO station is also overwhelmingly driven by fossil fuel emissions, with only minor contributions from a terrestrial ecosystem-induced decline and an ocean-driven increase (Supplementary Figs. 15, 16), making it continuously representative of the SCA trends and their underlying drivers over the northern temperate region. This result suggests that the SCA trend at MLO will closely track anthropogenic perturbations rather than terrestrial carbon dynamics under the SSP1-2.6 scenario.

Similar to the historical period, variations in terrestrial ecosystems remain the primary driver of SCA trends in boreal and northern temperate regions under the SSP5-8.5 scenario (Fig. 3c). Due to the accelerated fossil fuel emissions under SSP5-8.5, the positive fertilization effect of CO_2_ increases on vegetation productivity enhances spring-summer land carbon uptake^31–34^. The rate of this uptake increase far exceeds historical levels, resulting in a stronger amplification of SCA than previously observed (Supplementary Figs. 12, 13). Meanwhile, the increase in fossil fuel emissions also contributes to the rise in SCA, although to a much lesser extent than that driven by terrestrial ecosystems (Supplementary Fig. 13). Furthermore, SCA at both BRW and MLO stations exhibits a growth rate well above historical values, largely attributable to terrestrial ecosystem variability, indicating that these stations remain effective for monitoring changes in spring-summer carbon uptake by the land biosphere under high-emission scenarios (Supplementary Fig. 15).

### Attributions of trends in spring zero-crossing date and autumn zero-crossing date of atmospheric CO_2_

Under the SSP1-2.6 scenario, fossil fuel CO_2_ emissions exhibit greater contributions to the trends in SZC and AZC compared to the historical period, although their influence remains less dominant than that on the trend in SCA (Fig. 3d, e, g, h). Fossil fuel emissions are typically the primary driver of the SZC trend in the Eurasian sector, while terrestrial ecosystems continue to play the leading role in North America (Fig. 3e). Seasonal variability in fossil fuel emissions generally leads to a slight delay in SZC, while that in terrestrial ecosystems leads to a slight advancement (Supplementary Fig. 23). However, the SZC trend at BRW does not exhibit the delayed SZC signal associated with fossil fuel emissions, with the SZC remaining primarily advanced by terrestrial ecosystem processes (Supplementary Fig. 15). At MLO, the SZC advancement is partly due to fossil fuel emissions but mainly driven by terrestrial ecosystems (Supplementary Fig. 15). The attribution of the AZC trend resembles that of SZC (Fig. 3h), except that both fossil fuel emissions and terrestrial ecosystems generally contribute to its advancement (Supplementary Fig. 24). At both BRW and MLO, although terrestrial ecosystems contribute more substantially to the advancement of AZC, fossil fuel emissions also play a notable role in this shift (Supplementary Fig. 15). Neglecting these anthropogenic contributions could result in an overestimation of the spring and autumn variability of terrestrial ecosystems inferred from these atmospheric signals.

The earlier occurrences of SZC under the SSP5-8.5 scenario are mainly driven by terrestrial carbon dynamics (Fig. 3f). With the continuous increase in CO_2_ emissions under the high-emission scenario, the CO_2_ fertilization effect causes terrestrial ecosystems to initiate spring carbon uptake earlier and at a higher rate (Supplementary Figs. 23, 25). In contrast, changes in AZC arise from the combined influences of variations in terrestrial ecosystems, fossil fuel emissions, and oceanic processes, with terrestrial ecosystems making the largest contribution overall (Fig. 3i). However, the relative influence of ocean carbon fluxes on the AZC trend intensifies under high-emission scenarios, particularly in the mid-latitudes of the North Pacific, where ocean processes dominate the advancement of AZC. The changes in SZC and AZC simulated at the BRW and MLO stations also intuitively reflect the changes in the processes of spring carbon uptake and autumn carbon release by terrestrial ecosystems (Supplementary Fig. 15).

## Discussion

The future CO_2_ seasonal cycle is expected to evolve differently across scenarios, each governed by distinct underlying drivers. Our study reveals that under the SSP5-8.5 scenario, terrestrial ecosystems are projected to remain the primary contributor to CO_2_ seasonal variations, leading to a larger amplification in SCA and a more substantial advancement in SZC compared to the historical period. However, under the SSP1-2.6 scenario, the relative contribution of fossil fuel emissions on CO_2_ seasonal variability rises significantly, particularly by dominating declines in its seasonal amplitude—a complete reversal of their historically negligible role.

The diagnostic power of ground-based CO_2_ seasonal cycles as proxies of terrestrial carbon flux dynamics has shaped foundational paradigms in carbon-climate feedback research, from Keeling’s initial observations to contemporary earth system models (ESMs) ^1,4,5,11,14,35^. For future CO_2_ seasonal variations, previous studies have largely focused on high-emission scenarios, generally attributing the projected increase in SCA to enhanced terrestrial carbon uptake driven by continued CO_2_ fertilization^1,36,37^. Our study also confirms this pattern, and further addresses a critical gap by examining the drivers of CO_2_ seasonal variations under the mitigation scenarios, given the policy emphasis on implementing the Paris Agreement^38–41^. Particularly, under the SSP1-2.6 scenario, the global energy system undergoes a profound decarbonization transition, primarily relying on non-biomass renewables and bioenergy as the principal energy sources^16,42^. Fossil fuels are phased out, marked by a near-complete elimination of coal and substantial reductions in oil and gas use. This change is primarily concentrated in Western Europe, Eastern North America, and Eastern China (Supplementary Fig. 10)^22^. The substantial reduction in fossil fuel CO_2_ emissions, together with changes in their seasonal distribution, has altered the seasonal magnitude and phase of emissions, resulting in reduced differences between winter and summer emissions (Supplementary Figs. 18, 19). These changes in fossil fuel emissions collectively contribute to the decrease of SCA globally, consistent with findings previously reported at the regional scale^23^. Notably, this pattern remains robust even under scenarios in which the seasonality of fossil fuel emissions is substantially dampened or reversed (Supplementary Fig. 22). Meanwhile, as atmospheric CO_2_ concentrations increase more slowly and gradually stabilize, the fertilization effect weakens after mid-century, leading to a stabilization of terrestrial carbon uptake and a gradual reduction in seasonal variability^43,44^. The combination of a sharply reduced seasonal contrast in fossil fuel emissions and a relatively gradual shift in terrestrial ecosystem seasonality allows fossil fuel emissions to emerge as the dominant driver of SCA trends, marking a fundamental departure from historical attribution patterns.

As a result, the traditional use of the atmospheric CO_2_ seasonal cycle as a proxy for plant activity will likely be compromised under future low-emission scenarios like SSP1-2.6. While this presents a challenge for studying ecosystem dynamics, it also suggests that the signal is evolving. As the biospheric signal weakens, the trends of CO_2_ seasonal cycle will become increasingly influenced by fossil fuel emissions. This enhanced influence is not confined to the low-emission pathway like SSP1-2.6. Under the intermediate-emission scenario SSP2-4.5, the contribution from fossil fuel emissions increases after 2060, when emissions peak and subsequently decline sharply (Supplementary Fig. 8), thereby strengthening their imprint on the atmospheric CO_2_ seasonal signals (Supplementary Fig. 26). Ultimately, the utility of atmospheric CO_2_ seasonal cycle is not diminishing but evolving, transitioning from a proxy dominated by ecosystem fluxes toward an integrated metric capable of tracking anthropogenic emission pathways, particularly when fossil fuel emission reductions outpace changes in terrestrial ecosystem dynamics.

The robustness of our attribution rests on the relative seasonal variability of fossil fuel emissions compared with that of terrestrial carbon fluxes. Terrestrial model projections carry inherent uncertainties, particularly from nitrogen limitation treatments where models lacking explicit nitrogen cycling may overestimate vegetation responses to elevated CO_2_ conditions^45,46^. Further discrepancies are introduced by the treatment of carbon-climate feedbacks, as well as structural and parametric differences across models^47^. Importantly, under the SSP1-2.6 scenario, the relatively modest increase in atmospheric CO_2_ concentrations leads to weaker climate forcing, resulting in a more linear and predictable response of the Earth system, and consequently the projected changes in terrestrial ecosystems exhibit the lowest level of model spread among the four scenarios^43,44^ (Supplementary Fig. 27). We compared the simulated CO_2_ seasonality driven by terrestrial carbon fluxes from four representative ESMs (ACCESS-ESM1-5, CanESM5, NorESM2-LM, and MPI-ESM1-2-LR) (Supplementary Figs. 28–31), selected to span the full range of terrestrial flux variability across the ten ESMs (Supplementary Fig. 27). Despite substantial inter-model differences under high-emission scenarios, all four models consistently produced small SCA trends under SSP1-2.6 (Supplementary Figs. 28, 29). More importantly, these trends are driven by fossil fuel emissions at most regions of the Northern Hemisphere (Supplementary Fig. 32, 35). This finding confirms that under a strong mitigation pathway like SSP1-2.6, the influence of the terrestrial fluxes on atmospheric CO_2_ seasonality diminishes with high model agreement, leaving fossil fuel emissions the primary driver of the changing CO_2_ seasonal cycle (Supplementary Fig. 32–35). Despite these modeling limitations, the potential shift of the dominant fluxes in determining ground-based CO_2_ seasonal cycle variations under mitigation scenarios remains a concern that requires serious attention.

In summary, our projections reveal that the atmospheric CO_2_ seasonal cycle is not a static proxy for the terrestrial ecosystems but a dynamic signal that can be substantially influenced by anthropogenic emissions. Crucially, under strong mitigation scenario like SSP1-2.6, the fossil fuel emissions become the primary driver of SCA declines. This shift might complicate the monitoring of terrestrial ecosystem carbon dynamics using atmospheric signals in the future. Addressing this challenge requires integrating observational and modeling approaches, including the expansion of eddy covariance tower networks for direct high-frequency measurements of ecosystem carbon flux^48,49^, improvement in DGVMs to better capture seasonal dynamics and carbon-climate interactions^50,51^, and the use of atmospheric inversions that integrate CO_2_ concentration measurements with process-based estimates of terrestrial carbon flux^52–54^. Sustaining rigorous monitoring of both terrestrial carbon cycle dynamics and fossil fuel emissions is essential for verifying progress toward emission reduction targets required to constrain global warming.

## Methods

### Atmospheric CO_2_ observations

We used the weekly surface flask atmospheric CO_2_ observations from Observation Package (ObsPack) GLOBALVIEWplus v8.0 data products provided by the National Oceanic and Atmospheric Administration (NOAA). As the estimation of CO_2_ seasonal cycle can be strongly affected by the number and temporal distribution of observations within a year, we computed the Gini coefficient *G* to check the observation coverage quality^11^:

1 $$G=2\times \frac{\mathop{\sum }_{i=1}^{N}{x}_{i}\times i}{N\times \mathop{\sum }_{i=1}^{N}{x}_{i}}-\frac{N+1}{N}$$

where **x** is an increasing-ordered vector representing the number of measurements for each month *i*, and *N* is the number of months in a year (*N* = 12). Note that for one-year data aggregated into 12 months, the Gini coefficient ranges from 0 (perfect equality, identical observations every month) to a theoretical maximum of 11/12 (maximum inequality, all observations concentrated in a single month). If the combined number of years with *G* > 0.4 and years with no observations exceeds half of the observation time span, the station is discarded. Accordingly, we selected 27 out of 48 flask stations across the Northern Hemisphere that provide at least 20 years of good coverage observations (*G* ≤ 0.4) for analysis (Supplementary Table 1).

The detrended seasonal cycle of atmospheric CO_2_ for each station was extracted by a curve fitting method, CCGCRV^55^, that incorporated in a standard software provided by NOAA Earth System Research Laboratories (https://gml.noaa.gov/aftp/user/thoning/ccgcrv/). The curve fitting consists of a function fit composed of a quadratic polynomial and four harmonics to the time series, and a digital filtering of the residuals from the fit. Then a 80-days short-term cutoff and a 667-days long-term cutoff were applied to filter the residuals of the original data from the function to define interannual and short-term variations. The final detrended seasonal cycle is the annual harmonic part of the function fit plus the filtered residuals using the short-term cutoff value.

To evaluate the sensitivity of our results to the choice of detrending method, we also applied the singular spectrum analysis (SSA)^56^ to decompose the CO_2_ time series into multiple orthogonal components and to reconstruct the seasonal cycle by retaining variability within the 180–420 day frequency band. Comparisons between SSA and CCGCRV confirm that the derived trends in seasonal metrics are robust to the extraction methodology (Supplementary Figs. 5, 6) at the two representative stations, BRW and MLO. Given this consistency, we used the CCGCRV method for the primary analysis throughout the manuscript.

### Calculation of atmospheric CO_2_ seasonal parameters and their trends

In this study, we calculated three parameters of atmospheric CO_2_ seasonal cycle, including the seasonal cycle amplitude (SCA), the spring zero-crossing date (SZC), and the autumn zero-crossing date (AZC). SCA was the difference between the maximum and the minimum CO_2_ mole fractions of the seasonal cycle in each calendar year. SZC is the downward zero-crossing date of the CO_2_ seasonal cycle in spring. AZC is the upward zero-crossing date of the CO_2_ seasonal cycle in autumn. All of these seasonal parameters are illustrated in Supplementary Fig. 1. Given that our study focuses on the long-term characteristics of variations in the CO_2_ seasonal cycle, we applied an 11-year running average to the time series of SCA, SZC, and AZC^13^. Subsequently, the linear trends in SCA, SZC, and AZC were estimated using the Theil-Sen method, and their significance was evaluated using the Mann-Kendall non-parametric test. We additionally performed a sensitivity analysis using the original (unsmoothed) time series of CO_2_ seasonal parameters to estimate trends and their statistical significance. Overall, the application of a running mean has little influence on trend estimates, particularly for the long-term future scenarios that are the focus of this study (Supplementary Figs. 36, 37).

### Atmospheric transport simulations

We used the chemical transport model GEOS-Chem v12.9.3 to simulate the global 3-D atmospheric CO_2_ mole fractions at the 2°latitude × 2.5°longitude horizontal resolution with 47 layers in the vertical direction. GEOS-Chem was driven by the Modern-Era Retrospective analysis for Research and Applications, Version 2 (MERRA-2) meteorology data to transport CO_2_ fluxes from terrestrial ecosystems, fossil fuel emissions, and air-sea exchange into the atmosphere.

In this study, we focus on quantifying the contributions of net terrestrial CO_2_ fluxes, fossil fuel CO_2_ emissions, and air-sea CO_2_ fluxes to the trends in atmospheric CO_2_ seasonal cycles, both during the historical period and under various future scenarios. For the historical simulations, we designed one base transport experiment (HIS_Base) and three factorial transport experiments (HIS_Land, HIS_FF, and HIS_Ocean), all spanning 1979-2021 and initialized from a common 3-D CO_2_ field for 1 January 1979. This initial field was generated via a two-year spin-up simulation. Specifically, we first estimated an initial CO_2_ field for 1 January 1977 by taking the 1979 Copernicus Atmosphere Monitoring Service (CAMS) inversion (https://ads.atmosphere.copernicus.eu/datasets/cams-global-greenhouse-gas-inversion?tab=overview) data and subtracting the observed 1977-1978 global CO_2_ growth rates from NOAA Global Monitoring Laboratory from all model grid cells. We then ran GEOS-Chem for the years 1977-1978, using recycled 1979 CAMS fluxes that were scaled globally to match the Global Carbon Budget 2022^57^. The resulting 3-D CO_2_ field on 1 January 1979 served as our final, dynamically consistent restart file. In HIS_Base, the transport was driven by historically varying net terrestrial CO_2_ fluxes, fossil fuel CO_2_ emissions, air-sea CO_2_ fluxes, and meteorological fields, aiming to approximate the real evolution of atmospheric CO_2_. HIS_Land used time-varying fossil fuel emissions and air-sea CO_2_ fluxes but fixed net terrestrial CO_2_ fluxes at 1979 levels, while HIS_FF and HIS_Ocean similarly held fossil fuel and oceanic fluxes constant, respectively, with other components varying temporally.

To assess the potential future contributions of the component fluxes, we conducted simulations spanning from 2030 to 2100 under four distinct CMIP6 socioeconomic scenarios: SSP1-2.6, SSP2-4.5, SSP3-7.0, and SSP5-8.5. Under each scenario, we performed four transport experiments, namely CMIP6_Base, CMIP6_Land, CMIP6_FF, and CMIP6_Ocean, all initialized with a common CO_2_ field for 1 January 2030. This initial field was generated by running the model from the end of our historical simulation (31 December 2021) to 1 January 2030, driven by an annually repeating cycle of mean fluxes derived from the 2012-2021 historical period. In CMIP6_Base, all the three CO_2_ fluxes varied with time, with meteorological fields held constant at the 2021 value. In CMIP6_Land, CMIP6_FF, and CMIP6_Ocean, net terrestrial CO_2_ fluxes, fossil fuel CO_2_ emissions, and air-sea CO_2_ fluxes were fixed at the 2030 values, respectively, with the other two fluxes varied with time. To assess the potential impact of changing atmospheric circulation, we additionally performed a suite of sensitivity simulations driven by time-varying, scenario-specific meteorological fields from the CanESM5 and NorESM2-LM models (Supplementary Text 1). These tests confirm that the projected trends in CO_2_ seasonality (Supplementary Figs. 38–41) and their attribution (Supplementary Figs. 42–49) are robust to the choice of meteorological forcing.

We also assessed the impact of carbon-climate feedbacks on the simulation of CO_2_ seasonality trends, by performing targeted comparison using the terrestrial carbon fluxes from esm-ssp585 experiment in the Coupled Climate-Carbon Cycle Model Intercomparison Project (C4MIP), which includes such feedbacks^58^. ACCESS-ESM1-5, CanESM5, MPI-ESM1-2-LR, and NorESM2-LM are the models that participated in both the Scenario Model Intercomparison Project (ScenarioMIP) SSP5-8.5 and C4MIP esm-ssp585 experiments (Supplementary Figs. 50, 51). We examined the spatial patterns of trends in SCA, SZC and AZC from the native CO_2_ concentration output of the coupled models. Despite differences in atmospheric transport physics, the seasonality trends remain broadly consistent between these coupled simulations and those simulated by GEOS-Chem using SSP5-8.5 fluxes (Supplementary Fig. 52). This confirms that our primary analysis is sufficient to capture the dominant drivers of future atmospheric CO_2_ seasonality.

The factorial trends of SCA, SZC, and AZC caused by each component flux were assessed through the difference between the base simulation and the corresponding factorial simulation. Taking the fossil fuel-driven SCA trend as an example, we subtracted the SCA time series of the CMIP6_FF from the CMIP6_Base. Fossil fuel emissions’ contribution to the SCA trend was then defined as the linear trend of this difference time series, estimated using the Theil-Sen method. Since these factorial trends can be positive or negative, we quantified their relative importance using a ternary diagram. Because a ternary diagram requires all components to be non-negative and to sum to unity, we used the absolute value of each factorial trend, normalized by the sum of the absolute values of all three factorial trends, to quantify their relative importance (i.e., which factor is the strongest driver of change). The signed magnitude (increase or decrease) of each factorial trend is provided separately in the corresponding maps (e.g., Supplementary Figs. 13, 23 and 24). As a complementary analysis, we applied a vector projection method^4^ to quantify the relative contribution of each flux component to the overall shape of the annual CO_2_ seasonal cycle pattern within one year. The contribution fraction of flux *i* is calculated as:

2 $${C}_{m}=\mathop{\sum}_{i}{C}_{{mi}}\,{{{\rm{and}}}}\,{y}_{i}=\frac{{\sum}_{m}{C}_{{mi}}{C}_{m}}{{\sum}_{m}{C}_{m}^{2}}$$

where ${C}_{{mi}}$ is the concentration in month *m* contributed by flux component *i* (where ${C}_{{mi}}$ has been detrended and the annual mean removed, i.e., ${\sum}_{m}{C}_{{mi}}=0$). ${C}_{m}$ is the sum of the concentration from all flux components in month *m* and ${y}_{i}$ is the contribution to the seasonal cycle from flux *i*, expressed as a fraction of the total cycle. Summing ${y}_{i}$ over all fluxes adds to 1, but individual ${y}_{i}$ can be negative. This approach yields an assessment of how the relative influence of each flux component on the CO_2_ seasonal cycle within a year evolves over time.

### Historical surface CO_2_ fluxes

We used the multi-model ensemble mean of monthly net biome productivity (NBP) from 14 DGVMs (Supplementary Table 2) in the TRENDYv11 project^51^ to characterize the historical variations in land-atmosphere CO_2_ exchange from 1979 to 2021. Specifically, we employed the S3 simulations, which incorporate all forcings evolving over the historical period, including time-varying global annual mean atmospheric CO_2_ concentrations from the combination of ice core CO_2_ data^56^ and NOAA CO_2_ observations (http://www.esrl.noaa.gov/gmd/ccgg/trends/), CRUJRA 6-h climate forcing data from merging monthly Climate Research Unit gridded Time Series (CRU TS) data^59^ with 6-h Japanese 55-year Reanalysis (JRA-55) data^60^, and land use change from History Database of the Global Environment v3.3 (HYDE 3.3)^61^ and Land Use Harmonization v2 model (LUH2-GCB)^62^.

The global fossil fuel CO_2_ emissions from Multi-resolution Emission Inventory model for Climate and air pollution research (MEIC)^63^ spanning the period between 1979 and 2021 were used in this study. MEIC dataset was highly-resolved (0.1°×0.1°) and dynamically updated with detailed sectors, fuel types, and sub-country information. The temporal resolution of MEIC data was daily, which can better capture the seasonality of fossil fuel CO_2_ emissions compared with the traditional monthly emission datasets.

The historical air-sea CO_2_ fluxes were derived from the global ocean biogeochemistry model NEMO-PlankTOM12^64^, which is coupled with the global ocean general circulation model Nucleus for European Modeling of the Ocean version 3.6 (NEMOv3.6) and the biogeochemistry model Plankton Type Ocean Model version 12 (PlankTOM12)^65^. The air-sea CO_2_ flux data were provided at a monthly resolution with a spatial grid of 1° × 1°.

Due to the resolution mismatch between the original flux data and the transport simulations, we applied first-order conservative remapping using the Climate Data Operators (CDO) software to interpolate the flux data onto the 2° × 2.5° (latitude × longitude) grid of GEOS-Chem.

### CMIP6 future surface CO_2_ fluxes

We used fossil fuel CO_2_ emissions, net terrestrial CO_2_ fluxes, and air-sea CO_2_ fluxes under four different shared socio-economic pathways (SSPs) from CMIP6. The four SSPs we chose were SSP1-2.6, SSP2-4.5, SSP3-7.0, and SSP5-8.5, which represent the low end, medium part, medium to high end, and high end of the range of future forcing pathways, respectively.

In CMIP6, the historical forcings are based as far as possible on observations and cover the period from 1850 to 2014. For the future scenarios, forcings are provided by the integrated assessment model (IAM) community for the period from 2015 to 2100^66^. Future fossil fuel CO_2_ emissions are provided from 2015 and at decadal intervals from 2020 to 2100, at a spatial resolution of 0.5° × 0.5° and with monthly temporal resolution^22,67^. These projections are generated by downscaling annual, sector-resolved national emissions from IAMs using 2014 country- and sector-specific spatial proxies, primarily sourced from EDGAR v4.2. Monthly variability in these gridded emissions is prescribed using spatially explicit seasonality fractions primarily derived from the ECLIPSE dataset, supplemented by EDGAR v4.3 for international shipping and by Lamarque et al. (2010)^68^ for aviation. We generated annual emission projections from 2030 to 2100 by applying linear temporal interpolation to the available decadal data. To evaluate the sensitivity of our results to uncertainties in the seasonality of fossil fuel CO_2_ emissions, we constructed three alternative emission datasets (Supplementary Fig. 19). In the first case, sector-specific monthly profiles averaged over 2017–2021 from the MEIC global inventory were applied to the CMIP6 annual sector-specific emissions at each grid cell. We further designed two idealized experiments to explicitly perturb the seasonal structure of emissions beyond historically observed patterns. In one experiment, emission seasonality gradually transitions toward a uniform monthly distribution by 2100, thereby eliminating the seasonal cycle. In the other, emission seasonality is progressively reversed relative to the 2030 baseline, representing a pronounced phase shift. These three constructed future fossil fuel CO_2_ emission datasets are intended primarily as an uncertainty-bounding analysis rather than as realistic projections of future emission patterns. The resulting emissions were used to drive transport simulations CMIP6_Base and CMIP6_FF under four future scenarios. The results confirm that the projected seasonal cycle parameters are robust to these differences in prescribed emission timing (Supplementary Figs. 20–22).

For net terrestrial and air-sea CO_2_ fluxes, we used the ensemble mean estimates from ten ESMs participating in ScenarioMIP^69^ under four scenarios (Supplementary Table 3). The future net terrestrial and ocean CO_2_ fluxes for 2030–2100 from different ESMs are provided at a monthly resolution, though their spatial resolution varies. All the data were resampled to the 2°latitude × 2.5°longitude resolution to obtain the ensemble mean fluxes. In addition, to quantify the uncertainty in future simulations of the atmospheric CO_2_ seasonal cycle and its attribution arising from uncertainties in projected terrestrial CO_2_ fluxes, we conducted transport simulations driven by terrestrial CO_2_ fluxes from a representative subset of four ESMs: ACCESS-ESM1-5, CanESM5, NorESM2-LM and MPI-ESM1-2-LR. These models were selected because they collectively span the full range of NBP variability within the 10-model ensemble (Supplementary Fig. 27). For each model, we performed baseline and land factorial simulations across all four future scenarios.

## Supplementary information

Supplementary Information

Transparent Peer Review file

## Data Availability

The ObsPack atmospheric CO_2_ observations are available at https://gml.noaa.gov/ccgg/obspack/. The DGVM simulations from TRENDYv11 are available upon request from Stephen Stich (s.a.sitch@exeter.ac.uk). The air-sea CO_2_ fluxes from NEMO-PlankTOM12 are available at https://zenodo.org/records/7273309. The fossil fuel CO_2_ emissions from MEIC are available upon request from Qiang Zhang (qiangzhang@tsinghua.edu.cn). The NBP, fossil fuel CO_2_ emissions, and air-sea CO_2_ fluxes from CMIP6 are available at https://aims2.llnl.gov/search/cmip6/. The CO_2_ seasonal cycle parameters generated in this study have been deposited in the Zenodo database (https://zenodo.org/records/17035880). Any additional information may be obtained from the corresponding author upon request.

## Code availability

The scripts used for data processing and figure generation are available via Zenodo at (https://zenodo.org/records/17035880). The code of GEOS-Chem is available at (https://geoschem.github.io/).
