# Supplementary material for: Fossil fuel emissions dominate Northern Hemisphere CO2 seasonal cycle trends under mitigation scenarios
Source: Nat Commun. 2026 Jul 1;17:8201. doi: 10.1038/s41467-026-75003-x (PMC13462122; doi:10.1038/s41467-026-75003-x)
Supplement: Supplementary file 1 [file 41467_2026_75003_MOESM1_ESM.pdf]

Supplementary Information for

Fossil fuel emissions dominate Northern Hemisphere CO_2 seasonal cycle trends under mitigation scenarios

Jin et al.

This file includes:

Supplementary Text 1, Supplementary Figures 1-52, and Supplementary Table 1-5, and Supplementary References

Supplementary Text 1. Sensitivity of CO_2 Seasonal Cycle Projections to Future Meteorological Forcing

To evaluate the impact of future atmospheric circulation changes on CO_2 seasonal cycle, we performed a suite of sensitivity experiments using CMIP6 scenario-specific, time-varying meteorological data. First, we identified five key variables that dominate CO_2 atmospheric transport: surface pressure, planetary boundary layer height, zonal wind, meridional wind, and specific humidity. Among the ten ESMs that provided terrestrial and ocean CO_2 fluxes for all scenarios, only five (CanESM5, NorESM2-LM, NorESM2-MM, INM-CM4.8, and INM-CM5.0) provided all five of these meteorological variables (Supplementary Table 4). From this subset, we selected CanESM5 and NorESM2-LM as two representatives. Because CMIP6 data have coarser temporal and vertical resolution than the standard GEOS-Chem input (Supplementary Table 5), we performed piecewise linear interpolation to map the CMIP6 fields from their native pressure levels onto the 72-layer GEOS-Chem hybrid sigma-pressure grid. For layers below 1000 hPa or above 10 hPa, values from 1000 hPa or 10 hPa were used, respectively. Similar to the main experiments, we ran the baseline simulation (CMIP6_Base) and three factorial simulations (CMIP6_Land, CMIP6_FF, and CMIP6_Ocean) using the meteorological data from these two models for each scenario.

The results demonstrate that the projected trends in CO_2 seasonality are robust to the choice of meteorological forcing. Both at the BRW and MLO stations (Supplementary Fig. 33) and across the Northern Hemisphere (Supplementary Figs. 34-36), the simulated trends in SCA, SZC, and AZC during 2030-2100 under all future scenarios are largely consistent between simulations driven by constant 2021 MERRA-2 meteorological fields and those driven by time-varying meteorology from CanESM5 and NorESM2-LM. In addition, the spatial attribution patterns remain consistent (Supplementary Figs. 37-44). Under SSP1-2.6, fossil fuel emissions continue to dominate the SCA decline (Supplementary Figs. 39, 40), while under SSP5-8.5, terrestrial ecosystems remain the primary driver of changes in CO_2 seasonal cycle

((Supplementary Figs. 37, 38)).

These findings indicate that variations in future meteorological fields exert a minimal effect on the long-term trends of CO_2 seasonality compared to the dominant influence of changing surface fluxes.

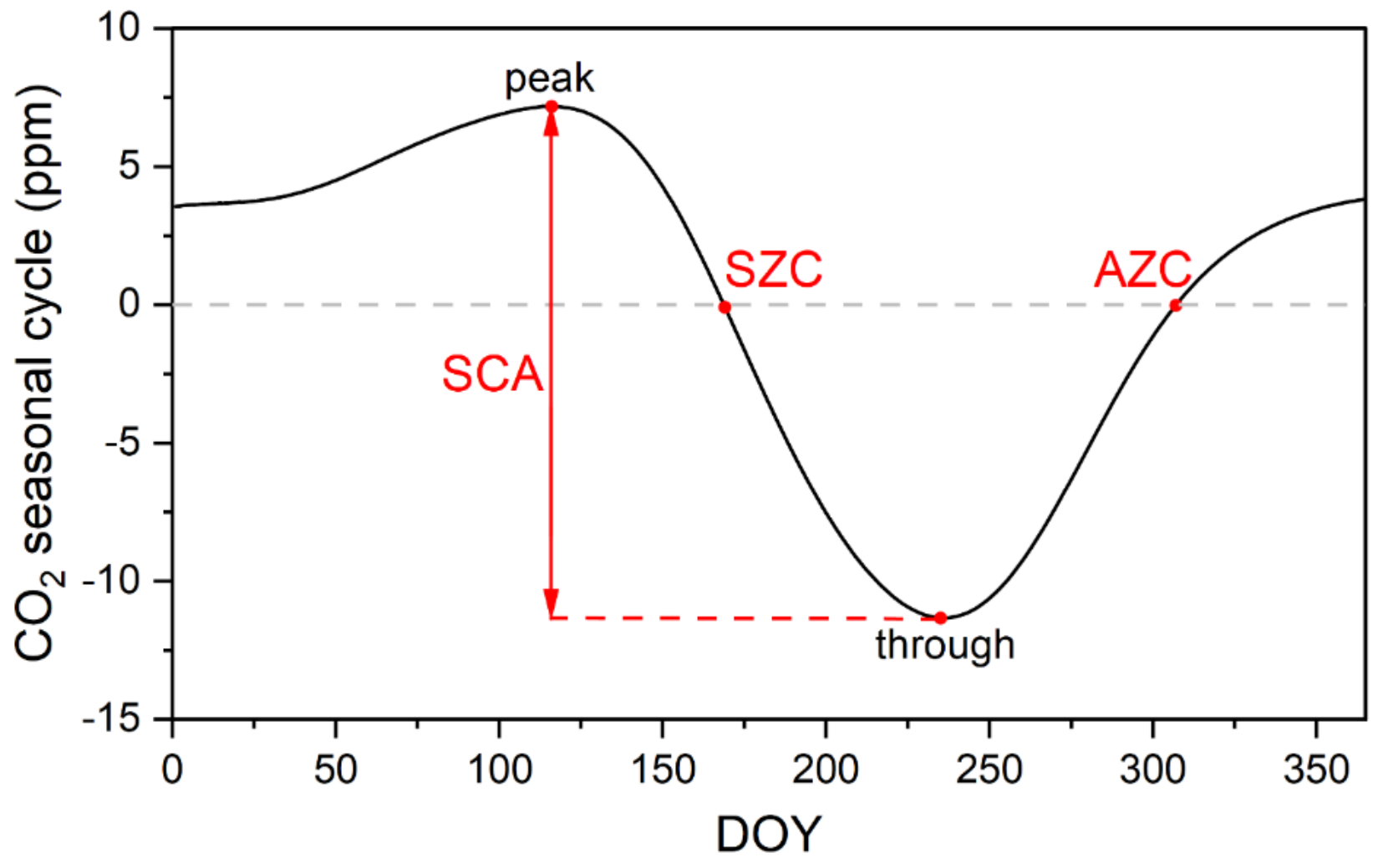

Supplementary Figure 1. Illustration of the three parameters of CO_2 seasonal cycle used in this study, including SCA, SZC, and AZC. The detrended CO_2 seasonal cycle here is the seasonal cycle at Barrow, Alaska in 2021.

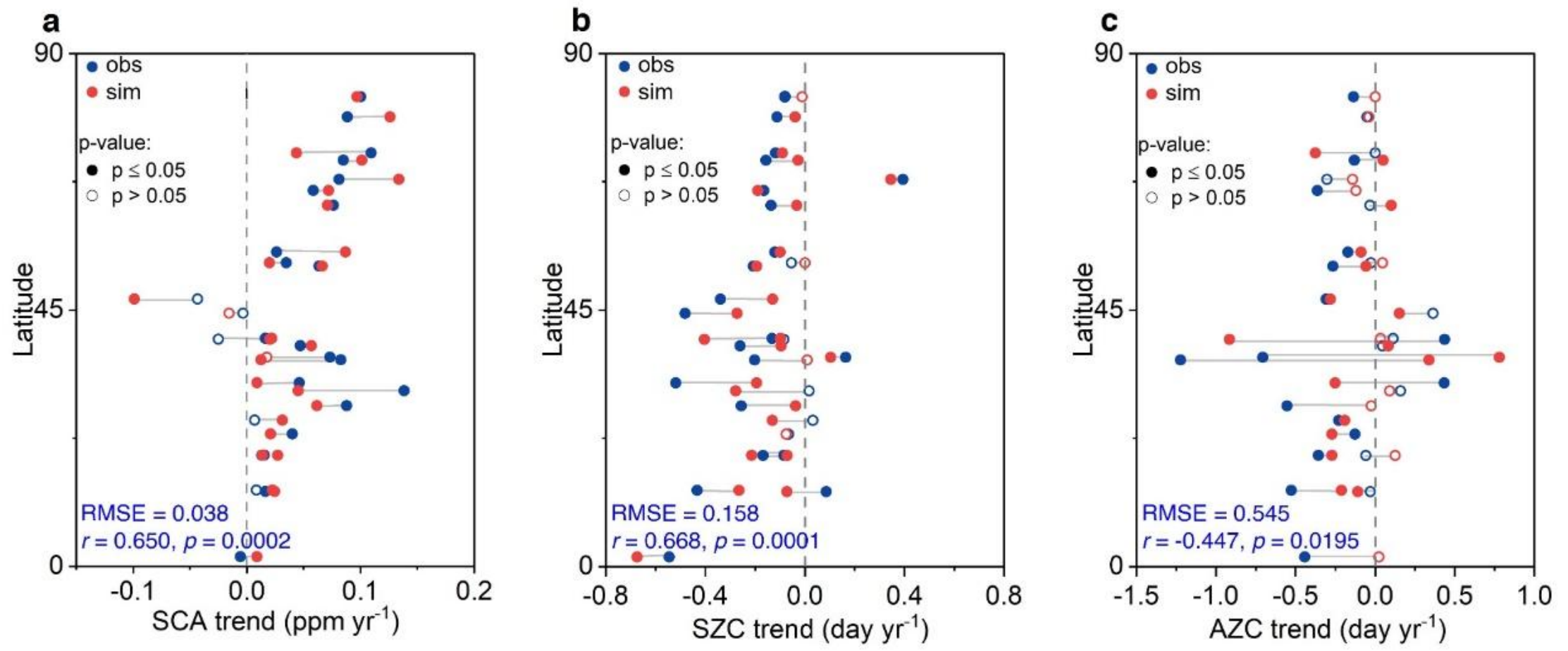

Supplementary Figure 2. Latitudinal distributions of observed and HIS_Base simulated trends in SCA (**a**), SZC (**b**), and AZC (**c**) at 27 surface stations across the Northern Hemisphere during 1979–2021. For all scatters, the solid symbol indicates that it passes significance test ($p\leq0.05$), while the hollow symbol indicates that it fails the significance test ($p> 0.05$). For each panel, the root mean square error (RMSE) between the observed and simulated values, together with the Pearson correlation coefficient and the corresponding significance test, is reported in the lower-left corner.

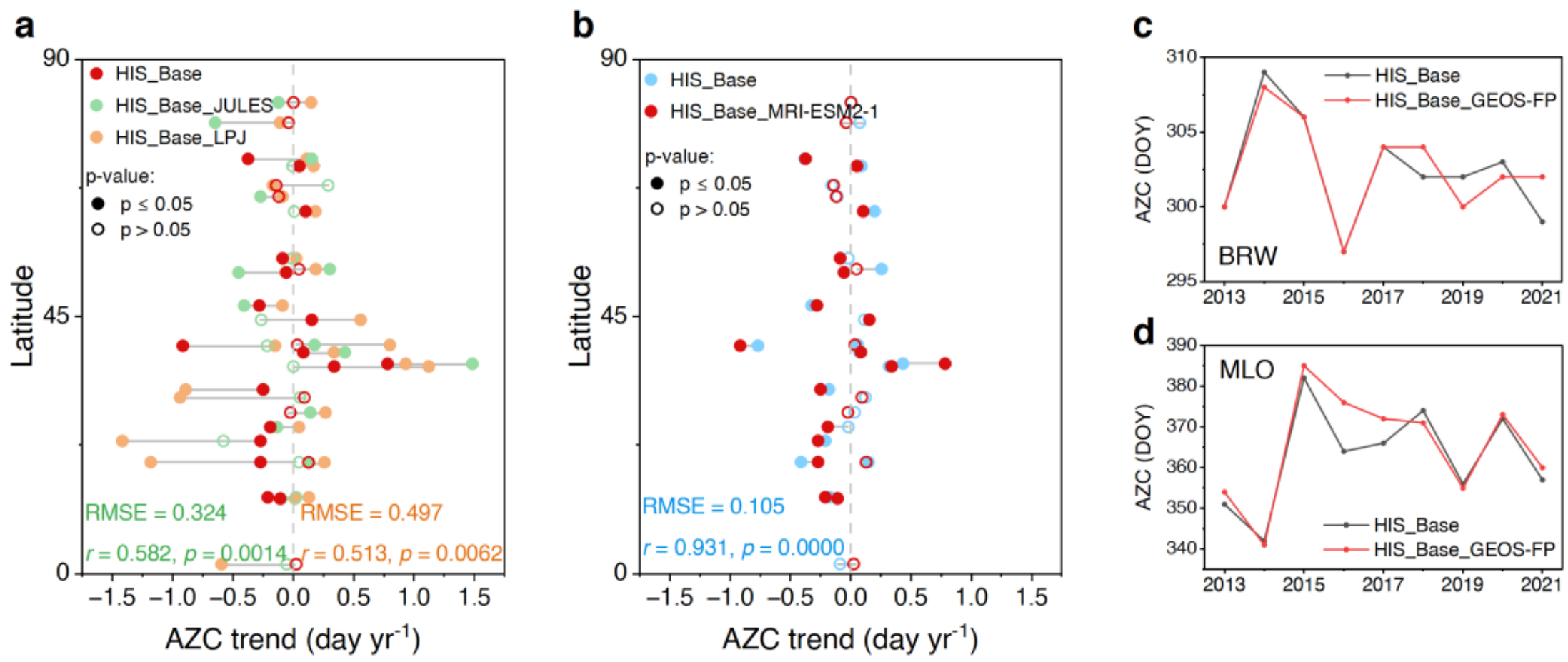

Supplementary Figure 3. Latitudinal distributions of simulated trends in AZC at 27 Northern Hemisphere surface stations during 1979-2021 (**a, b**), and time series of AZC at the BRW and MLO stations for 2013-2021 (**c, d**). Panel (a) shows the AZC trends from the baseline simulation (HIS_Base) and from sensitivity simulations in which NBE was replaced by that derived from the JULES (HIS_Base_JULES) or LPJ (HIS_Base_LPJ) DGVMs. Panel (b) shows the AZC trends from HIS_Base and from a sensitivity simulation in which the air-sea CO_2 flux was replaced by that from the MRI-ESM2-1 ocean model (HIS_Base_MRI-ESM2-1). Panels (c) and (d) show the 2013-2021 AZC time series at BRW and MLO, respectively, from HIS_Base and from a sensitivity simulation driven by GEOS-FP meteorological fields (HIS_Base_GEOS-FP). Because GEOS-FP meteorological fields are available only from April 2012 onward, the time series are shown starting in 2013. For all scatters, the solid symbol indicates that it passes the significance test ($p \leq 0.05$), while the hollow symbol indicates that it fails the significance test ($p > 0.05$). For panels (a) and (b), the root mean square error (RMSE) between the AZC trends from the baseline and sensitivity simulations, together with the Pearson correlation coefficients and the corresponding significance tests, are reported at the bottom of the figure.

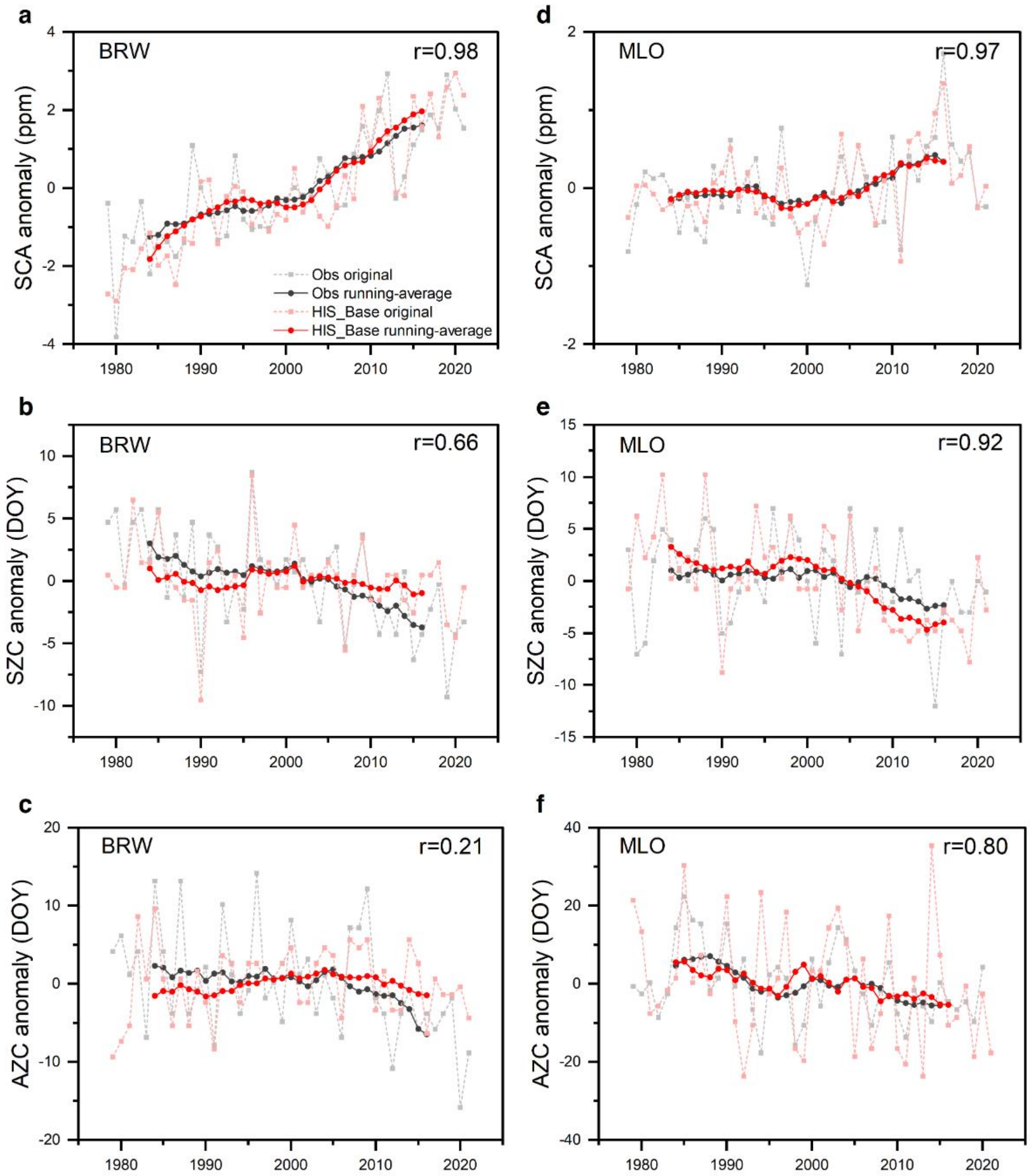

Supplementary Figure 4. Time series of observed and HIS_Base simulated SCA, SZC and AZC anomalies at BRW (**a-c**) and MLO (**d-f**) stations from 1979 to 2021. The upper-right numbers in each plot denote the correlation coefficient between the 11-yr running averaged observed and simulated seasonal parameters.

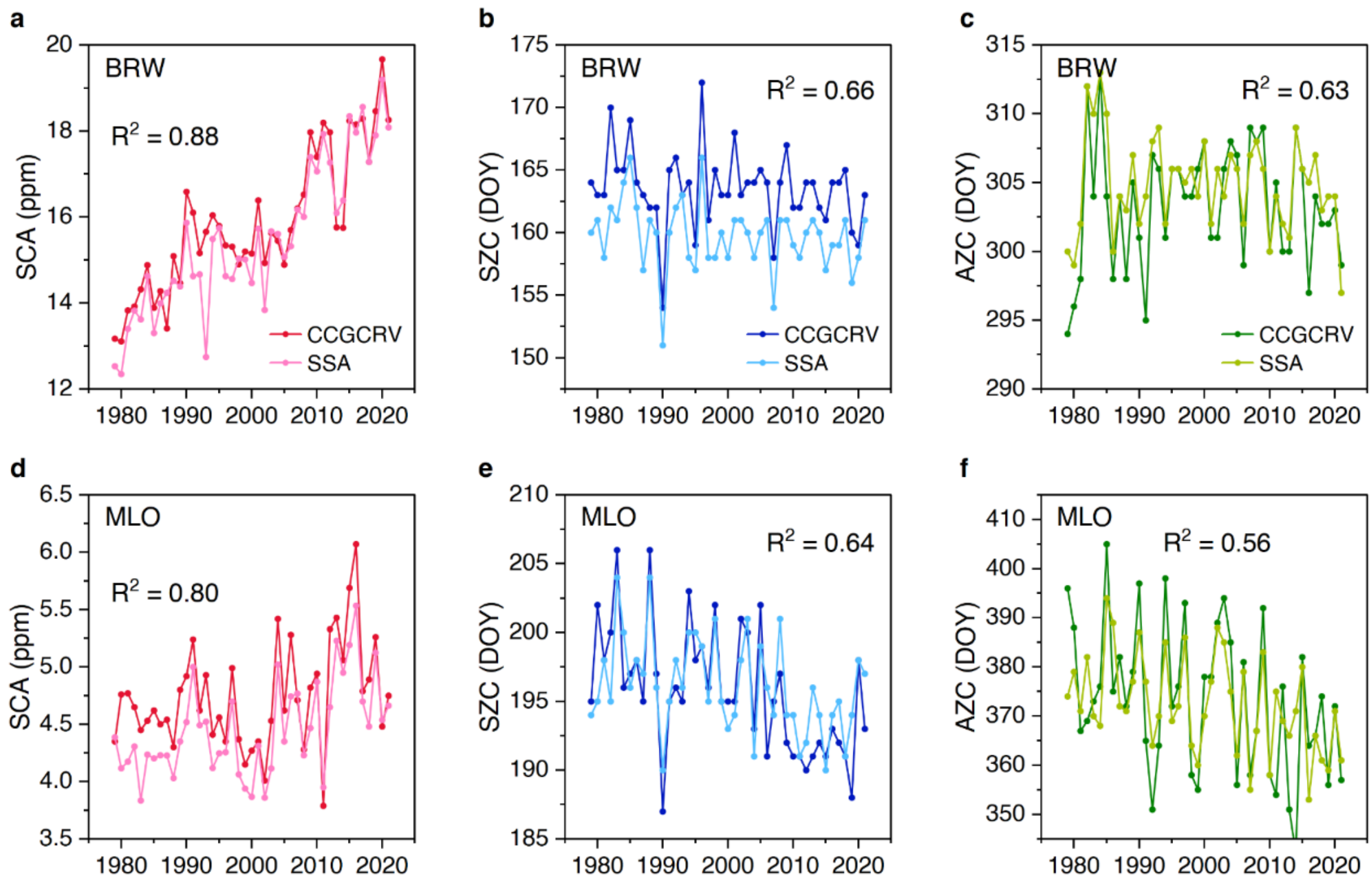

Supplementary Figure 5. Key metrics of CO_2 seasonal cycle derived using CCGCRV and SSA at the BRW and MLO stations during 1979-2021. Shown are comparisons of SCA (**a, d**), SZC (**b, e**), and AZC (**c, f**) from the historical baseline simulation (HIS_Base) at BRW (top row) and MLO (bottom row).

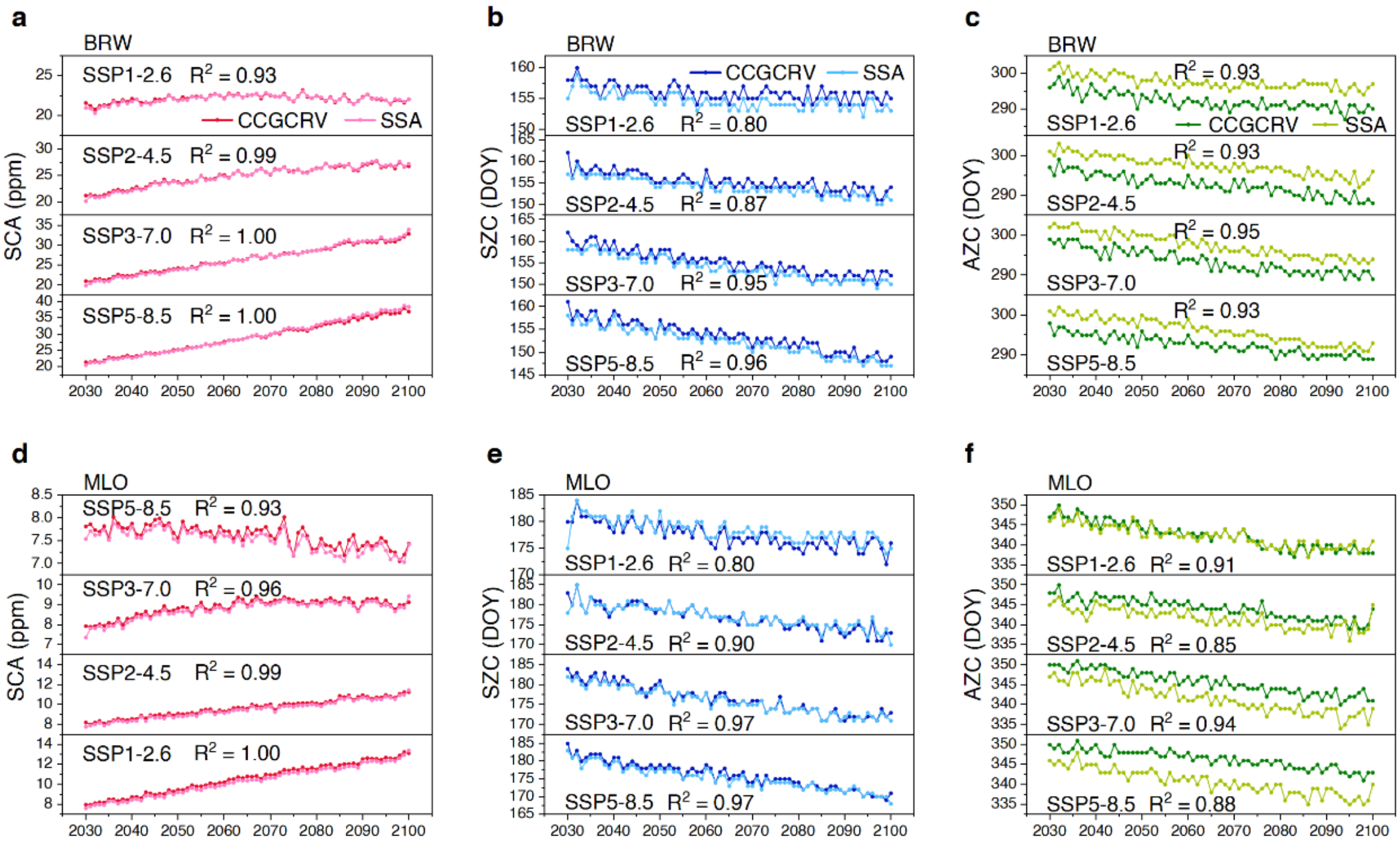

Supplementary Figure 6. Key metrics of CO_2 seasonal cycle derived using CCGCRV and SSA at the BRW and MLO stations under different scenarios for 2030-2100. Shown are comparisons of SCA (a, d), SZC (b, e), and AZC (c, f) from the CMIP6 baseline simulations (CMIP6_Base) at BRW (top row) and MLO (bottom row).

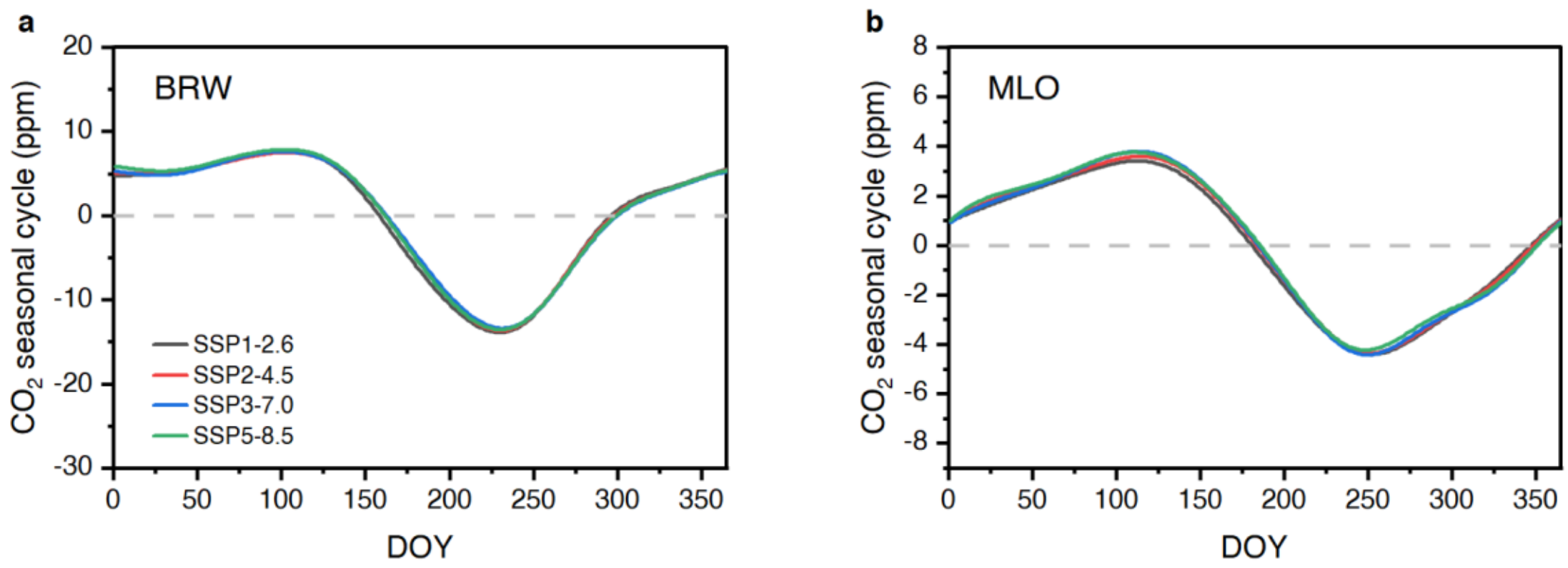

Supplementary Figure 7. Detrended seasonal cycles of atmospheric CO_2 simulated for 2030 under the SSP1-2.6, SSP2-4.5, SSP3-7.0, and SSP5-8.5 scenarios at BRW (**a**) and MLO (**b**) stations.

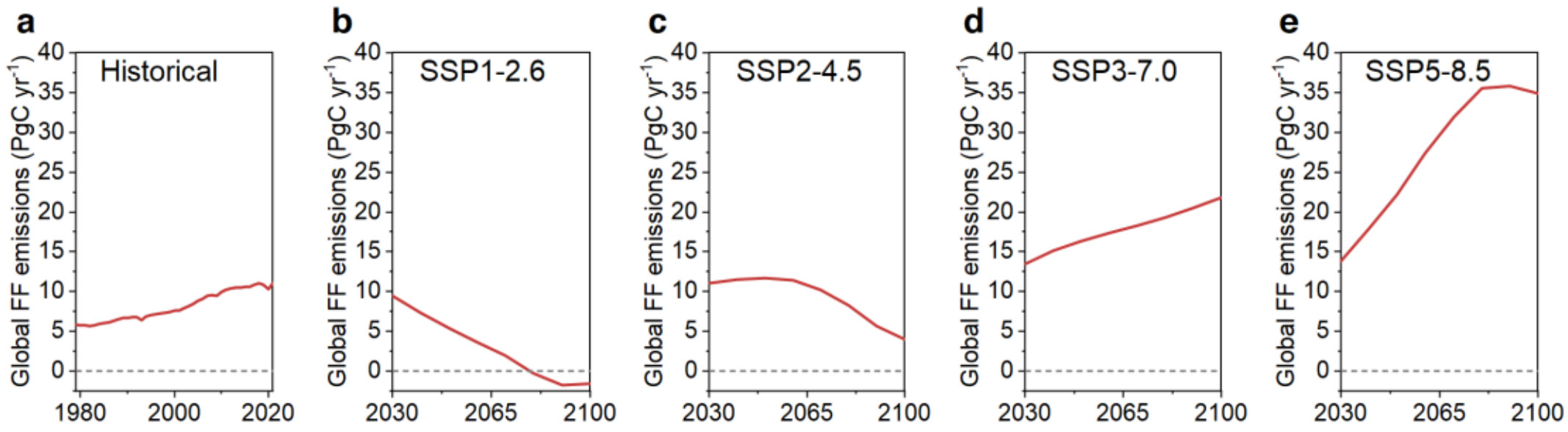

Supplementary Figure 8. Global fossil fuel CO_2 emissions under historical and future scenarios: historical period (**a**), SSP1-2.6 (**b**), SSP2-4.5 (**c**), SSP3-7.0 (**d**), and SSP5-8.5 (**e**) scenarios.

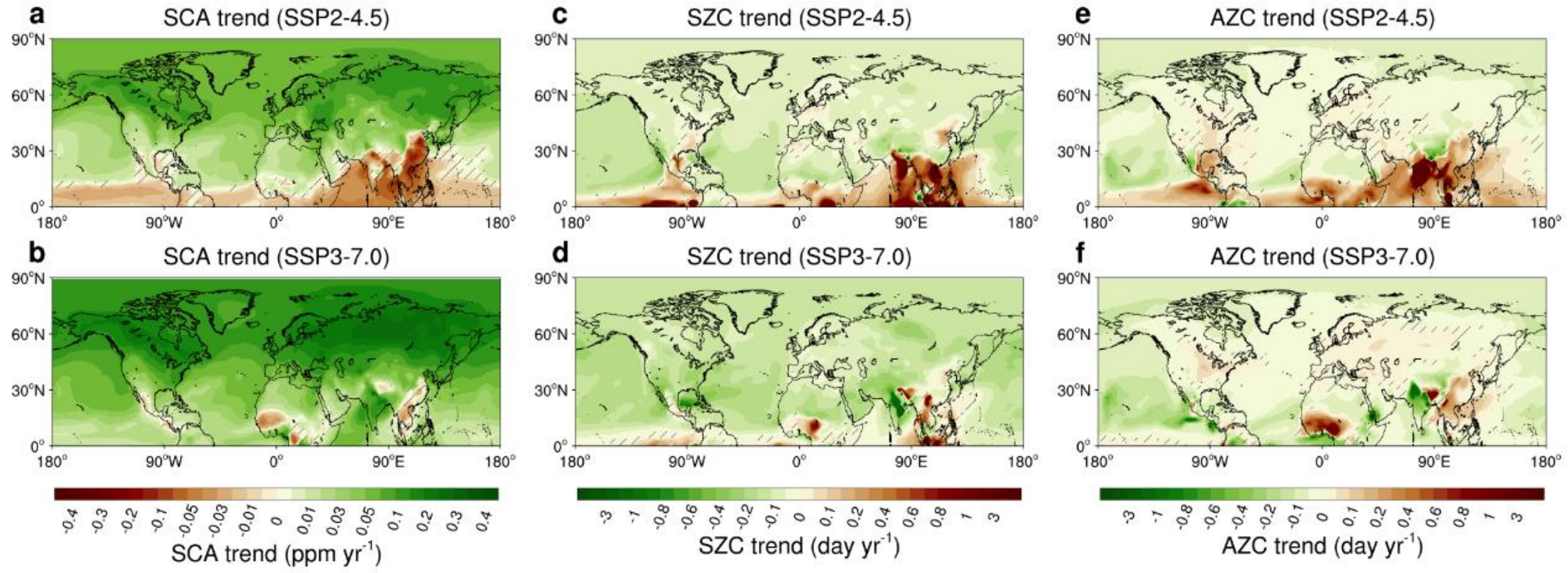

Supplementary Figure 9. Spatial patterns of simulated trends in atmospheric CO_2 seasonal parameters across the Northern Hemisphere for the future scenarios. Simulated trends in SCA (**a, b**), SZC (**c, d**), and AZC (**e, f**) under the SSP2-4.5 (top row) and SSP3-7.0 (bottom row) scenarios. The hatched areas indicate that the trends in these regions did not pass the significance test ($p > 0.05$).

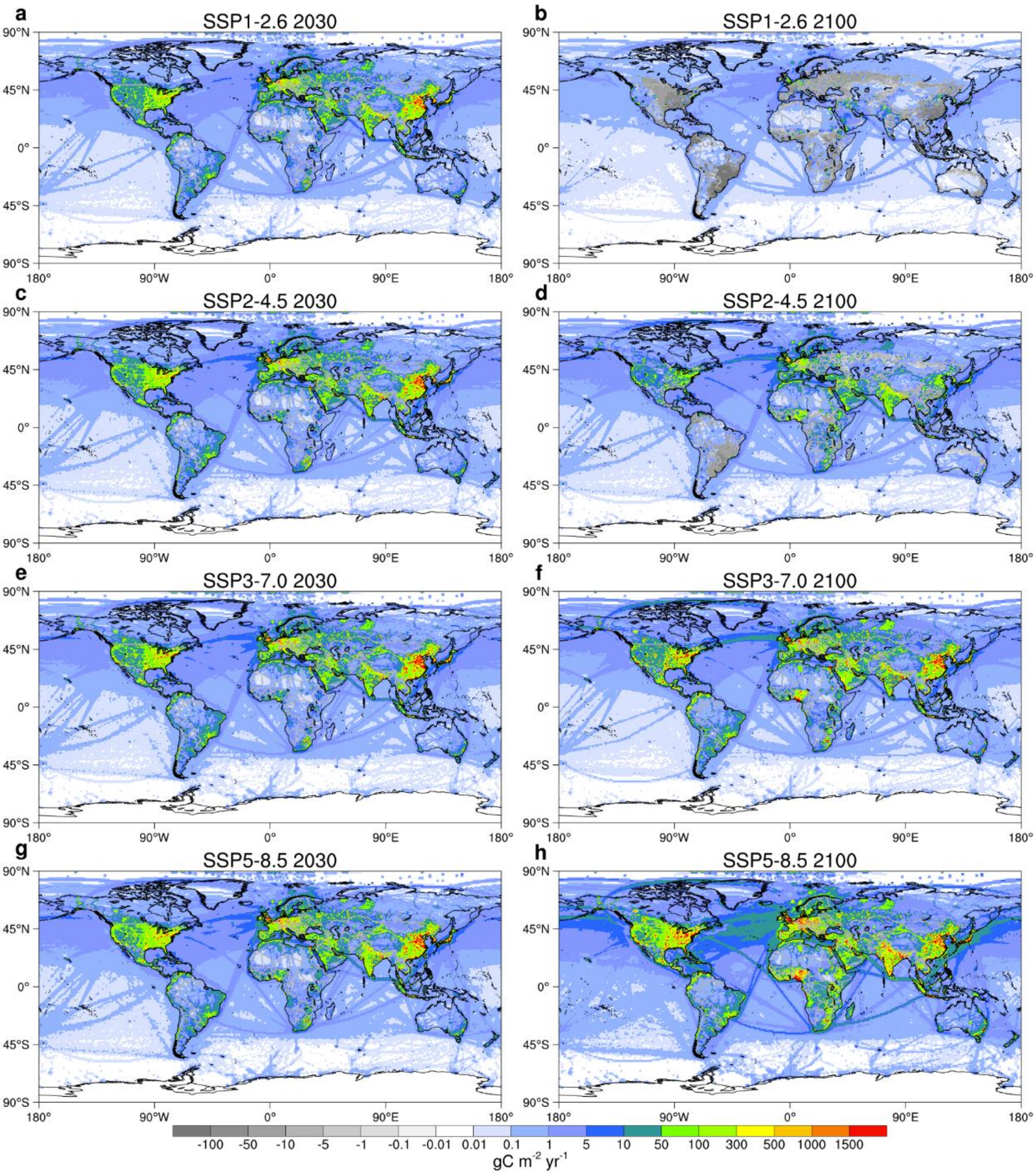

Supplementary Figure 10. Projected global fossil fuel CO_2 emission distributions for 2030 (left panels) and 2100 (right panels) under SSP1-2.6 (**a, b**), SSP2-4.5 (**c, d**), SSP3-7.0 (**e, f**), and SSP5-8.5 (**g, h**) scenarios.

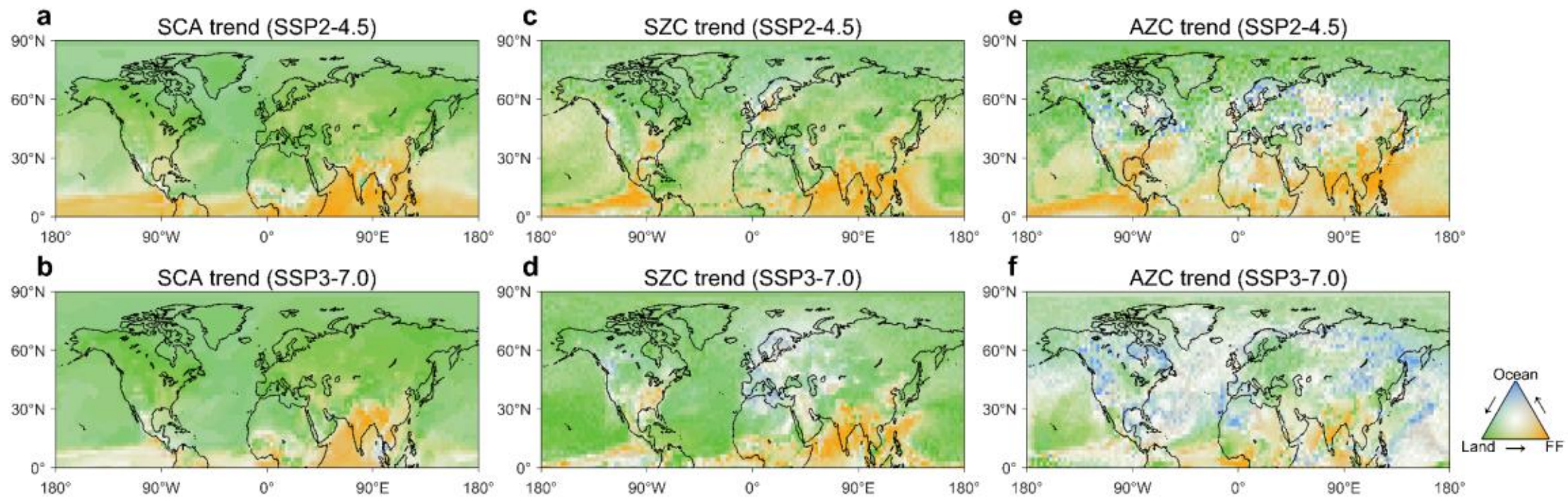

Supplementary Figure 11. Relative contributions of net terrestrial CO_2 fluxes, fossil fuel CO_2 emissions, and air-sea CO_2 fluxes to trends in SCA (**a, b**), SZC (**c, d**), and AZC (**e, f**) across the Northern Hemisphere under the SSP2-4.5 (top row) and SSP3-7.0 (bottom row) scenarios.

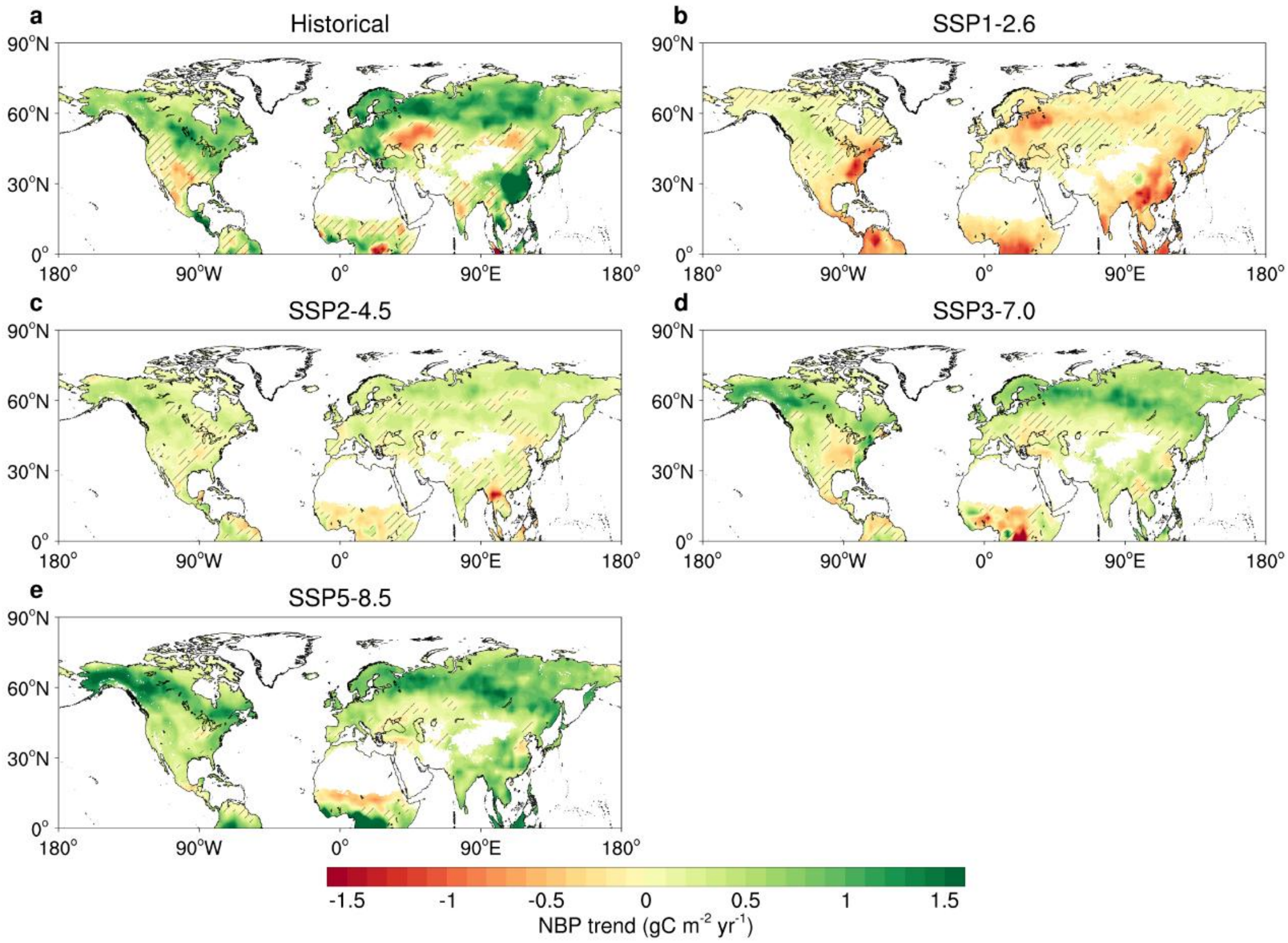

Supplementary Figure 12. Trends in integrated NBP from May to August in the Northern Hemisphere under historical (**a**), SSP1-2.6 (**b**), SSP2-4.5 (**c**), SSP3-7.0 (**d**), and SSP5-8.5 (**e**) scenarios. The hatched areas indicate that the trends in these regions did not pass the significance test ($p > 0.05$).

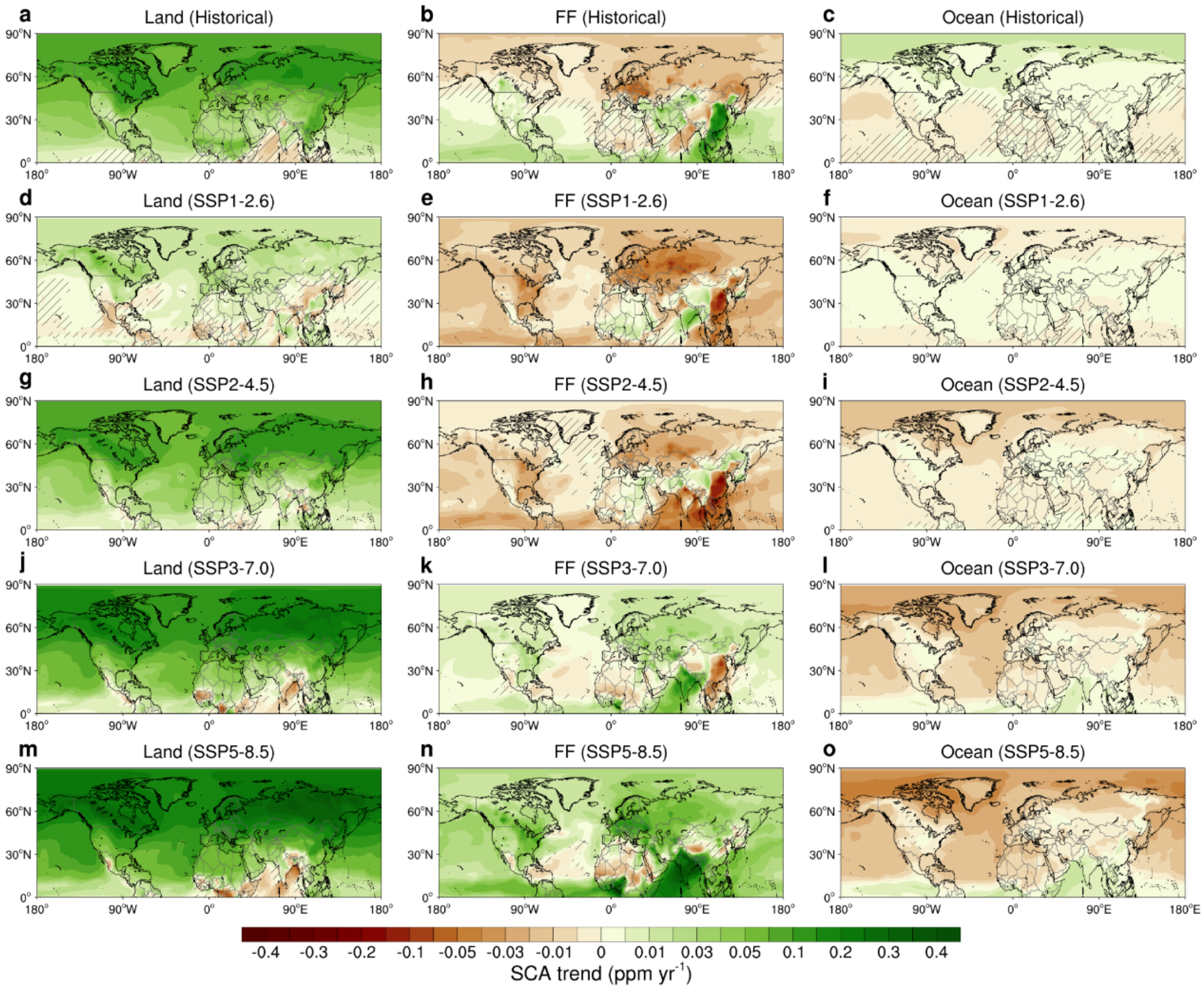

Supplementary Figure 13. SCA trends attributed to variations in the terrestrial ecosystem (left panels), fossil fuel CO_2 emissions (middle panels), and air-sea CO_2 exchange (right panels) during the historical period (**a-c**) and under the SSP1-2.6 (**d-f**), SSP2-4.5 (**g-i**), SSP3-7.0 (**j-l**), and SSP5-8.5 (**m-o**) scenarios, estimated from the differences between base simulations and the corresponding factorial simulations. The hatched areas indicate that the trends in these regions did not pass the significance test ($p > 0.05$).

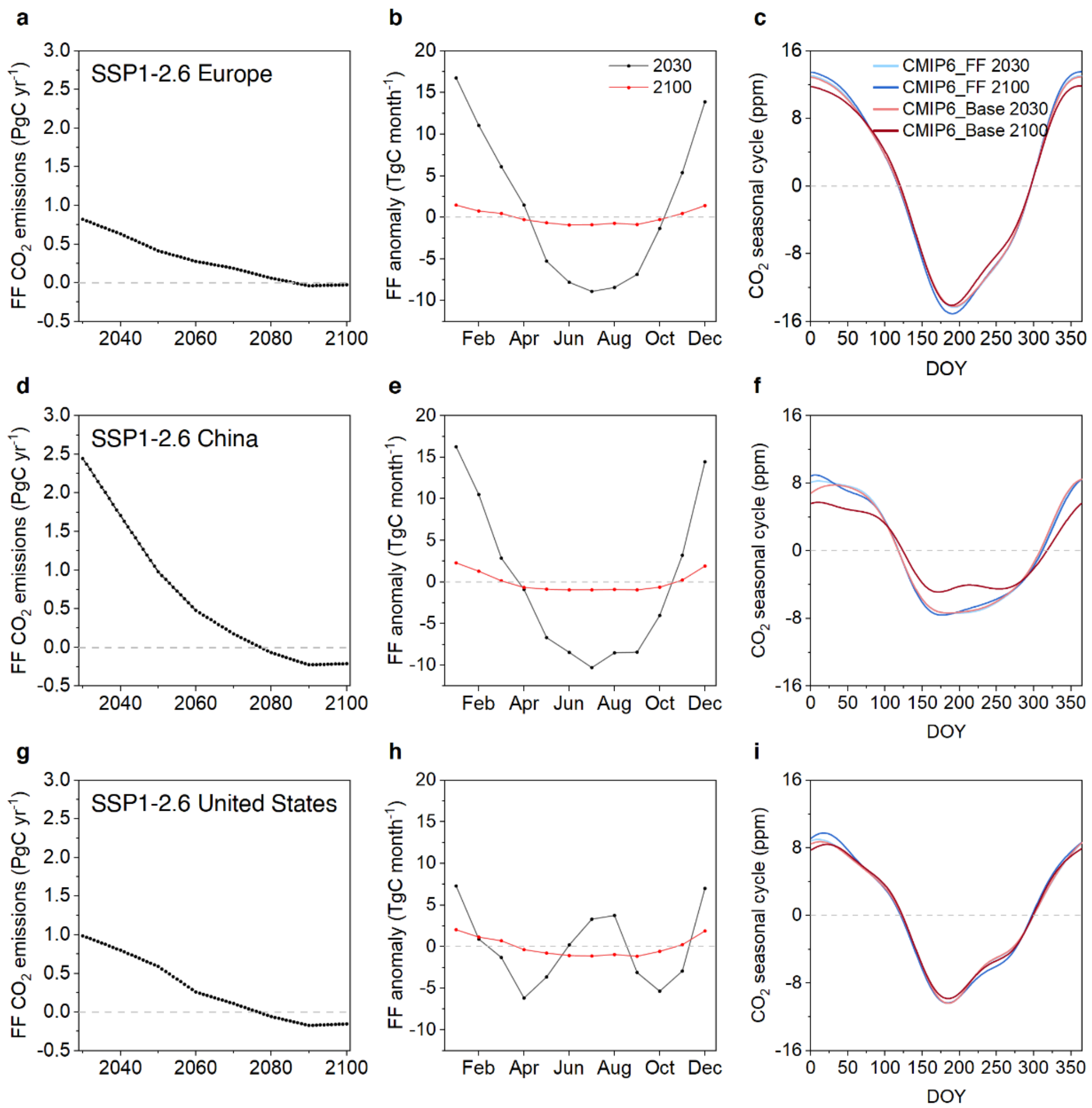

Supplementary Figure 14. Annual fossil fuel CO_2 emissions from 2030 to 2100 (**a, d, g**), monthly fossil fuel emission anomalies relative to the annual mean in 2030 and 2100 (**b, e, h**), and detrended CO_2 seasonal cycles in 2030 and 2100 from base simulations (CMIP6_Base) and factorial simulations (CMIP6_FF) (**c, f, i**) over Europe (top row), China (middle row), and the United States (bottom row) under the SSP1-2.6 scenario.

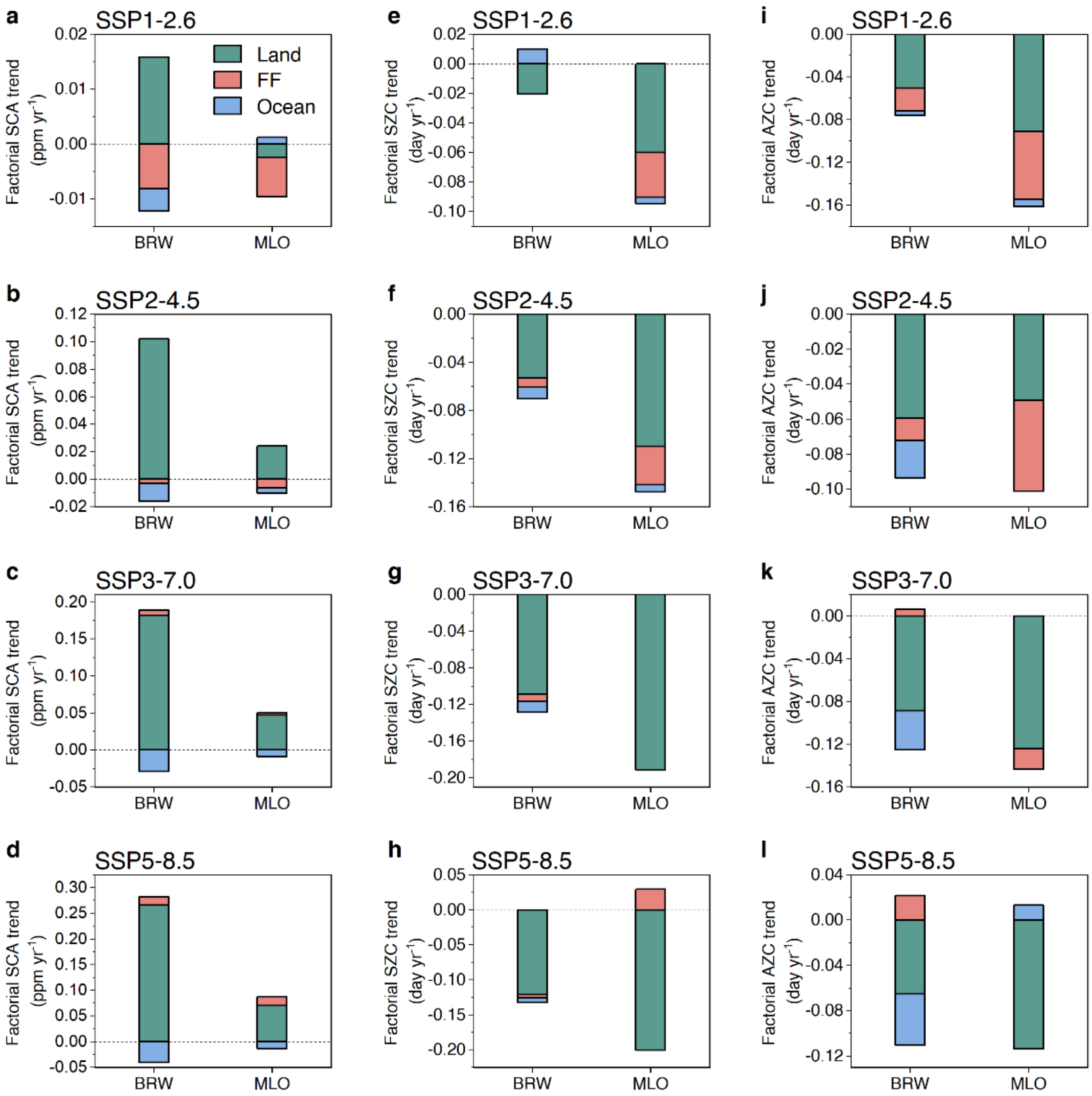

Supplementary Figure 15. Trends in SCA (**a-d**), SZC (**e-h**), and AZC (**i-l**) caused by variations in terrestrial ecosystems (Land), fossil fuel CO_2 emissions (FF), and air-sea CO_2 fluxes (Ocean), respectively, under the SSP1-2.6 (top row), SSP2-4.5 (second row), SSP3-7.0 (third row), and SSP5-8.5 (bottom row) scenarios. The factorial trends were inferred from differences between the base simulations and corresponding factorial simulations.

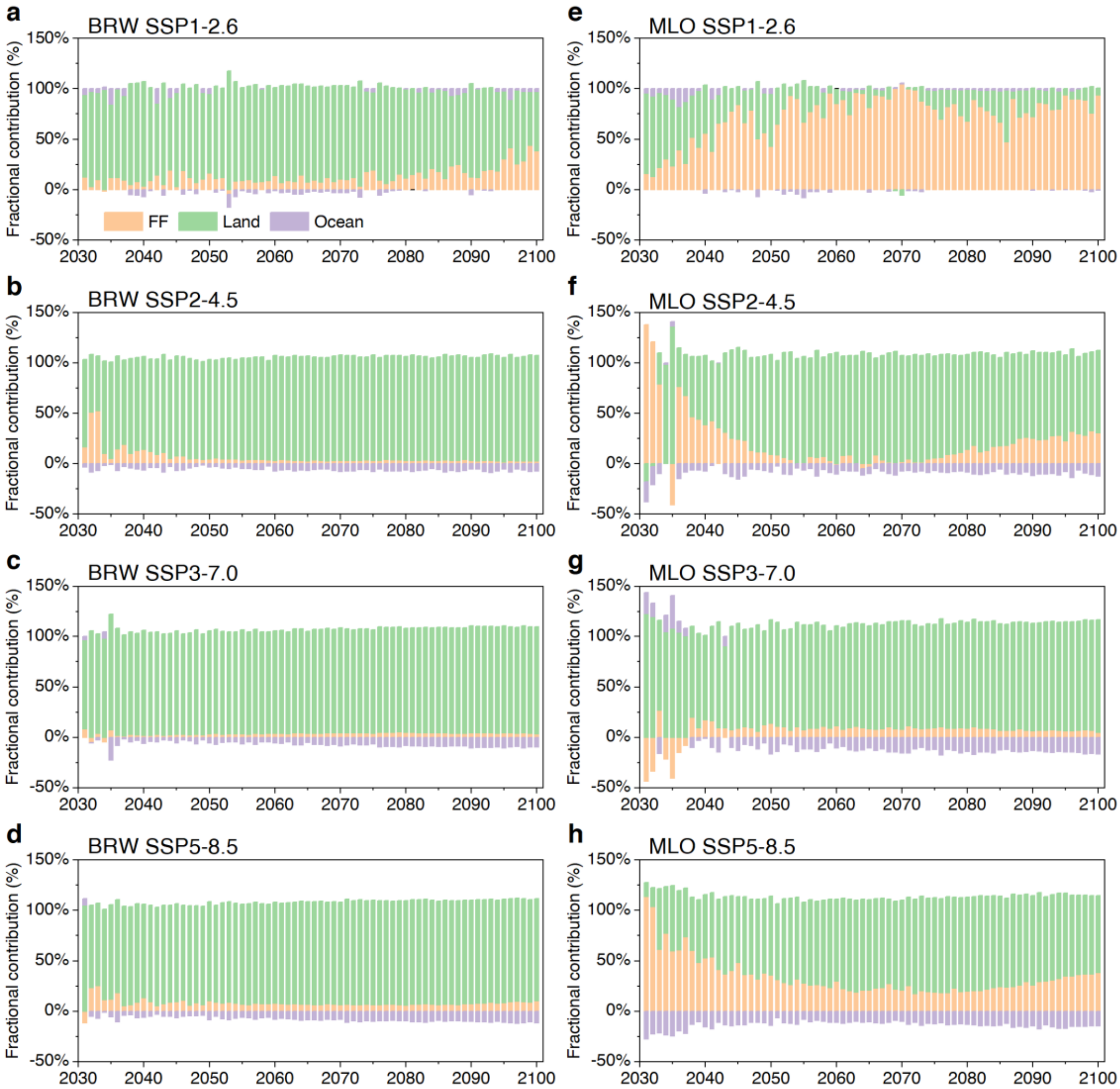

Supplementary Figure 16. Time series of the factorial contributions of fossil fuel CO_2 emissions (FF), net terrestrial CO_2 fluxes (Land), and air-sea CO_2 fluxes (Ocean) to the atmospheric CO_2 seasonal cycle, derived using the vector projection method, at the BRW (**a-d**) and MLO (**e-h**) stations under the SSP1-2.6 (top row), SSP2-4.5 (second row), SSP3-7.0 (third row), and SSP5-8.5 (bottom row) scenarios.

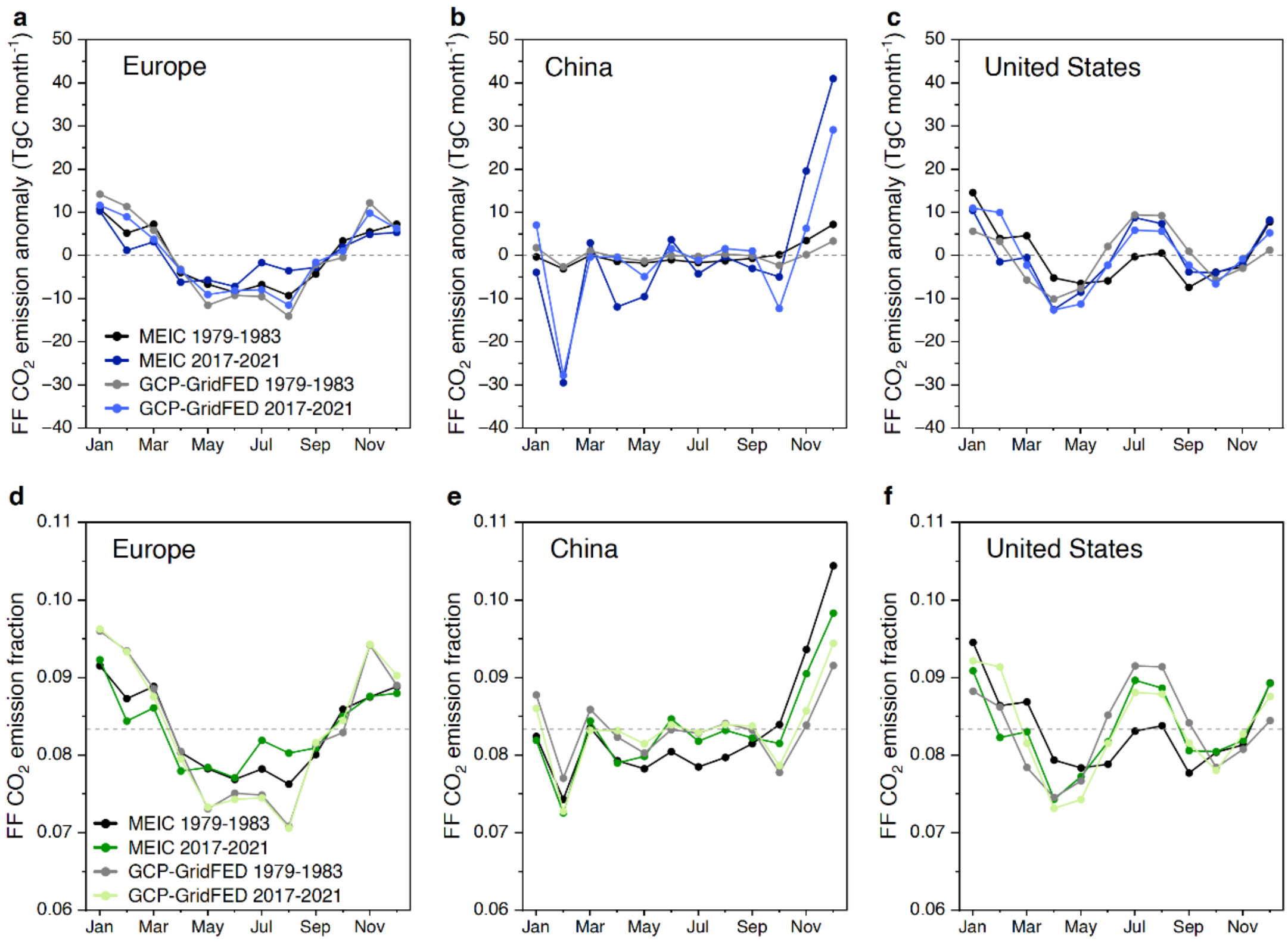

Supplementary Figure 17. Fossil fuel CO_2 emission anomalies (monthly emissions minus the annual-mean monthly emission; **a-c**) and fossil fuel CO_2 emission fractions (monthly emissions divided by the annual total emission; **d-f**) averaged over 1979-1983 and 2017-2021, estimated from the MEIC and GCP-GridFED datasets. Panels show emissions from Europe (left column), China (middle column), and the United States (right column).

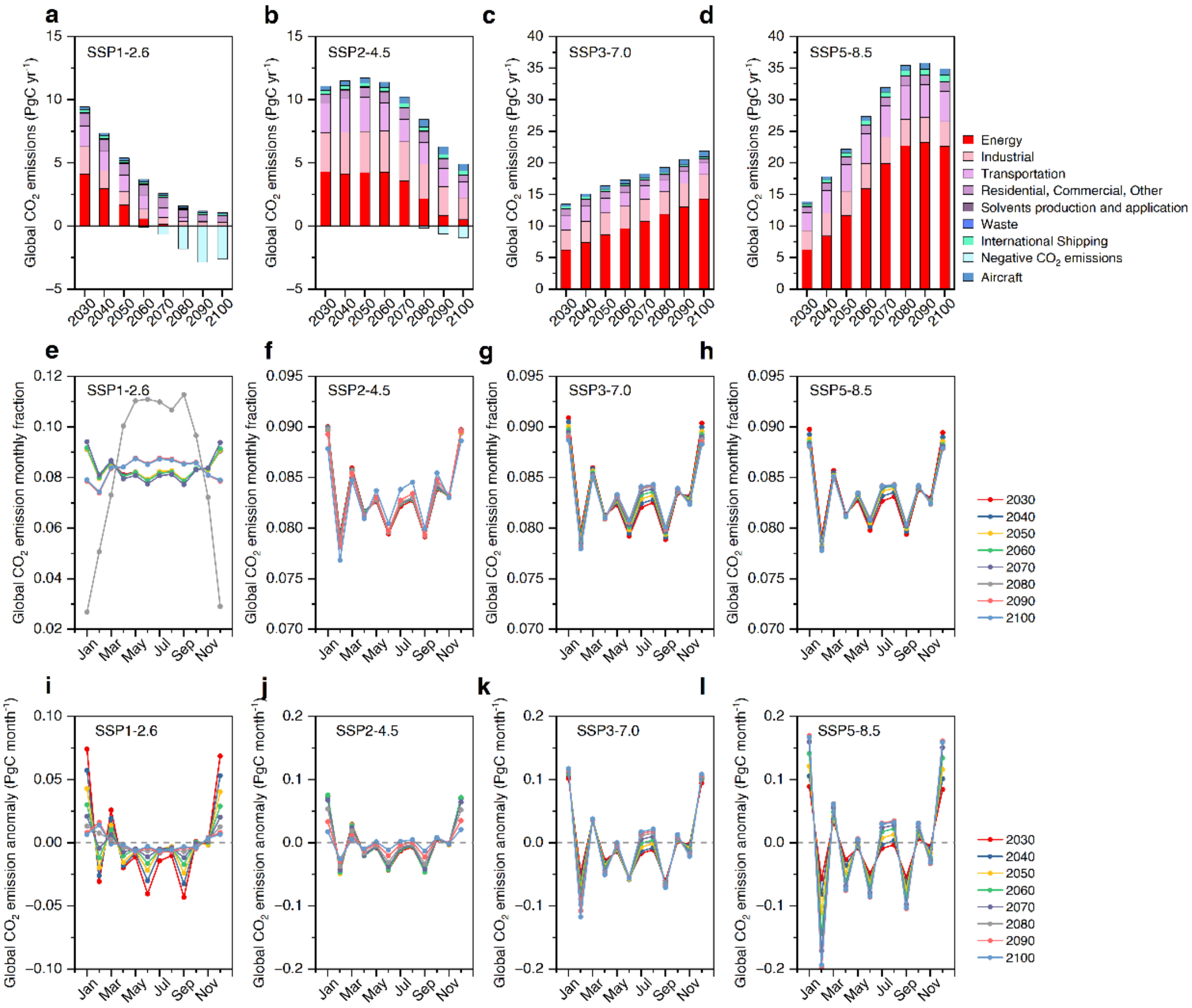

Supplementary Figure 18. Global CO_2 emissions by sector (**a-d**), monthly emission fractions (**e-h**), and monthly emission anomalies (**i-l**) under the SSP1-2.6 (first column), SSP2-4.5 (second column), SSP3-7.0 (third column) and SSP5-8.5 (fourth column) scenarios. All results are derived from the original CMIP6 projections.

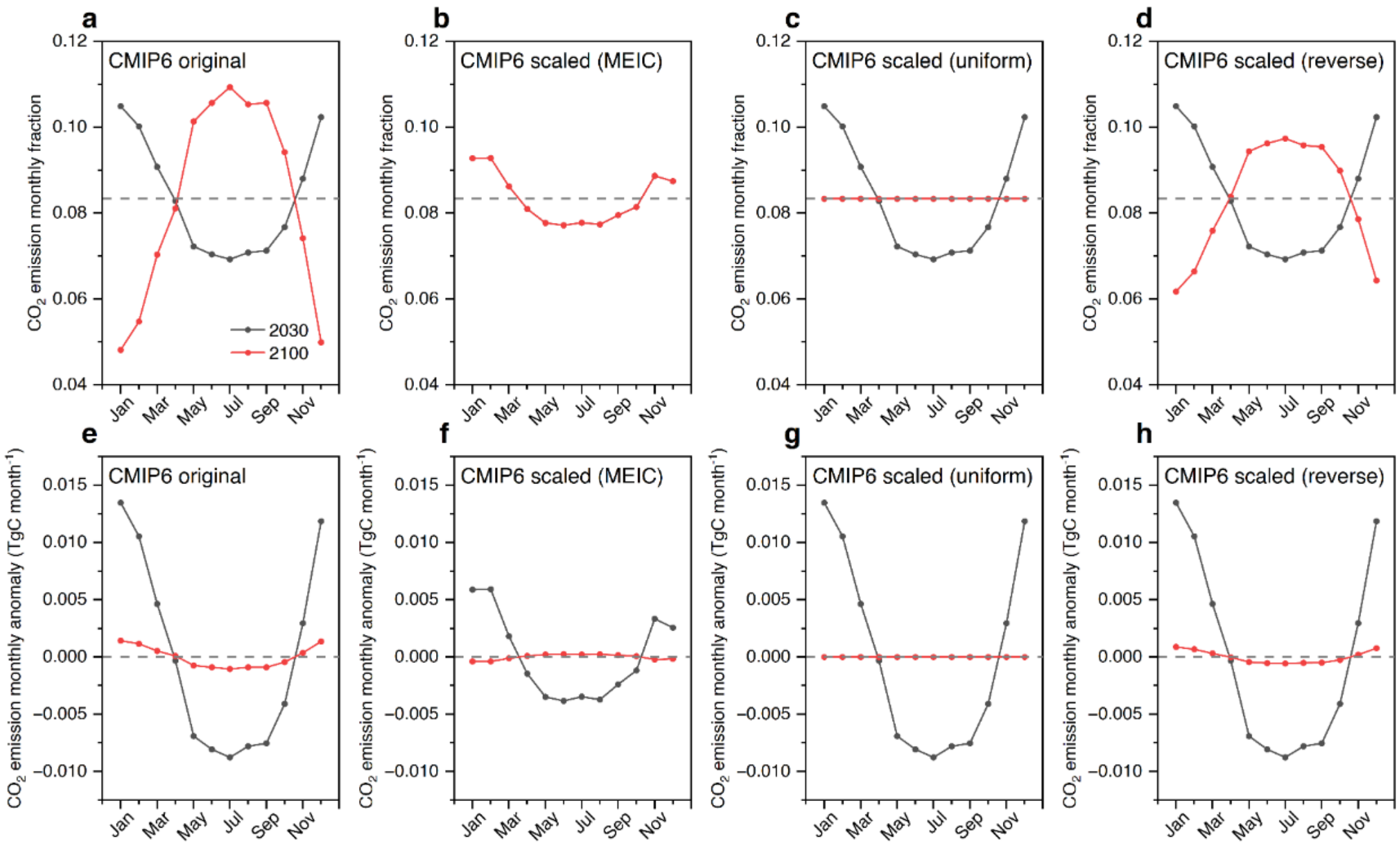

Supplementary Figure 19. Monthly CO_2 emission fractions (**a-d**) and anomalies (**e-h**) at a single grid cell (52.25°N, 8.25°E). Columns show results from the original CMIP6 projections (first column); CMIP6 projections scaled using 2017-2021 mean MEIC sector-specific seasonal fractions (second column); CMIP6 projections with seasonal fractions linearly transitioning from the 2030 distribution to a uniform distribution by 2100 (third column); and CMIP6 projections with seasonal fractions linearly transitioning from the 2030 distribution to a distribution that is mirror-symmetric about the annual mean by 2100 (fourth column).

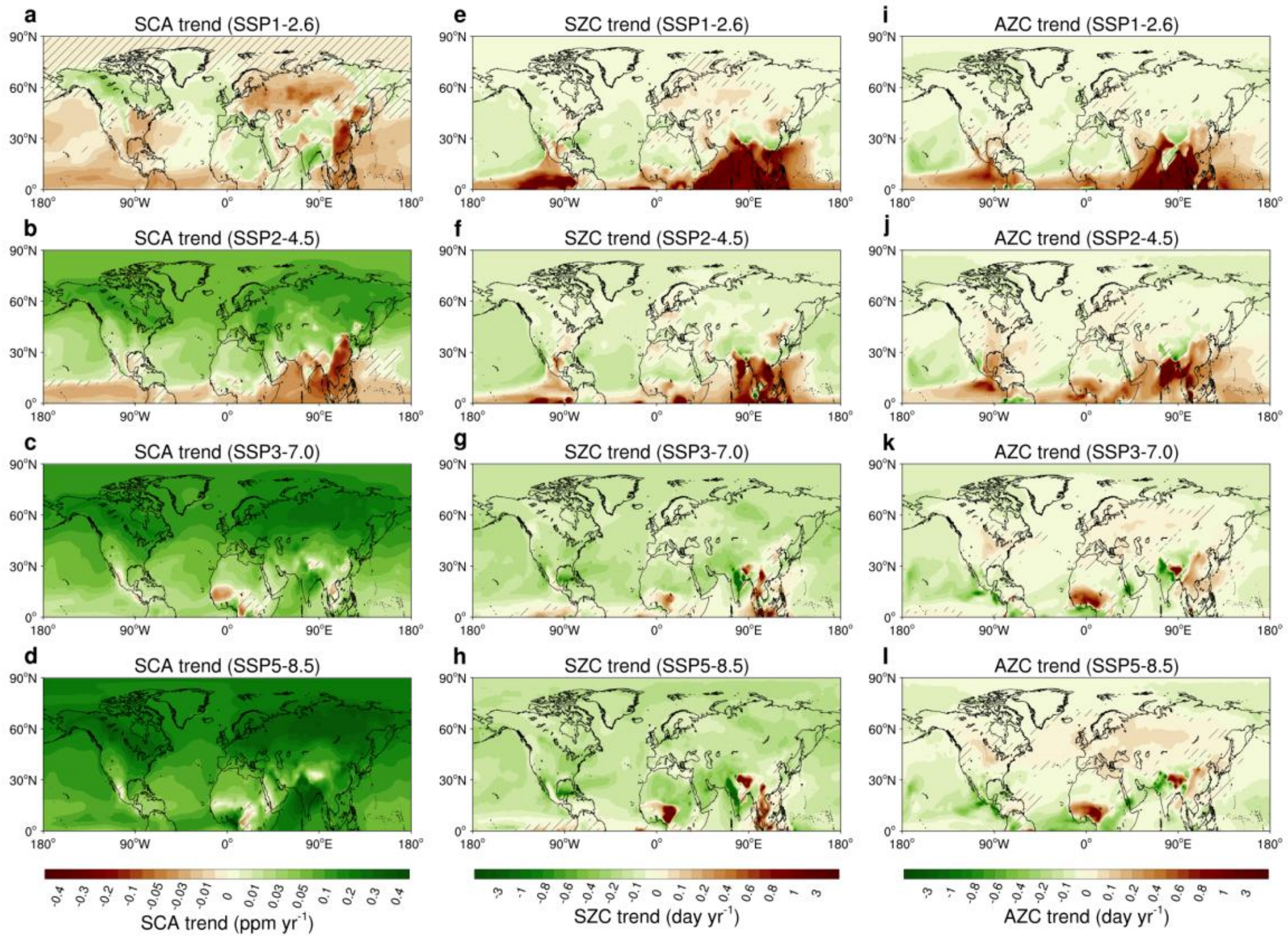

Supplementary Figure 20. Spatial patterns of simulated trends in atmospheric CO_2 seasonal parameters across the Northern Hemisphere under four future scenarios. Fossil fuel CO_2 emissions in each scenario are derived by scaling CMIP6 sectoral monthly emissions using sector-specific monthly fractional contributions averaged over 2017-2021 from the MEIC inventory. Simulated trends in seasonal cycle amplitude (SCA; **a-d**), spring zero-crossing date (SZC; **e-h**), and autumn zero-crossing date (AZC; **i-l**) for the SSP1-2.6 (top row), SSP2-4.5 (second row), SSP3-7.0 (third row), and SSP5-8.5 scenarios (bottom row). Hatched areas denote regions where trends are not statistically significant ($p > 0.05$).

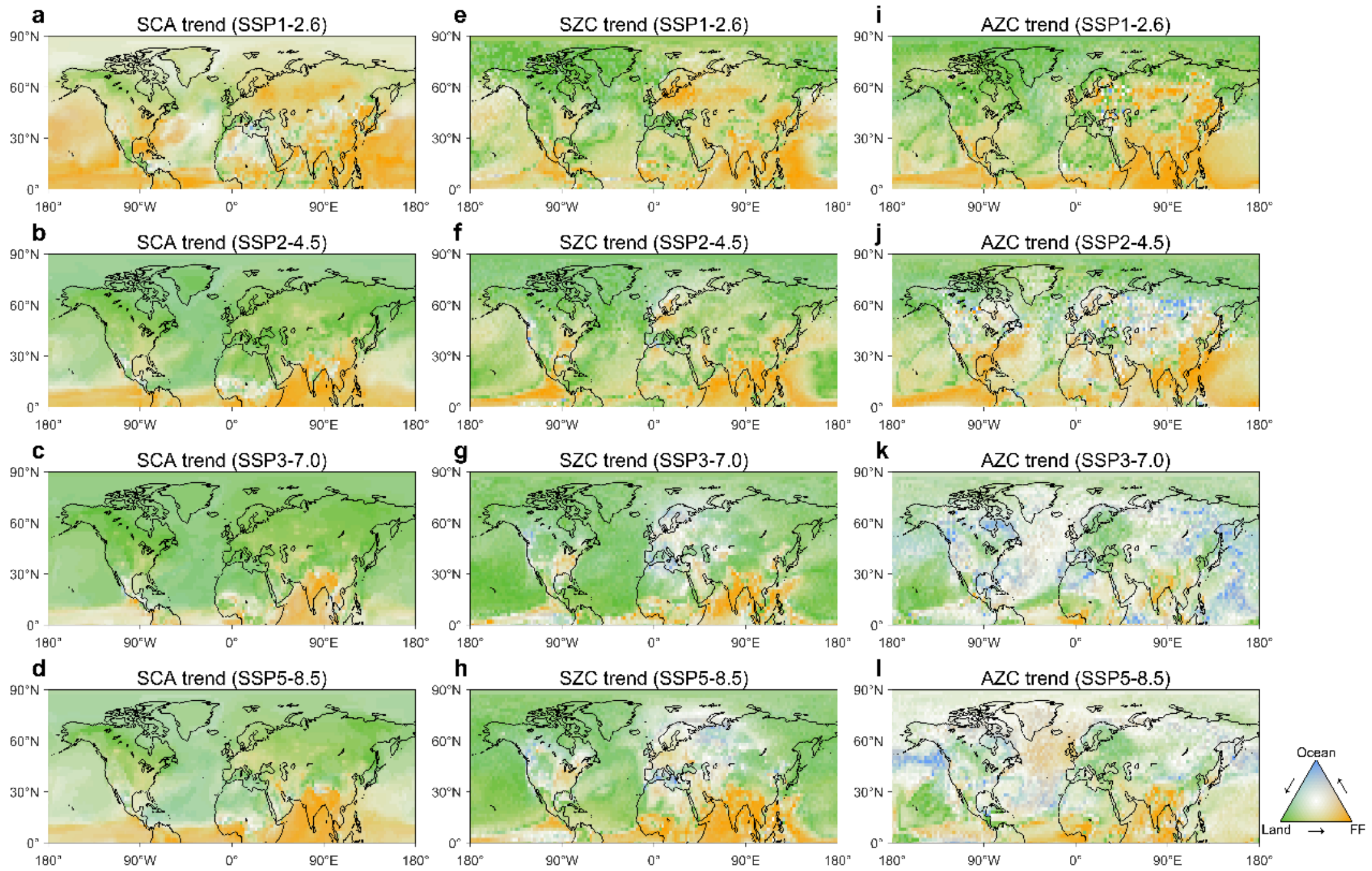

Supplementary Figure 21. Relative proportions of net terrestrial CO_2 fluxes, fossil fuel CO_2 emissions, and air-sea CO_2 exchange contributing to trends in atmospheric CO_2 seasonal parameters across the Northern Hemisphere from 2030 to 2100 are shown under different future scenarios. Fossil fuel CO_2 emissions in each scenario are derived by scaling CMIP6 sectoral monthly emissions using sector-specific monthly fractional contributions averaged over 2017-2021 from the MEIC inventory. Panels display the relative proportions of the three component fluxes associated with trends in SCA (**a-d**), SZC (**e-h**), and AZC (**i-l**) for the SSP1-2.6 (top row), SSP2-4.5 (second row), SSP3-7.0 (third row), and SSP5-8.5 (bottom row) scenarios.

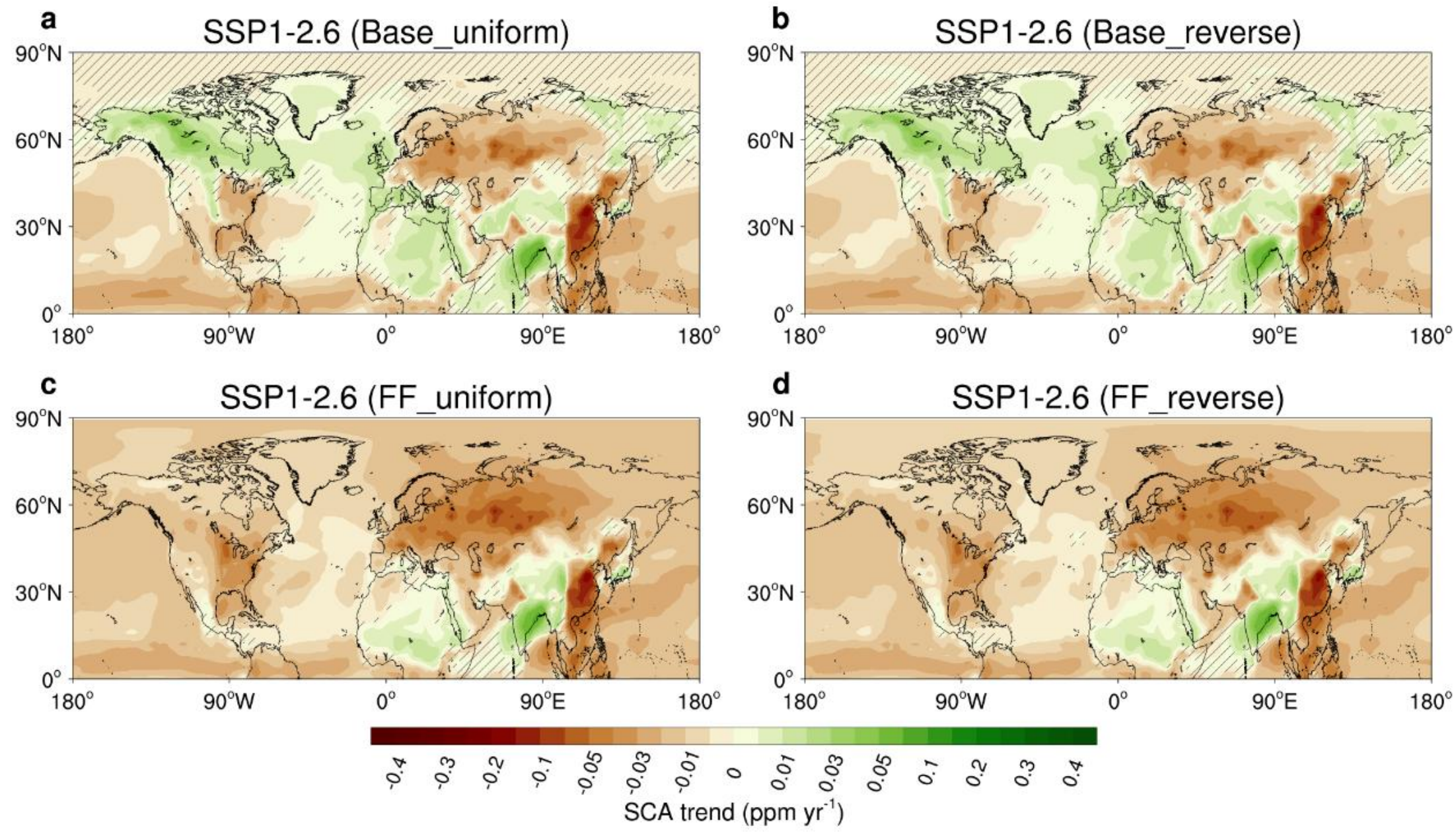

Supplementary Figure 22. Spatial patterns of SCA trends across the Northern Hemisphere during 2030-2100, derived from CMIP6_Base simulations (**a, b**), and the SCA trends attributable to fossil fuel CO_2 emissions (FF), estimated as the difference between CMIP6_Base and CMIP6_FF (**c, d**). In the first column, simulations are based on a rescaled seasonal distribution of CMIP6 FF emissions, in which the monthly fractions are progressively adjusted from 2030 onwards to converge to a uniform distribution by 2100. In the second column, simulations are based on an alternative rescaling of the seasonal emission distribution, where the monthly fractions are gradually modified from 2030 to achieve, by 2100, a reversed (mirror-symmetric with respect to the annual mean) configuration of the 2030 distribution. Hatched areas denote regions where trends are not statistically significant ($p > 0.05$).

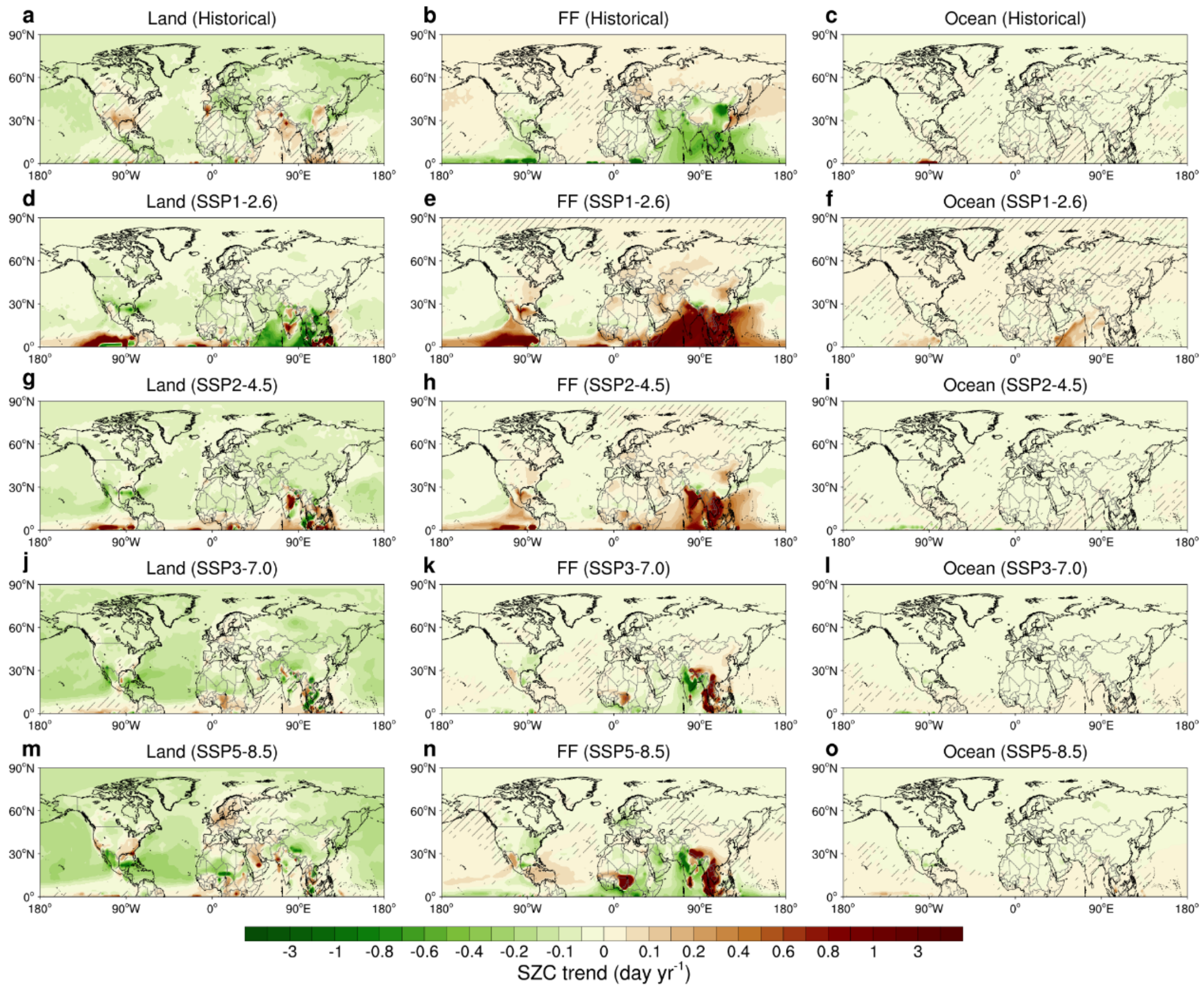

Supplementary Figure 23. SZC trends attributed to variations in the terrestrial ecosystem (left panels), fossil fuel CO_2 emissions (middle panels), and air-sea CO_2 exchange (right panels) during the historical period (**a-c**) and under the SSP1-2.6 (**d-f**), SSP2-4.5 (**g-i**), SSP3-7.0 (**j-l**), and SSP5-8.5 (**m-o**) scenarios, estimated from the differences between base simulations and the corresponding factorial simulations. The hatched areas indicate that the trends in these regions did not pass the significance test ($p > 0.05$).

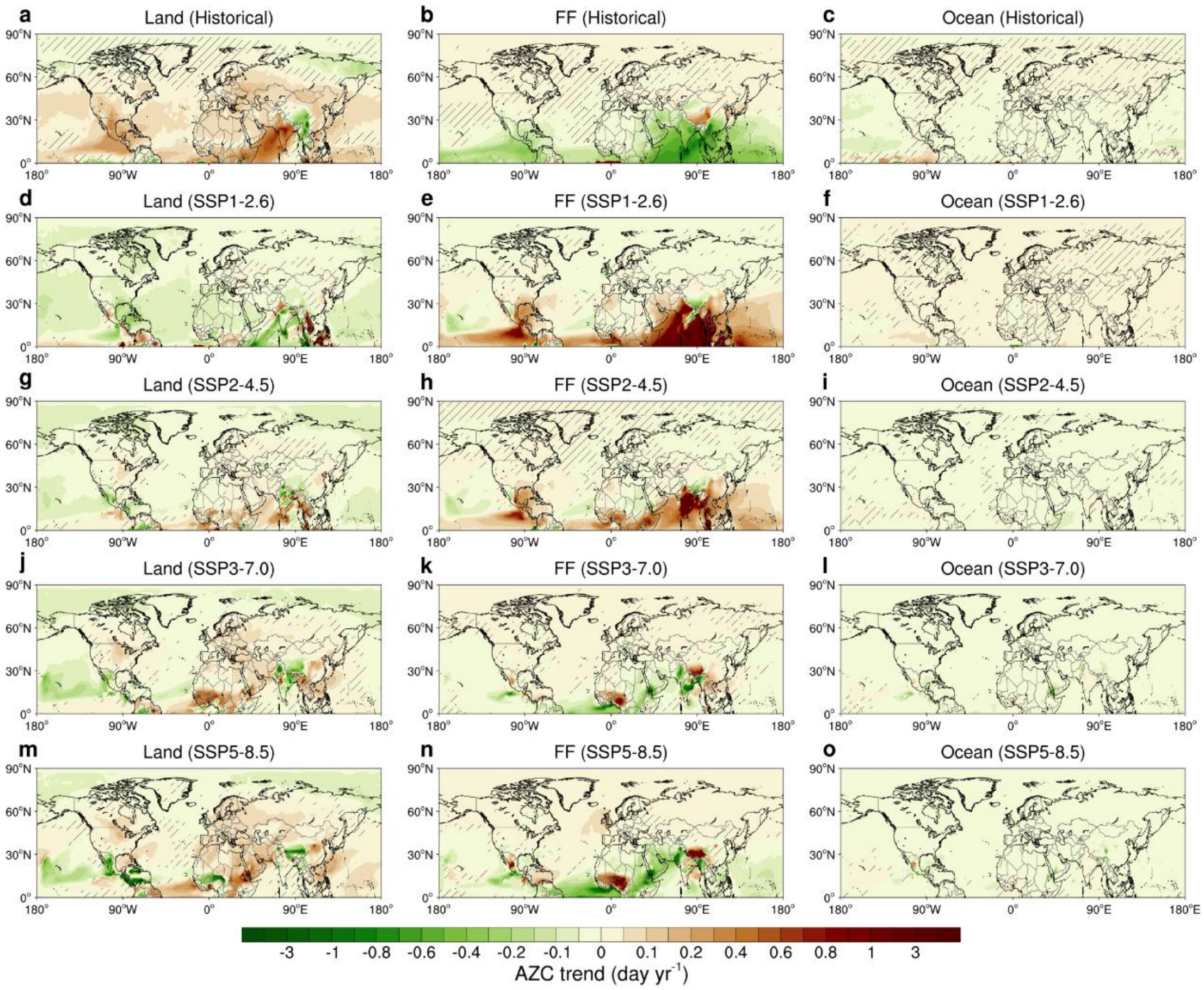

Supplementary Figure 24. AZC trends attributed to variations in the terrestrial ecosystem (left panels), fossil fuel CO_2 emissions (middle panels), and air-sea CO_2 exchange (right panels) during the historical period (**a-c**) and under the SSP1-2.6 (**d-f**), SSP2-4.5 (**g-i**), SSP3-7.0 (**j-l**), and SSP5-8.5 (**m-o**) scenarios, estimated from the differences between base simulations and the corresponding factorial simulations. The hatched areas indicate that the trends in these regions did not pass the significance test ($p > 0.05$).

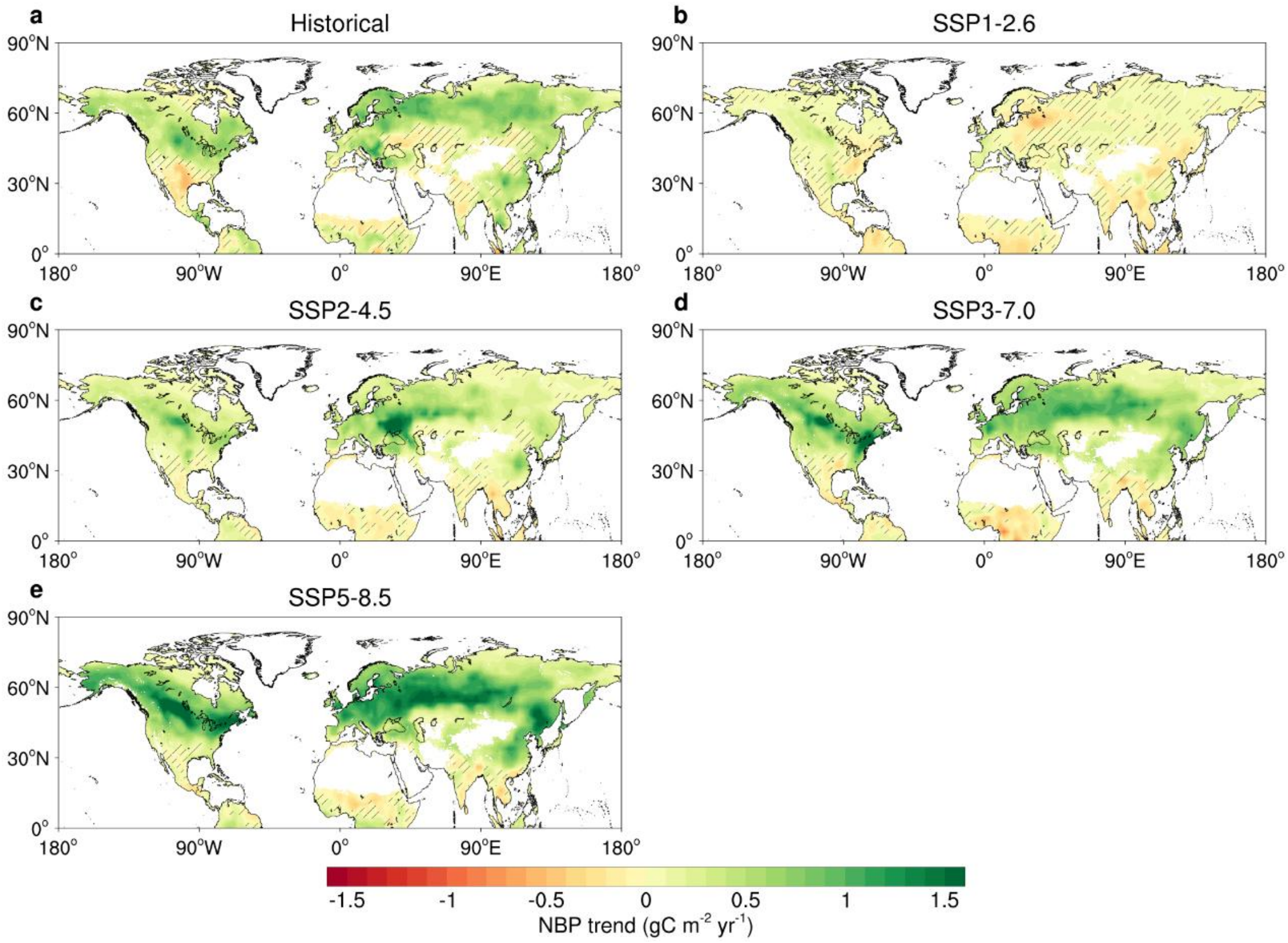

Supplementary Figure 25. Trends in integrated NBP from May to June in the Northern Hemisphere under historical (**a**), SSP1-2.6 (**b**), SSP2-4.5 (**c**), SSP3-7.0 (**d**), and SSP5-8.5 (**e**) scenarios. The hatched areas indicate that the trends in these regions did not pass the significance test ($p > 0.05$).

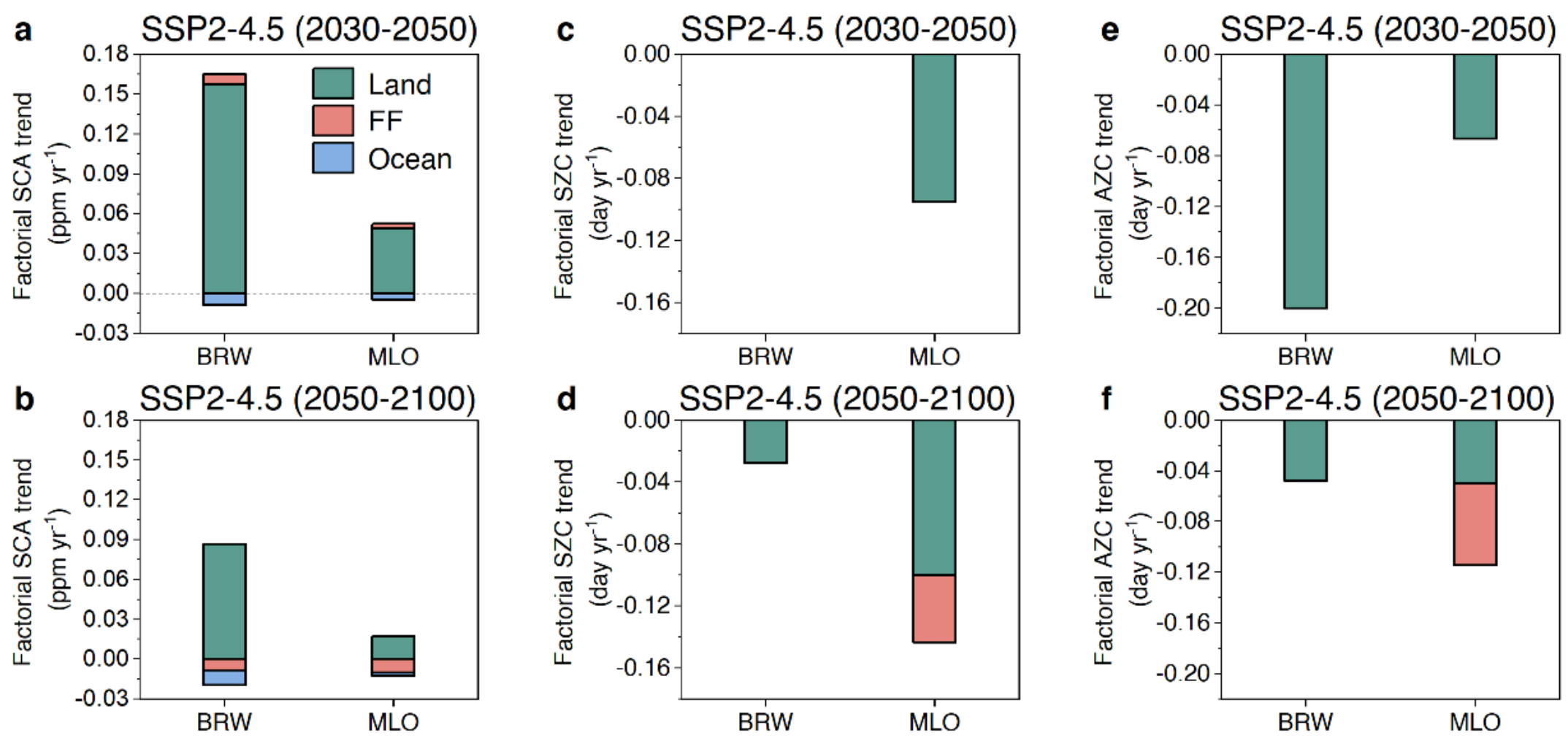

Supplementary Figure 26. Trends in SCA (**a, b**), SZC (**c, d**), and AZC (**e, f**) caused by variations in terrestrial ecosystems (Land), fossil fuel CO_2 emissions (FF), and air-sea CO_2 fluxes (Ocean) for 2030–2050 (top row) and 2050–2100 (bottom row) under the SSP2-4.5 scenario. The factorial trends were inferred from differences between the base simulations and corresponding factorial simulations.

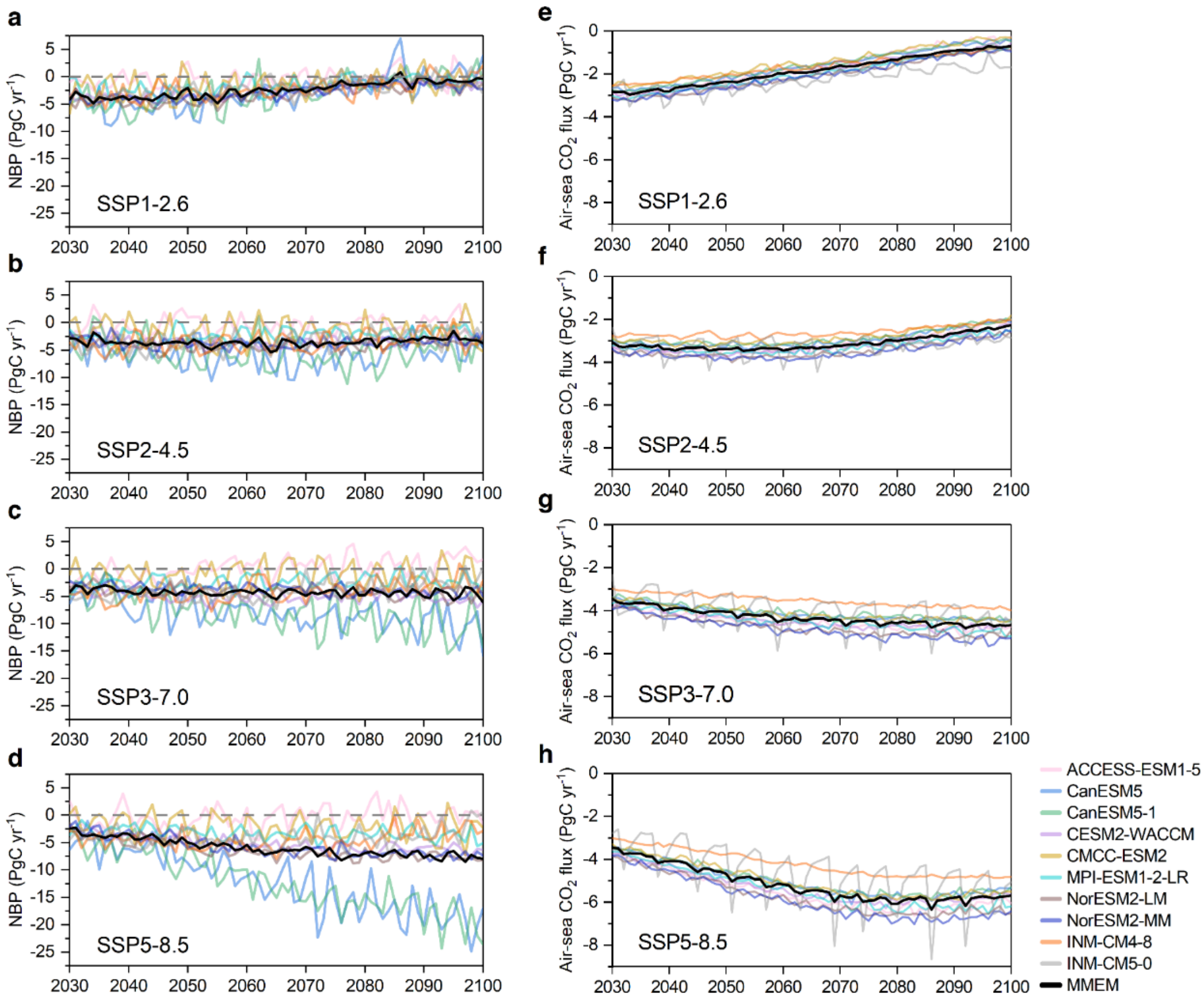

Supplementary Figure 27. Projections of NBE (**a-d**) and air-sea CO_2 fluxes (**e-h**) (2030–2100) from ten Earth system models (ESMs) and their multi-model ensemble mean (MMEM) under the SSP1-2.6 (top row), SSP2-4.5 (second row), SSP3-7.0 (third row), and SSP5-8.5 (bottom row) scenarios. Negative values indicate carbon sinks, while positive values indicate carbon sources.

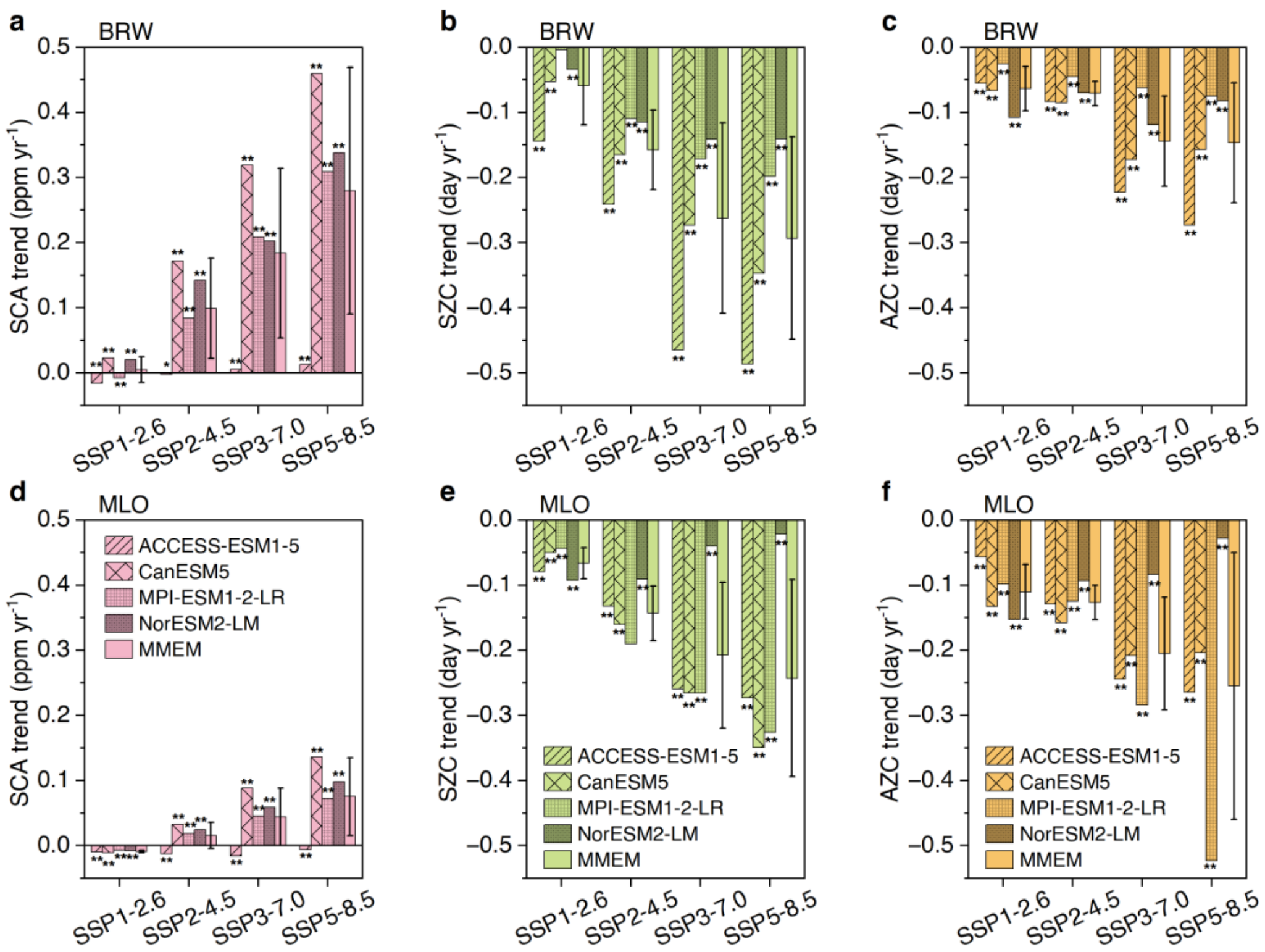

Supplementary Figure 28. Variations in the seasonality of atmospheric CO_2 under four future scenarios at the Barrow, Alaska (BRW; **a-c**), and Mauna Loa, Hawaii (MLO; **d-f**) stations. The trends were simulated using terrestrial and ocean CO_2 fluxes from four ESMs, including ACCESS-ESM1-5, CanESM5, MPI-ESM1-2-LR, and NorESM2-LM. MMEM denotes the multi-model ensemble mean across these models, and the error bars represent the inter-model standard deviation. The panels display the trends in SCA (left column), SZC (middle column), and AZC (right column). Statistically significant trends ($p < 0.05$) are marked by two dots above the bars, and marginally significant trends ($p < 0.1$) by one dot.

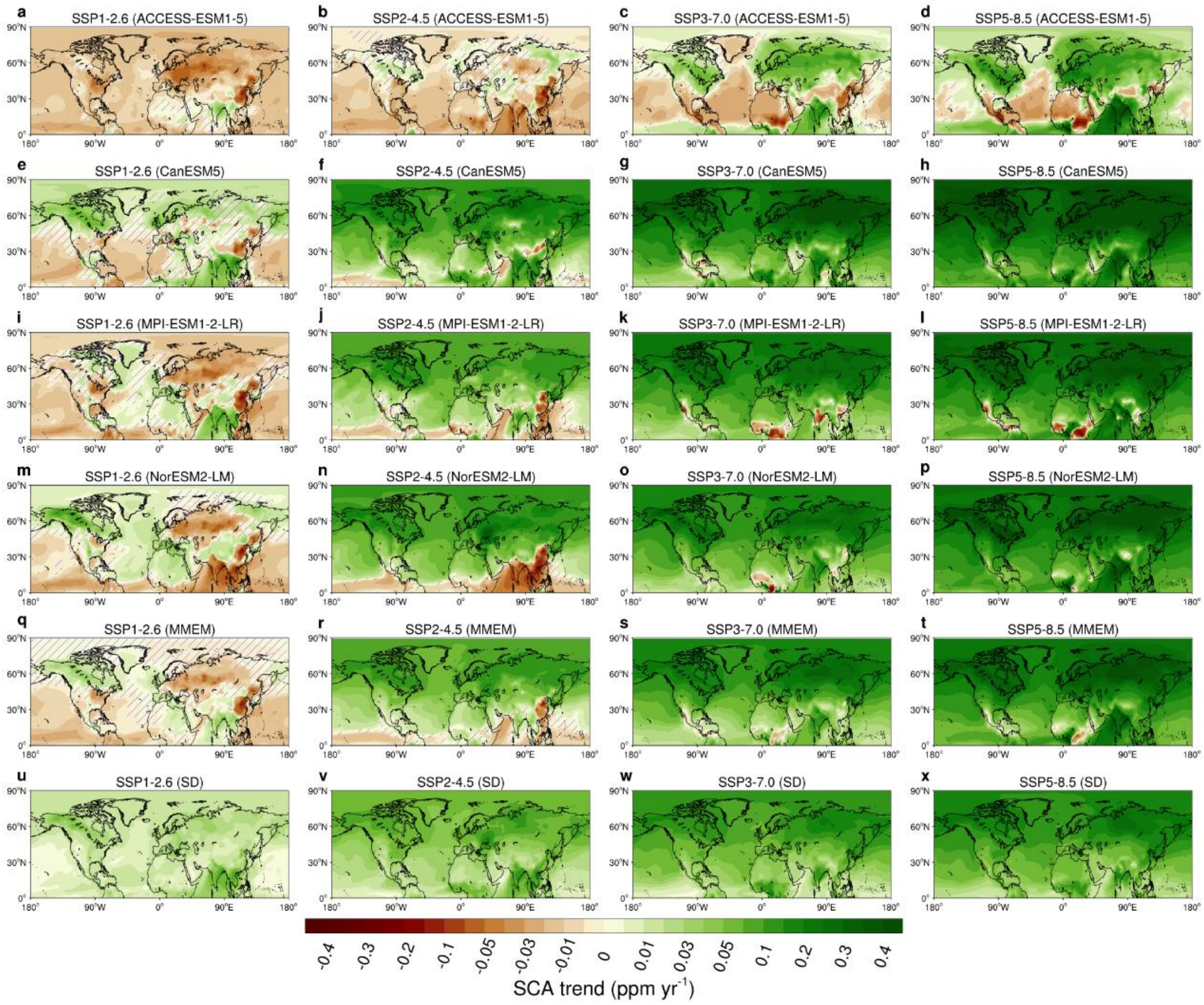

Supplementary Figure 29. Spatial patterns of simulated SCA trends across the Northern Hemisphere from 2030 to 2100, driven by different terrestrial and ocean CO_2 fluxes, under four future scenarios. Panels show SCA trends driven by terrestrial and ocean CO_2 fluxes from ACCESS-ESM1-5 (**a-d**), CanESM5 (**e-h**), MPI-ESM1-2-LR (**i-l**), and NorESM2-LM (**m-p**), along with the multi-model ensemble mean of the four estimates (**q-t**) and the standard deviation of these estimates (**u-x**). Results are shown for SSP1-2.6 (first column), SSP2-4.5 (second column), SSP3-7.0 (third column), and SSP5-8.5 (fourth column). Hatched areas denote regions where trends are not statistically significant ($p > 0.05$).

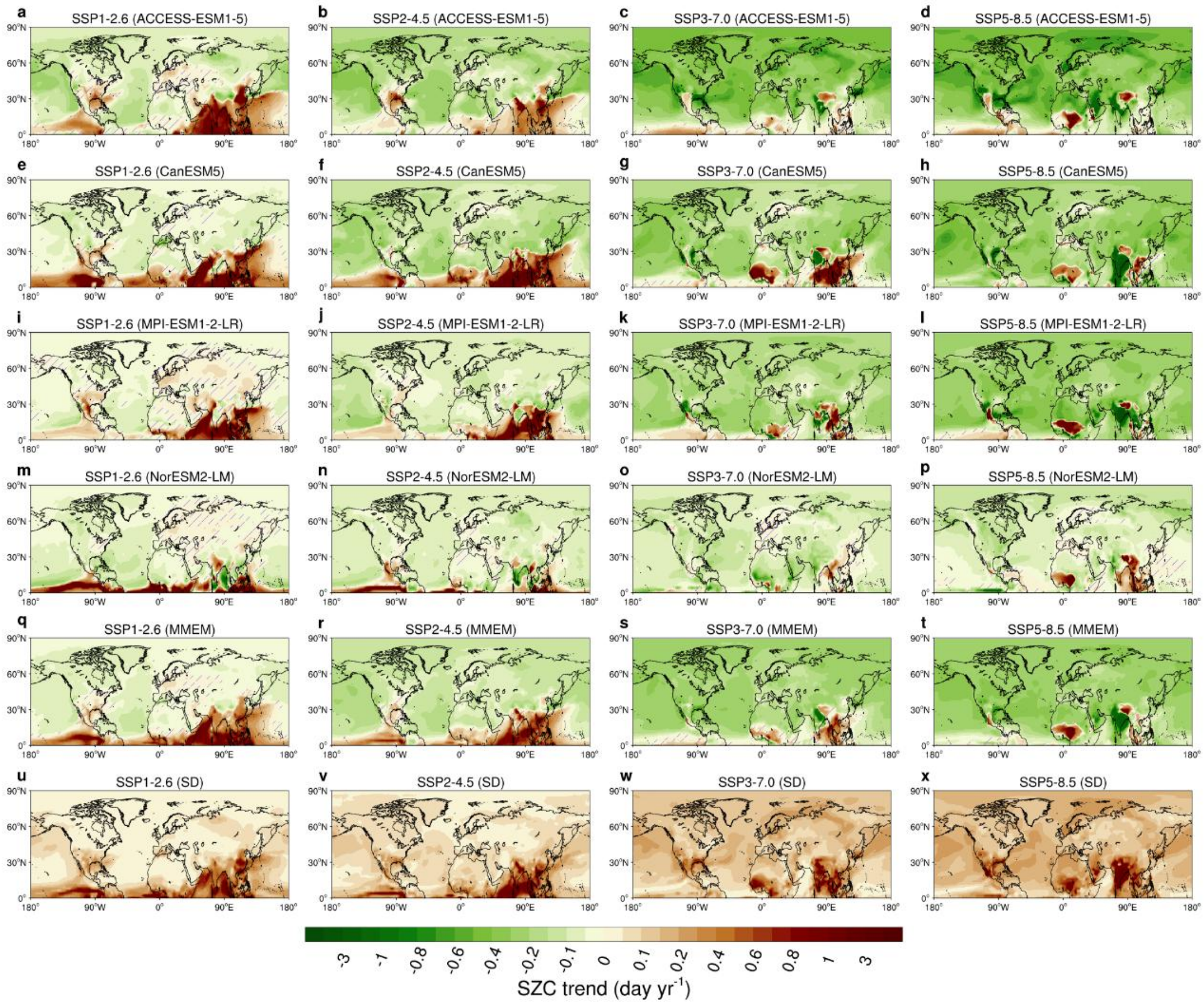

Supplementary Figure 30. Same as Supplementary Fig. 26, but showing the SZC trend.

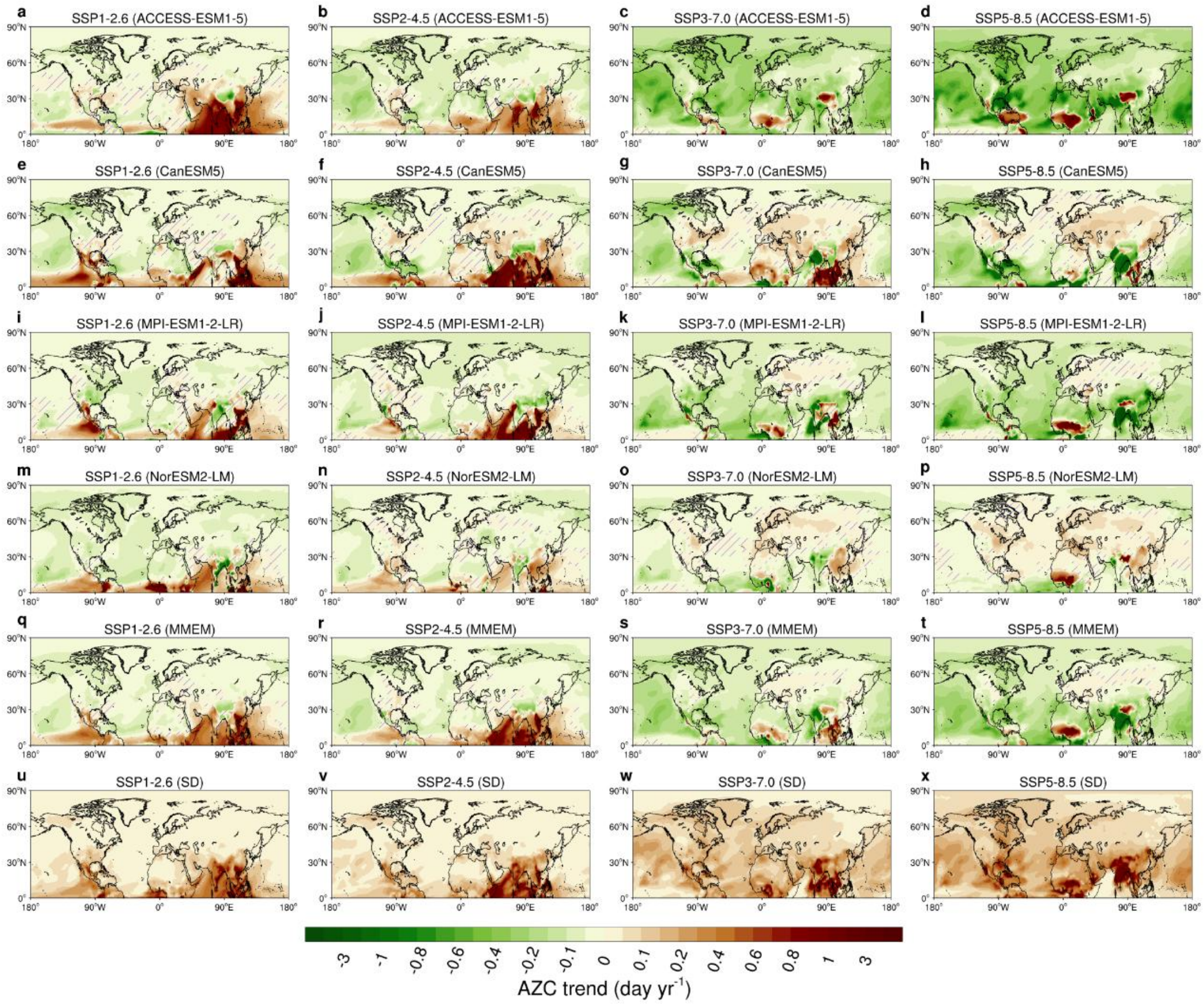

Supplementary Figure 31. Same as Supplementary Fig. 26, but showing the AZC trend.

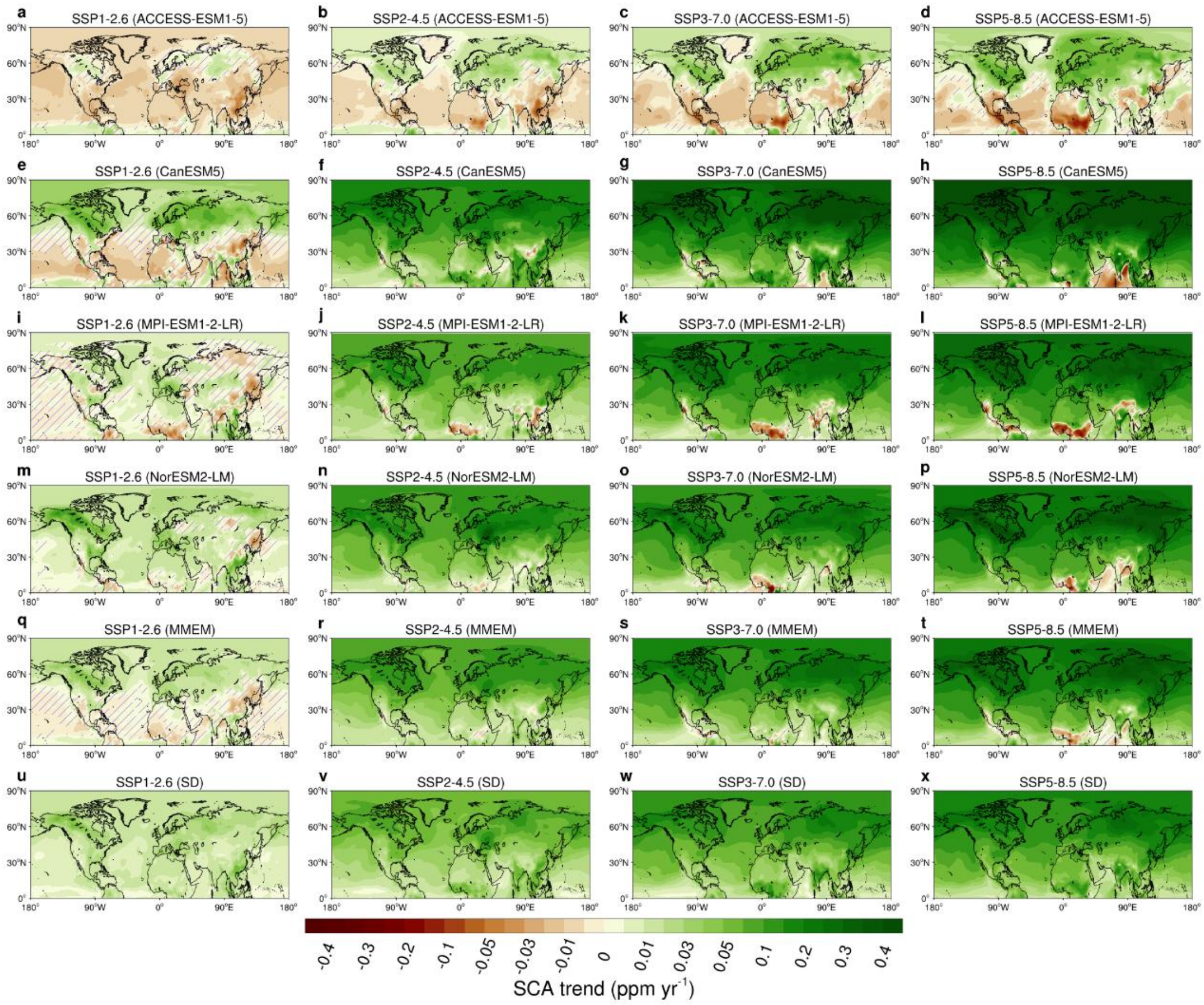

Supplementary Figure 32. Spatial patterns of SCA trends caused by terrestrial ecosystem across the Northern Hemisphere from 2030 to 2100 under four future scenarios, estimated from the differences between CMIP6_Base and CMIP6_Land. Panels show SCA trends caused by terrestrial CO_2 fluxes from ACCESS-ESM1-5 (**a-d**), CanESM5 (**e-h**), MPI-ESM1-2-LR (**i-l**), and NorESM2-LM (**m-p**), along with the multi-model ensemble mean of the four estimates (**q-t**) and the standard deviation of these estimates (**u-x**). Results are shown for SSP1-2.6 (first column), SSP2-4.5 (second column), SSP3-7.0 (third column), and SSP5-8.5 (fourth column). Hatched areas denote regions where trends are not statistically significant ($p > 0.05$).

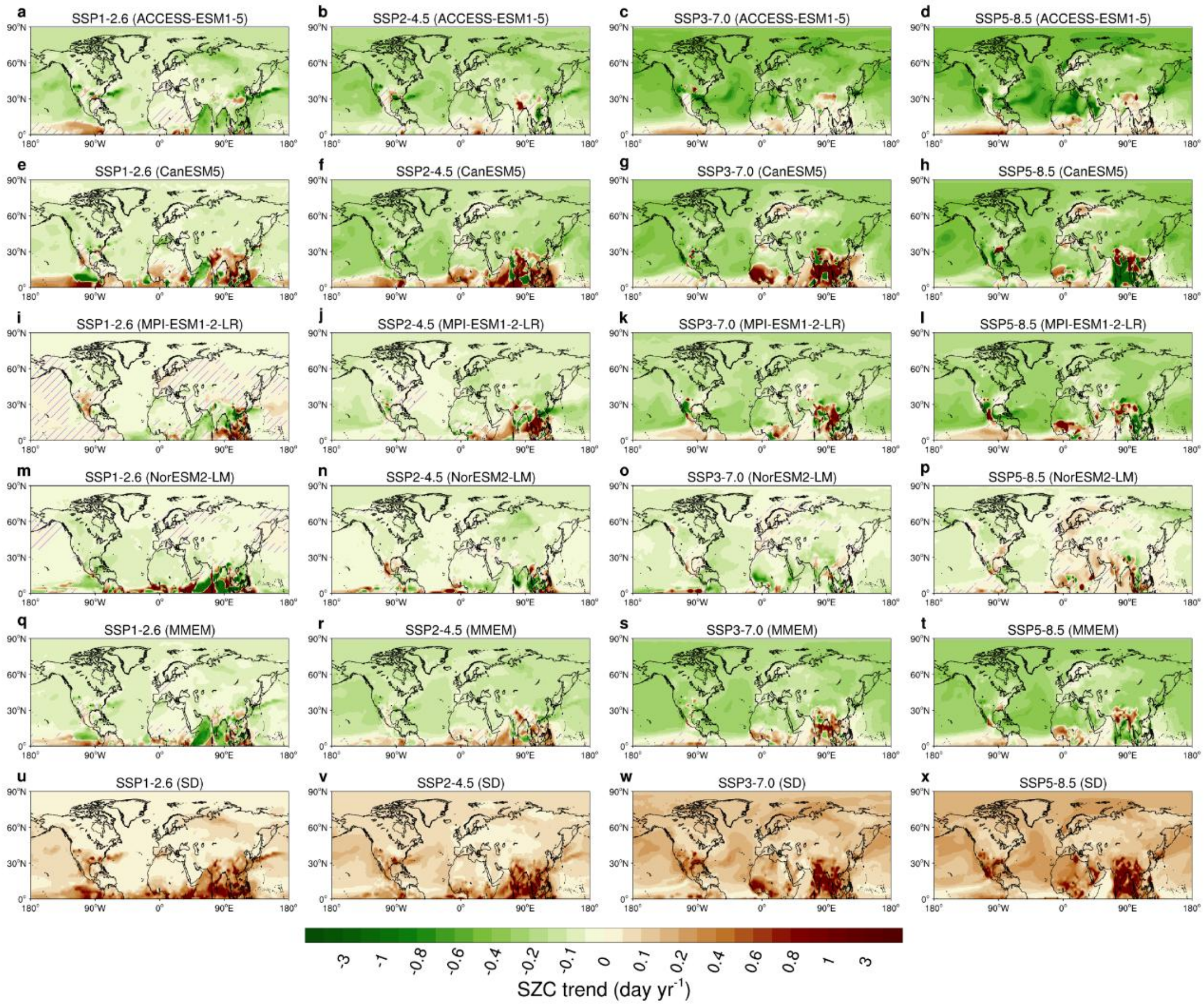

Supplementary Figure 33. Same as Supplementary Fig. 29, but showing the SZC trends caused by terrestrial ecosystems.

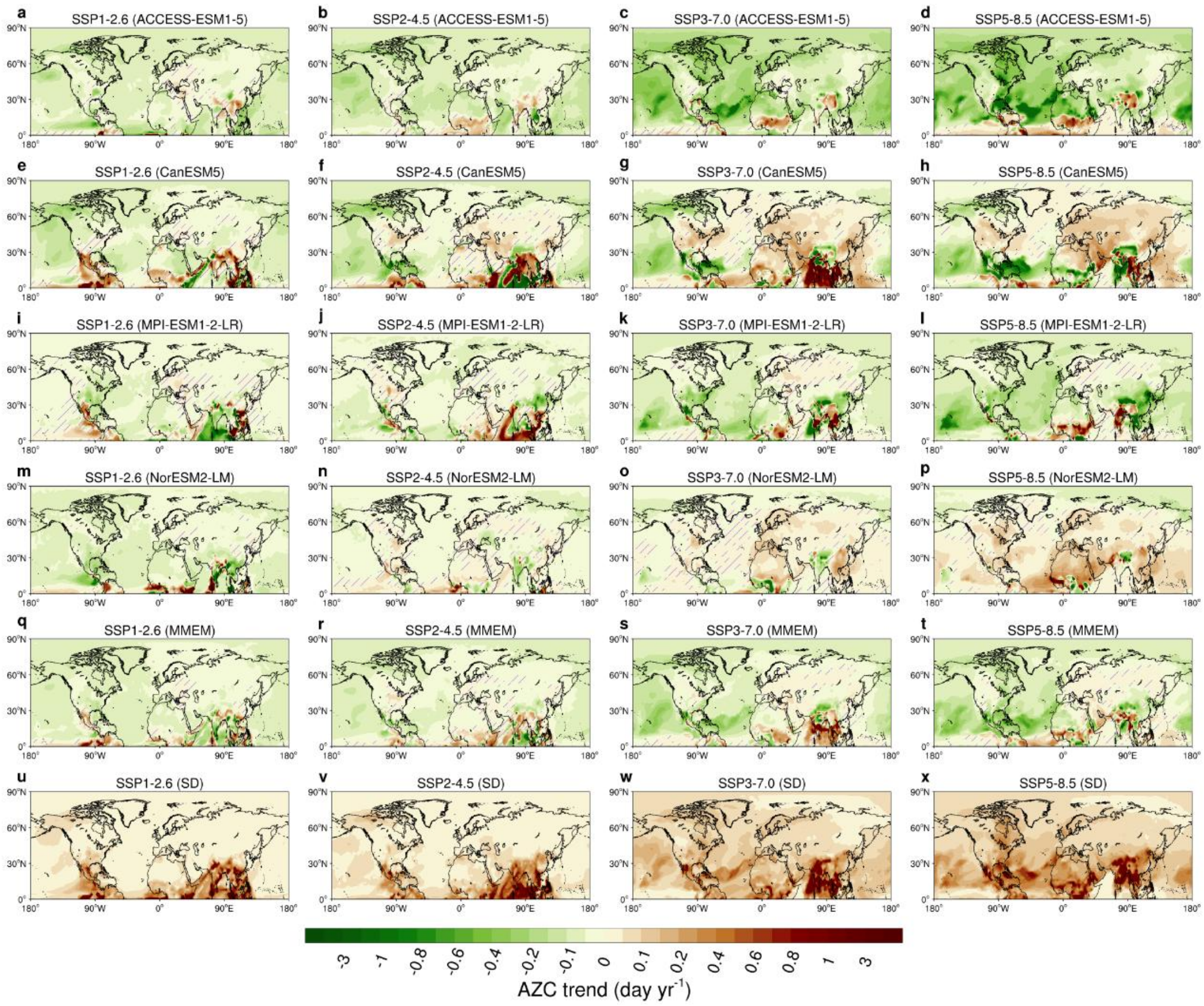

Supplementary Figure 34. Same as Supplementary Fig. 29, but showing the AZC trends caused by terrestrial ecosystems.

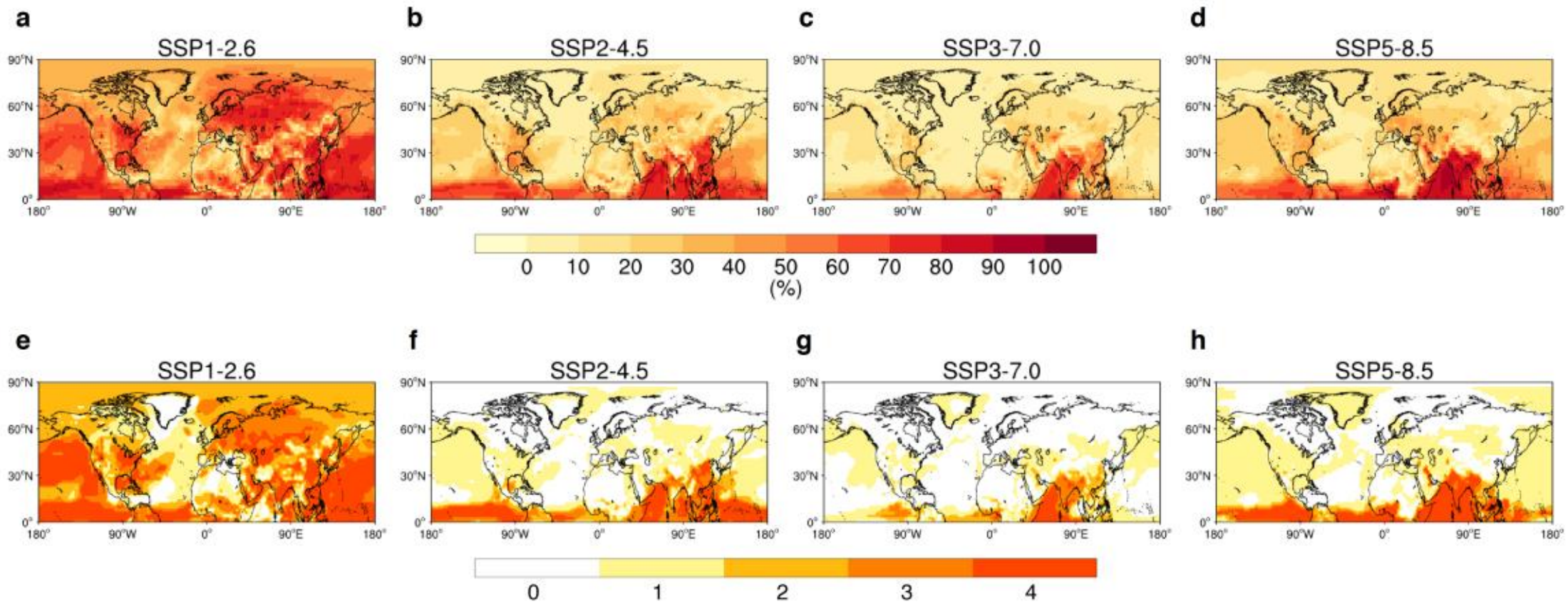

Supplementary Figure 35. Based on future transport simulations driven by carbon fluxes from four Earth system models (ACCESS-ESM1-5, CanESM5, NorESM2-LM, and MPI-ESM1-2-LR), panels (**a-d**) show the relative contribution of fossil-fuel driven SCA trends to the overall SCA trends by all components. Panels (**e-h**) show, at each grid cell in the Northern Hemisphere, the number of models in which the contribution of fossil fuel-driven SCA trends exceeds that driven by terrestrial ecosystem. Columns from left to right correspond to the SSP1-2.6, SSP2-4.5, SSP3-7.0, and SSP5-8.5 scenarios, respectively.

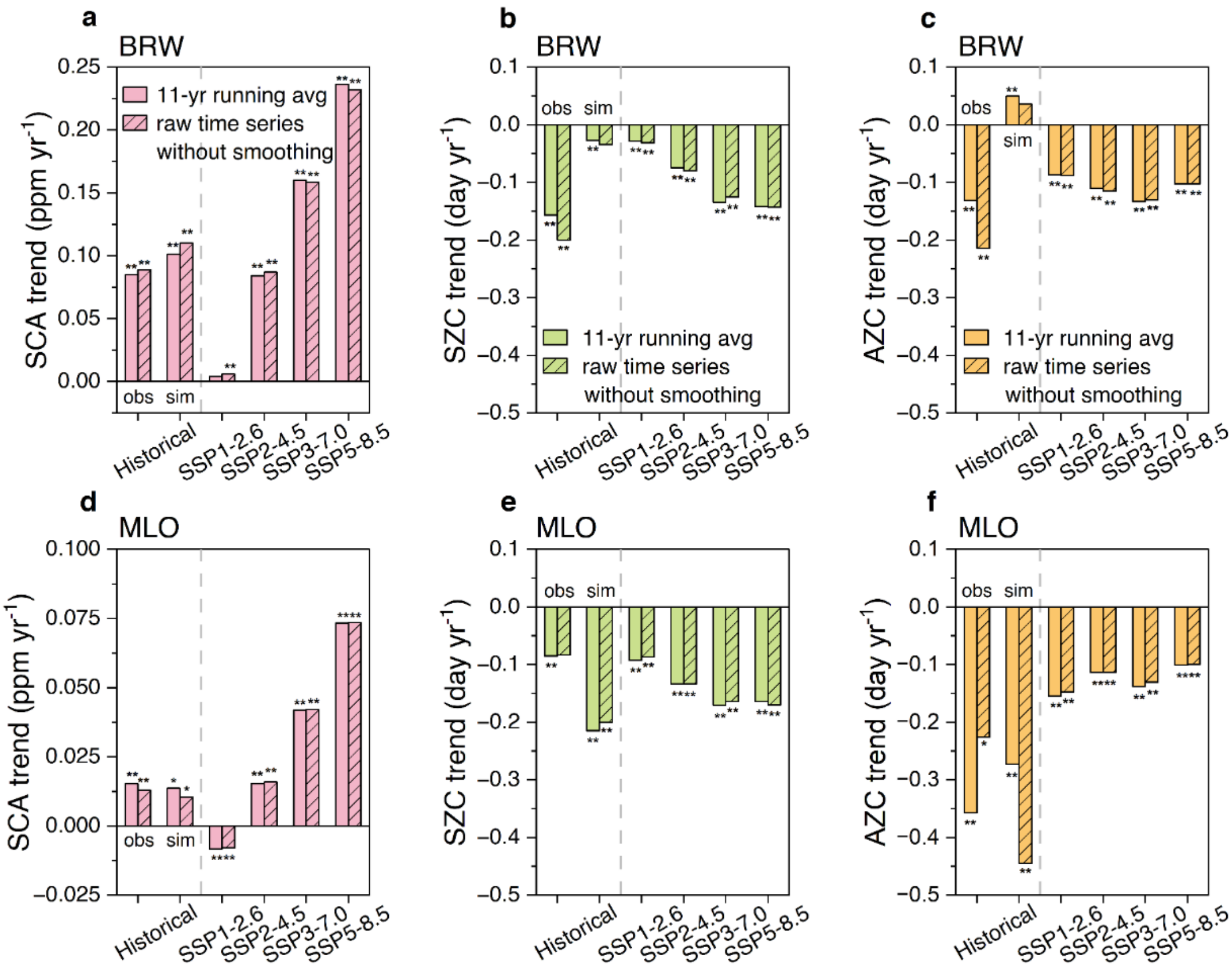

Supplementary Figure 36. Trends in the seasonality of atmospheric CO_2 under the historical period and four future scenarios at Barrow, Alaska (BRW; **a-c**), and Mauna Loa, Hawaii (MLO; **d-f**). "11-yr running avg" denotes trends calculated from 11-year running-mean time series of CO_2 seasonal parameters, whereas "raw time series without smoothing" denotes trends calculated from the original (unsmoothed) time series of the same parameters. The panels display the trends in SCA (left column), SZC (middle column), and AZC (right column). Statistically significant trends ($p < 0.05$) are marked by two dots above the bars, and marginally significant trends ($p < 0.1$) by one dot.

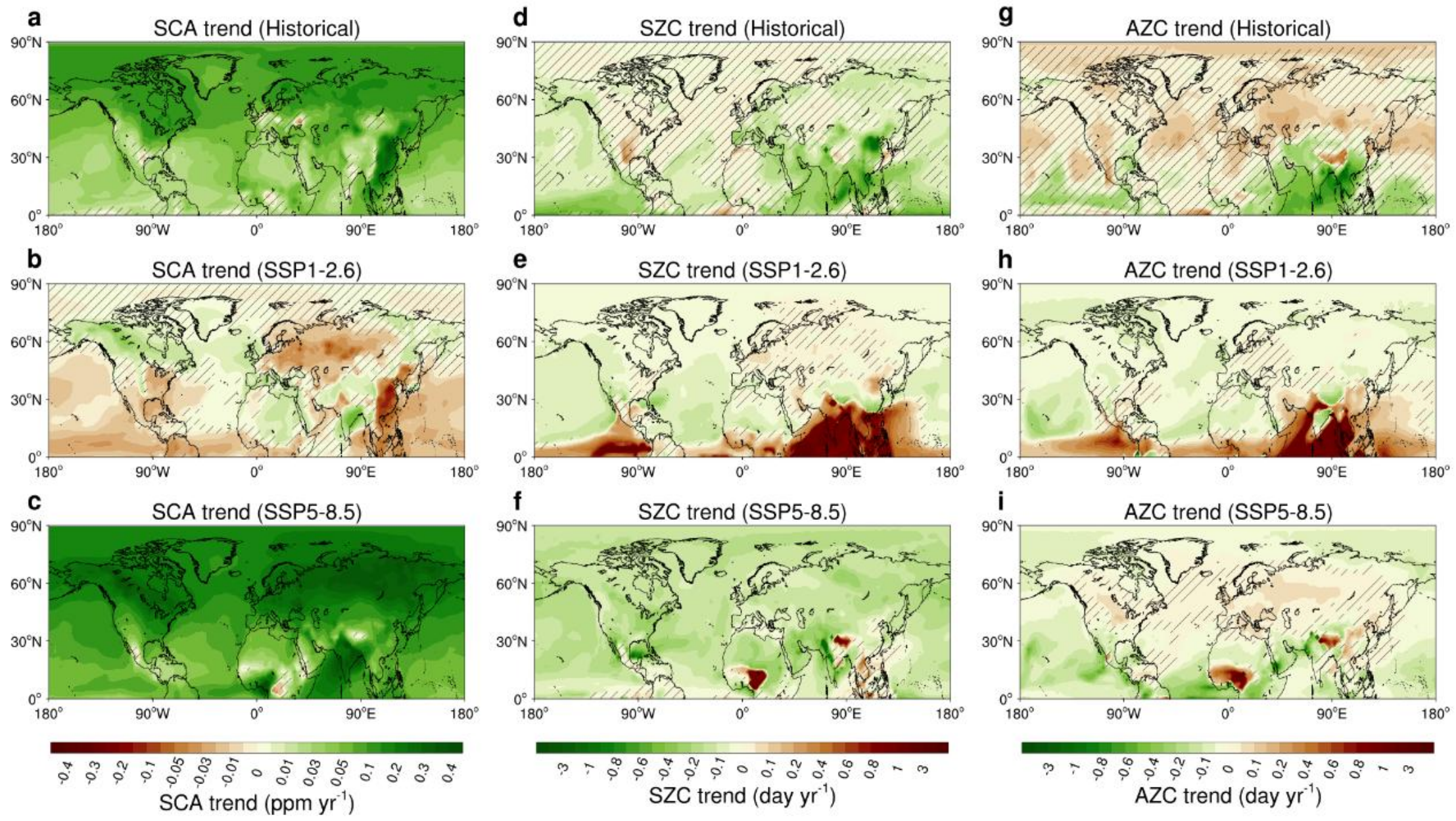

Supplementary Figure 37. Spatial patterns of simulated trends in atmospheric CO_2 seasonal parameters across the Northern Hemisphere under historical and future scenarios. Trends are calculated from the original time series of each seasonal parameter, rather than from 11-year running averages of the respective time series (Fig. 2). Simulated trends in seasonal cycle amplitude (SCA; **a-c**), spring zero-crossing date (SZC; **d-f**), and autumn zero-crossing date (AZC; **g-i**) for the historical period (top row), SSP1-2.6 scenario (second row), and SSP5-8.5 scenario (bottom row). Hatched areas denote regions where trends are not statistically significant ($p > 0.05$).

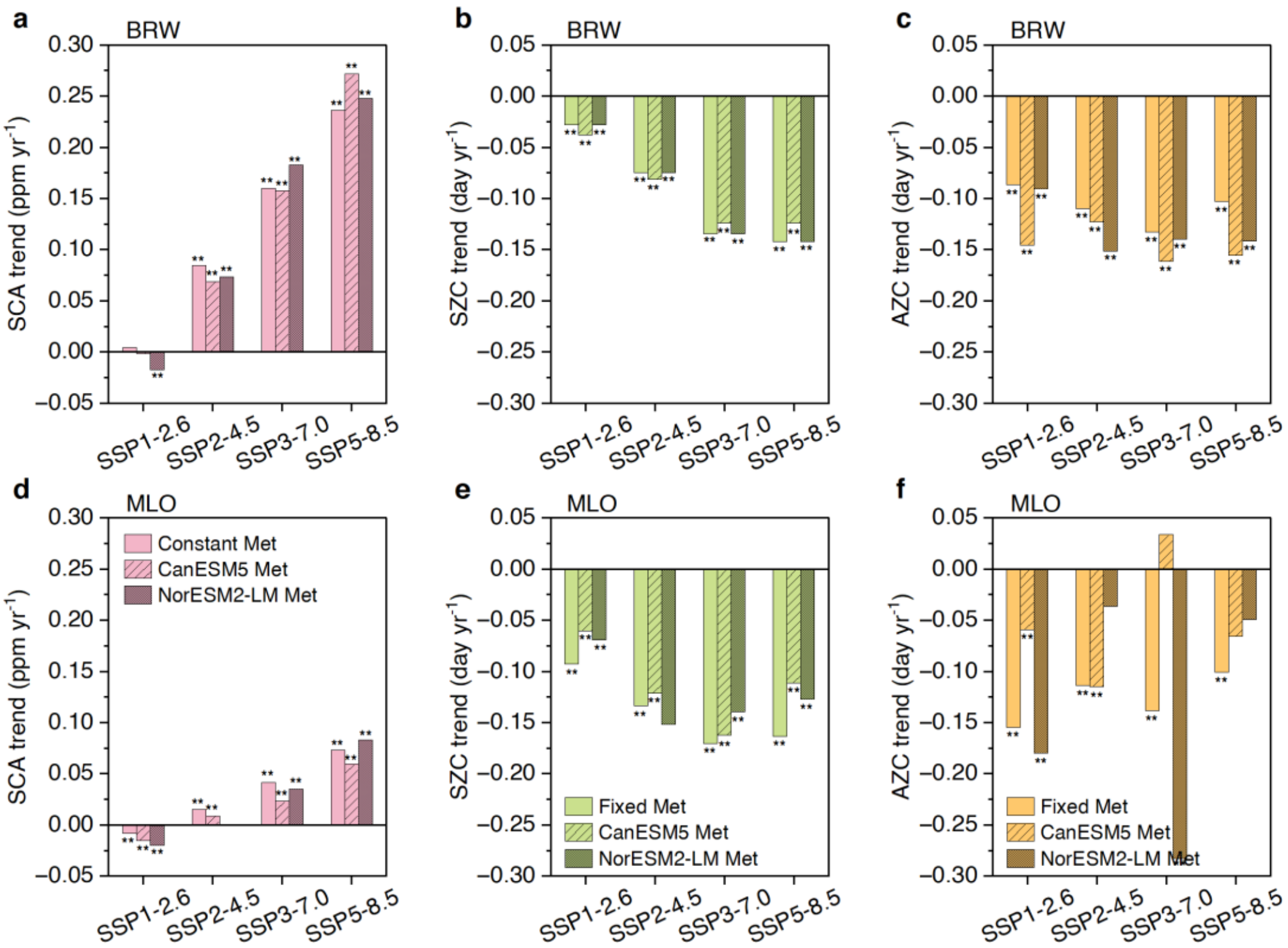

Supplementary Figure 38. Variations in the seasonality of atmospheric CO_2 under four future scenarios at the Barrow, Alaska (BRW; **a-c**), and Mauna Loa, Hawaii (MLO; **d-f**) stations. “Constant Met”, “CanESM5 Met”, and “NorESM2-LM Met” denote trends in CO_2 seasonal parameters simulated using constant meteorological fields in 2021 from MERRA-2, time-varying meteorological fields from CanESM5, and time-varying meteorological fields from NorESM2-LM, respectively. The panels display the trends in SCA (left column), SZC (middle column), and AZC (right column). Statistically significant trends ($p < 0.05$) are marked by two dots above the bars, and marginally significant trends ($p < 0.1$) by one dot.

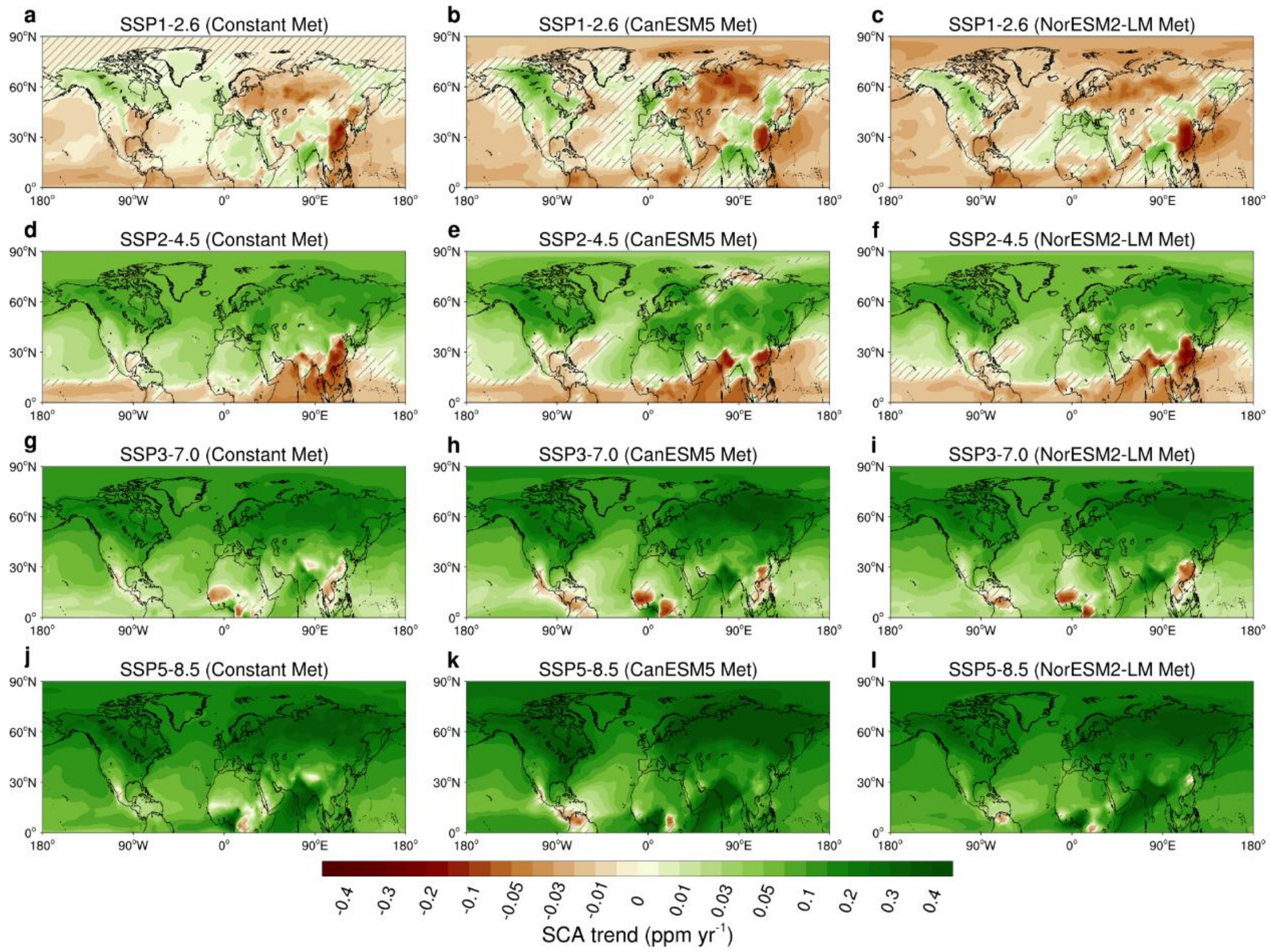

Supplementary Figure 39. Spatial patterns of simulated SCA trends across the Northern Hemisphere from 2030 to 2100 under different future scenarios, driven by different meteorological fields. Panels show SCA trends under SSP1-2.6 (**a-c**), SSP2-4.5 (**d-f**), SSP3-7.0 (**g-i**), and SSP5-8.5 (**j-l**), driven by fixed 2021 meteorological fields from MERRA-2 (left column), time-varying meteorological fields from CanESM5 (middle column), and time-varying meteorological fields from NorESM2-LM (right column). Hatched areas denote regions where trends are not statistically significant ($p > 0.05$).

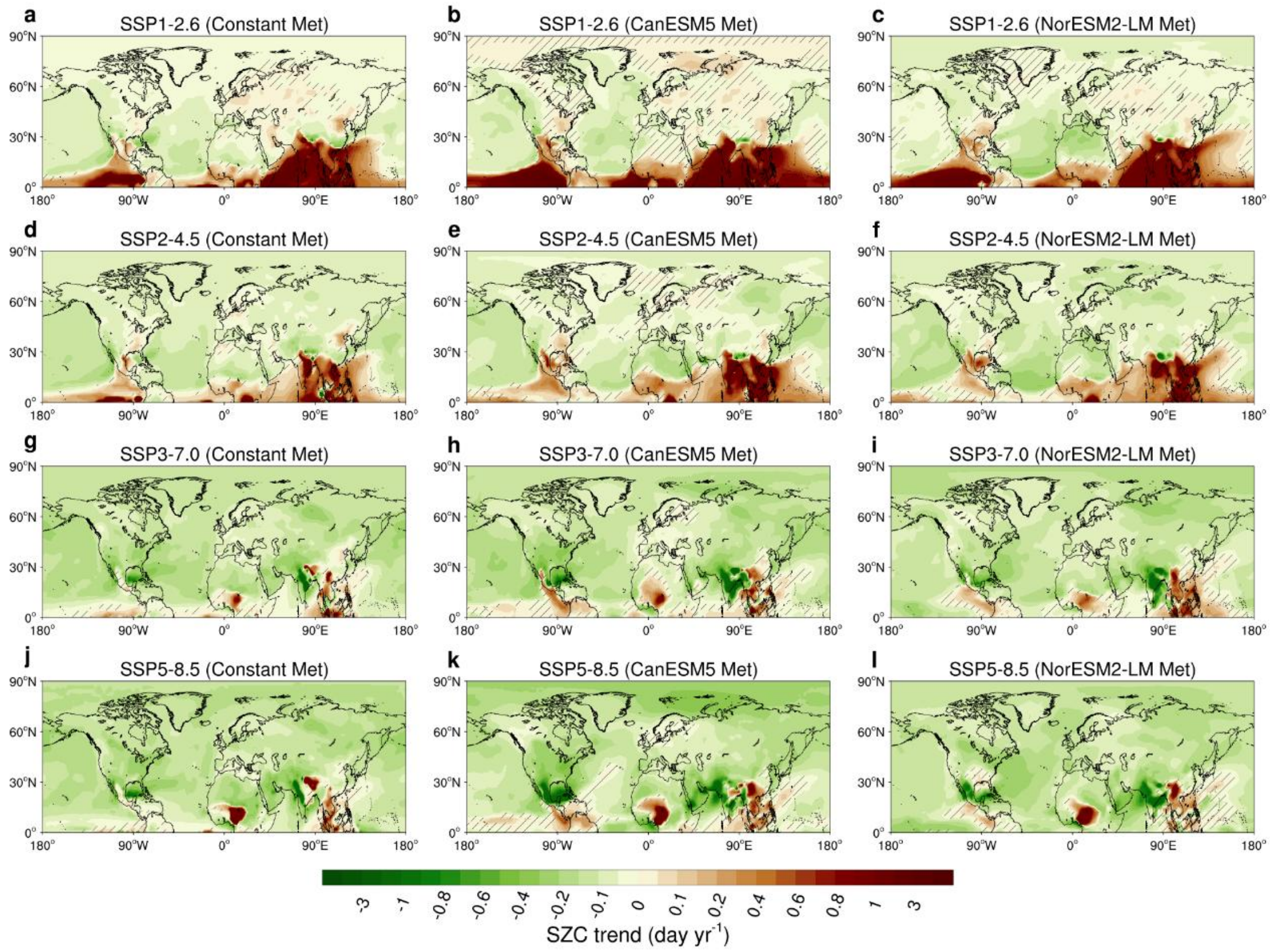

Supplementary Figure 40. Same as Supplementary Fig. 34, but showing the SZC trend.

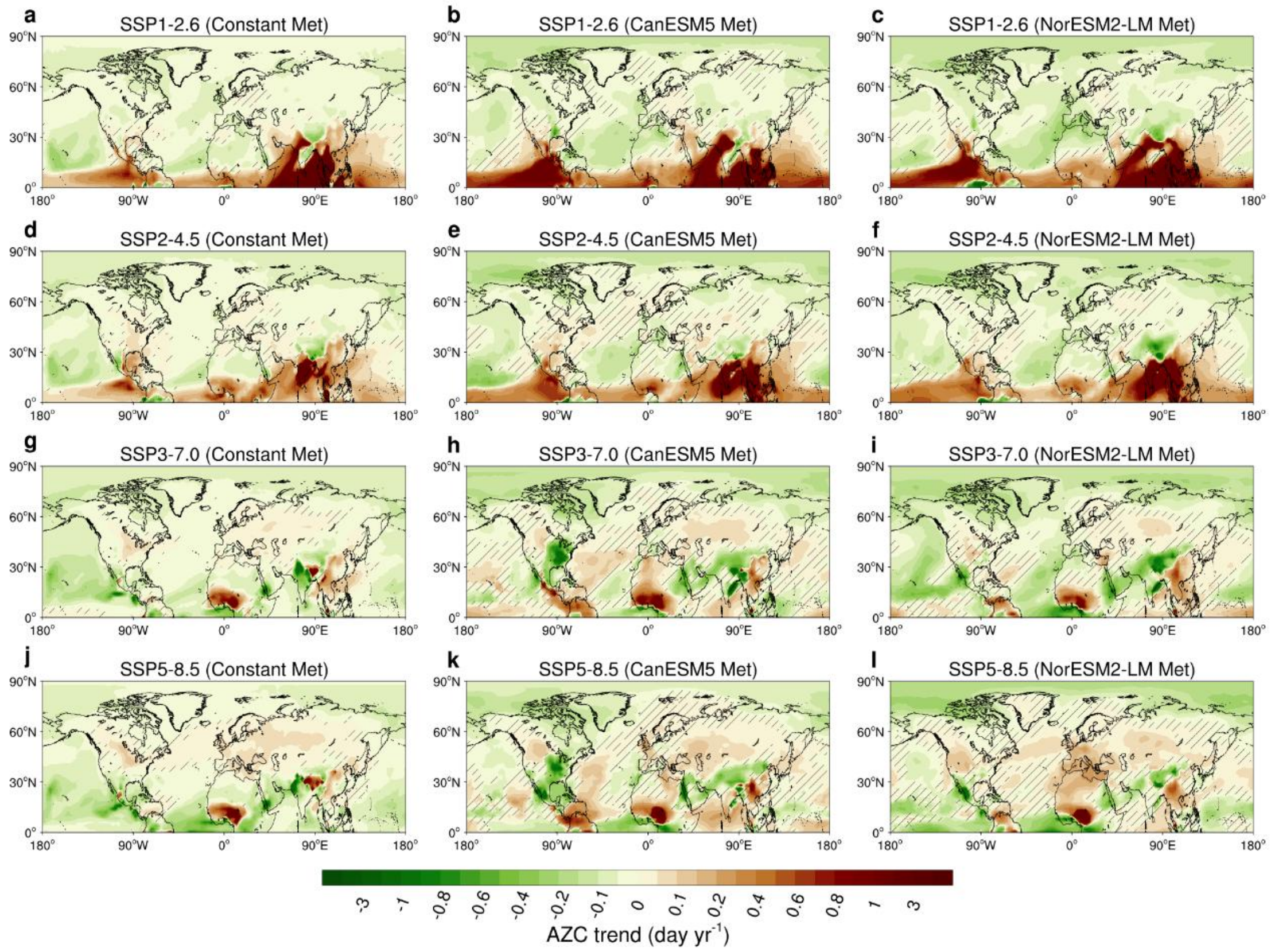

Supplementary Figure 41. Same as Supplementary Fig. 34, but showing the AZC trend.

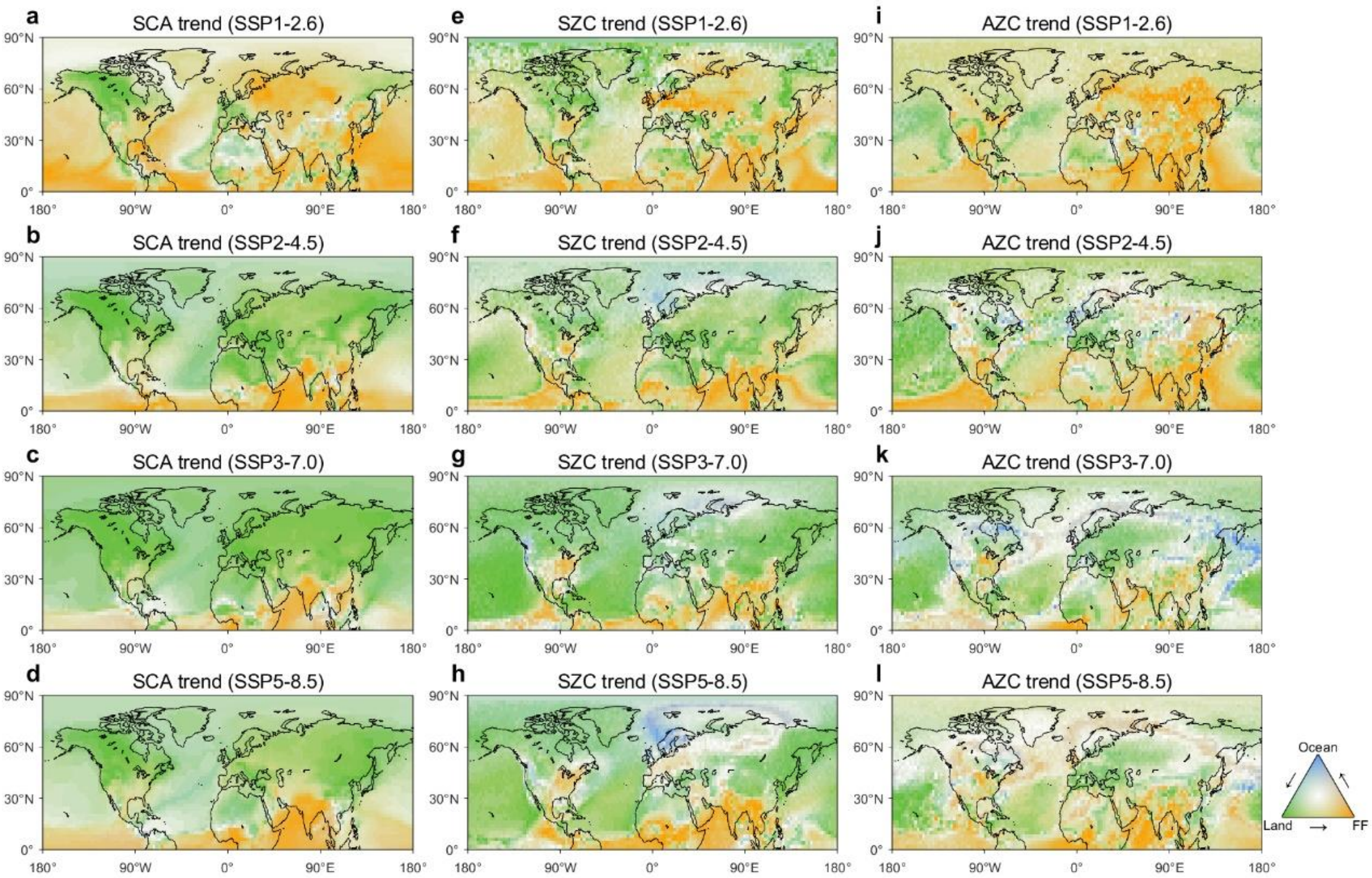

Supplementary Figure 42. Driven by time-varying meteorological fields from CanESM5, the relative proportions of net terrestrial CO_2 fluxes, fossil fuel CO_2 emissions, and air-sea CO_2 exchange contributing to trends in atmospheric CO_2 seasonal parameters across the Northern Hemisphere from 2030 to 2100 are shown under different future scenarios. Panels display the relative proportions of the three component fluxes associated with trends in SCA (**a-d**), SZC (**e-h**), and AZC (**i-l**) for the SSP1-2.6 (top row), SSP2-4.5 (second row), SSP3-7.0 (third row), and SSP5-8.5 (bottom row) scenarios.

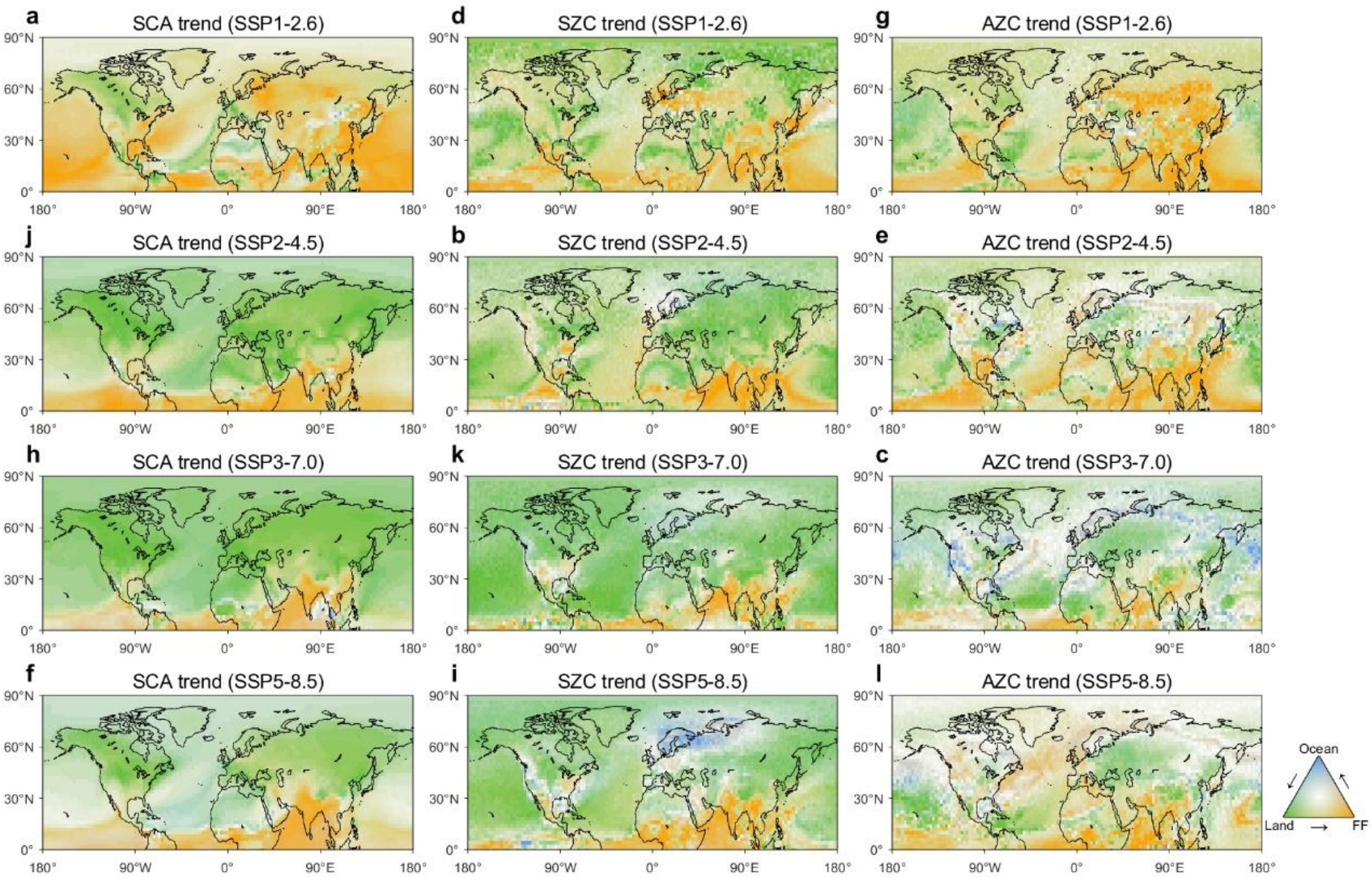

Supplementary Figure 43. Same as Supplementary Fig. 37, but showing the relative proportions of the three CO_2 fluxes from simulations driven by meteorological fields from NorESM2-LM.

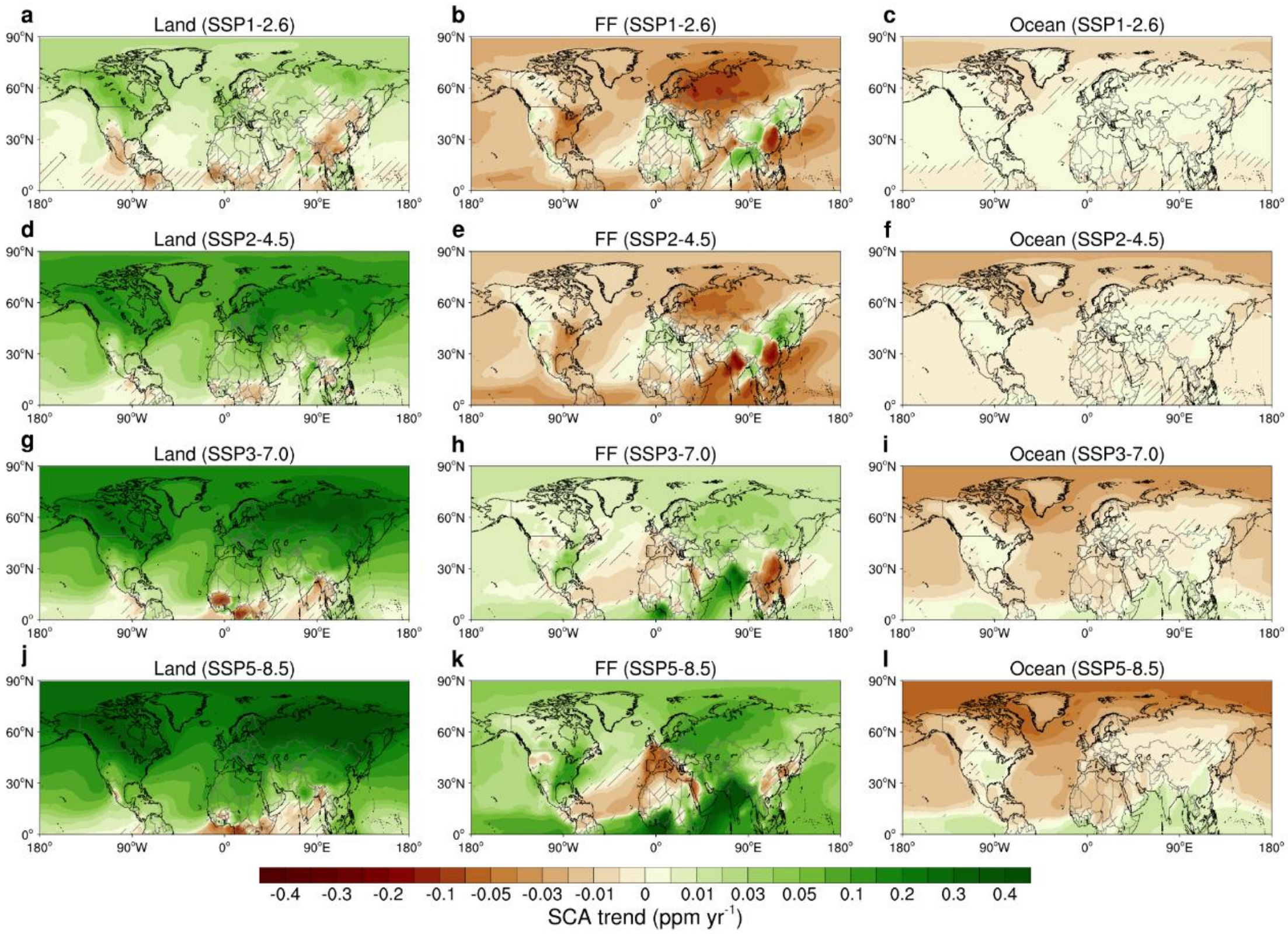

Supplementary Figure 44. Driven by time-varying meteorological fields from CanESM5, the spatial patterns of simulated SCA trends from 2030 to 2100 are attributed to variations in the terrestrial ecosystem (left panels), fossil fuel CO_2 emissions (middle panels), and air-sea CO_2 exchange (right panels). Results are shown for the SSP1-2.6 (**a-c**), SSP2-4.5 (**d-f**), SSP3-7.0 (**g-i**), and SSP5-8.5 (**j-l**) scenarios, estimated from the differences between the base simulations and the corresponding factorial simulations. The hatched areas indicate that the trends in these regions did not pass the significance test ($p > 0.05$).

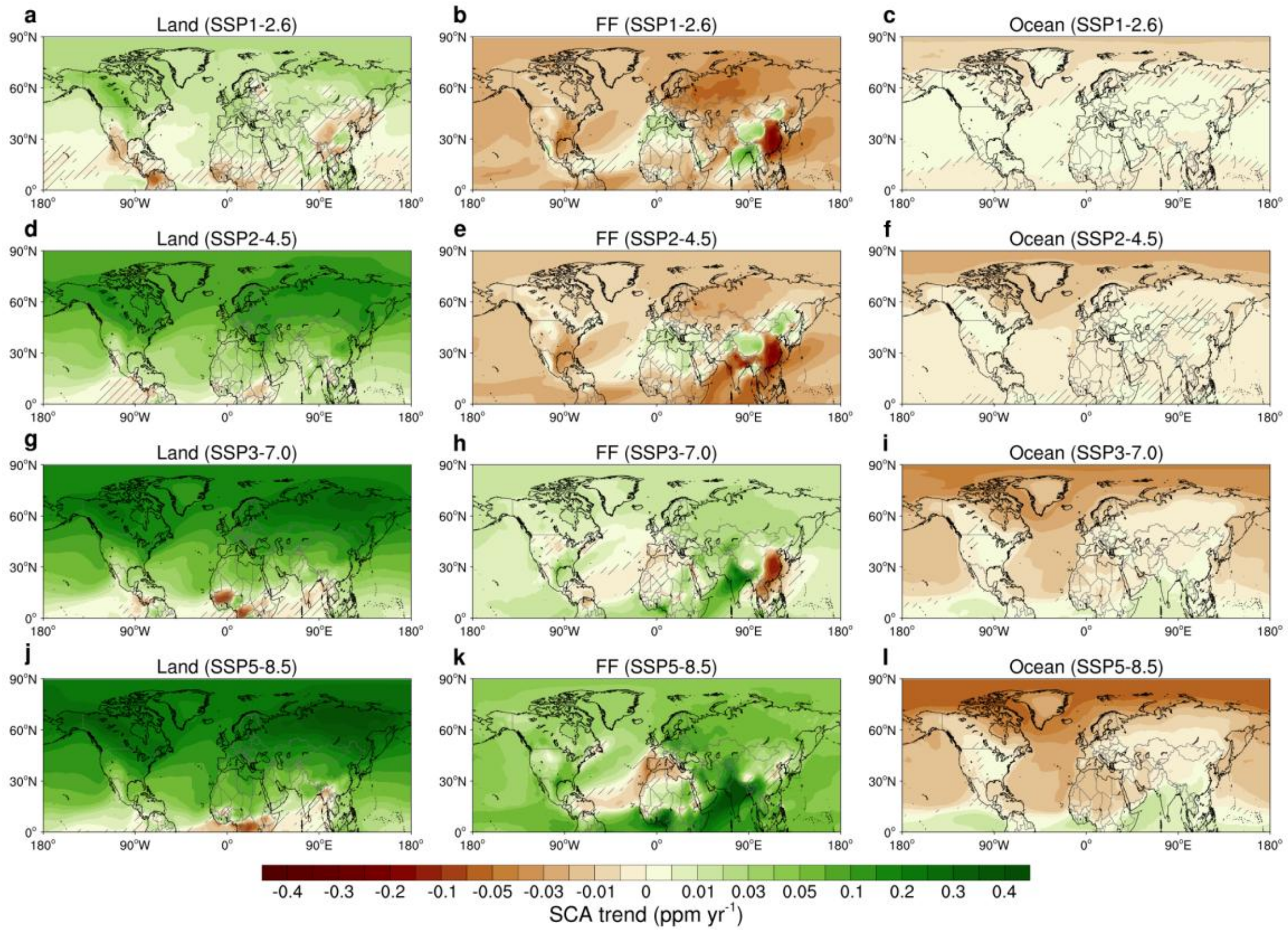

Supplementary Figure 45. Same as Supplementary Fig. 39, but showing the attribution of the SCA trend under simulations driven by meteorological fields from NorESM2-LM.

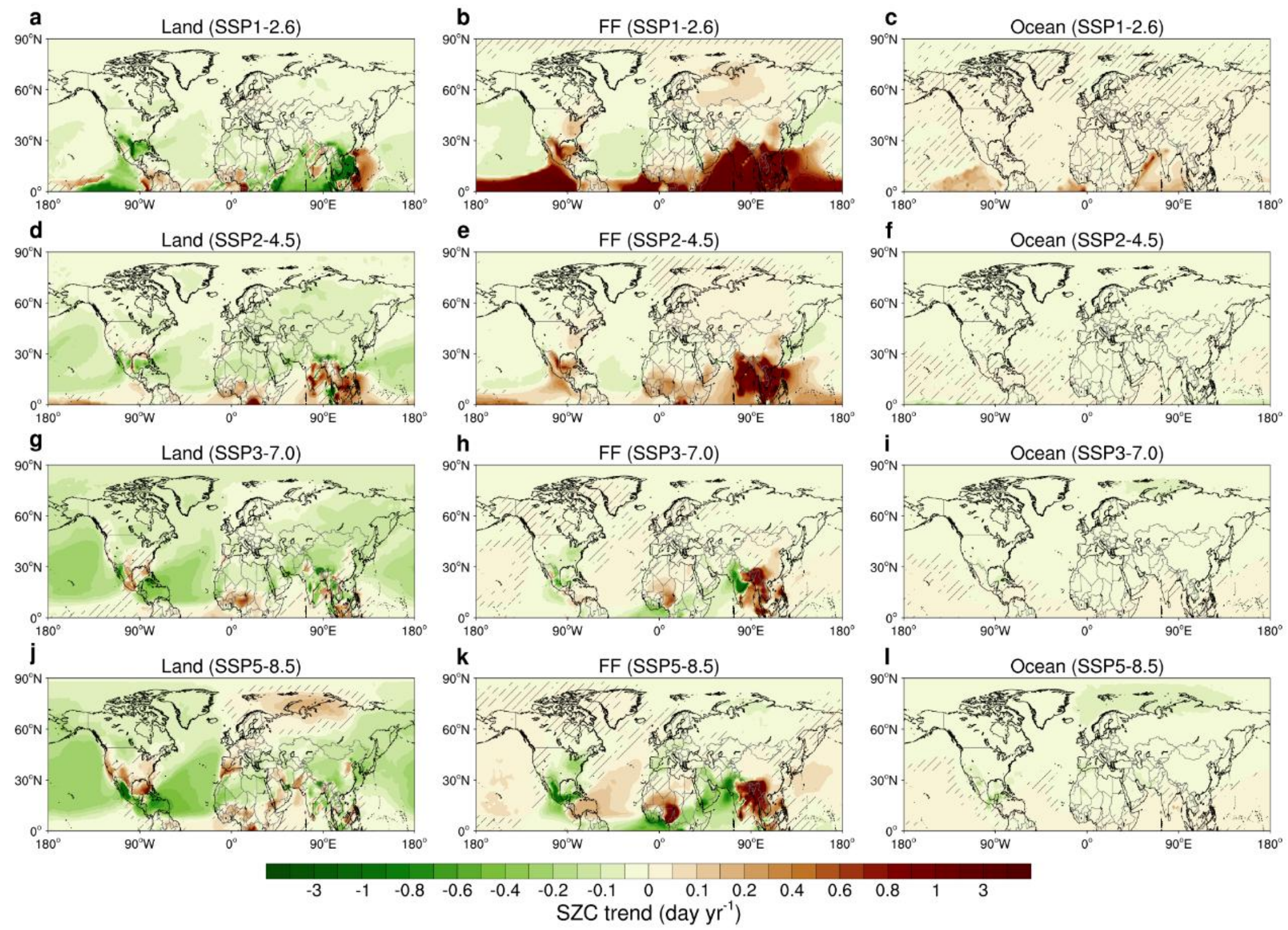

Supplementary Figure 46. Driven by time-varying meteorological fields from CanESM5, the spatial patterns of simulated SZC trends from 2030 to 2100 are attributed to variations in the terrestrial ecosystem (left panels), fossil fuel CO_2 emissions (middle panels), and air-sea CO_2 exchange (right panels). Results are shown for the SSP1-2.6 (**a-c**), SSP2-4.5 (**d-f**), SSP3-7.0 (**g-i**), and SSP5-8.5 (**j-l**) scenarios, estimated from the differences between the base simulations and the corresponding factorial simulations. The hatched areas indicate that the trends in these regions did not pass the significance test ($p > 0.05$).

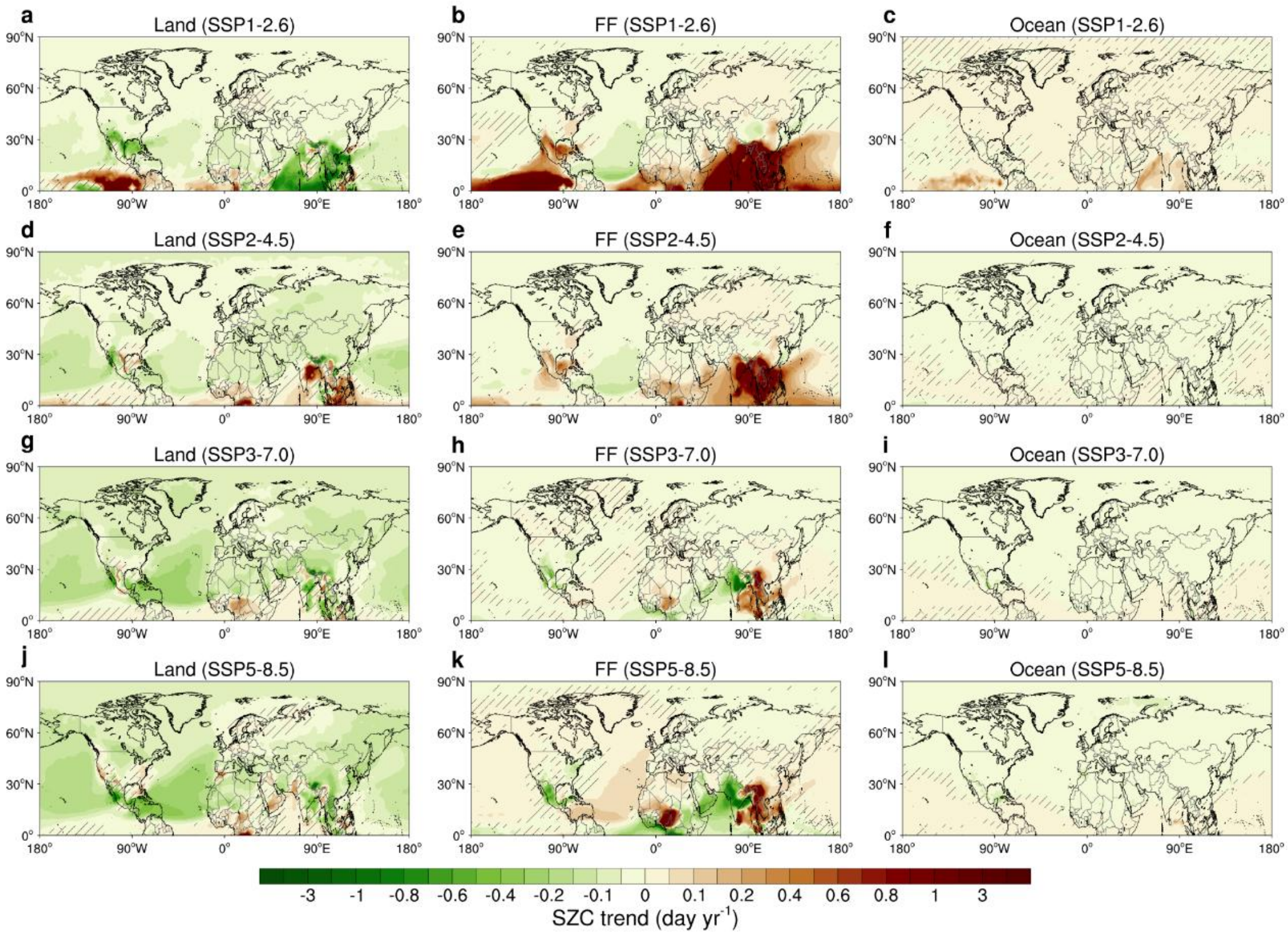

Supplementary Figure 47. Same as Supplementary Fig. 41, but showing the attribution of the SZC trend under simulations driven by meteorological fields from NorESM2-LM.

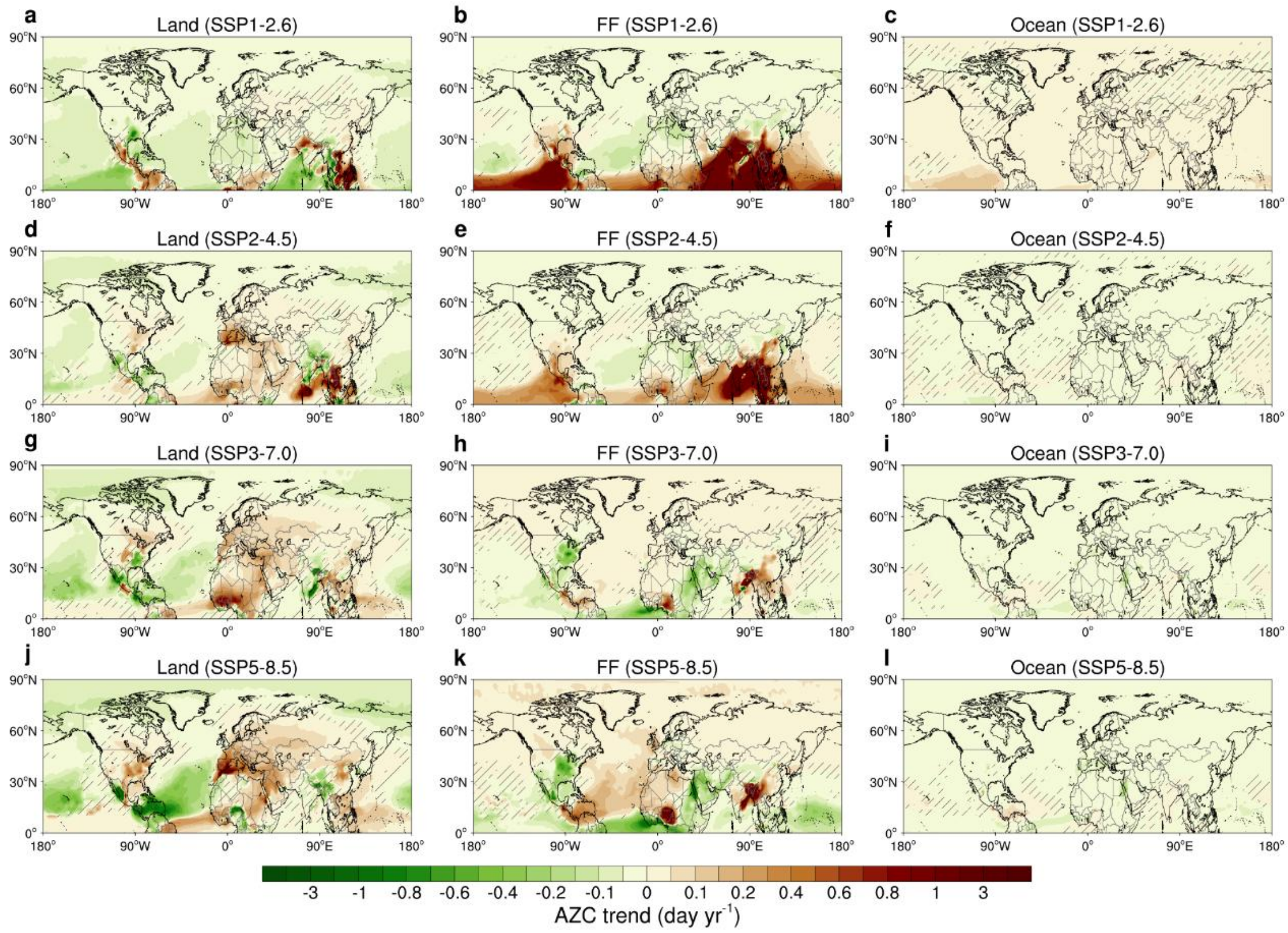

Supplementary Figure 48. Driven by time-varying meteorological fields from CanESM5, the spatial patterns of simulated AZC trends from 2030 to 2100 are attributed to variations in the terrestrial ecosystem (left panels), fossil fuel CO_2 emissions (middle panels), and air-sea CO_2 exchange (right panels). Results are shown for the SSP1-2.6 (**a-c**), SSP2-4.5 (**d-f**), SSP3-7.0 (**g-i**), and SSP5-8.5 (**j-l**) scenarios, estimated from the differences between the base simulations and the corresponding factorial simulations. The hatched areas indicate that the trends in these regions did not pass the significance test ($p > 0.05$).

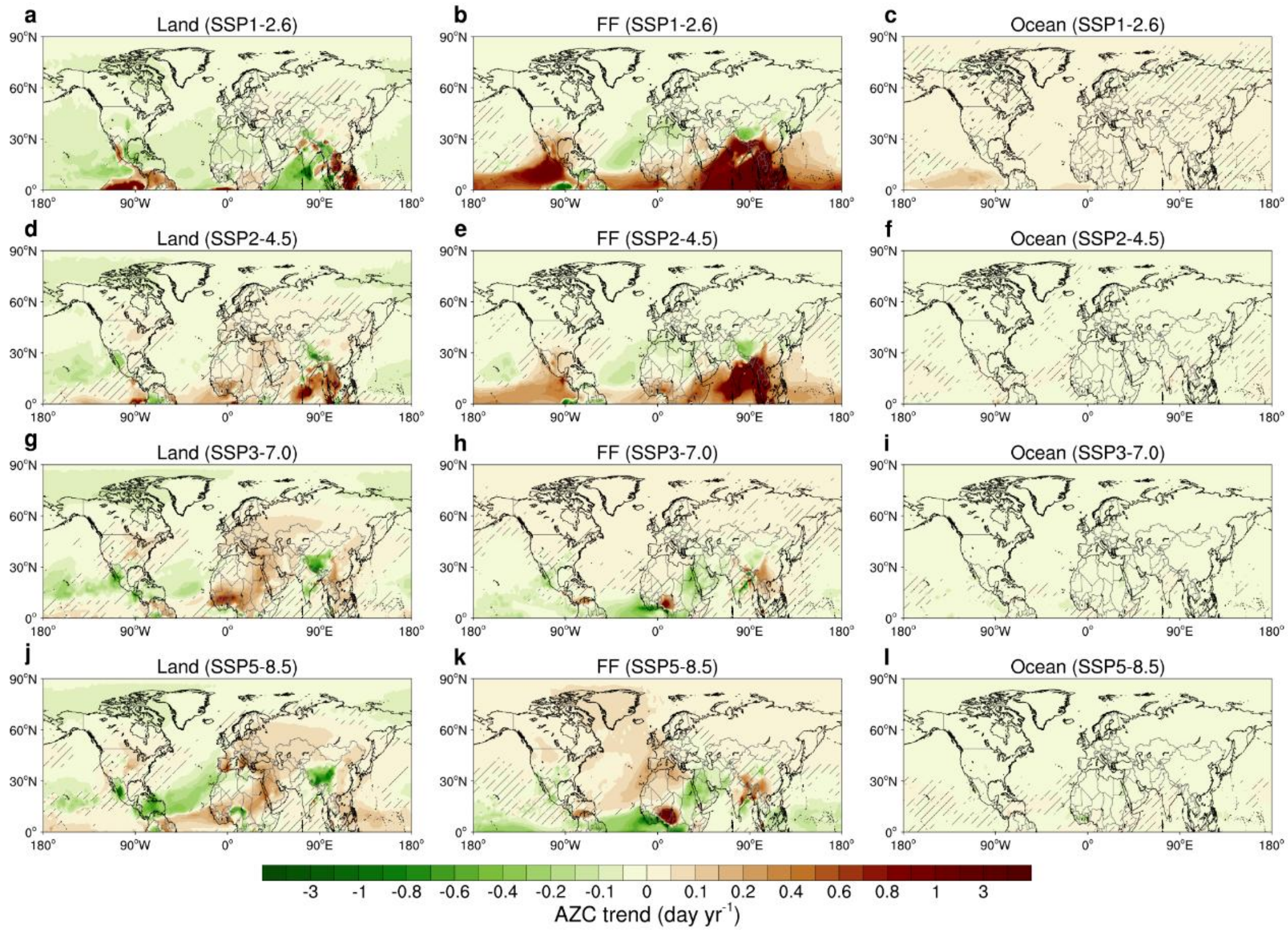

Supplementary Figure 49. Same as Supplementary Fig. 43, but showing the attribution of the AZC trend under simulations driven by meteorological fields from NorESM2-LM.

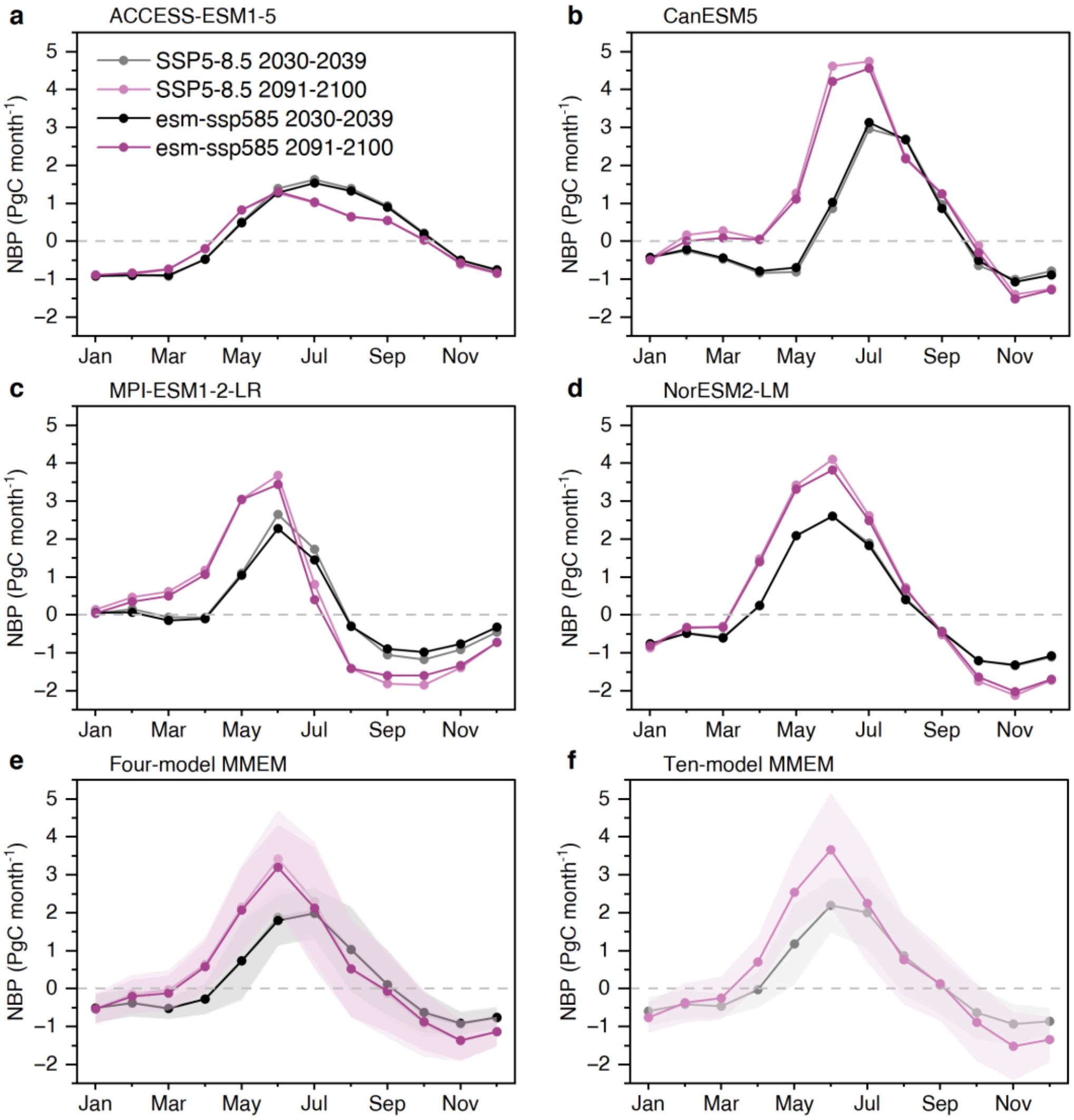

Supplementary Figure 50. Ten-year mean monthly NBP over the Northern Hemisphere for 2030-2039 and 2091-2100. Panels (a-d) show results from ACCESS-ESM1-5, CanESM5, MPI-ESM1-2-LR, and NorESM2-LM, respectively, and panel (e) shows their four-model multi-model ensemble mean (MMEM); all are shown under both the SSP5-8.5 and esm-ssp585 scenarios. Panel (f) shows the ten-model MMEM under the SSP5-8.5 scenario only. The shaded area represents the standard deviation across models. Note that MPI-ESM1-2-LR provides NBP data only through 2099, so the purple dotted lines in panel (c) represent the mean NBP for 2091-2099.

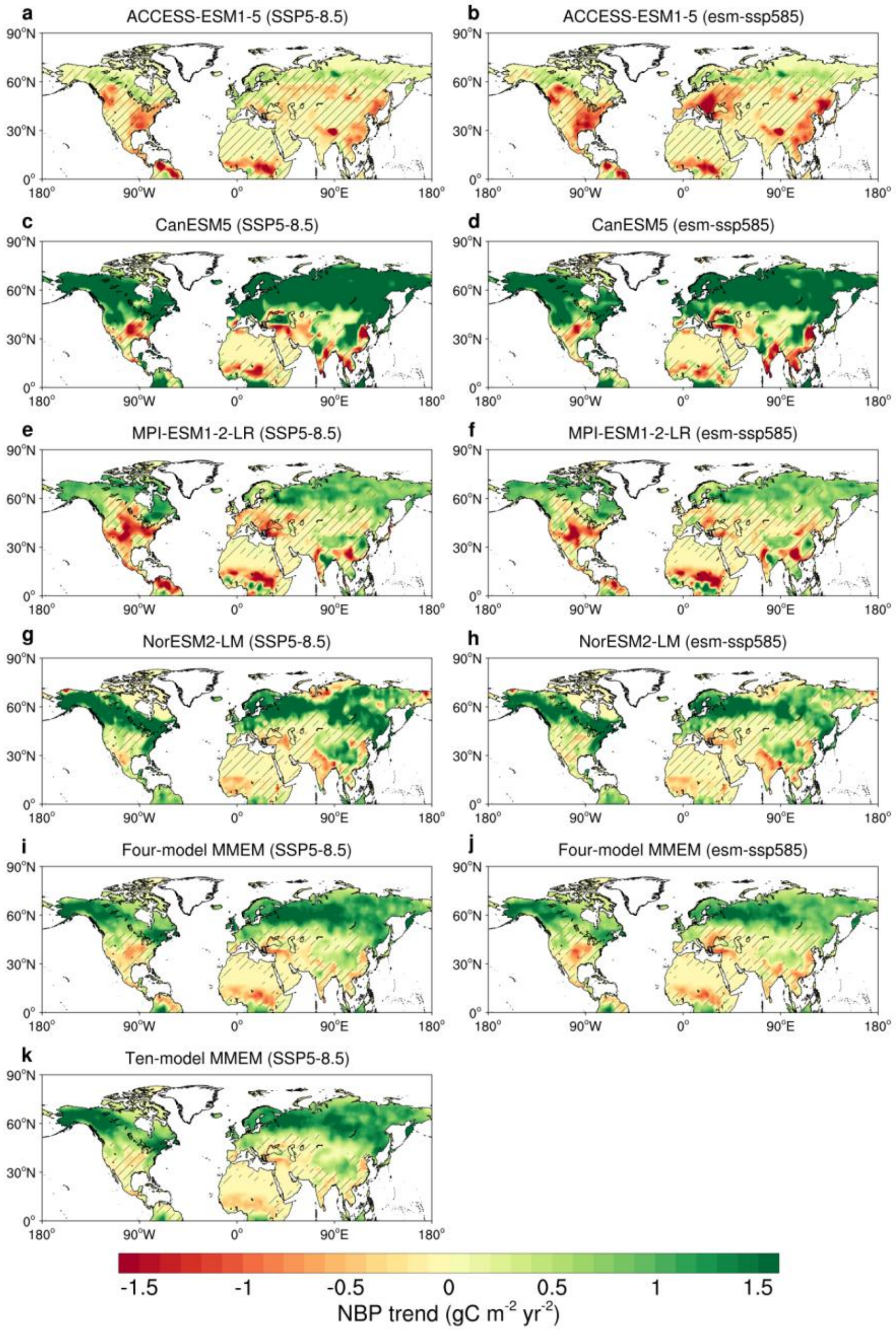

Supplementary Figure 51. Trends in integrated May-August NBP over 2030-2100 across the Northern Hemisphere. Panels show results from ACCESS-ESM1-5 (a, b), CanESM5 (c, d), MPI-ESM1-2-LR (e, f), NorESM2-LM (g, h), and their four-model multi-model ensemble mean (MMEM; i, j). Panel (k) shows the ten-model MMEM NBP trend under SSP5-8.5. The left column shows results under the SSP5-8.5 scenario, and the right column shows results under the esm-ssp585 scenario. The hatched areas indicate that the trends in these regions did not pass the significance test ($p > 0.05$). Note that MPI-ESM1-2-LR provides NBP data only through 2099, so the NBP trend from this model is calculated for 2091-2099.

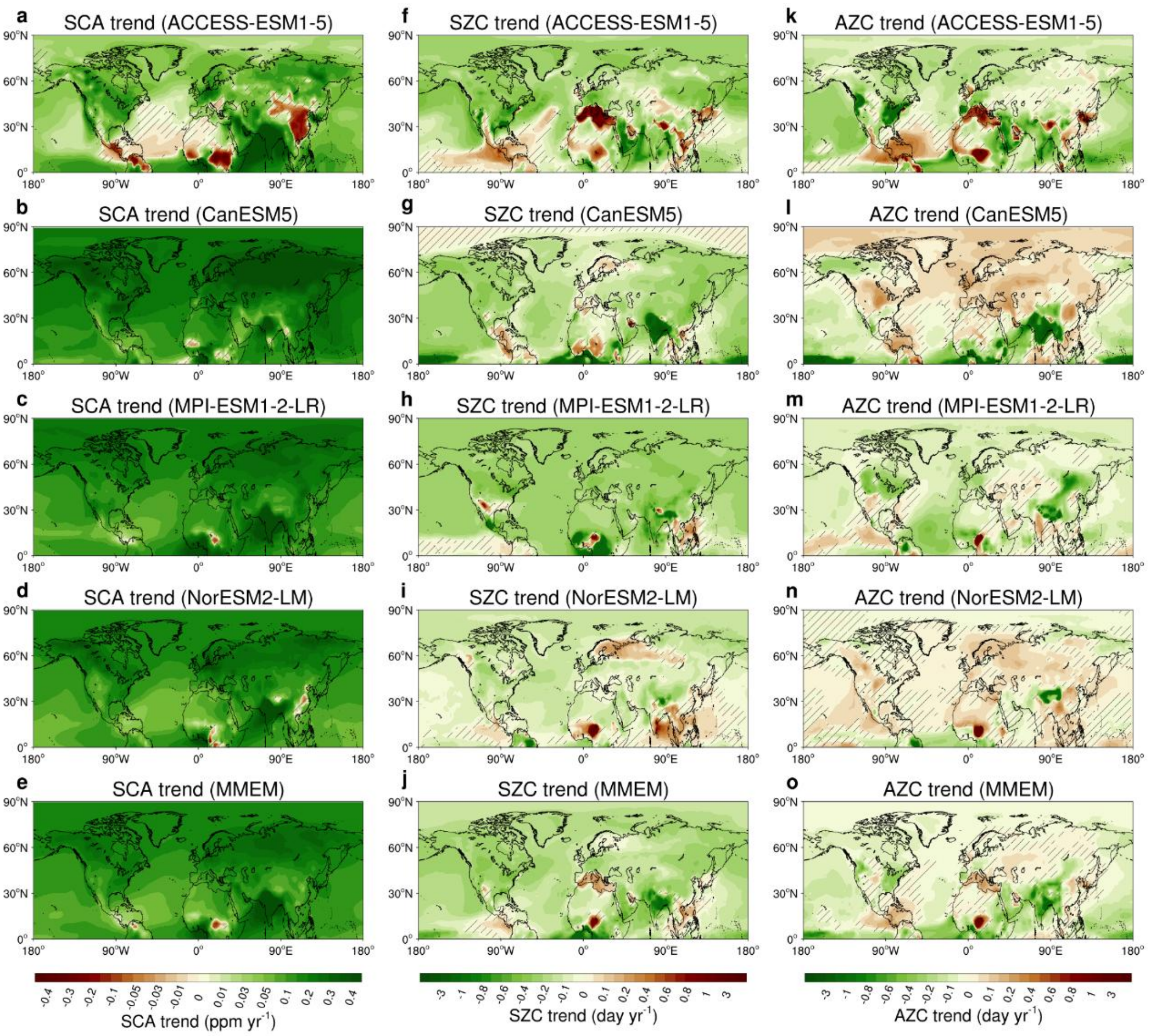

Supplementary Figure 52. Spatial patterns of simulated trends in SCA (**a-e**), SZC (**f-j**), and AZC (**k-o**) over 2030-2100 across the Northern Hemisphere from the esm-ssp585 simulations. Panels show results from ACCESS-ESM1-5 (first row), CanESM5 (second row), MPI-ESM1-2-LR (third row), NorESM2-LM (fourth row), and the ensemble-mean estimates from these four models (bottom row). The hatched areas indicate that the trends in these regions did not pass the significance test ($p > 0.05$). Note that MPI-ESM1-2-LR provides atmospheric CO_2 concentration data only through 2099, so the trends of CO_2 seasonal parameters from this model were calculated for 2030-2099.

Supplementary Table 1. Information about the 27 atmospheric CO_2 monitoring stations across the Northern Hemisphere used in this study.

**Station name**	**Abbreviation**	**Latitude**	**Longitude**	**Observation Period**
Alert, Nunavut	ALT	82.45	–62.51	1986–2021
Ny-Alesund, Svalbard	ZEP	78.91	11.89	1995–2021
Summit	SUM	72.60	–38.42	2001–2021
Barrow, Alaska	BRW	71.32	–156.61	1979–2021
Pallas-Sammaltunturi, GAW Station	PAL	67.97	24.12	2002–2021
Ocean Station M	STM	66.00	2.00	1981–2009
Storhofdi, Vestmannaeyjar	ICE	63.40	–20.29	1992–2021
Cold Bay, Alaska	CBA	55.21	–162.72	1979–2021
Mace Head, County Galway	MHD	53.33	–9.90	1992–2021
Shemya Island, Alaska	SHM	52.72	174.10	1986–2020
Hegyhatsal	HUN	46.95	16.65	1993–2021
Ulaan Uul	UUM	44.45	111.10	1993–2020
Niwot Ridge, Colorado	NWR	40.05	–105.63	1979–2021
Wendover, Utah	UTA	39.90	–113.72	1994–2021
Terceira Island, Azores	AZR	38.77	–27.38	1979–2021
Tae-ahn Peninsula	TAP	36.73	126.13	1991–2021
Mt. Waliguan	WLG	36.27	100.92	1992–2021
Tudor Hill, Bermuda	BMW	32.26	–64.88	1990–2021
WIS Station, Negev Desert	WIS	30.86	34.78	1996–2021
Sand Island, Midway	MID	28.22	–177.37	1986–2021
Key Biscayne, Florida	KEY	25.67	–80.20	1979–2021
Assekrem	ASK	23.26	5.63	1996–2019
Cape Kumukahi, Hawaii	KUM	19.56	–154.89	1979–2021
Mauna Loa, Hawaii	MLO	19.54	–155.58	1979–2021

Mariana Islands, Guam	GMI	13.39	144.66	1980–2019
Ragged Point, Barbados	RPB	13.16	–59.43	1988–2021
Christmas Island	CHR	1.70	–157.15	1979–2020

Supplementary Table 2. Overview of the fourteen dynamic global vegetation models (DGVMs) employed in this study. All fourteen models follow the TRENDY v11 protocol[1,2].

**Model**	**Spatial resolution**	**Period**	**Citation**
CLASSIC	1°×1°	1701–2021	Melton et al. (2020)[3]
CLM5.0	0.94°×1.25°	1701–2021	Lawrence et al. (2019)[4]
IBIS	1°×1°	1700–2021	Yuan et al. (2014)[5]
ISAM	0.5°×0.5°	1700–2021	Meiyappan et al. (2015)[6]
ISBA-CTRIP	1°×1°	1700–2021	Decharme et al. (2019)[7]
JSBACH	1.86°×1.875°	1701–2021	Reick et al. (2021)[8]
JULES	1.25°×1.875°	1700–2021	Wiltshire et al. (2021)[9]
LPJ	0.5°×0.5°	1700–2021	Sitch et al. (2003)[10]
LPX-Bern	0.5°×0.5°	1700–2021	Keel et al. (2016)[11]
OCN	1°×1°	1700–2021	Zaehle and Friend (2010)[12]
ORCHIDEE	0.5°×0.5°	1700–2021	Vuichard et al. (2019)[13]
SDGVM	1°×1°	1900–2021	Walker et al. (2017)[14]
VISIT-NIES	0.5°×0.5°	1700–2021	Ito (2019)[15]
VISIT	0.5°×0.5°	1860–2021	Kato et al. (2013)[16]

Supplementary Table 3. Information of CMIP6 Earth system models (ESMs) used in this study.

Model name	Institute	Land surface model	Citation
ACCESS-ESM1-5	CSIRO (Australia)	CABLE2.4	Ziehn et al. (2017)[17]
CanESM5	CCCMA (Canada)	CLASS3.6-CTEM1.2	Swart et al. (2019)[18]
CanESM5-1	CCCMA (Canada)	CLASS3.6-CTEM1.2	Swart et al. (2019)[18]
CESM2-WACCM	NCAR (USA)	CLM5	Danabasoglu et al. (2020)[19]
CMCC-ESM2	CMCC (Italy)	CLM4.5	Lovato et al. (2022)[20]
MPI-ESM1-2-LR	MPI (Germany)	JSBACH 3.2	Mauritsen et al. (2019)[21]
NorESM2-LM	NCC (Norway)	CLM5	Seland et al. (2020)[22]
NorESM2-MM	NCC (Norway)	CLM5	Seland et al. (2020)[22]
INM-CM4.8	INM (Russia)	INM-LND1	Volodin et al. (2018)[23]
INM-CM5.0	INM (Russia)	INM-LND1	Volodin et al. (2017)[24]

Supplementary Table 4. Availability of meteorological variables from the ten EMSs used in this study under the SSP1-2.6, SSP2-4.5, SSP3-7.0, and SSP5-8.5 scenarios, where √ indicates the variable is provided and ✕ indicates it is not provided.

	ACCESS-ESM1-5	CanESM5	CanESM5-1	CESM2-WACCM	CMCC-ESM2	MPI-ESM1-2-LR	NorESM2-LM	NorESM2-MM	INM-CM4.8	INM-CM5.0
ps	√	√	√	√	√	√	√	√	√	√
bldep	✕	√	✕	✕	✕	✕	√	√	√	√
ua	√	√	√	√	√	√	√	√	√	√
va	√	√	√	√	√	√	√	√	√	√
hus	√	√	√	√	√	√	√	√	√	√

Supplementary Table 5. Comparison of meteorological variables between MERRA-2 and CMIP6.

2-dimensional variable							
Variables in MERRA-2				Variables in CMIP6			
Abbreviation (standard name)	Unit	Frequency	-	Abbreviation (standard name)	Unit	Frequency	-
PS (surface_pressure)	Pa	3-hourly	-	ps (surface_air_pressure)	Pa	monthly	-
PBLH (planetary_boundary_layer_height)	m	hourly	-	bldep (atmosphere_boundary_layer_thickness)	m	monthly	-
3-dimensional variable							
Variables in MERRA-2				Variables in CMIP6			
Abbreviation (standard name)	Unit	Frequency	Vertical grid	Abbreviation (standard name)	Unit	Frequency	Vertical grid
U (eastward_wind)	m s^{-1}	3-hourly	hybrid sigma-pressure grid with 72 layers	ua (eastward_wind)	m s^{-1}	daily	8 layers at 1000, 850, 700, 500, 250, 100, 50, and 10 hPa
V (northward_wind)	m s^{-1}	3-hourly	hybrid sigma-pressure grid with 72 layers	va (northward_wind)	m s^{-1}	daily	8 layers at 1000, 850, 700, 500, 250, 100, 50, and 10 hPa
QV (specific_humidity)	kg kg^{-1}	3-hourly	hybrid sigma-pressure grid with 72 layers	hus (specific_humidity)	kg kg^{-1}	daily	8 layers at 1000, 850, 700, 500, 250, 100, 50, and 10 hPa

Supplementary References

1. Sitch, S. et al. Trends and drivers of terrestrial sources and sinks of carbon dioxide: An overview of the TRENDY project. *Glob. Biogeochem. Cycle* **38**, e2024GB008102 (2024).
2. Friedlingstein, P. et al. Global Carbon Budget 2022. *Earth Syst. Sci. Data* **14**, 4811-4900 (2022).
3. Melton, J. R. et al. CLASSIC v1.0: the open-source community successor to the Canadian Land Surface Scheme (CLASS) and the Canadian Terrestrial Ecosystem Model (CTEM) – Part 1: Model framework and site-level performance. *Geosci. Model Dev.* **13**, 2825-2850 (2020).
4. Lawrence, D. M. et al. The Community Land Model Version 5: Description of New Features, Benchmarking, and Impact of Forcing Uncertainty. *J. Adv. Model. Earth Syst.* **11**, 4245-4287 (2019).
5. Yuan, W. et al. Multiyear precipitation reduction strongly decreases carbon uptake over northern China. *J. Geophys. Res.-Biogeosci.* **119**, 881-896 (2014).
6. Meiyappan, P., Jain, A. K. & House, J. I. Increased influence of nitrogen limitation on CO2 emissions from future land use and land use change. *Glob. Biogeochem. Cycle* **29**, 1524-1548 (2015).
7. Decharme, B. et al. Recent Changes in the ISBA-CTRIP Land Surface System for Use in the CNRM-CM6 Climate Model and in Global Off-Line Hydrological Applications. *J. Adv. Model. Earth Syst.* **11**, 1207-1252 (2019).
8. Reick, C. H. et al. JSBACH 3-The land component of the MPI Earth System Model: documentation of version 3.2. (2021).
9. Wiltshire, A. J. et al. JULES-CN: a coupled terrestrial carbon–nitrogen scheme (JULES vn5.1). *Geosci. Model Dev.* **14**, 2161-2186 (2021).
10. Sitch, S. et al. Evaluation of ecosystem dynamics, plant geography and terrestrial carbon cycling in the LPJ dynamic global vegetation model. *Glob. Change Biol.* **9**, 161-185 (2003).
11. Keel, S. G. et al. Simulating oxygen isotope ratios in tree ring cellulose using a dynamic global vegetation model. *Biogeosciences* **13**, 3869-3886 (2016).
12. Zaehle, S. & Friend, A. D. Carbon and nitrogen cycle dynamics in the O-CN land surface model: 1. Model description, site-scale evaluation, and sensitivity to parameter estimates. *Glob. Biogeochem. Cycle* **24** (2010).
13. Vuichard, N. et al. Accounting for carbon and nitrogen interactions in the global terrestrial ecosystem model ORCHIDEE (trunk version, rev 4999): multi-scale evaluation of gross primary production. *Geosci. Model Dev.* **12**, 4751-4779 (2019).
14. Walker, A. P. et al. The impact of alternative trait-scaling hypotheses for the maximum photosynthetic carboxylation rate (Vcmax) on global gross primary production. *New Phytol.* **215**, 1370-1386 (2017).
15. Ito, A. Disequilibrium of terrestrial ecosystem CO2 budget caused by disturbance-induced emissions and non-CO2 carbon export flows: a global model assessment. *Earth Syst. Dynam.* **10**, 685-709 (2019).
16. Kato, E., Kinoshita, T., Ito, A., Kawamiya, M. & Yamagata, Y. Evaluation of spatially explicit emission scenario of land-use change and biomass burning using a process-based biogeochemical model. *J. Land Use Sci.* **8**, 104-122 (2013).
17. Ziehn, T., Lenton, A., Law, R. M., Matear, R. J. & Chamberlain, M. A. The carbon cycle in the Australian Community Climate and Earth System Simulator (ACCESS-ESM1) – Part 2: Historical simulations. *Geosci. Model Dev.* **10**, 2591-2614 (2017).

18 Swart, N. C. et al. The Canadian Earth System Model version 5 (CanESM5.0.3). *Geosci. Model Dev.* **12**, 4823-4873 (2019).

19 Danabasoglu, G. et al. The Community Earth System Model Version 2 (CESM2). *J. Adv. Model. Earth Syst.* **12** (2020).

20 Lovato, T. et al. CMIP6 Simulations With the CMCC Earth System Model (CMCC-ESM2). *J. Adv. Model. Earth Syst.* **14**, e2021MS002814 (2022).

21 Mauritsen, T. et al. Developments in the MPI-M Earth System Model version 1.2 (MPI-ESM1.2) and Its Response to Increasing CO2. *J. Adv. Model. Earth Syst.* **11**, 998-1038 (2019).

22 Seland, Ø. et al. Overview of the Norwegian Earth System Model (NorESM2) and key climate response of CMIP6 DECK, historical, and scenario simulations. *Geosci. Model Dev.* **13**, 6165-6200 (2020).

23 Volodin, E. M. et al. Simulation of the modern climate using the INM-CM48 climate model. *Izv. Atmos. Ocean. Phys.* **33**, 367-374 (2018).

24 Volodin, E. M. et al. Simulation of the present-day climate with the climate model INMCM5. *Clim. Dyn.* **49**, 3715-3734 (2017).
