# Supplementary material for: Fossil fuel emissions dominate Northern Hemisphere CO2 seasonal cycle trends under mitigation scenarios
Source: Nat Commun. 2026 Jul 1;17:8201. doi: 10.1038/s41467-026-75003-x (PMC13462122; doi:10.1038/s41467-026-75003-x)
Supplement: Supplementary file 2 [file 41467_2026_75003_MOESM2_ESM.pdf]

nature portfolio

Peer Review File

Fossil fuel emissions dominate Northern Hemisphere CO2 seasonal cycle trends under mitigation scenarios

Corresponding Author: Dr Yue He

This manuscript has been previously reviewed at another journal. This document only contains information relating to versions considered at Nature Communications.

This file contains all reviewer reports in order by version, followed by all author rebuttals in order by version.

Version 0:

Reviewer comments:

Reviewer #1

(Remarks to the Author)
This work has not a prospect to get published in the journal for the reasons as indicated below.

1) Language and structure: The article contains errors and inconsistencies in sentence structure.

2) Discussion of limitations: The article should have discussed the limitations of the research methodology in more detail and address potential biases in the results due to parameter choices

3) The research methods adopted in this study should have been briefly introduced and clearly stated the significance of this study

4) Authors always have to write a paragraph saying: "The rest of the paper is organized as follows. Section 2 contains the literature review. Section 3 contains the methodology (method). Section 4 contains the results. Section 5 contains the conclusions and recommendations". So, the reader knows what's coming next

5) Even though authors can send unformat papers for evaluation, it is recommended to send the manuscript with the following format structure: (1) Abstract, (2) Introduction, (3)Experimental, (4)Results and discussion, (5)Conclusion, and (6)References. With a scientific format reviewers are able to evaluate the proposed paper with more accuracy and faster. Please, think about in your future attempt

6) A concise and factual abstract and conclusion should have been provided

7) Author should have compared the results with literature. As it is not possible, tried to highlight the importance and innovation of the study.

(Remarks on code availability)

Reviewer #2

(Remarks to the Author)
This manuscript assessed and attributed potential changes in the CO2 seasonal cycle under four future scenarios through 2100, based on the atmospheric transport modeling combined with factorial simulations. And they found that under the low-emission scenario (SSP1-2.6), the decline in SCA is predominantly driven by fossil fuel emissions rather than land sinks. Overall, the written is well organized, However, for a journal like NC, the reliability and significance of this work are still open to question.

Major comments:
(1) You detrended the CO2 time series by a curve fitting method. Here I wonder whether different detrend methods can induce uncertainties when you calculating the date of SCZ and AZC.
(2) You used the GOES-Chem to simulate the global 3-D atmospheric CO2. However,
- (a) you did not mention how to initialize the 3-D CO2 when you ran the model starting from 1979 and 2030.
- (b) Importantly, after reading the entire manuscript, I noticed that the future simulations were conducted using constant meteorological fields from 2021. This assumption seems unreasonable, as atmospheric circulation patterns are expected to differ across scenarios. Would it be possible to force the transport model with scenario-specific climate data instead?
- (c) In the future simulations, you used the ensemble-mean carbon fluxes from ten CMIP6 models. However, please note that each model exhibits its own seasonal cycle in both climate and the carbon cycle. Therefore, it would be more appropriate to run the simulations using each model's individual carbon fluxes. You could then calculate the SCA for each case and present the ensemble mean and associated uncertainties.
-(d) You mentioned that you used ten ESMs, but as far as I know, in the SSP1-2.6, SSP2-4.5, SSP3-7.0, and SSP5-8.5 scenarios, most of these models are forced with prescribed CO_2 concentrations and satellite-derived climatological LAI. For example, if the model uses CLM as the land component, these future simulations are typically conducted in this manner (although you should verify this). Only the esmSSP585 experiment requires the models to run with fully interactive carbon–climate feedbacks, including dynamic global vegetation. If your simulations are based on the former type of runs, then the resulting projections of terrestrial carbon sinks in the future might be unrealistic.
(3) I understand that you calculated the SCA as the difference between the maximum and minimum CO_2 mole fractions within each calendar year. If so, when you conducted the factorial simulations by isolating the carbon fluxes from land, ocean, and fossil fuel emissions (FFE), the derived SCA in each experiment would represent different physical meanings. In your baseline simulation, for instance, the SCA might be determined by the difference between April and September CO_2 levels, while in the CMIP6_FF experiment, the timing of the maximum and minimum could shift, potentially occurring in different months. I am not sure whether I have understood this correctly — could you please clarify how you defined and compared the SCAs across different factorial simulations?
(4) You conclude that in SSP1-2.6, the decline in SCA is predominantly driven by fossil fuel emissions rather than land sinks. However, this conclusion critically depends on the accuracy of the seasonal cycle of fossil fuel emissions (FFE). Although you present some results for Europe, China, and the United States, I can confirm that the variation over China shown in Supplementary Figure 10e is incorrect. According to datasets such as MEIC and CDIAC, FFE over China typically peaks in December and reaches a minimum in January or February. Therefore, before drawing this conclusion, you need to first examine and compare the seasonal variations of FFE in several key regions between the datasets you used and other widely recognized inventories (e.g., MEIC, CDIAC). Otherwise, the validity of this conclusion remains questionable.
(5) In SSP2-4.5, fossil fuel emissions (FFE) also decline, but the results do not consistently show that FFE dominates the decrease in SCA. Therefore, the statement in Lines 171–173 — "reduced fossil fuel emissions generally contribute to the decline in SCA" — appears too strong and should be toned down. In addition, the phrase in Lines 288–289 — "broadening from an ecological proxy to a critical metric for tracking global climate policy and its impacts" — seems somewhat exaggerated and would benefit from a more cautious expression.
Some minors:
(1) Line 38, can also refers to "Zeng, N., Zhao, F., Collatz, J., Kalnay, E., Salawitch, R. J., West, T. O., & Guanter, (2014). Agricultural Green Revolution as a driver of increasing atmospheric CO2 seasonal amplitude. Nature, 515(7527), 394-397. https://www.ncbi.nlm.nih.gov/pubmed/25409829"
(2) Line 379: CMIP5_FF -> CMIP6_FF
(3) In Supplementary Figure 2, it would be helpful to include some statistical metrics, such as RMSE or correlation coefficients, to quantitatively illustrate the performance of the GEOS-Chem simulations.

(Remarks on code availability)

Reviewer #3

(Remarks to the Author)
This manuscript investigates how future emission scenarios affect the amplitude and phase of the atmospheric CO2 seasonal cycle in the Northern Hemisphere, and identifies the key contributing fluxes (land biosphere, ocean, fossil fuel). The authors use GEOS-Chem simulations driven by state-of-the-art CMIP6 projections, providing valuable insights into a topic relevant to carbon-cycle research and emission monitoring. The main conclusions that terrestrial fluxes dominate seasonal-cycle amplification under high-emission scenarios, whereas reduced fossil fuel seasonality drives the seasonal amplitude decrease under strong mitigation (SSP1-2.6)—are important, novel, and challenge the conventional understanding linking seasonal cycles predominantly to terrestrial biosphere changes. This makes the study potentially significant to the broader atmospheric sciences and climate policy communities.
However, there are several methodological and interpretational issues that must be addressed before the manuscript can be considered suitable for publication. Thus, I recommend major revision, focusing on the following points:
Major points:
1.Fixed future meteorology: The decision to use fixed 2021 meteorology for future scenario simulations isolates flux-driven changes but ignores potentially important atmospheric responses (e.g., boundary layer height, atmospheric circulation, mixing). This assumption likely influences amplitude and phase results. Please explicitly evaluate this assumption through sensitivity tests (e.g., alternative meteorological years or projected climate-model meteorology) to demonstrate that your findings are robust against this simplification.
2.Representation of fossil fuel emission seasonality: Your key conclusion relies strongly on the projected reduction of fossil fuel emission seasonality derived from IAM scenarios. Future changes in heating methods, renewables penetration, or

sectoral shifts may substantially alter these monthly emission patterns. Please provide at least one sensitivity experiment with alternative seasonal distributions (such as fixed 2030 patterns scaled by yearly totals or adjusted sectoral weighting) to confirm the robustness of your main conclusion. Additionally, explicitly quantifying fossil fuel seasonality indices and their correlation with atmospheric CO2 seasonality would greatly strengthen your attribution.
3.Attribution method clarification: There is an inconsistency between methods (describing a vector projection approach) and the caption of Fig. 3 (implying contributions based on absolute trend magnitudes). Please clarify exactly how contributions are attributed and justify the chosen approach. If possible, showing both attribution methods would strengthen confidence in the robustness of conclusions.
4.Propagation of land flux uncertainty: You use multi-model means for terrestrial fluxes without explicitly propagating model spread into atmospheric CO2 metrics. Given significant inter-model differences in future terrestrial fluxes, providing uncertainty ranges for CO2 seasonal amplitude and phase metrics based on terrestrial flux ensemble spread would enhance the credibility of your analysis.
5.Historical validation enhancement: You acknowledge weaker historical performance in simulating autumn zero-crossing dates. It would be helpful to provide a more quantitative evaluation across the entire observational network (e.g., RMSE, correlation statistics) and explicitly discuss whether this bias originates from terrestrial flux uncertainty, ocean fluxes, or atmospheric transport.
Smaller issues:
•Title: Consider explicitly stating "Northern Hemisphere" to clearly define the geographic scope of the study.
•Lines 160–161: Briefly clarify in which regions ocean flux contributions might still be relevant (e.g., subpolar North Atlantic or North Pacific).
•Gini coefficient clarification (lines 326–331): Explain briefly that "11/12" is the theoretical maximum for 12 monthly bins to prevent confusion.
•Line 369: Replace "real revolutions" with "real evolution".
•Line 379: "CMIP5_FF" should be corrected to "CMIP6_FF".
•Lines 443–444: Verify that the Zenodo record number provided matches exactly with your submitted checklist.
•Figure 1 (lines 617–631): Clarify explicitly that trends are computed using 11-year running means followed by Theil–Sen slopes, with significance tested using Mann–Kendall tests. Also, explicitly state units: SCA (ppm), SZC and AZC (day of year).
•Figure 3 (lines 640–656): Clearly specify the attribution methodology used here and ensure it is consistent with the description provided in Methods. If trends are used in attribution, clarify briefly: "Underlying trends computed as in Fig. 1."
•Supplementary typo (Supplement lines 39–41): Correct "SSP53-7.0" to "SSP3-7.0".

(Remarks on code availability)

Version 1:

Reviewer comments:

Reviewer #2

(Remarks to the Author)
General Comments:
The authors have clearly put in a substantial amount of work for this revision, evidenced by the 88 sensitivity simulations and the robust cross-validation using SSA. The transition to time-varying meteorological fields is a major step forward that effectively addresses the previous concerns over transport versus flux forcing. However, from a physical and consistency standpoint, I still find several critical gaps that need to be resolved, particularly regarding the disconnect between the new evidence presented in the supplements and the core narrative in the main text.

1. While the authors revised the magnitude and seasonal profile of Chinese FF emissions in Supplementary Figures 17 and 18 to address previously noted biases (e.g., the Lunar New Year effect), it is not at all clear whether these corrections were integrated into the primary global results in the Main Text. If the core narrative and Figures 2 and 3 still rely on the original, biased CMIP6 inventory, the scientific integrity of the global attribution is compromised. Furthermore, for an assessment of CO2 concentration responses under net-zero pathways in China, the authors can refer to the recent study by Tan et al. (2025, Science China Earth Sciences, 10.1007/s11430-024-1502-y). This regional simulation provides an ideal simulation for how local source changes impact CO2 seasonality.
2. The study’s conclusion that FF emissions dominate future SCA trends hinges on the assumption of a "fixed fractional profile" for emissions through 2100. Although the Discussion briefly acknowledges the "profound decarbonization transition" under SSP1-2.6, the current analysis fails to address the massive uncertainty introduced by the structural change of the emission "shape." In a deep mitigation future, electrification (e.g., heat pumps replacing boilers) and the dominance of intermittent renewables will fundamentally reshape the seasonal phase and timing of residual FF use, rather than merely reducing its absolute magnitude. The authors must significantly deepen the discussion on this point: specifically, how might a shift in the peak-emission month or a change in the winter-to-summer emission ratio in key regions (like Eurasia) counteract the trend-driving effect of total magnitude reduction? Without a more rigorous exploration of this non-linear uncertainty, the claim that FF magnitude reduction is the sole driver remains overly idealized.
3. I noted that Supplementary Figure 17 is missing any citation in the main text. Additionally, given the lower performance in simulating the Autumn Zero-Crossing (AZC) trend compared to SCA (in the validation), the authors should use much more cautious language in the Abstract and Conclusion. Over-stating the confidence in autumn parameters is not advisable when the models themselves still struggle to capture the basic phenological timing of the carbon cycle during the senescence

phase.

(Remarks on code availability)

Reviewer #3

(Remarks to the Author)
Recommendation: Minor revision.

The manuscript has improved following this revision. The additional sensitivity analyses on fossil-fuel emission seasonality, time-varying meteorology, and uncertainty propagation from land-model differences provide stronger support for your primary conclusion: under strong mitigation scenarios, fossil-fuel emission patterns (both total emission reductions and smaller winter–summer contrasts) could become the main determinant of future Northern Hemisphere CO2 seasonal-cycle amplitude trends, rather than terrestrial fluxes.

Major points:

1. The revised manuscript clearly states that the ternary plots indicate relative importance based on absolute factorial trends (p.17, lines 439–449), which is appropriate. However, the use of absolute values means the direction (positive or negative contributions) is not explicitly visible. It would be helpful if the Fig. 3 caption or associated text clearly stated that the ternary plots reflect only the magnitude of each factor’s influence, while the direction (increase/decrease) can be found separately in the detailed maps (e.g., Supplementary Fig. 13).

2. The analysis employs an 11-year running mean before estimating trends and conducting Mann–Kendall significance tests (p.15, lines 378–381; Fig. 1 caption, p.27, lines 735–737). While this smoothing approach is valid, it introduces serial correlation that can affect the accuracy of statistical significance tests. Please briefly clarify whether autocorrelation was explicitly considered or corrected for, or confirm via a simple sensitivity check using unsmoothed annual data. A brief sentence on this would strengthen confidence in the statistical results.

3. The manuscript argues that opposing regional trends under SSP1-2.6 "compromise" BRW’s representativeness for the northern high-latitude domain (p.6, lines 109–114). Although the argument itself makes sense, "compromise" implies a stronger quantitative basis. It would be beneficial either to provide quantitative support (e.g., temporal correlation between BRW seasonal amplitude and the broader boreal average historically versus SSP1-2.6), or alternatively, slightly adjust wording to indicate "reduces" or "weakens" representativeness, tied explicitly to the spatial pattern shown.

4. The sensitivity test applying recent MEIC seasonal distributions (2017–2021) to future CMIP6 emissions totals (p.20, lines 510–516; Results p.8, lines 185–188) is useful. Still, readers may interpret this as a realistic future scenario. Please briefly clarify in the Methods that this sensitivity test is intended primarily as an uncertainty bounding exercise rather than a realistic prediction of future fossil-fuel emission patterns. Also, mention explicitly that the MEIC data used here refers to the global gridded dataset, as many readers associate MEIC specifically with Chinese inventories (p.18, lines 473–478; p.20, lines 510–513).

Minor editorial issues:

1. The description of future fossil-fuel emissions in CMIP6 scenarios appears twice (p.19, lines 498–501). Combine or delete one instance to remove redundancy.

2. Correct "will closely tracks" to "will closely track" (p.9, lines 198–199).

3. Add "the" to "In boreal region," making it "In the boreal region," (p.8, line 163).

(Remarks on code availability)
I did not attempt to run the code, as reproducing the full workflow likely requires substantial external datasets and computational resources beyond the scope of this review.

Version 2:

Reviewer comments:

Reviewer #2

(Remarks to the Author)
The authors have well addressed my concerns. While I still believe that incorporating the simulations driven by emissions with revised seasonality into the main text would be preferable, I accept the authors' reassurance that this adjustment does not alter their primary conclusions. Consequently, I recommend that the manuscript be accepted as is.

(Remarks on code availability)

Reviewer #3

(Remarks to the Author)
The authors have responded carefully to my previous comments, and I think the manuscript is now in good shape. In my view, the manuscript is suitable for publication after final editorial checks.

(Remarks on code availability)

Open Access This Peer Review File is licensed under a Creative Commons Attribution 4.0 International License, which permits use, sharing, adaptation, distribution and reproduction in any medium or format, as long as you give appropriate credit to the original author(s) and the source, provide a link to the Creative Commons license, and indicate if changes were made.
In cases where reviewers are anonymous, credit should be given to 'Anonymous Referee' and the source.
The images or other third party material in this Peer Review File are included in the article's Creative Commons license, unless indicated otherwise in a credit line to the material. If material is not included in the article's Creative Commons license and your intended use is not permitted by statutory regulation or exceeds the permitted use, you will need to obtain permission directly from the copyright holder.
To view a copy of this license, visit https://creativecommons.org/licenses/by/4.0/

EDITOR COMMENTS

As you will see from the reports copied below, the reviewers raise important concerns. We find that these concerns limit the strength of the study, and therefore we ask you to address them with additional work. Without substantial revisions, we will be unlikely to send the paper back to review.

In particular, it is essential to address the methodological limitations and uncertainties associated with the model simulations to ensure the reliability of the findings, with clear validations and explanations provided where appropriate. The issues related to the GEOS-Chem simulations, especially the incorrect SCA variations in China, should be carefully revisited and corrected. In addition, the narrative and interpretation of the results should be further refined to clarify the methodological advantages over previous studies and to strengthen the explanations of CO_2 seasonality and future projections, thereby enhancing the robustness and scientific rigor of the study.

Response:

We would like to thank the editor and reviewers for their insightful comments, which have been invaluable in strengthening our study. We have undertaken a substantial revision of the manuscript to address all the concerns raised. Please find below the point-to-point responses. The reviewers' comments are in black color, the responses are in blue. All the pages and line numbers indicated in this letter of response correspond to the revised version of text.

REVIEWER COMMENTS

Reviewer #1 (Remarks to the Author):

This work has not a prospect to get published in the journal for the reasons as indicated below.

1) Language and structure: The article contains errors and inconsistencies in sentence structure.

Response #1:

We thank the reviewer for raising the issues. We have thoroughly proofread the manuscript to correct grammatical errors, typos and inconsistencies in sentence structure, and had the text polished by native English-speaking co-authors. We hope these revisions have substantially improved the quality of the writing and have made our scientific arguments easier to follow.

2) Discussion of limitations: The article should have discussed the limitations of the research methodology in more detail and address potential biases in the results due to parameter choices.

Response #2:

We thank the reviewer for this valuable suggestion. We agree that a transparent discussion of limitations and potential biases is essential. In response, we have performed 72 additional transport simulations that systematically evaluate three primary sources of uncertainty:

1. Meteorological forcing: We assessed the impact of future atmospheric circulation changes using time-varying fields from two CMIP6 models;

2. Terrestrial flux uncertainty: We propagated the spread of terrestrial biosphere models by running simulations driven by four individual ESM fluxes;

3. Fossil fuel emission seasonality: We tested the sensitivity of our attribution to an alternative emission dataset with a different seasonality than the original CMIP6 one.

All sensitivity experiments confirm the robustness of our main conclusions. These analyses are now described in the Methods section and Supplementary Text 1. Full technical details are provided in Responses to Reviewer 2 (#2b, #2c, and #4).

3) The research methods adopted in this study should have been briefly introduced and clearly

stated the significance of this study.

Response #3:

We thank the reviewer for the suggestion. We have revised and enhanced the description of the Methods section, including detailed information on: 1) the historical atmospheric CO_2 observations; 2) the calculation of CO_2 seasonal cycle parameters; 3) the configuration of the GEOS-Chem transport simulations with sensitivity experiments; and 4) the CO_2 flux datasets used to drive the transport simulations.

We have also refined the Introduction and Discussion sections to explicitly state the significance of our findings. We highlight that understanding the future drivers of CO_2 seasonality, specifically the shift from biospheric to fossil fuel dominance under mitigation scenarios, is critical for accurately interpreting future atmospheric CO_2 monitoring data, and validating the effectiveness of emission reduction strategies.

4) Authors always have to write a paragraph saying: "The rest of the paper is organized as follows. Section 2 contains the literature review. Section 3 contains the methodology (method). Section 4 contains the results. Section 5 contains the conclusions and recommendations". So, the reader knows what's coming next.

Response #4:

We thank the reviewer for this suggestion. We agree with the reviewer about the importance of clearly setting expectations for the reader. However, we are constrained by the specific formatting guidelines of *Nature Communications*, which differ from the "Introduction, Literature review, Method, Results, Conclusions" structure found in many disciplines. Specifically, the journal requires the Introduction to flow directly into the Results, with a comprehensive Methods section placed at the end of the manuscript.

To address the intent of the reviewer's suggestion within these constraints, we have revised the final paragraph of the Introduction to more explicitly outline the logical progression of the investigation and the key questions addressed, serving as a conceptual guide for the reader before entering the Results section. The corresponding revisions are shown in Lines 63-73 as

follows:

"In this study, we investigate the evolution of the Northern Hemisphere (0°-90°N) atmospheric CO_2 seasonal cycle from the historical period (1979-2021) through 2100 under four CMIP6 scenarios (SSP1-2.6, SSP2-4.5, SSP3-7.0, and SSP5-8.5). We first validate our atmospheric transport simulations against historical records from 27 stations, with a detailed focus on two representative stations: Barrow, Alaska (BRW) and Mauna Loa, Hawaii (MLO). Subsequently, using a suite of factorial simulations, we isolate and quantify the distinct contributions of terrestrial ecosystems, fossil fuel emissions, and air-sea fluxes to projected changes in SCA, SZC, and AZC. Finally, we assess how the contributions of these drivers shift under different mitigation pathways, providing critical insights into the future utility of atmospheric CO_2 seasonality for monitoring the carbon cycle."

5) Even though authors can send unformat papers for evaluation, it is recommended to send the manuscript with the following format structure: (1) Abstract, (2) Introduction, (3) Experimental, (4) Results and discussion, (5) Conclusion, and (6) References. With a scientific format reviewers are able to evaluate the proposed paper with more accuracy and faster. Please, think about in your future attempt.

Response #5:

We thank the reviewer for this practical advice. As discussed in Response #4, we have strictly adhered to the formatting guidelines of *Nature Communications*, which mandates that the Methods section be placed at the end of the manuscript. Within this structure, we have revised the final paragraph of the Introduction to explicitly preview the study's logical flow for the reader. We appreciate the suggestion and will keep it in mind for future submissions to journals with more flexible formatting.

6) A concise and factual abstract and conclusion should have been provided

Response #6:

We thank the reviewer for this suggestion. The revised Abstract is as follows (Lines 18-32):

"Variations in the atmospheric CO_2 seasonal cycle across the Northern Hemisphere have

historically been dominated by terrestrial ecosystems, making ground-based observations a reliable proxy for terrestrial carbon dynamics. However, whether this dominance will persist under future scenarios remains uncertain. Here, we combine atmospheric transport modeling with factorial simulations to assess and attribute future changes in the CO_2 seasonal cycle through 2100. We find that projected changes vary substantially across scenarios, with divergent underlying drivers. Under the high-emission scenario (SSP5-8.5), increasing land sinks continue to govern and amplify CO_2 seasonal variability. In contrast, under the low-emission scenario (SSP1-2.6), a widespread decline in CO_2 seasonal amplitude is predominantly driven by fossil fuel emissions rather than land sinks. These findings indicate that established CO_2 observational frameworks might become unreliable for monitoring variations in the terrestrial carbon cycle under mitigation pathways. Instead, they might become more indicative of fossil fuel emissions, underscoring the urgent need for new interpretative approaches to support sectoral climate policy verification."

We have also revised the concluding paragraph in the Discussion section to summarize the study's implications in Lines 320-333:

"In summary, our projections reveal that the atmospheric CO_2 seasonal cycle is not a static proxy for the terrestrial ecosystems but a dynamic signal that can be substantially influenced by anthropogenic emissions. Crucially, under strong mitigation scenario like SSP1-2.6, the fossil fuel emissions become the primary driver of SCA declines. This shift might complicate the monitoring of terrestrial ecosystem carbon dynamics using atmospheric signals in the future. Addressing this challenge requires integrating observational and modeling approaches, including the expansion of eddy covariance tower networks for direct high-frequency measurements of ecosystem carbon flux[47,48], improvement in DGVMs to better capture seasonal dynamics and carbon-climate interactions[49,50], and the use of advanced atmospheric inversions that integrate CO_2 concentration measurements with process-based estimates of terrestrial carbon flux[51-53]. Sustaining rigorous monitoring of both terrestrial carbon cycle dynamics and fossil fuel emissions is essential for verifying progress toward emission reduction targets required to constrain global warming."

7) Author should have compared the results with literature. As it is not possible, tried to

highlight the importance and innovation of the study.

Response #7:

We thank the reviewer for this suggestion. Previous studies have primarily focused on changes in the atmospheric CO_2 seasonal cycle during the historical period (e.g. Graven et al., 2013; Forkel et al., 2016; Piao et al., 2018). Our analysis of the historical period corroborates these findings, confirming that terrestrial ecosystems have been dominating the SCA increase and the advance of SZC. Regarding future projections, limited studies have explored the SCA trends under high-emission scenarios (Zhao and Zeng, 2014; Liu et al., 2024). Our results under SSP5-8.5 are consistent with these studies, confirming a continued amplification of SCA driven by enhanced terrestrial carbon uptake via sustained CO_2 fertilization.

Beyond confirming existing trends, our study addresses a critical gap by providing the first systematic assessment of CO_2 seasonal variations under strong mitigation scenario (e.g. SSP1-2.6). Unlike high-emission pathways, we identify a widespread decline in SCA across the Northern Hemisphere under SSP1-2.6, driven primarily by fossil fuel carbon emissions rather than terrestrial ecosystem processes. This finding suggests that the atmospheric CO_2 seasonal cycle is not a static proxy for the terrestrial ecosystems but a dynamic signal sensitive to emission scenarios. Consequently, we advocate for new interpretive frameworks to analyze atmospheric signals to verify climate policies.

We have summarized the previous studies in the Introduction section and highlighted this study's innovation and its implications in the revised Abstract and concluding paragraph (as detailed in Response #6).

Reference

Forkel, M. *et al.* Enhanced seasonal CO_2 exchange caused by amplified plant productivity in northern ecosystems. *Science* **351**, 696-699 (2016).

Graven, H. D. *et al.* Enhanced seasonal exchange of CO_2 by northern ecosystems since 1960. *Science* **341**, 1085-1089 (2013).

Liu, Z. *et al.* Seasonal CO_2 amplitude in northern high latitudes. *Nat. Rev. Earth Environ.* **5**, 802-817 (2024).

Piao, S. *et al.* On the causes of trends in the seasonal amplitude of atmospheric CO_2. *Glob. Change Biol.* **24**, 608-616 (2018).

Zhao, F. & Zeng, N. Continued increase in atmospheric CO_2 seasonal amplitude in the 21st century projected by the CMIP5 Earth system models. *Earth Syst. Dynam.* **5**, 423-439 (2014).

Reviewer #2 (Remarks to the Author):

This manuscript assessed and attributed potential changes in the CO_2 seasonal cycle under four future scenarios through 2100, based on the atmospheric transport modeling combined with factorial simulations. And they found that under the low-emission scenario (SSP1-2.6), the decline in SCA is predominantly driven by fossil fuel emissions rather than land sinks. Overall, the written is well organized, However, for a journal like NC, the reliability and significance of this work are still open to question.

Response:

We thank the reviewer for their thorough and thoughtful review of our manuscript.

1. Reliability: To rigorously address the concern about model uncertainty, we have performed extensive new analyses involving 72 additional future transport simulations (Response Table 1), a substantial expansion compared to our original suite of 16 simulations. These sensitivity experiments systematically tested the impacts of meteorological uncertainty, terrestrial ecosystem CO_2 flux uncertainty, and fossil fuel CO_2 emission seasonality on the attribution of CO_2 seasonal cycle. All these sensitivity tests confirm the robustness of our primary finding: under mitigation scenario (e.g. SSP1-2.6), the decline in the SCA is predominantly driven by fossil fuel emissions rather than terrestrial ecosystem.
2. Significance: These robust results highlight a critical gap in current carbon monitoring frameworks. We demonstrate that under future mitigation policies, the atmospheric CO_2 seasonal cycle will shift from a proxy for terrestrial ecosystems to a signal indicative of fossil fuel emissions. This finding is significant because it challenges established monitoring strategies and underscores the need for new verification methods to support climate policies.

Below, we provide detailed point-by-point responses to your specific concerns.

Response Table 1. Sensitivity experiments of future transport simulations under different scenarios driven by alternative meteorological fields, terrestrial CO_2 fluxes, and fossil fuel CO_2 emissions.

Experiment Type	Scenarios	Key inputs	Simulation Configuration	Total number of additional simulations

Meteorological uncertainty	SSP1-2.6, SSP2-4.5, SSP3-7.0, SSP5-8.5	Meteorological fields from 2 ESMs: CanESM5, NorESM2-LM	4 factorial simulations (CMIP6_Base, CMIP6_Land, CMIP6_FF, CMIP6_Ocean)	32 (4×2×4)
Terrestrial CO_2 flux uncertainty	SSP1-2.6, SSP2-4.5, SSP3-7.0, SSP5-8.5	Terrestrial CO_2 fluxes from 4 ESMs: ACCESS-ESM1-5, CanESM5, NorESM2-LM, MPI-ESM1-2-LR	2 factorial simulations (CMIP6_Base, CMIP6_Land)	32 (4×4×2)
Fossil fuel emission uncertainty	SSP1-2.6, SSP2-4.5, SSP3-7.0, SSP5-8.5	Fossil fuel emissions scaled by MEIC seasonal cycle	2 factorial simulations (CMIP6_Base, CMIP6_FF)	8 (4×2)

Major comments:

(1) You detrended the CO_2 time series by a curve fitting method. Here I wonder whether different detrend methods can induce uncertainties when you calculating the date of SZC and AZC.

Response #1:

We thank the reviewer for this insightful comment. To address this issue, we additionally applied the singular spectrum analysis (SSA) method (Vautard et al., 1992), a non-parametric time-series decomposition approach, to extract the CO_2 seasonal cycle by retaining signal components with periodicities between 180 and 420 days (Liu et al., 2025).

We compared the SCA, SZC, and AZC metrics derived from CCGCRV and SSA methods for historical and future scenarios at the two representative stations, BRW and MLO. The SCA is highly consistent between the two methods, characterized by strong correlations at both stations. While there are minor offsets in the absolute SZC and AZC dates, the long-term trends, which are the primary focus of this study, remain broadly consistent across both historical and future scenarios (Supplementary Figs. 5, 6). These comparisons confirm that our conclusions regarding the evolution of the CO_2 seasonal cycle are robust and not artifacts of specific curve-fitting methodology.

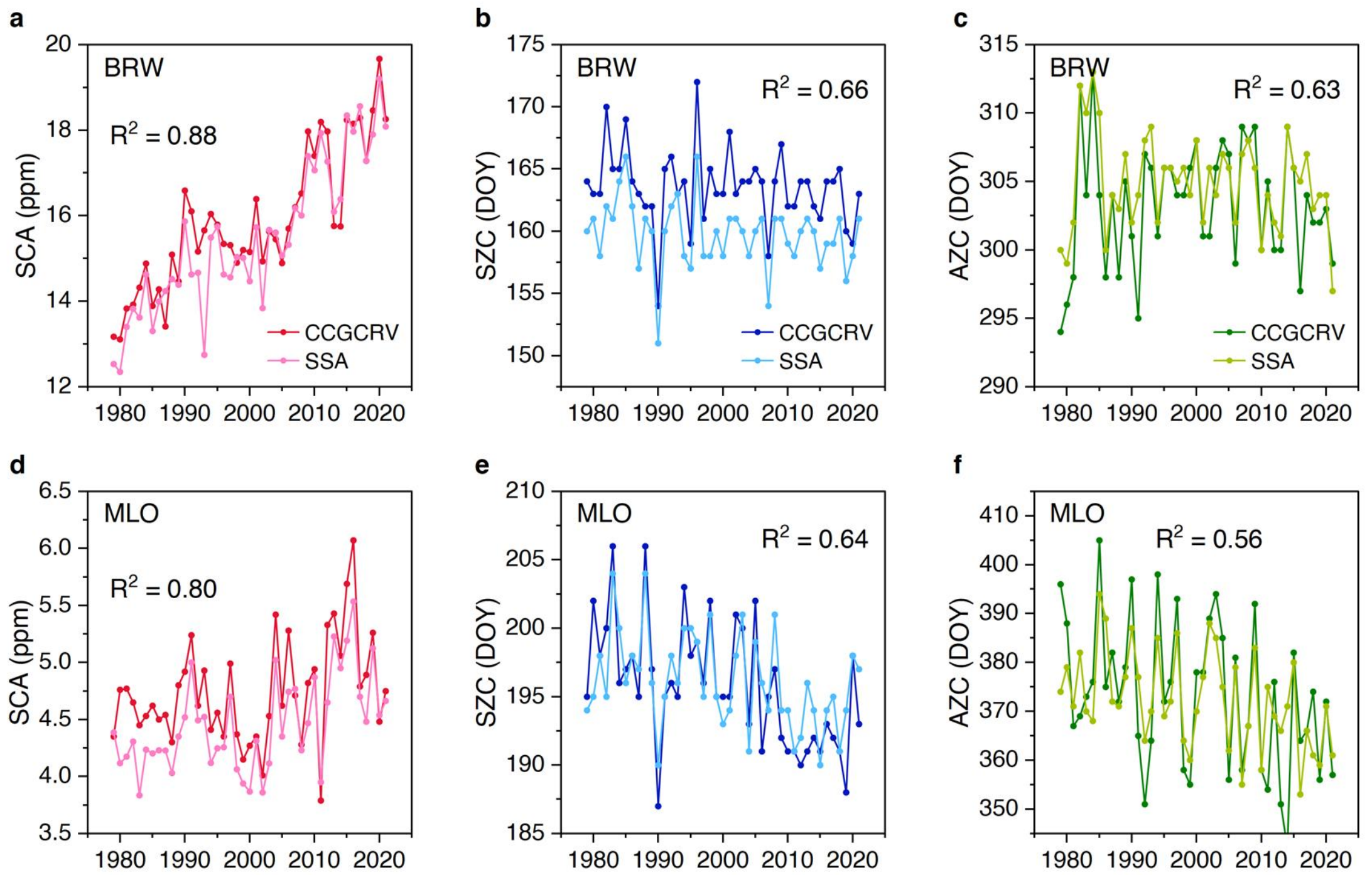

Supplementary Figure 5. Key metrics of CO_2 seasonal cycle derived using CCGCRV and SSA at the BRW and MLO stations during 1979-2021. Shown are comparisons of SCA (**a, d**), SZC (**b, e**), and AZC (**c, f)** from the historical baseline simulation (HIS_Base) at BRW (top row) and MLO (bottom row).

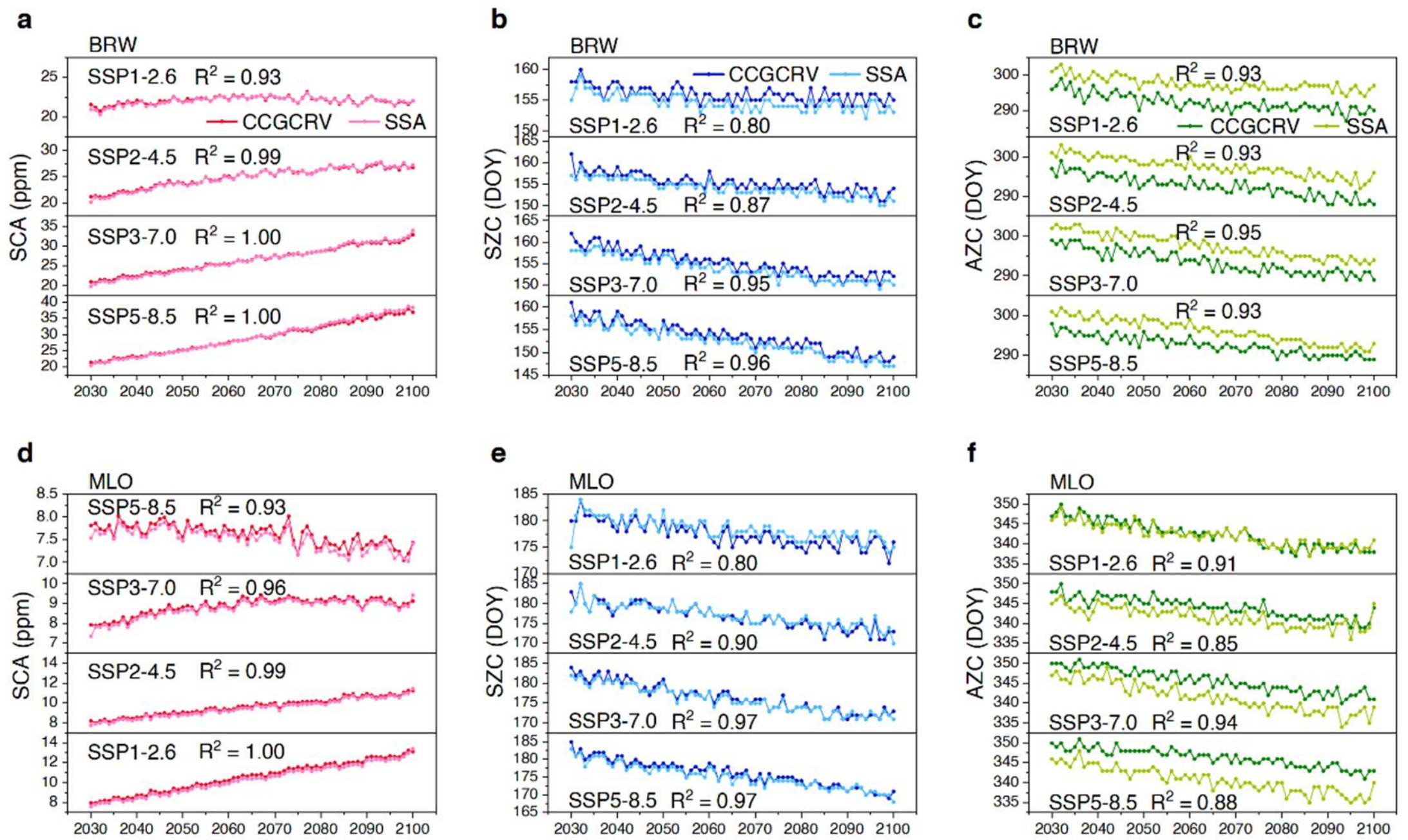

Supplementary Figure 6. Key metrics of CO_2 seasonal cycle derived using CCGCRV and SSA at the BRW and MLO stations under different scenarios for 2030-2100. Shown are comparisons of SCA (**a, d**), SZC (**b, e**), and AZC (**c, f)** from the CMIP6 baseline simulations (CMIP6_Base)

at BRW (top row) and MLO (bottom row).

We have added the following content to the Methods section in Lines 362-369.

"To evaluate the sensitivity of our results to the choice of detrending method, we also applied the singular spectrum analysis (SSA)[58] to decompose the CO_2 time series into multiple orthogonal components and to reconstruct the seasonal cycle by retaining variability within the 180–420 day frequency band. Comparisons between SSA and CCGCRV confirm that the derived trends in seasonal metrics are robust to the extraction methodology (Supplementary Figs. 5, 6) at the two representative stations, BRW and MLO. Given this consistency, we used the CCGCRV method for the primary analysis throughout the manuscript."

Reference

Vautard, R., Yiou, P. & Ghil, M. Singular-spectrum analysis: A toolkit for short, noisy chaotic signals. *Physica D: Nonlinear Phenomena* **58**, 95-126 (1992).

Liu, G. et al. Trends in the seasonal amplitude of atmospheric methane. *Nature* **641**, 660-665 (2025).

(2) You used the GEOS-Chem to simulate the global 3-D atmospheric CO_2. However,

- (a) you did not mention how to initialize the 3-D CO_2 when you ran the model starting from 1979 and 2030.

Response #2a:

We thank the reviewer for pointing out this omission. For the historical simulations from 1979 to 2021, we performed a two-year spin-up to ensure the initial 3-D CO_2 field was consistent with the GEOS-Chem transport model. We first estimated an initial CO_2 field for 1 January 1977 by taking the 1979 CAMS inversion (https://ads.atmosphere.copernicus.eu/datasets/cams-global-greenhouse-gas-inversion?tab=overview) data and subtracting the observed global CO_2 growth rates from NOAA GML for 1977-1978 for all model grid cells. Then, we ran GEOS-Chem from 1977 to 1978 using recycled 1979 CAMS fluxes scaled to match the global total from the Global Carbon Budget 2022 (Friedlingstein et al., 2022). The resulting 3-D CO_2 field on 1 January 1979 served

as our final, dynamically consistent restart file, minimizing any potential bias from using an initial field generated by a different transport model in CAMS.

Since CMIP6 scenarios do not provide gridded CO_2 concentration fields, we generated a consistent initial field for 1 January 2030 for all scenarios. We extended from the end of our historical simulation (31 December 2021) to 1 January 2030, where the model was forced with a climatology of monthly fluxes averaged over 2012-2021. Using this identical starting point for all scenarios (SSP1-2.6, SSP2-4.5, SSP3-7.0, and SSP5-8.5) ensures that any divergence in CO_2 concentrations and seasonal cycle metrics after 2030 is attributable solely to the prescribed CMIP6 SSP emission pathways.

We have added the following details to the Methods section (Lines 392-402 and 411-416):

"For the historical simulations, we designed one base transport experiment (HIS_Base) and three factorial transport experiments (HIS_Land, HIS_FF, and HIS_Ocean), all spanning 1979-2021 and initialized from a common 3-D CO_2 field for 1 January 1979. This initial field was generated via a two-year spin-up simulation. Specifically, we first estimated an initial CO_2 field for 1 January 1977 by taking the 1979 CAMS inversion (https://ads.atmosphere.copernicus.eu/datasets/cams-global-greenhouse-gas-inversion?tab=overview) data and subtracting the observed 1977-1978 global CO_2 growth rates from NOAA GML from all model grid cells. We then ran GEOS-Chem for the years 1977-1978, using recycled 1979 CAMS fluxes that were scaled globally to match the Global Carbon Budget 2022[55]. The resulting 3-D CO_2 field on 1 January 1979 served as our final, dynamically consistent restart file."

"Under each scenario, we performed four transport experiments, namely CMIP6_Base, CMIP6_Land, CMIP6_FF, and CMIP6_Ocean, all initialized with a common CO_2 field for 1 January 2030. This initial field was generated by running the model from the end of our historical simulation (31 December 2021) to 1 January 2030, driven by an annually repeating cycle of mean fluxes derived from the 2012-2021 historical period."

Reference

Friedlingstein, P. et al. Global Carbon Budget 2022. *Earth Syst. Sci. Data* **14**, 4811-4900 (2022).

- (b) Importantly, after reading the entire manuscript, I noticed that the future simulations were conducted using constant meteorological fields from 2021. This assumption seems unreasonable, as atmospheric circulation patterns are expected to differ across scenarios. Would it be possible to force the transport model with scenario-specific climate data instead?

Response #2b:

We thank the reviewer for the insightful comment. To address the impact of changing meteorology and atmospheric circulation patterns, we conducted a comprehensive sensitivity analysis involving 32 additional transport simulations driven by time-varying CMIP6 meteorological fields. First, we performed a systematic sensitivity analysis to identify which meteorological variables influence CO_2 transport in GEOS-Chem. We substituted individual MERRA-2 fields one at a time with their CMIP6 counterparts and evaluated the resulting change in simulated CO_2 concentrations. We found that transport is governed by five key CMIP6 variables, namely surface pressure (ps), planetary boundary layer height (bldep), eastward wind (ua), northward wind (va), and specific humidity (hus). Other variables, such as temperature and vertical pressure velocity, had negligible effects on simulated CO_2 fields. Based on this finding, our subsequent simulations driven by time-varying meteorological data will only consider the five identified key variables from the CMIP6 models.

Due to computational constraints, we chose 2 representative CMIP6 models, CanESM5 and NorESM2-LM, that provided the complete set of the five key meteorological fields in all scenarios (Supplementary Table 4). For each scenario, we ran the baseline and three factorial experiments using time-varying meteorological data from both models. This resulted in a total of 32 simulations (2 meteorological datasets × 4 scenarios × 4 experiments).

As the meteorological data provided by CMIP6 have much coarser temporal and vertical resolution than GEOS-Chem (Supplementary Table 5), we used the daily or monthly CMIP6 fields and performed piecewise linear interpolation to map them from the eight fixed pressure levels provided by CMIP6 onto the 72-layer GEOS-Chem hybrid sigma-pressure grid. For layers below 1000 hPa or above 10 hPa, values from 1000 hPa or 10 hPa were used.

Supplementary Table 4. Availability of meteorological variables from the ten EMSs used in

this study under the SSP1-2.6, SSP2-4.5, SSP3-7.0, and SSP5-8.5 scenarios, where √ indicates the variable is provided and ✕ indicates it is not provided.

	ACCESS-ESM1-5	CanESM5	CanESM5-1	CESM2-WACCM	CMCC-ESM2	MPI-ESM1-2-LR	NorESM2-LM	NorESM2-MM	INM-CM4.8	INM-CM5.0
ps	√	√	√	√	√	√	√	√	√	√
bldep	✕	√	✕	✕	✕	✕	√	√	√	√
ua	√	√	√	√	√	√	√	√	√	√
va	√	√	√	√	√	√	√	√	√	√
hus	√	√	√	√	√	√	√	√	√	√

Supplementary Table 5. Comparison of meteorological variables between MERRA-2 and CMIP6.

2-dimensional variable							
Variables in MERRA-2				Variables in CMIP6			
Abbreviation (standard name)	Unit	Frequency	-	Abbreviation (standard name)	Unit	Frequency	-
PS (surface_pressure)	Pa	3-hourly	-	ps (surface_air_pressure)	Pa	monthly	-
PBLH (planetary_boundary_layer_height)	m	hourly	-	bldep (atmosphere_boundary_layer_thickness)	m	monthly	-
3-dimensional variable							
Variables in MERRA-2				Variables in CMIP6			
Abbreviation (standard name)	Unit	Frequency	Vertical grid	Abbreviation (standard name)	Unit	Frequency	Vertical grid
U (eastward_wind)	$m\ s^{-1}$	3-hourly	hybrid sigma-pressure grid with 72 layers	ua (eastward_wind)	$m\ s^{-1}$	daily	8 layers at 1000, 850, 700, 500, 250, 100, 50, and 10 hPa
V (northward_wind)	$m\ s^{-1}$	3-hourly	hybrid sigma-pressure grid with 72 layers	va (northward_wind)	$m\ s^{-1}$	daily	8 layers at 1000, 850, 700, 500, 250, 100, 50, and 10 hPa

QV (specific_humidity)	kg kg^{-1}	3-hourly	hybrid sigma-pressure grid with 72 layers	hus (specific_humidity)	kg kg^{-1}	daily	8 layers at 1000, 850, 700, 500, 250, 100, 50, and 10 hPa

(1) Projections of trends in CO_2 seasonal parameters under simulations driven by different meteorological data

The projected trends in SCA, SZC and AZC are insensitive to the choice of meteorological forcing. The results driven by time-varying CMIP6 meteorology are largely consistent with the original simulations using constant 2021 fields (Supplementary Fig. 33), as exemplified at BRW and MLO under all future scenarios, with the exception of the AZC trend at MLO, which shows some sensitivity to meteorological forcing but does not alter the attribution conclusions. Likewise, the magnitudes and spatial patterns of SCA, SZC, and AZC trends also show strong coherence across simulations driven by different meteorological datasets in all scenarios (Supplementary Figs. 34-36). Although some differences exist, they are confined to small regions along the western Atlantic and Pacific margins in the northern tropics under SSP2-4.5 and SSP3-7.0 scenarios. The insensitivity of CO_2 seasonal metrics to atmospheric transport was also found in previous studies focusing on historical periods (Graven et al., 2013; Forkel et al., 2016; Piao et al., 2018), and our results confirm that this phenomenon will likely persist in the future.

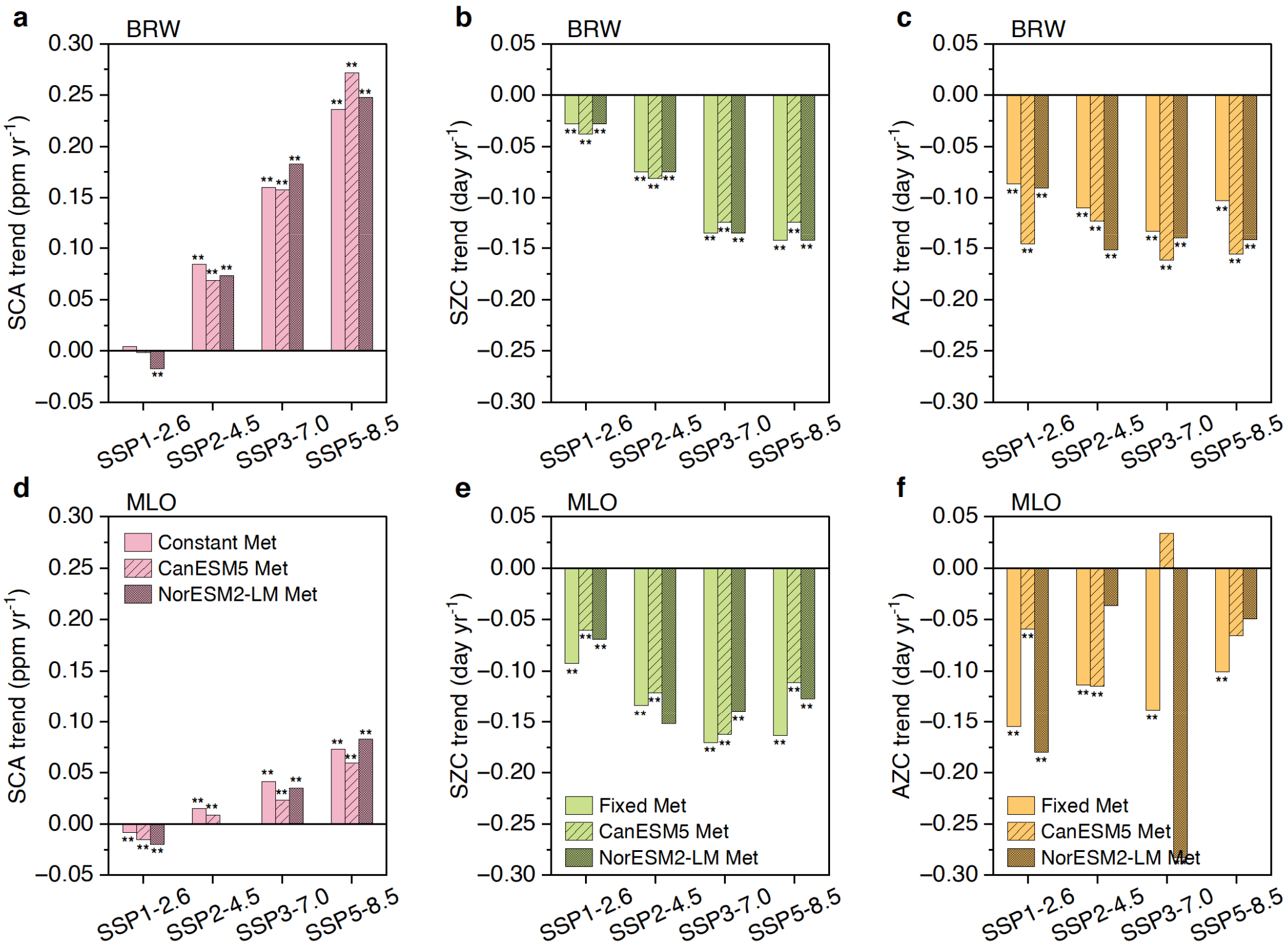

Supplementary Figure 33. Variations in the seasonality of atmospheric CO_2 under four future scenarios at the Barrow, Alaska (BRW; **a-c**), and Mauna Loa, Hawaii (MLO; **d-f**) stations. "Constant Met", "CanESM5 Met", and "NorESM2-LM Met" denote trends in CO_2 seasonal parameters simulated using constant meteorological fields in 2021 from MERRA-2, time-varying meteorological fields from CanESM5, and time-varying meteorological fields from NorESM2-LM, respectively. The panels display the trends in SCA (left column), SZC (middle column), and AZC (right column). Statistically significant trends ($p < 0.05$) are marked by two dots above the bars, and marginally significant trends ($p < 0.1$) by one dot.

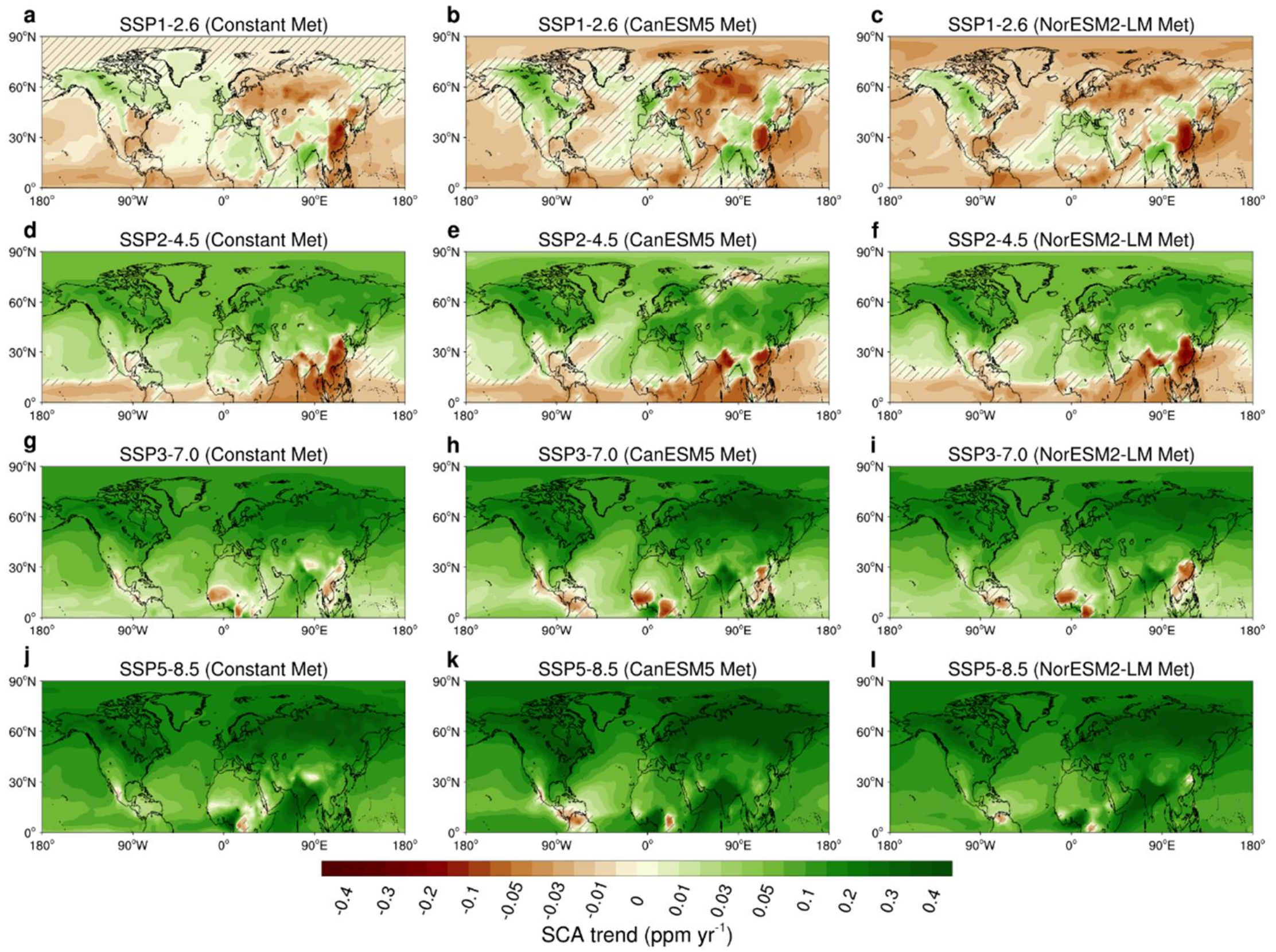

Supplementary Figure 34. Spatial patterns of simulated SCA trends across the Northern Hemisphere from 2030 to 2100 under different future scenarios, driven by different meteorological fields. Panels show SCA trends under SSP1-2.6 (**a-c**), SSP2-4.5 (**d-f**), SSP3-7.0 (**g-i**), and SSP5-8.5 (**j-l**), driven by fixed 2021 meteorological fields from MERRA-2 (left column), time-varying meteorological fields from CanESM5 (middle column), and time-varying meteorological fields from NorESM2-LM (right column). Hatched areas denote regions where trends are not statistically significant ($p > 0.05$).

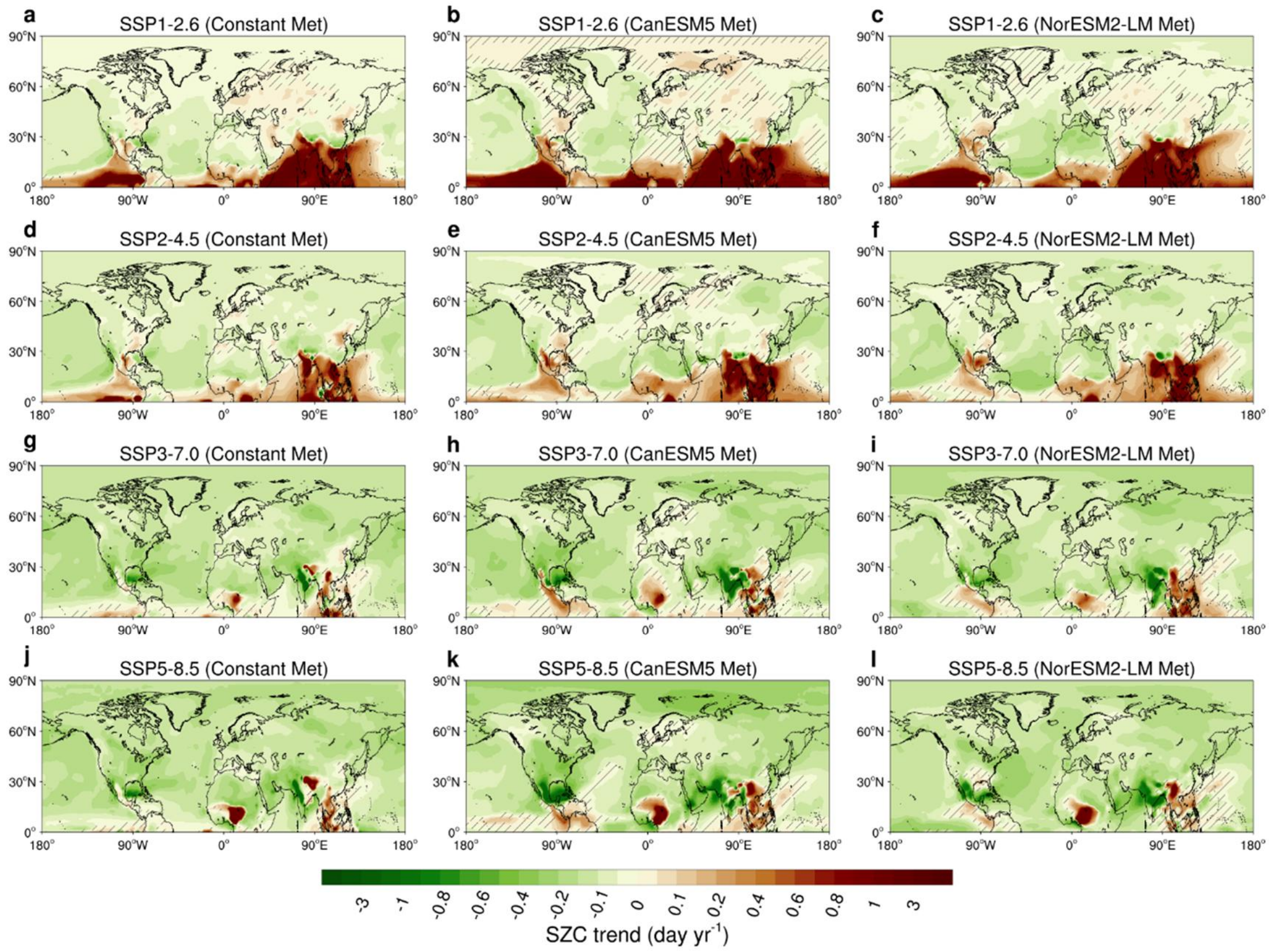

Supplementary Figure 35. Same as Supplementary Fig. 34, but showing the SZC trend.

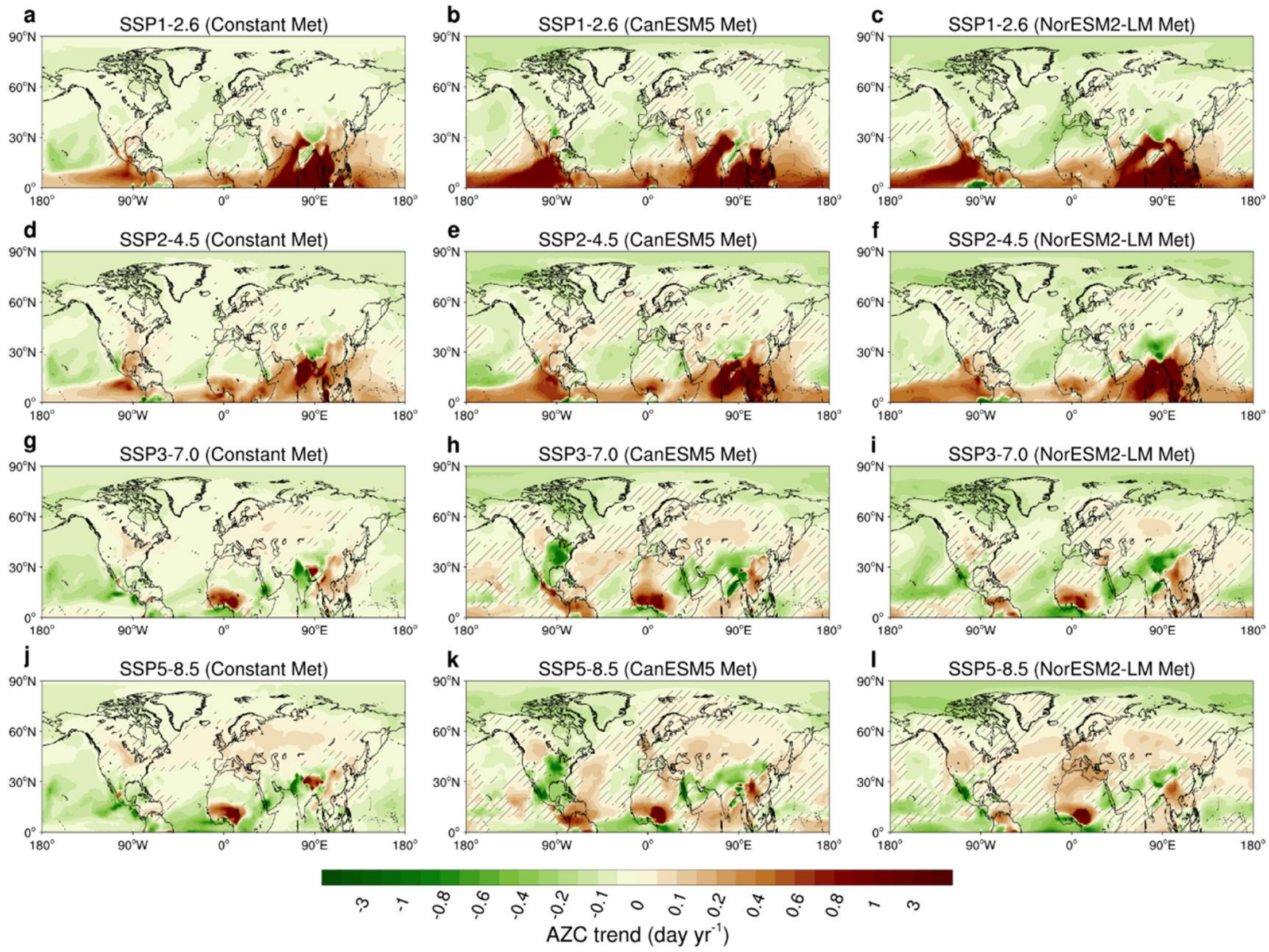

Supplementary Figure 36. Same as Supplementary Fig. 34, but showing the AZC trend.

(2) Attribution of projected trends in CO_2 seasonal parameters under simulations driven by different meteorological data

Using meteorological fields from CanESM5 and NorESM2-LM, the spatial patterns of the relative contributions of net terrestrial CO_2 fluxes, fossil fuel CO_2 emissions, and air-sea CO_2 exchange to trends in atmospheric CO_2 seasonal metrics across the Northern Hemisphere closely resemble those derived from simulations driven by the constant 2021 MERRA-2 fields (Fig. 3, Supplementary Figs. 37, 38). As a result, the attribution of these trends remains unchanged (Supplementary Figs. 39-44). Under the SSP1-2.6 scenario, fossil fuel emissions remain the dominant driver of the SCA decline, and their contribution to shifts in SZC and AZC is markedly greater than during the historical period. In contrast, under the SSP5-8.5 scenario, terrestrial ecosystems remain the primary driver of changes in CO_2 seasonal parameters.

These extensive sensitivity tests confirm that future circulation changes exert a secondary effect compared to the magnitude of flux changes. Consequently, our original approach using constant meteorology is justified, and our main conclusions regarding the drivers of the CO_2 seasonal cycle are robust.

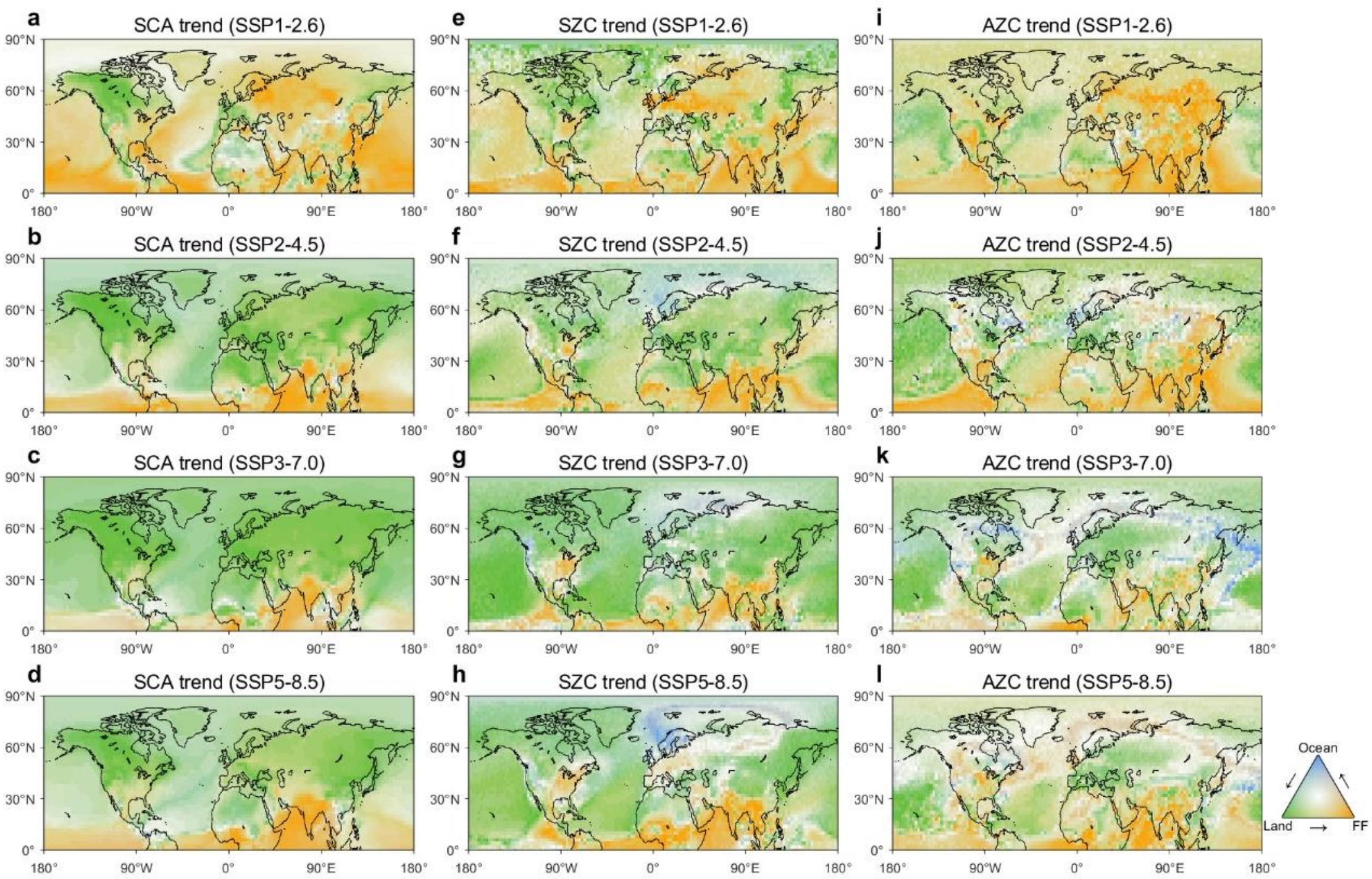

Supplementary Figure 37. Driven by time-varying meteorological fields from CanESM5, the relative proportions of net terrestrial CO_2 fluxes, fossil fuel CO_2 emissions, and air-sea CO_2

exchange contributing to trends in atmospheric CO_2 seasonal parameters across the Northern Hemisphere from 2030 to 2100 are shown under different future scenarios. Panels display the relative proportions of the three component fluxes associated with trends in SCA (**a-d**), SZC (**e-h**), and AZC (**i-l**) for the SSP1-2.6 (top row), SSP2-4.5 (second row), SSP3-7.0 (third row), and SSP5-8.5 (bottom row) scenarios.

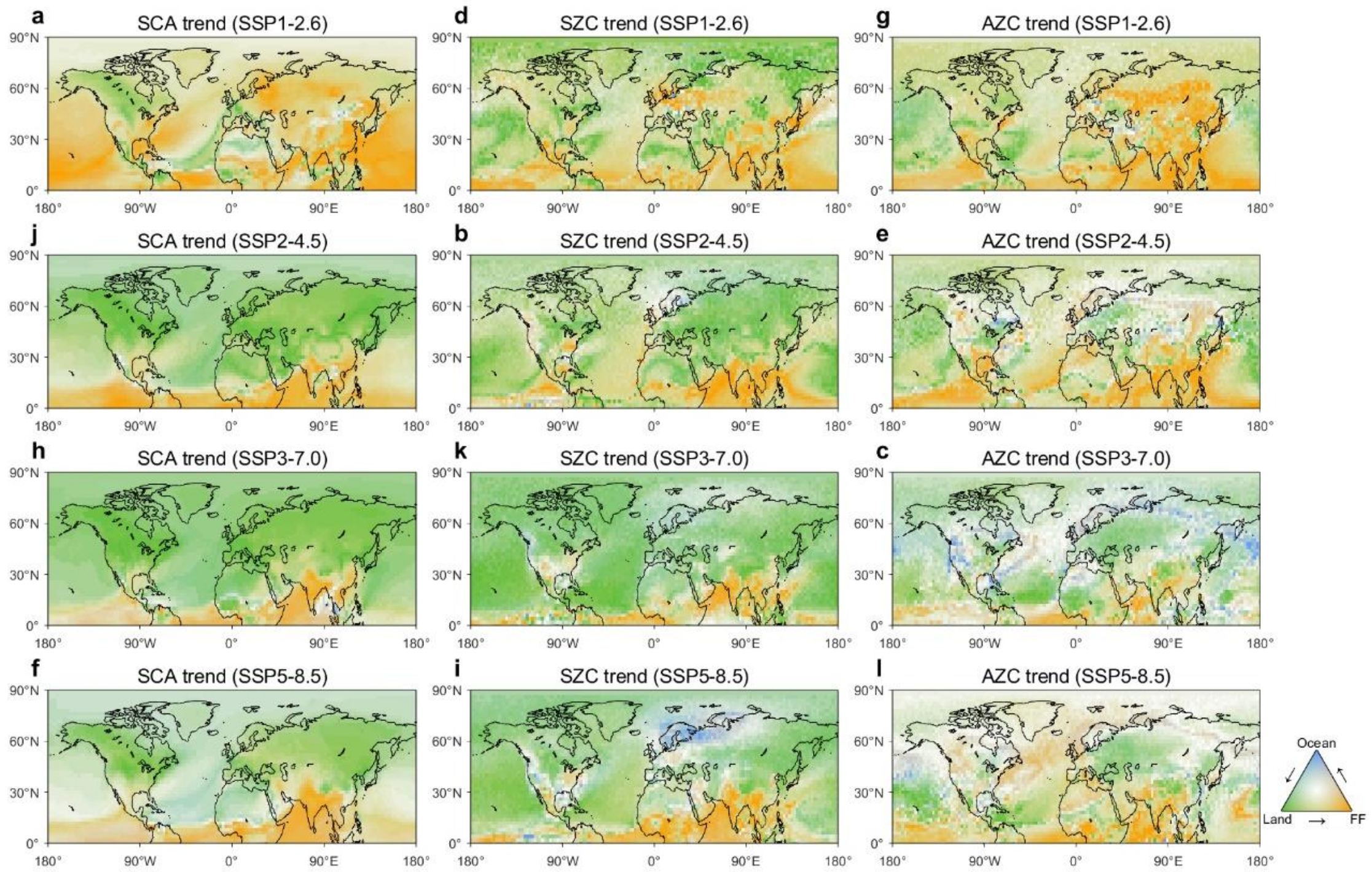

Supplementary Figure 38. Same as Supplementary Fig. 37, but showing the relative proportions of the three CO_2 fluxes from simulations driven by meteorological fields from NorESM2-LM.

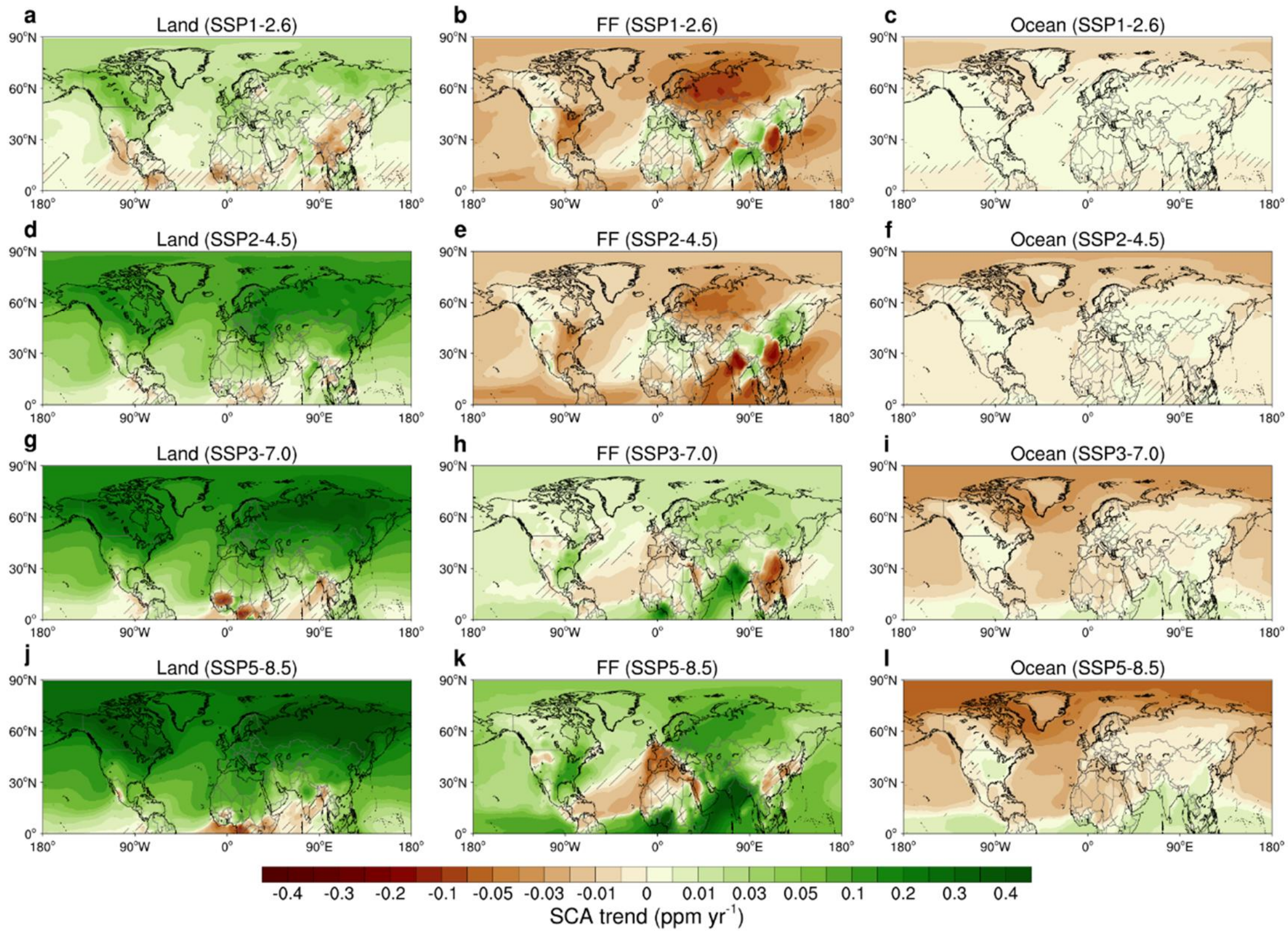

Supplementary Figure 39. Driven by time-varying meteorological fields from CanESM5, the spatial patterns of simulated SCA trends from 2030 to 2100 are attributed to variations in the terrestrial ecosystem (left panels), fossil fuel CO_2 emissions (middle panels), and air-sea CO_2 exchange (right panels). Results are shown for the SSP1-2.6 (**a-c**), SSP2-4.5 (**d-f**), SSP3-7.0 (**g-i**), and SSP5-8.5 (**j-l**) scenarios, estimated from the differences between the base simulations and the corresponding factorial simulations. The hatched areas indicate that the trends in these regions did not pass the significance test ($p > 0.05$).

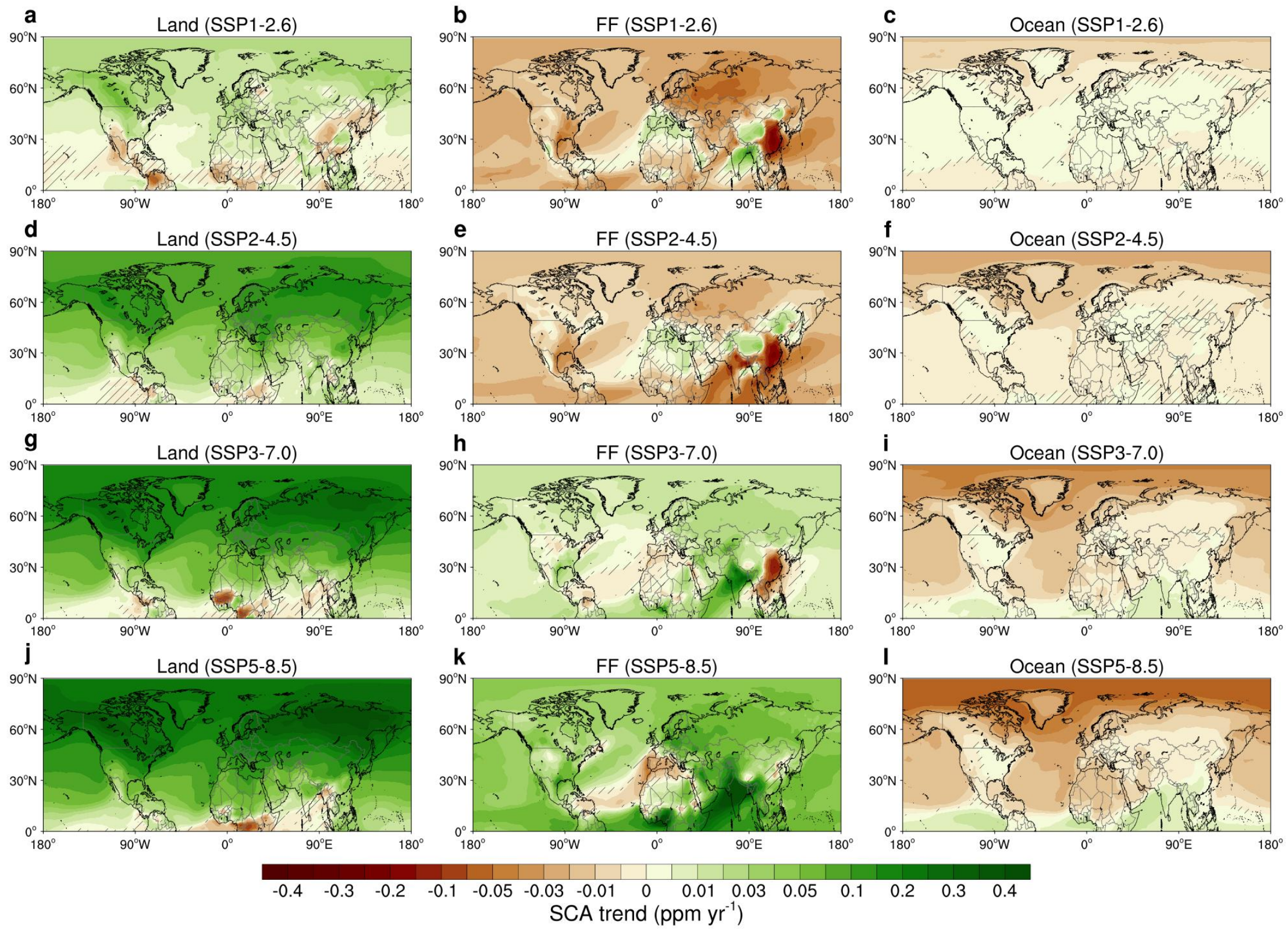

Supplementary Figure 40. Same as Supplementary Fig. 39, but showing the attribution of the SCA trend under simulations driven by meteorological fields from NorESM2-LM.

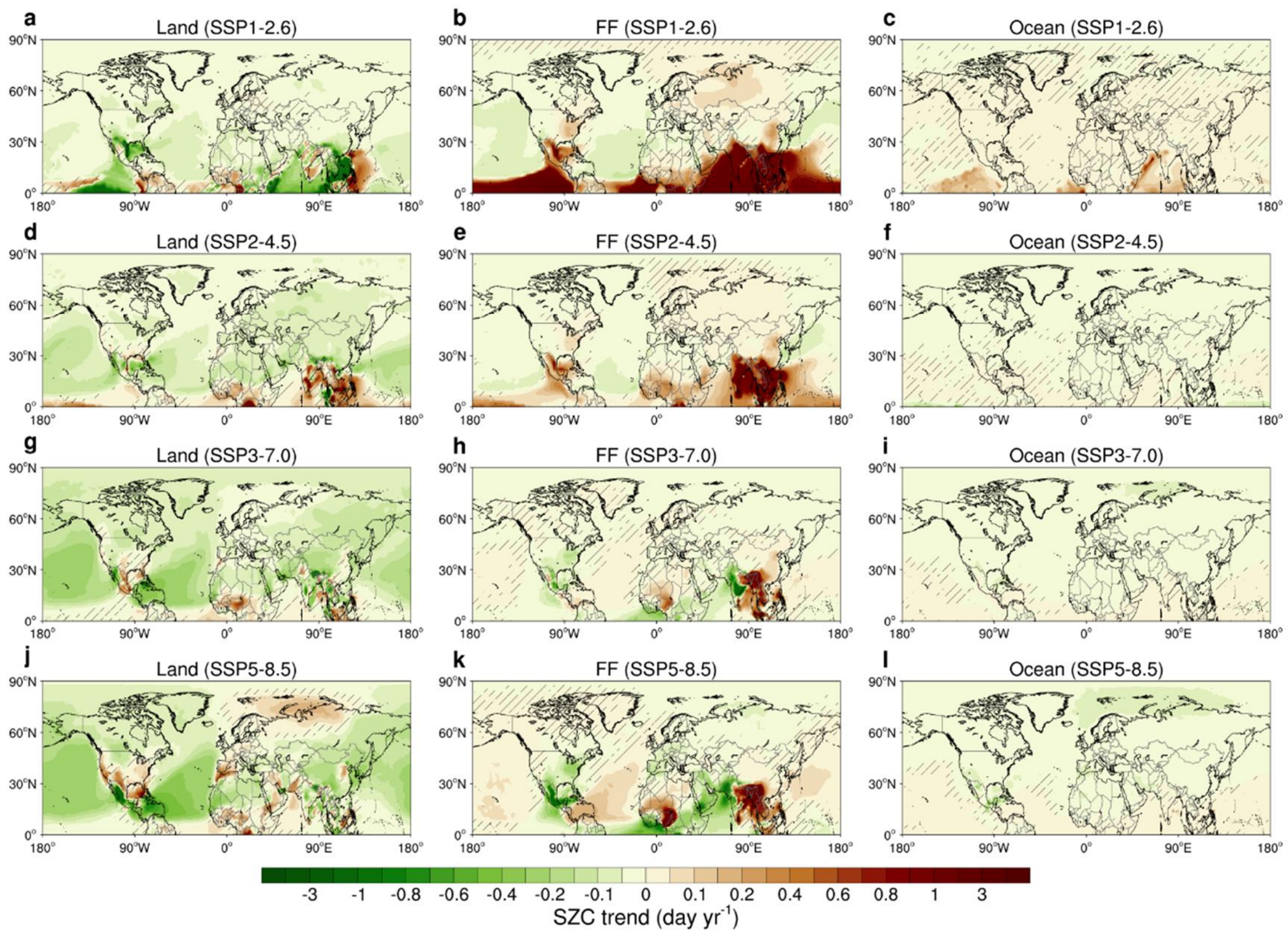

Supplementary Figure 41. Driven by time-varying meteorological fields from CanESM5, the

spatial patterns of simulated SZC trends from 2030 to 2100 are attributed to variations in the terrestrial ecosystem (left panels), fossil fuel CO_2 emissions (middle panels), and air-sea CO_2 exchange (right panels). Results are shown for the SSP1-2.6 (**a-c**), SSP2-4.5 (**d-f**), SSP3-7.0 (**g-i**), and SSP5-8.5 (**j-l**) scenarios, estimated from the differences between the base simulations and the corresponding factorial simulations. The hatched areas indicate that the trends in these regions did not pass the significance test ($p > 0.05$).

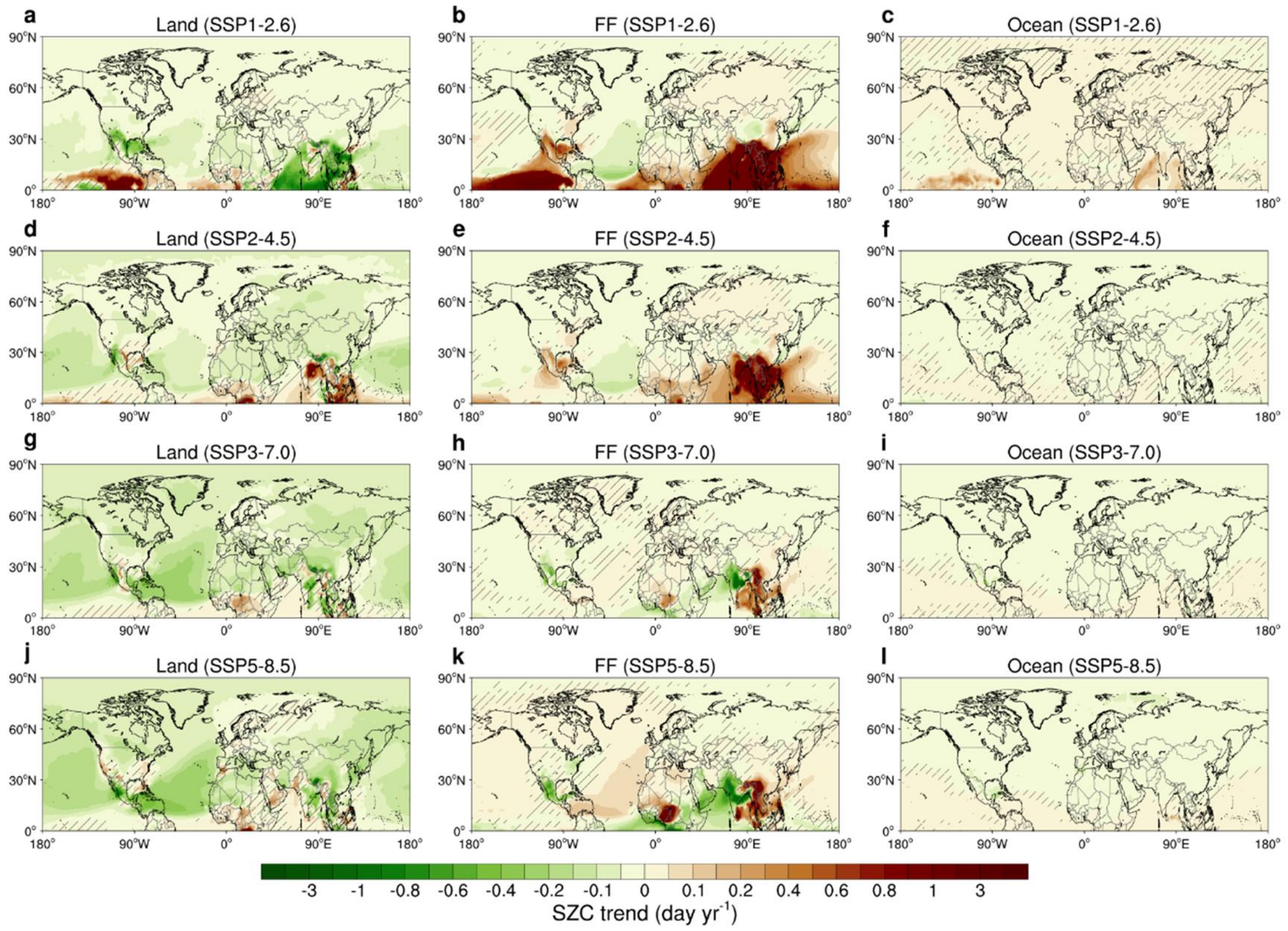

Supplementary Figure 42. Same as Supplementary Fig. 41, but showing the attribution of the SZC trend under simulations driven by meteorological fields from NorESM2-LM.

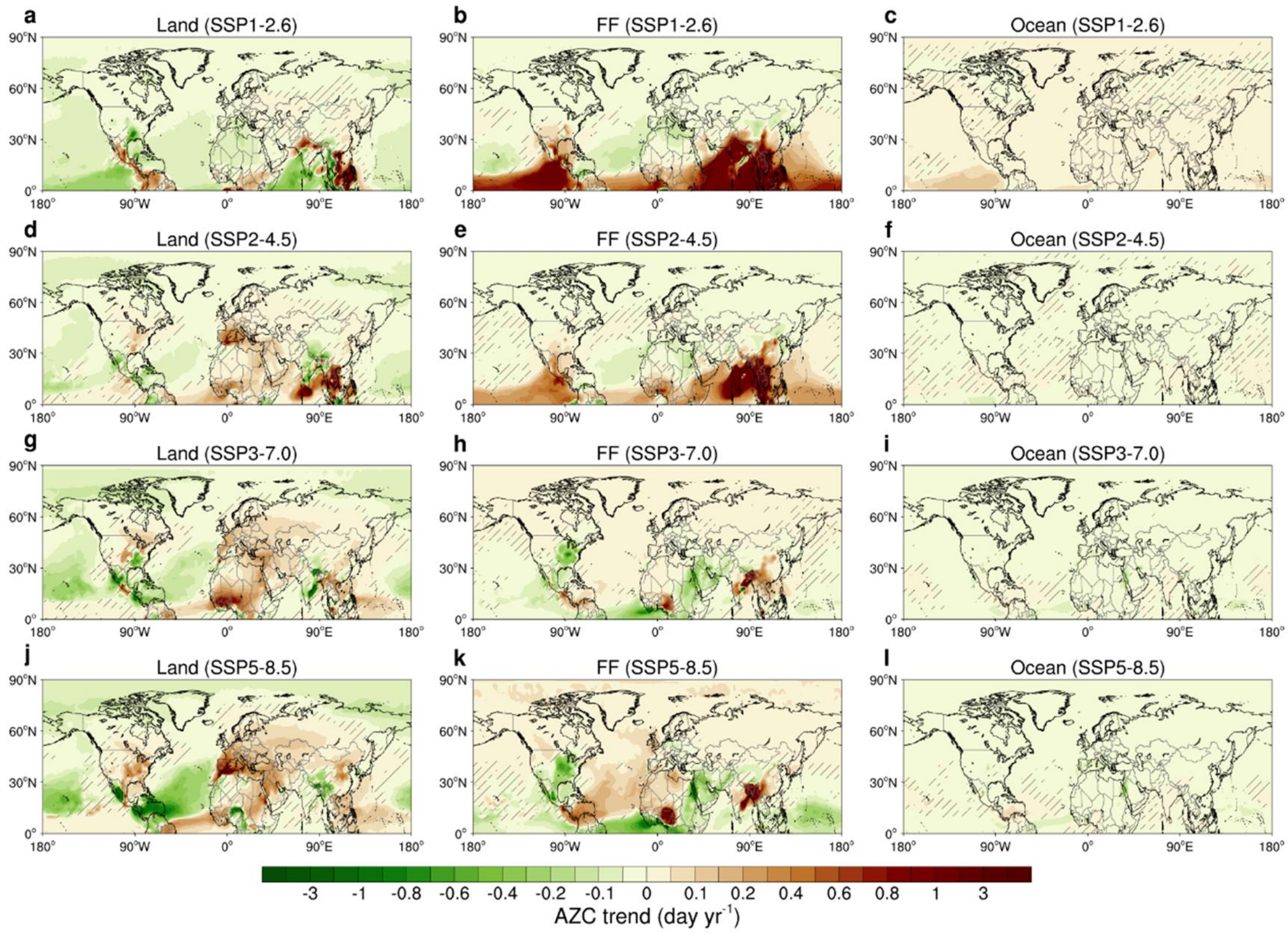

Supplementary Figure 43. Driven by time-varying meteorological fields from CanESM5, the spatial patterns of simulated AZC trends from 2030 to 2100 are attributed to variations in the terrestrial ecosystem (left panels), fossil fuel CO_2 emissions (middle panels), and air-sea CO_2 exchange (right panels). Results are shown for the SSP1-2.6 (**a-c**), SSP2-4.5 (**d-f**), SSP3-7.0 (**g-i**), and SSP5-8.5 (**j-l**) scenarios, estimated from the differences between the base simulations and the corresponding factorial simulations. The hatched areas indicate that the trends in these regions did not pass the significance test ($p > 0.05$).

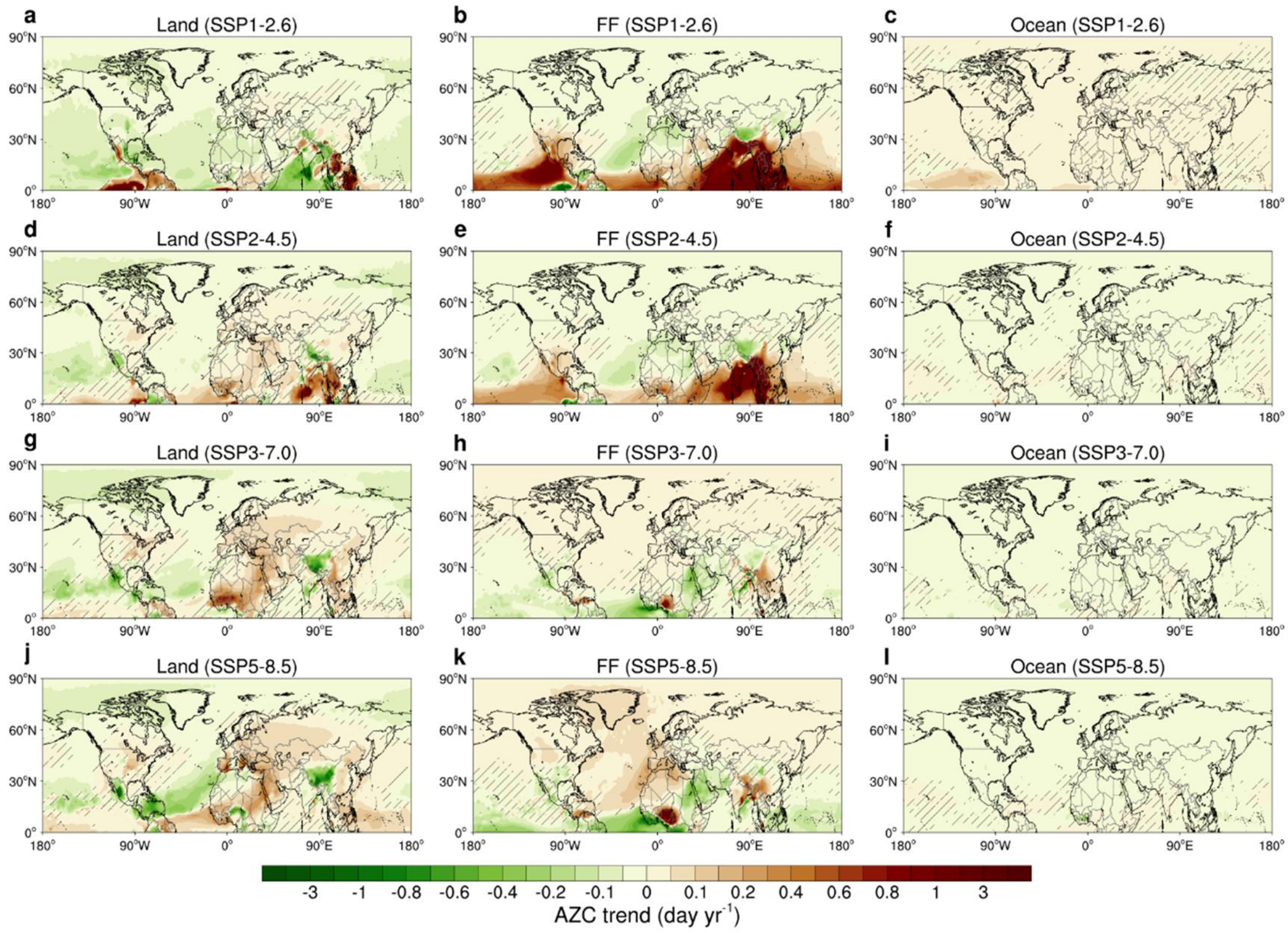

Supplementary Figure 44. Same as Supplementary Fig. 43, but showing the attribution of the AZC trend under simulations driven by meteorological fields from NorESM2-LM.

(3) Revisions incorporated into the revised manuscript

a. Methods

We have added the following sentences to the Methods section in Lines 420-426.

"To assess the potential impact of changing atmospheric circulation, we additionally performed a suite of sensitivity simulations driven by time-varying, scenario-specific meteorological fields from the CanESM5 and NorESM2-LM models (Supplementary Text 1). These tests confirm that the projected trends in CO_2 seasonality (Supplementary Figs. 33-36) and their attribution (Supplementary Figs. 37-44) are robust to the choice of meteorological forcing."

b. Supplementary Information

We have added the following texts to the Supplementary Information in Lines 12-44.

"Supplementary Text 1. Sensitivity of CO_2 Seasonal Cycle Projections to Future Meteorological Forcing

To evaluate the impact of future atmospheric circulation changes on CO_2 seasonal cycle, we performed a suite of sensitivity experiments using CMIP6 scenario-specific, time-varying meteorological data. First, we identified five key variables that dominate CO_2 atmospheric transport: surface pressure, planetary boundary layer height, zonal wind, meridional wind, and specific humidity. Among the ten ESMs that provided terrestrial and ocean CO_2 fluxes for all scenarios, only five (CanESM5, NorESM2-LM, NorESM2-MM, INM-CM4.8, and INM-CM5.0) provided all five of these meteorological variables (Supplementary Table 4). From this subset, we selected CanESM5 and NorESM2-LM as two representatives. Because CMIP6 data have coarser temporal and vertical resolution than the standard GEOS-Chem input (Supplementary Table 5), we performed piecewise linear interpolation to map the CMIP6 fields from their native pressure levels onto the 72-layer GEOS-Chem hybrid sigma-pressure grid. For layers below 1000 hPa or above 10 hPa, values from 1000 hPa or 10 hPa were used, respectively. Similar to the main experiments, we ran the baseline simulation (CMIP6_Base) and three factorial simulations (CMIP6_Land, CMIP6_FF, and CMIP6_Ocean) using the meteorological data from these two models for each scenario.

The results demonstrate that the projected trends in CO_2 seasonality are robust to the choice of meteorological forcing. Both at the BRW and MLO stations (Supplementary Fig. 33) and across the Northern Hemisphere (Supplementary Figs. 34-36), the simulated trends in SCA, SZC, and AZC during 2030-2100 under all future scenarios are largely consistent between simulations driven by constant 2021 MERRA-2 meteorological fields and those driven by time-varying meteorology from CanESM5 and NorESM2-LM. In addition, the spatial attribution patterns remain consistent (Supplementary Figs. 37-44). Under SSP1-2.6, fossil fuel emissions continue to dominate the SCA decline (Supplementary Figs. 39, 40), while under SSP5-8.5, terrestrial ecosystems remain the primary driver of changes in CO_2 seasonal cycle ((Supplementary Figs. 37, 38)).

These findings indicate that variations in future meteorological fields exert a minimal effect on the long-term trends of CO_2 seasonality compared to the dominant influence of changing surface fluxes."

Reference

Graven, H. D. et al. Enhanced seasonal exchange of CO_2 by northern ecosystems since 1960. *Science* **341**, 1085-1089 (2013).

Forkel, M. et al. Enhanced seasonal CO_2 exchange caused by amplified plant productivity in northern ecosystems. *Science* **351**, 696-699 (2016).

Piao, S. et al. On the causes of trends in the seasonal amplitude of atmospheric CO_2. *Glob. Change Biol.* **24**, 608-616 (2018).

- (c) In the future simulations, you used the ensemble-mean carbon fluxes from ten CMIP6 models. However, please note that each model exhibits its own seasonal cycle in both climate and the carbon cycle. Therefore, it would be more appropriate to run the simulations using each model's individual carbon fluxes. You could then calculate the SCA for each case and present the ensemble mean and associated uncertainties.

Response #2c:

We appreciate the reviewer's constructive comment. To quantify the uncertainty arising from differences in the carbon fluxes of individual models, we have conducted an additional suite of simulations forced by each model's individual carbon fluxes. Conducting the full analysis (10 ESMs × 4 scenarios × 4 factorial experiments) would require 160 simulations, which is computationally demanding. Therefore, we selected four representative ESMs, ACCESS-ESM1-5, CanESM5, NorESM2-LM, and MPI-ESM1-2-LR. These models were chosen to span the full range of NBP variability across the CMIP6 ensemble. As shown in Supplementary Fig. 24, CanESM5 and ACCESS-ESM1-5 represent the high and low ends of NBP, respectively, while NorESM2-LM and MPI-ESM1-2-LR are close to the ensemble mean. In addition, these four models were selected because they are included in the CMIP6 esm-ssp585 experiment, allowing for analysis and comparison in addressing the reviewer's subsequent comment (Comment #2d) on coupled carbon-climate interactions. To isolate the uncertainty associated specifically with carbon fluxes, these simulations were driven by constant 2021 MERRA-2 meteorological fields, a decision justified by our previous findings that atmospheric transport has a negligible influence on CO_2 seasonal shapes. For each model and scenario, we ran the CMIP6_Base and CMIP6_Land experiments, resulting in 32 simulations (4 carbon flux datasets

× 4 scenarios × 2 factorial simulations).

(1) Projections of trends in CO_2 seasonal metrics forced by different carbon fluxes

The four ESMs (ACCESS-ESM1-5, CanESM5, NorESM2-LM, and MPI-ESM1-2-LR) exhibit distinct NBP trends, with this inter-model spread widening under higher-emission scenarios (Supplementary Fig. 24, 45, 46). Specifically, while CanESM5, NorESM2-LM, and MPI-ESM1-2-LR project a strengthening of NBP under the SSP5-8.5 scenario, ACCESS-ESM1-5 simulates a much more limited enhancement and, in some mid-latitude regions, a significant weakening of the sink (detailed in Response #2d).

When these fluxes were used to drive the atmospheric model, the mean trends in CO_2 seasonal parameters simulated using the carbon fluxes from these four ESMs are highly similar to those obtained using the multi-model mean fluxes from all ten ESMs in the original manuscript (Figs. 1, 2, Supplementary Figs. 25-28). This close agreement suggests that the inter-model spread derived from the four individual models provides a reasonable approximation of the mean and spread of the full ten-model ensemble. The resulting trends in CO_2 seasonality from individual models reflected the underlying flux differences but revealed some consistent patterns. At the station level (BRW and MLO), nearly all models produced larger SCA trends under higher-emission scenarios, except ACCESS-ESM1-5, which relates to its small NBP trends (Supplementary Fig. 25). A consistent advance in the timing of SZC and AZC was observed under higher-emission scenarios across the model ensemble, with an exception of NorESM2-LM, which produced smaller shifts at MLO.

The global distributions of these trends show similar phenomenon. The spatial patterns of SCA trends from CanESM5, NorESM2-LM, and MPI-ESM1-2-LR closely resembled the ten-model ensemble mean shown in the manuscript (Supplementary Fig. 26), while ACCESS-ESM1-5 produced the weakest positive trends in the high latitudes and negative trends in the mid-latitude regions. The SZC and AZC trends from the four models are close to the mean of ten ESMs shown in the manuscript (Supplementary Figs. 27, 28), with an exception of NorESM2-LM showing weaker time shifts in the tropics. These differences underscore that the terrestrial carbon cycle in the tropics is the key source of inter-model uncertainty.

Although the substantial spread among different models within each scenario highlights the significant impacts of uncertain terrestrial CO_2 fluxes on atmospheric CO_2 seasonality, our primary conclusion for the mitigation scenario (SSP1-2.6) holds true across all models. They consistently showed that the trends in CO_2 seasonality are much smaller under SSP1-2.6 scenarios than the historical periods and high-emission scenarios.

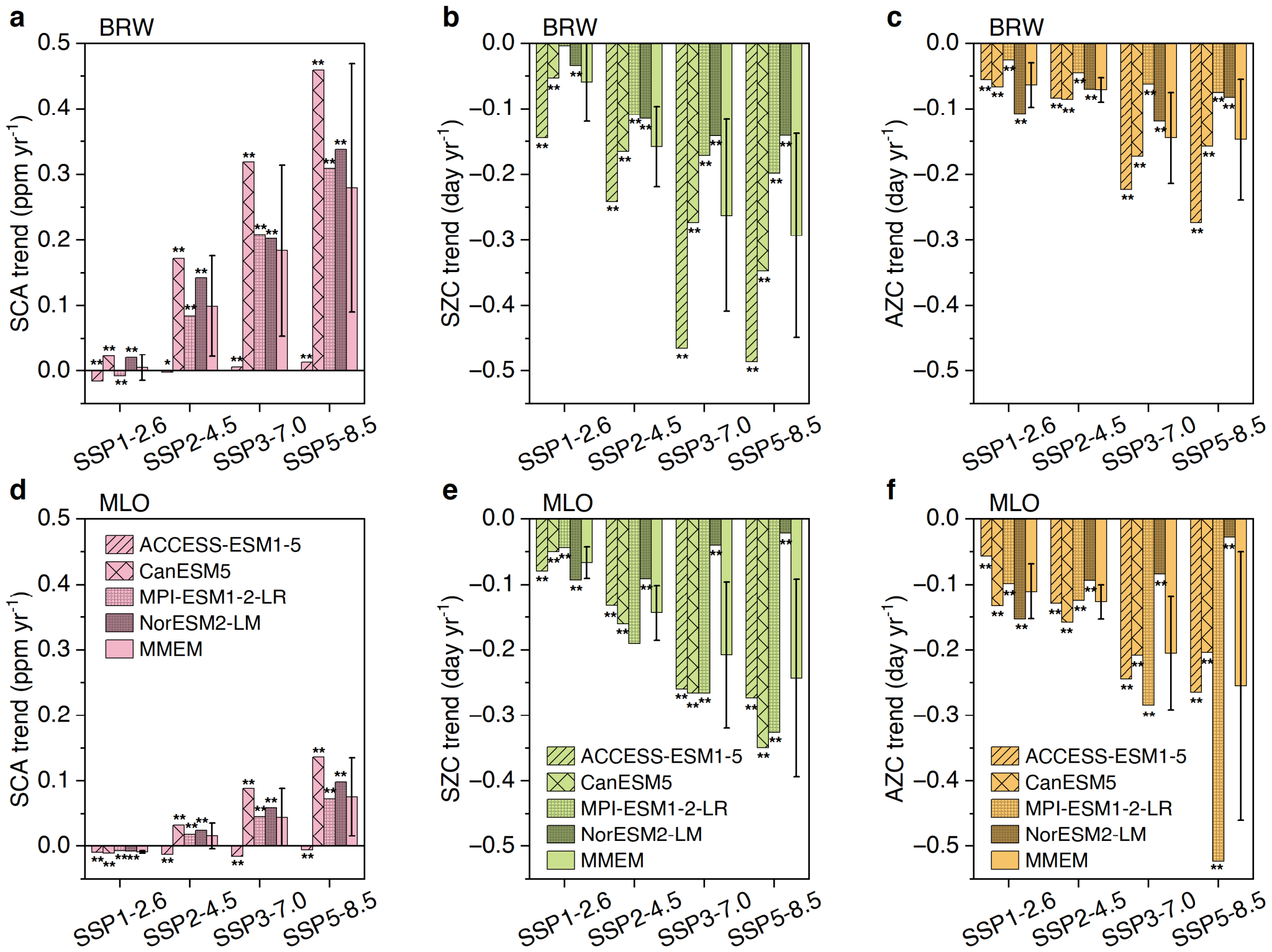

Supplementary Figure 25. Variations in the seasonality of atmospheric CO_2 under four future scenarios at the Barrow, Alaska (BRW; **a-c**), and Mauna Loa, Hawaii (MLO; **d-f**) stations. The trends were simulated using terrestrial and ocean CO_2 fluxes from four ESMs, including ACCESS-ESM1-5, CanESM5, MPI-ESM1-2-LR, and NorESM2-LM. MMEM denotes the multi-model ensemble mean across these models, and the error bars represent the inter-model standard deviation. The panels display the trends in SCA (left column), SZC (middle column), and AZC (right column). Statistically significant trends ($p < 0.05$) are marked by two dots above the bars, and marginally significant trends ($p < 0.1$) by one dot.

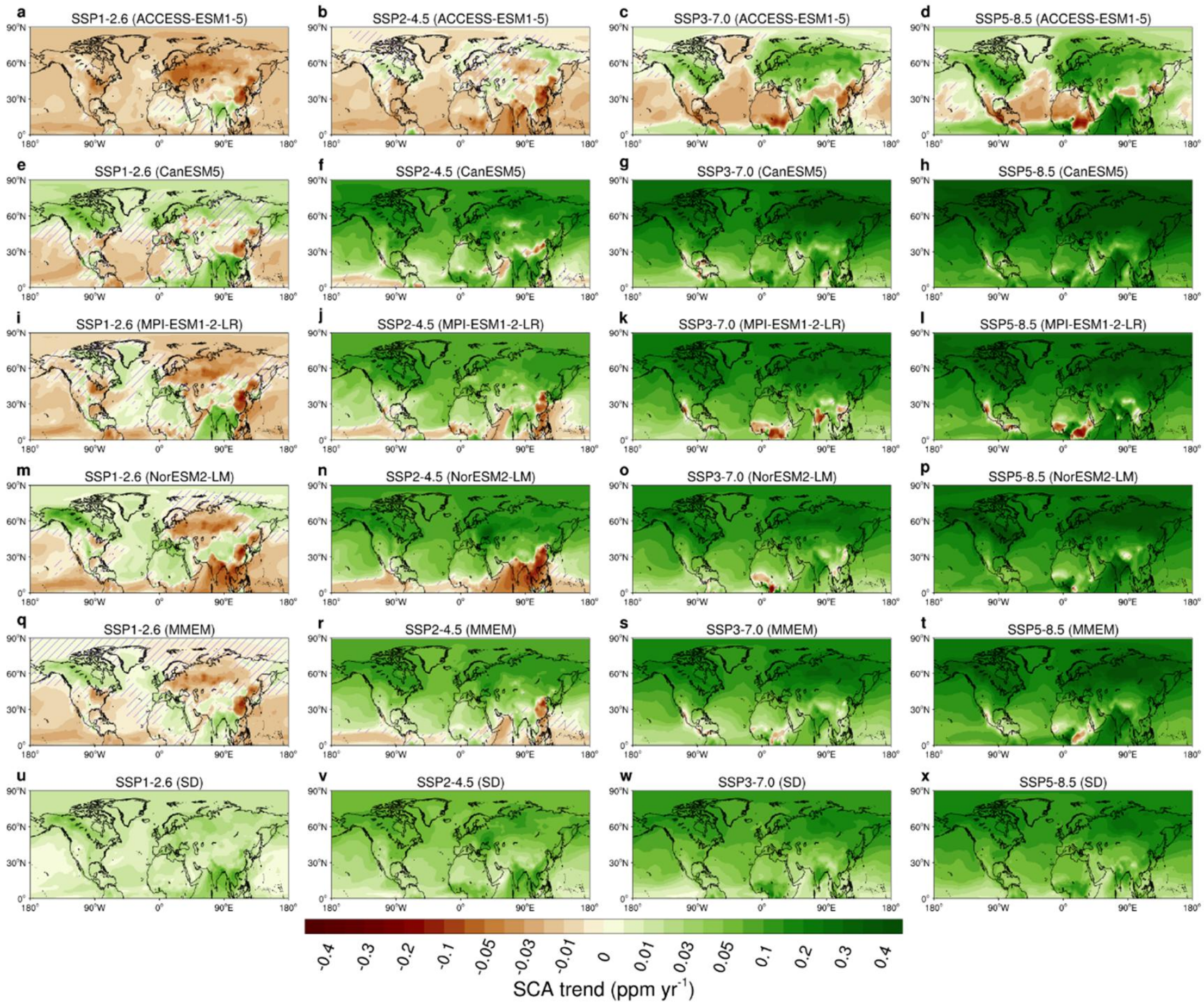

Supplementary Figure 26. Spatial patterns of simulated SCA trends across the Northern Hemisphere from 2030 to 2100, driven by different terrestrial and ocean CO_2 fluxes, under four future scenarios. Panels show SCA trends driven by terrestrial and ocean CO_2 fluxes from ACCESS-ESM1-5 (**a-d**), CanESM5 (**e-h**), MPI-ESM1-2-LR (**i-l**), and NorESM2-LM (**m-p**), along with the multi-model ensemble mean of the four estimates (**q-t**) and the standard deviation of these estimates (**u-x**). Results are shown for SSP1-2.6 (first column), SSP2-4.5 (second column), SSP3-7.0 (third column), and SSP5-8.5 (fourth column). Hatched areas denote regions where trends are not statistically significant ($p > 0.05$).

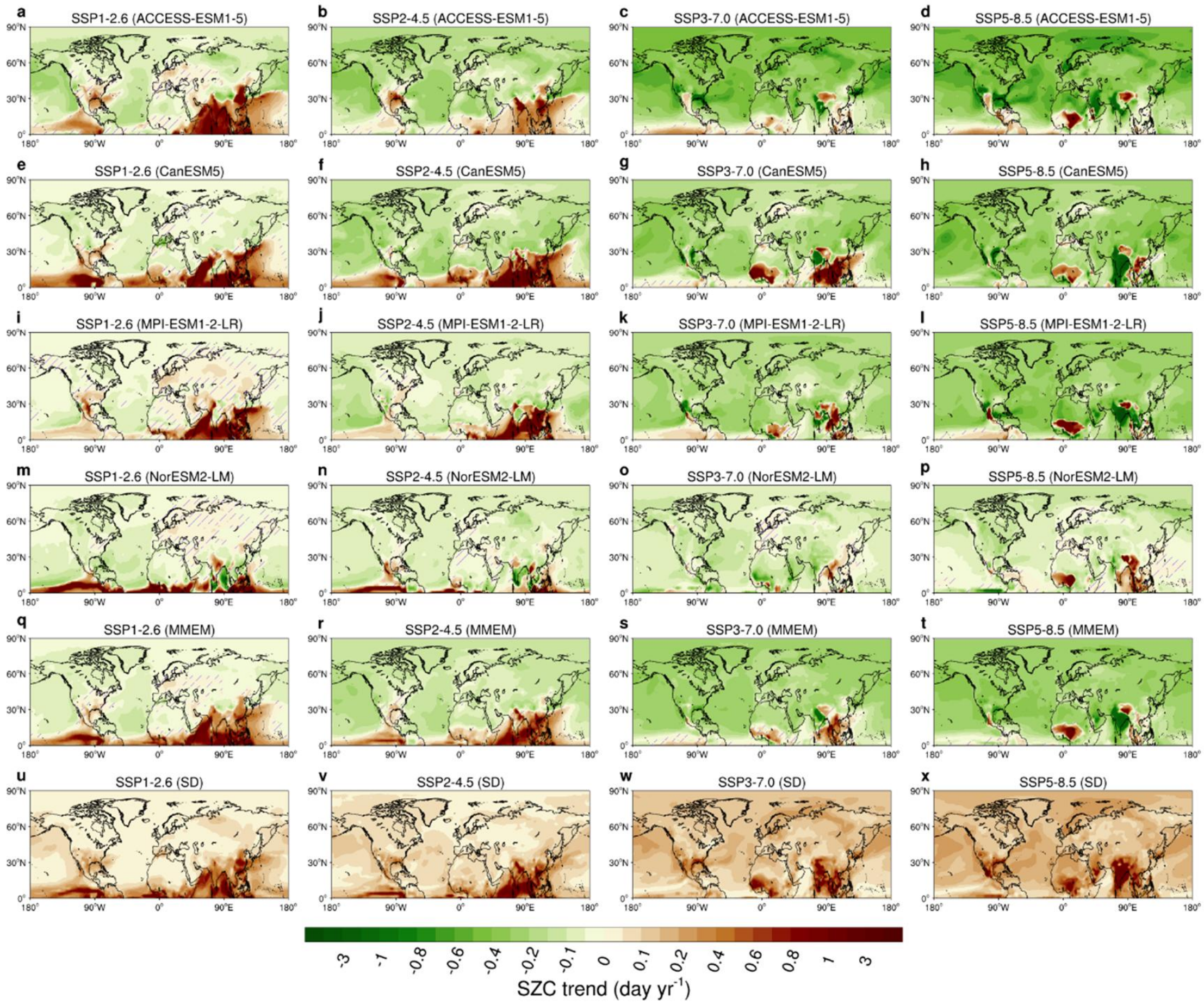

Supplementary Figure 27. Same as Supplementary Fig. 26, but showing the SZC trend.

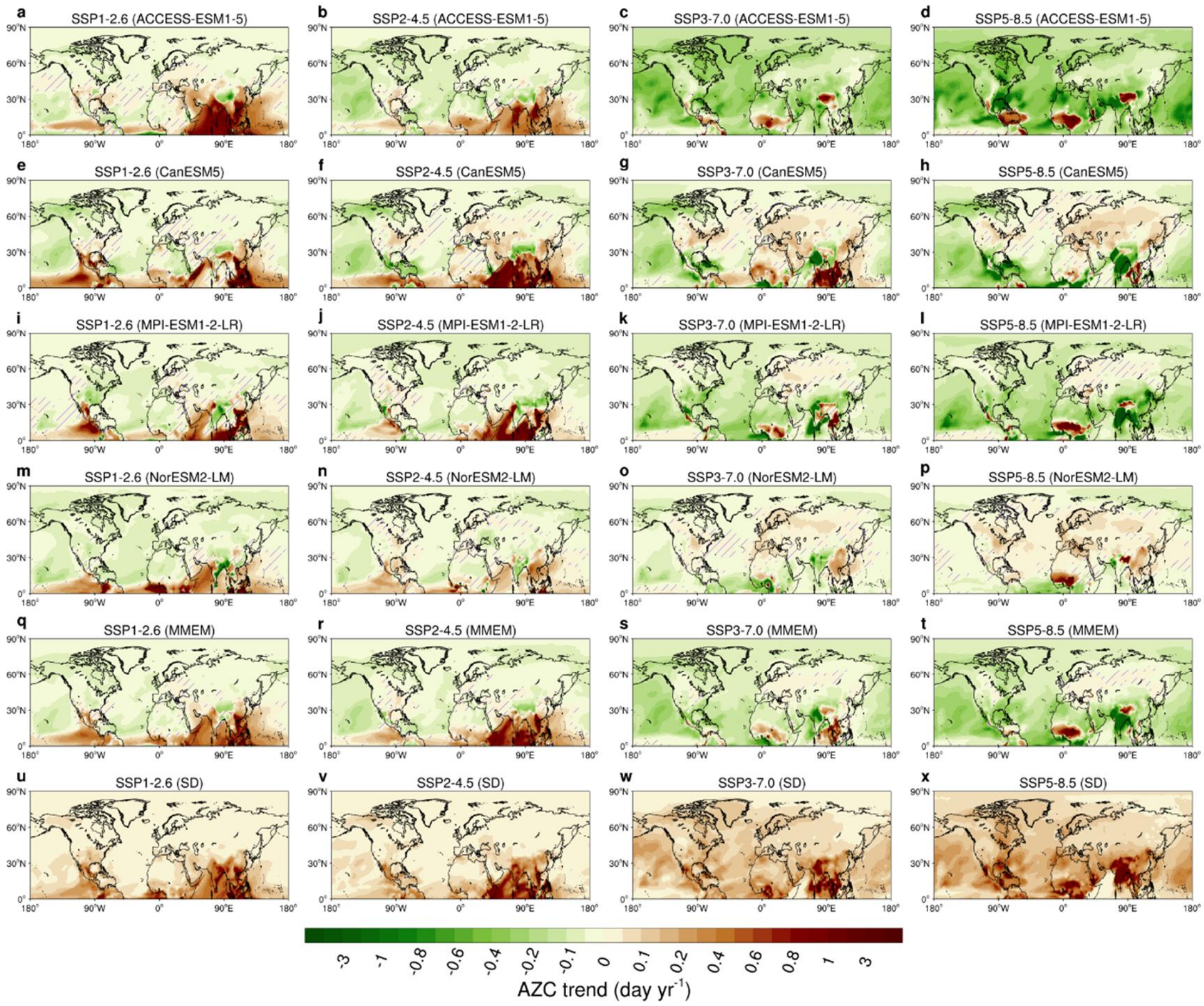

Supplementary Figure 28. Same as Supplementary Fig. 26, but showing the AZC trend.

(2) Trends in CO_2 seasonal metrics caused by different terrestrial CO_2 fluxes

Under the SSP1-2.6 scenario, the CO_2 seasonal trends simulated using different terrestrial CO_2 fluxes do exhibit some differences. But both the mean and standard deviation of the trends attributable to terrestrial ecosystems among the four models are smaller than the trends driven by fossil fuel CO_2 emissions, especially for SCA (Supplementary Figs. 29-32). This confirms our conclusion about the dominance of fossil fuel emissions over the Northern Hemisphere in CO_2 seasonality under this scenario. Under higher-emission scenarios, terrestrial ecosystems continue to be the dominant factor influencing trends in CO_2 seasonality, which is also consistent with our results in the manuscript.

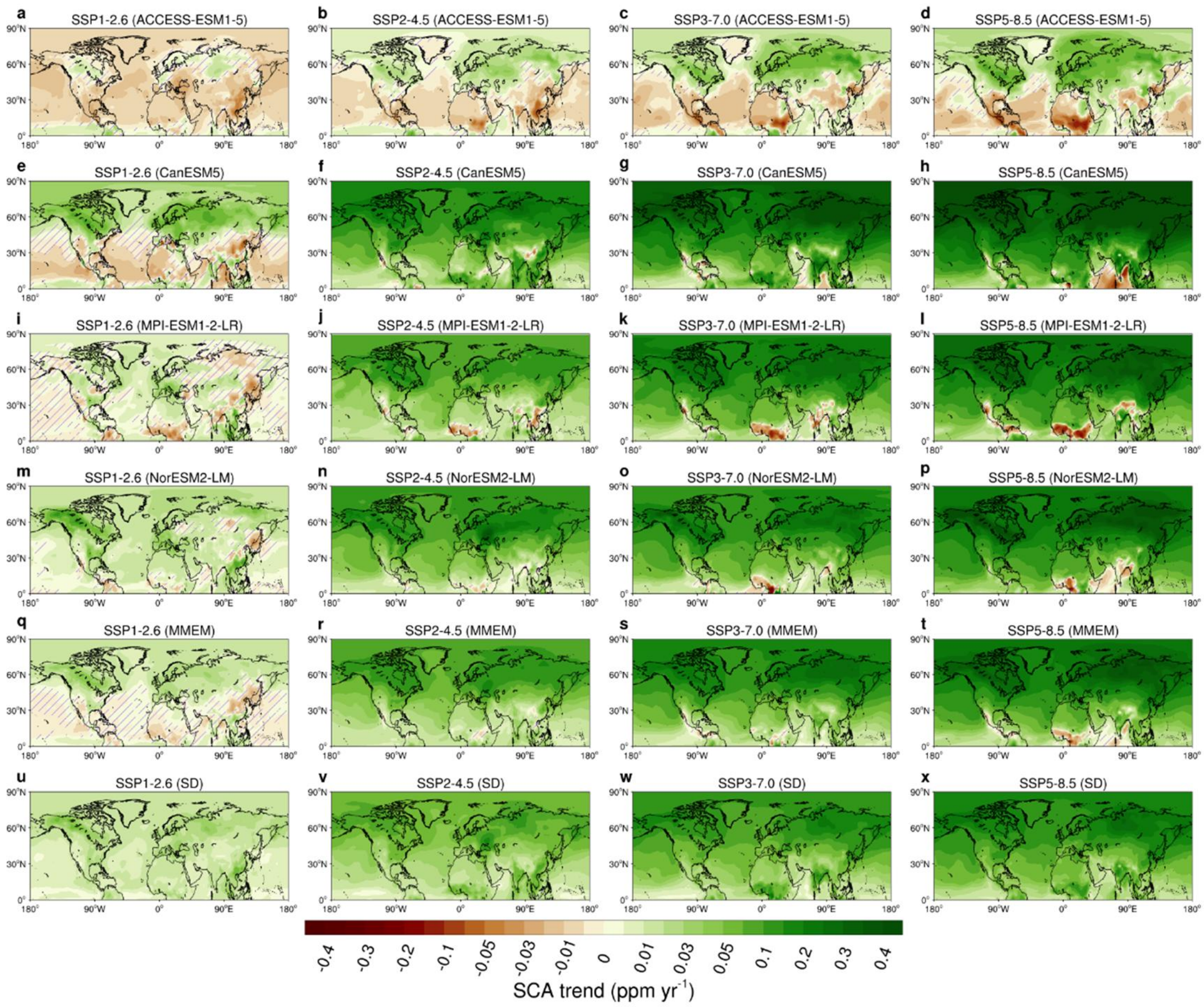

Supplementary Figure 29. Spatial patterns of SCA trends caused by terrestrial ecosystem across the Northern Hemisphere from 2030 to 2100 under four future scenarios, estimated from the differences between CMIP6_Base and CMIP6_Land. Panels show SCA trends caused by terrestrial CO_2 fluxes from ACCESS-ESM1-5 (**a-d**), CanESM5 (**e-h**), MPI-ESM1-2-LR (**i-l**), and NorESM2-LM (**m-p**), along with the multi-model ensemble mean of the four estimates (**q-t**) and the standard deviation of these estimates (**u-x**). Results are shown for SSP1-2.6 (first column), SSP2-4.5 (second column), SSP3-7.0 (third column), and SSP5-8.5 (fourth column). Hatched areas denote regions where trends are not statistically significant ($p > 0.05$).

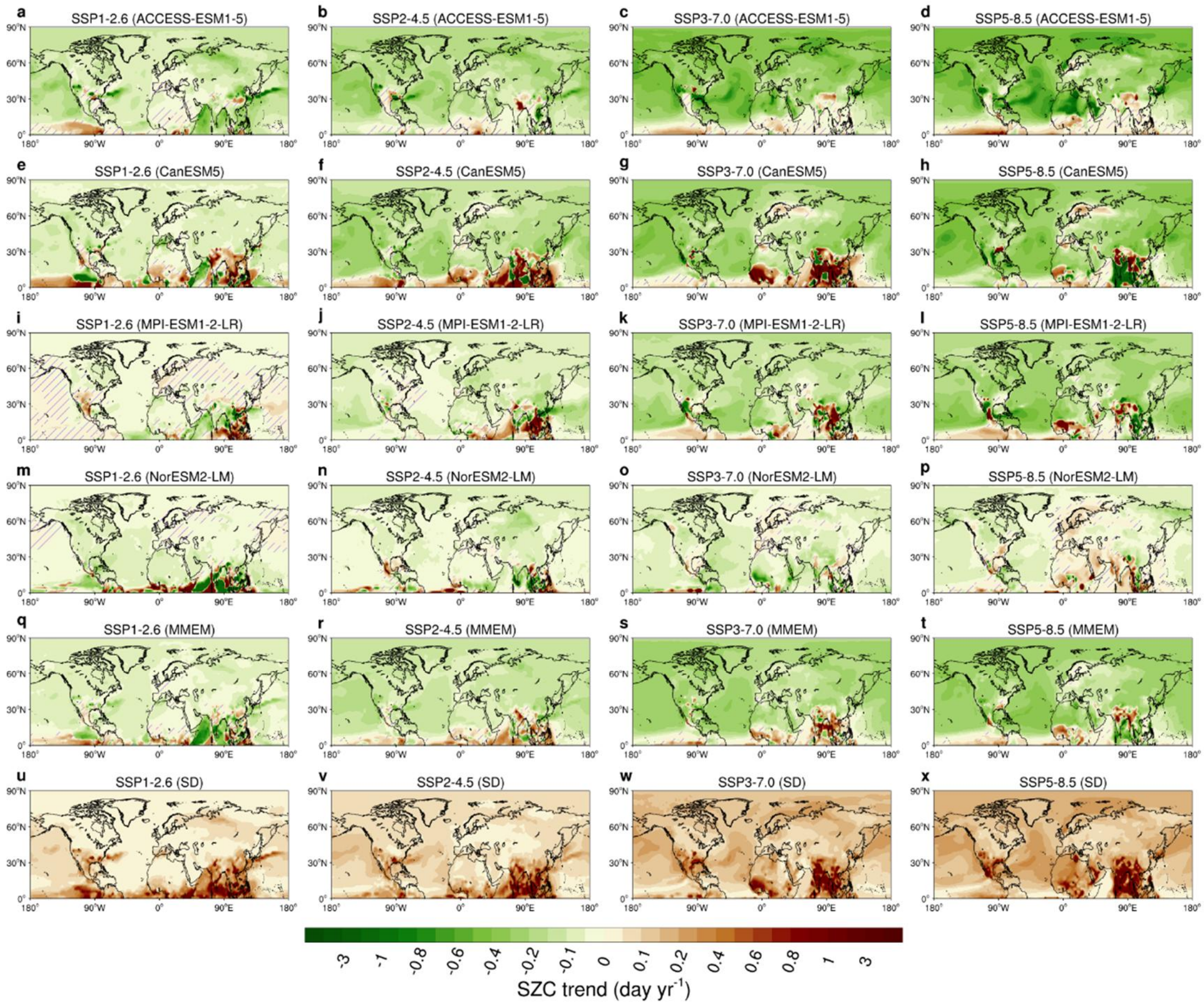

Supplementary Figure 30. Same as Supplementary Fig. 29, but showing the SZC trends caused by terrestrial ecosystems.

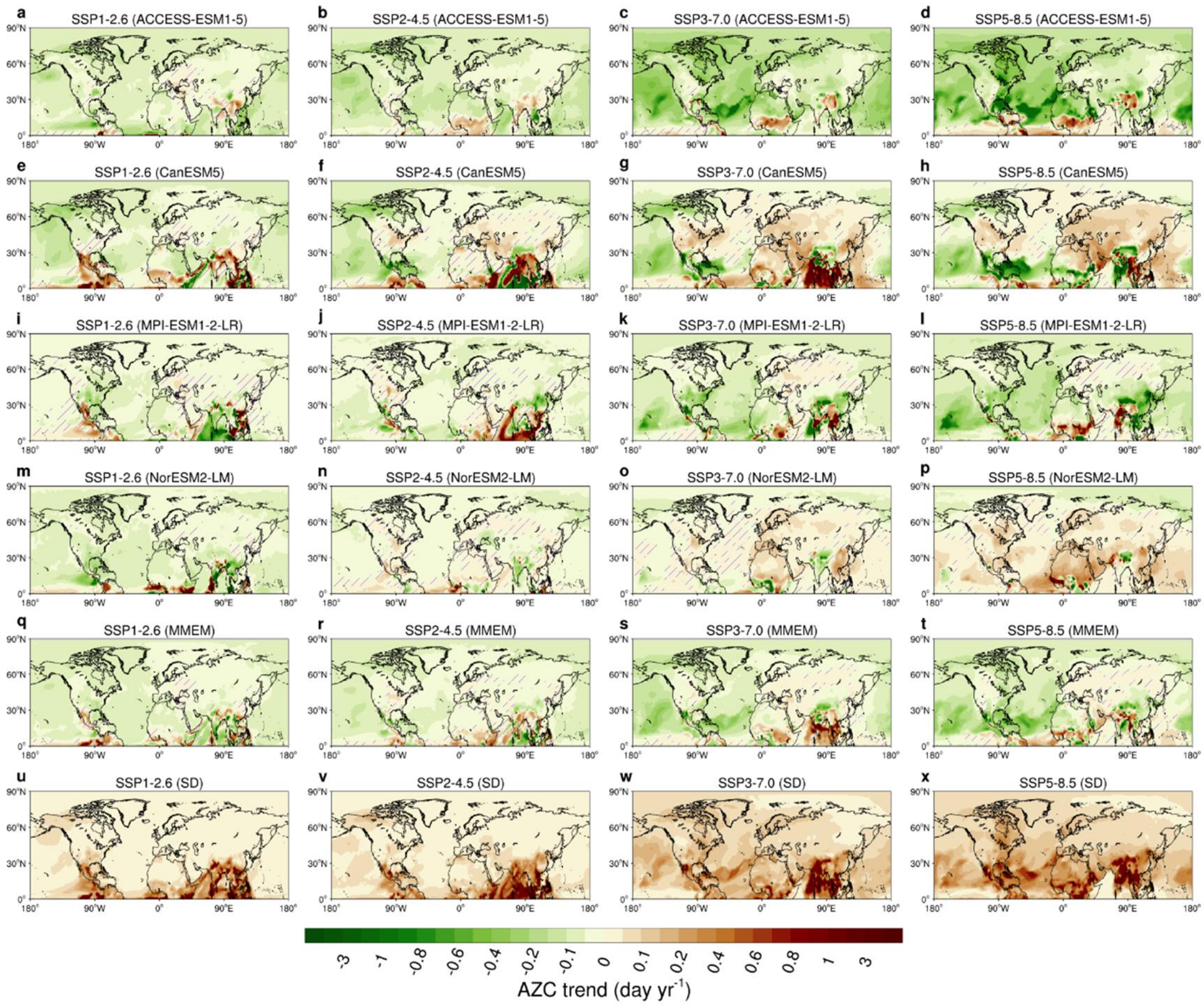

Supplementary Figure 31. Same as Supplementary Fig. 29, but showing the AZC trends caused by terrestrial ecosystems.

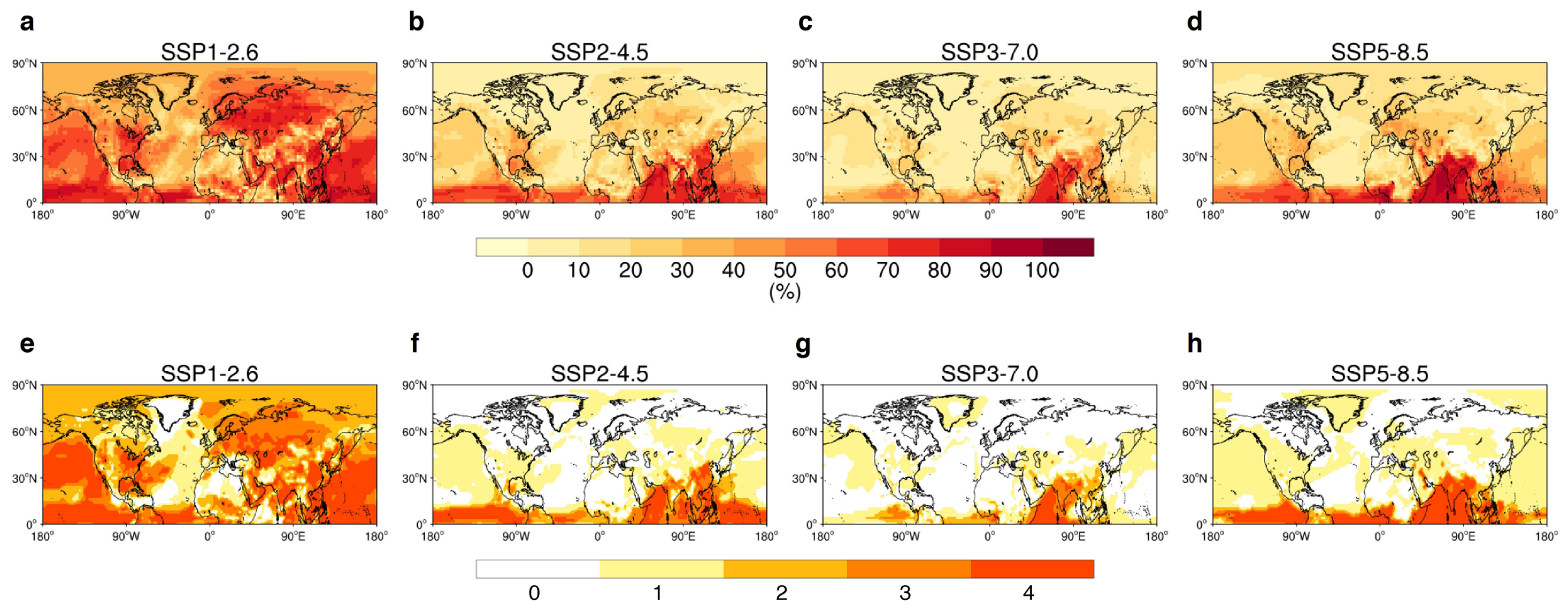

Supplementary Figure 32. Based on future transport simulations driven by carbon fluxes from four Earth system models (ACCESS-ESM1-5, CanESM5, NorESM2-LM, and MPI-ESM1-2-LR), panels (**a-d**) show the relative contribution of fossil-fuel driven SCA trends to the overall SCA trends by all components. Panels (**e-h**) show, at each grid cell in the Northern Hemisphere,

the number of models in which the contribution of fossil fuel-driven SCA trends exceeds that driven by terrestrial ecosystem. Columns from left to right correspond to the SSP1-2.6, SSP2-4.5, SSP3-7.0, and SSP5-8.5 scenarios, respectively.

(3) Revisions incorporated into the revised manuscript

a. Methods

We have added the following content to the Methods section in Lines 517-530.

"For net terrestrial and air-sea CO_2 fluxes, we used the ensemble mean estimates from ten Earth system models (ESMs) participating in the Scenario Model Intercomparison Project (ScenarioMIP)[68] under four scenarios (Supplementary Table 3). The future net terrestrial and ocean CO_2 fluxes for 2030–2100 from different ESMs are provided at a monthly resolution, though their spatial resolution varies. All the data were resampled to the 2°latitude × 2.5°longitude resolution to obtain the ensemble mean fluxes. In addition, to quantify the uncertainty in future simulations of the atmospheric CO_2 seasonal cycle and its attribution arising from uncertainties in projected terrestrial CO_2 fluxes, we conducted transport simulations driven by terrestrial CO_2 fluxes from a representative subset of four ESMs: ACCESS-ESM1-5, CanESM5, NorESM2-LM and MPI-ESM1-2-LR. These models were selected because they collectively span the full range of NBP variability within the 10-model ensemble (Supplementary Fig. 24). For each model, we performed baseline and LAND factorial simulations across all four future scenarios."

b. Discussion

We have added the following sentences to the Discussion section in Lines 305-316.

"We compared the simulated CO_2 seasonality driven by terrestrial carbon fluxes from four representative ESMs (ACCESS-ESM1-5, CanESM5, NorESM2-LM, and MPI-ESM1-2-LR) (Supplementary Figs. 25-28), selected to span the full range of terrestrial flux variability across the ten ESMs (Supplementary Fig. 24). Despite substantial inter-model differences under high-emission scenarios, all four models consistently produced small SCA trends under SSP1-2.6 (Supplementary Figs. 25, 26). More importantly, these trends are driven by fossil fuel emissions

at most regions of the Northern Hemisphere (Supplementary Fig. 29, 32). This finding confirms that under a strong mitigation pathway like SSP1-2.6, the influence of the terrestrial fluxes on atmospheric CO_2 seasonality diminishes with high model agreement, leaving fossil fuel emissions the primary driver of the changing CO_2 seasonal cycle (Supplementary Fig. 29-32)."

-(d) You mentioned that you used ten ESMs, but as far as I know, in the SSP1-2.6, SSP2-4.5, SSP3-7.0, and SSP5-8.5 scenarios, most of these models are forced with prescribed CO_2 concentrations and satellite-derived climatological LAI. For example, if the model uses CLM as the land component, these future simulations are typically conducted in this manner (although you should verify this). Only the esmSSP585 experiment requires the models to run with fully interactive carbon-climate feedbacks, including dynamic global vegetation. If your simulations are based on the former type of runs, then the resulting projections of terrestrial carbon sinks in the future might be unrealistic.

Response #2d:

We greatly appreciate this insightful comment and agree that the inclusion of climate-carbon cycle feedback is a critical consideration for future projections. The reviewer is correct that the ScenarioMIP simulations (SSP1-2.6, SSP2-4.5, SSP3-7.0, SSP5-8.5) are driven by prescribed CO_2 concentrations, and thus the land carbon sink does not fully interact with the evolving climate in the same way as in full coupled runs (O'Neill et al., 2016). To quantify the impact of these feedbacks on our conclusions, we performed a targeted comparison using the terrestrial carbon fluxes from esm-ssp585 experiment in the Coupled Climate-Carbon Cycle Model Intercomparison Project (C4MIP), which explicitly enables interactive carbon-climate feedbacks (Jones et al., 2016). Among the esm-ssp585 simulations from the r1i1p1f1 ensemble member, seven EMSs provide both terrestrial carbon fluxes and simulated gridded CO_2 concentrations. Four of them are also included in the SSP5-8.5 experiments: ACCESS-ESM1-5, CanESM5, MPI-ESM1-2-LR, and NorESM2-LM. Here, we first compared the seasonal variations in NBP during 2030-2100 under the esm-ssp585 experiment with those under SSP5-8.5. We then compared trends in SCA, SZC and AZC derived from the native CO_2 concentrations of the coupled models with those simulated by GEOS-Chem driven by the corresponding SSP5-8.5 fluxes.

(1) The projections of seasonal variations in terrestrial carbon sinks under SSP5-8.5 and esm-ssp585

All the four models show consistent seasonal behavior and variability of NBP between SSP5-8.5 and esm-ssp585 experiments (Supplementary Fig. 45). However, significant inter-model differences exist. Three of the four models (CanESM5, MPI-ESM1-2-LR, and NorESM2-LM) show an increase in the amplitude of the NBP seasonal cycle, driven primarily by enhanced carbon uptake during spring and summer and an earlier start of the growing season. This increase is most pronounced in the northern high-latitudes, which exhibit strong positive NBP trends from May to August (Supplementary Fig. 46). In contrast, ACCESS-ESM-1-5 projects a dampened NBP amplitude by 2100, with decreasing carbon uptake in mid-latitudes and slightly increasing trend in high-latitudes.

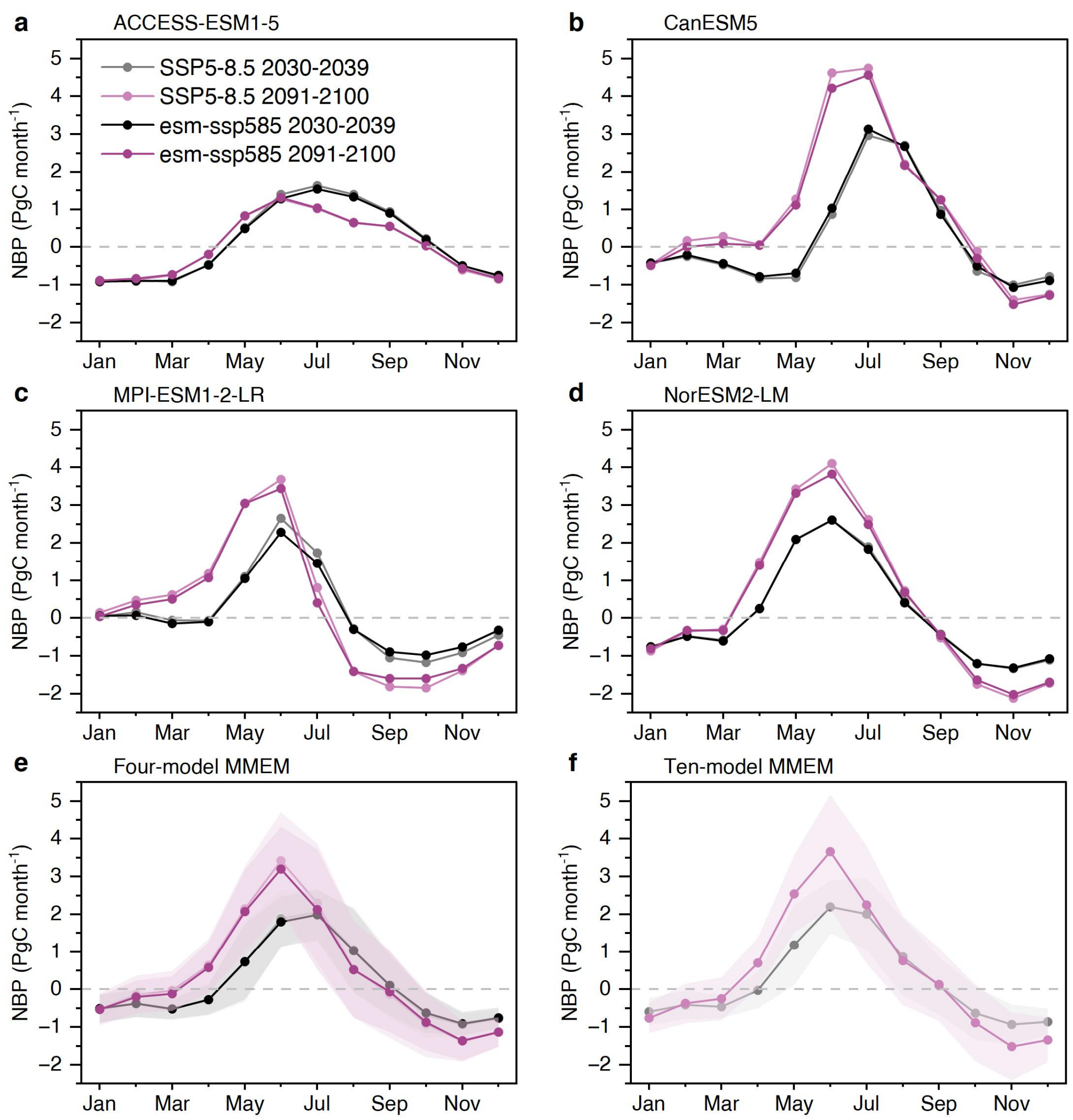

Supplementary Figure 45. Ten-year mean monthly NBP over the Northern Hemisphere for 2030-2039 and 2091-2100. Panels (a-d) show results from ACCESS-ESM1-5, CanESM5, MPI-ESM1-2-LR, and NorESM2-LM, respectively, and panel (e) shows their four-model multi-model ensemble mean (MMEM); all are shown under both the SSP5-8.5 and esm-ssp585 scenarios. Panel (f) shows the ten-model MMEM under the SSP5-8.5 scenario only. The shaded area represents the standard deviation across models. Note that MPI-ESM1-2-LR provides NBP data only through 2099, so the purple dotted lines in panel (c) represent the mean NBP for 2091-2099.

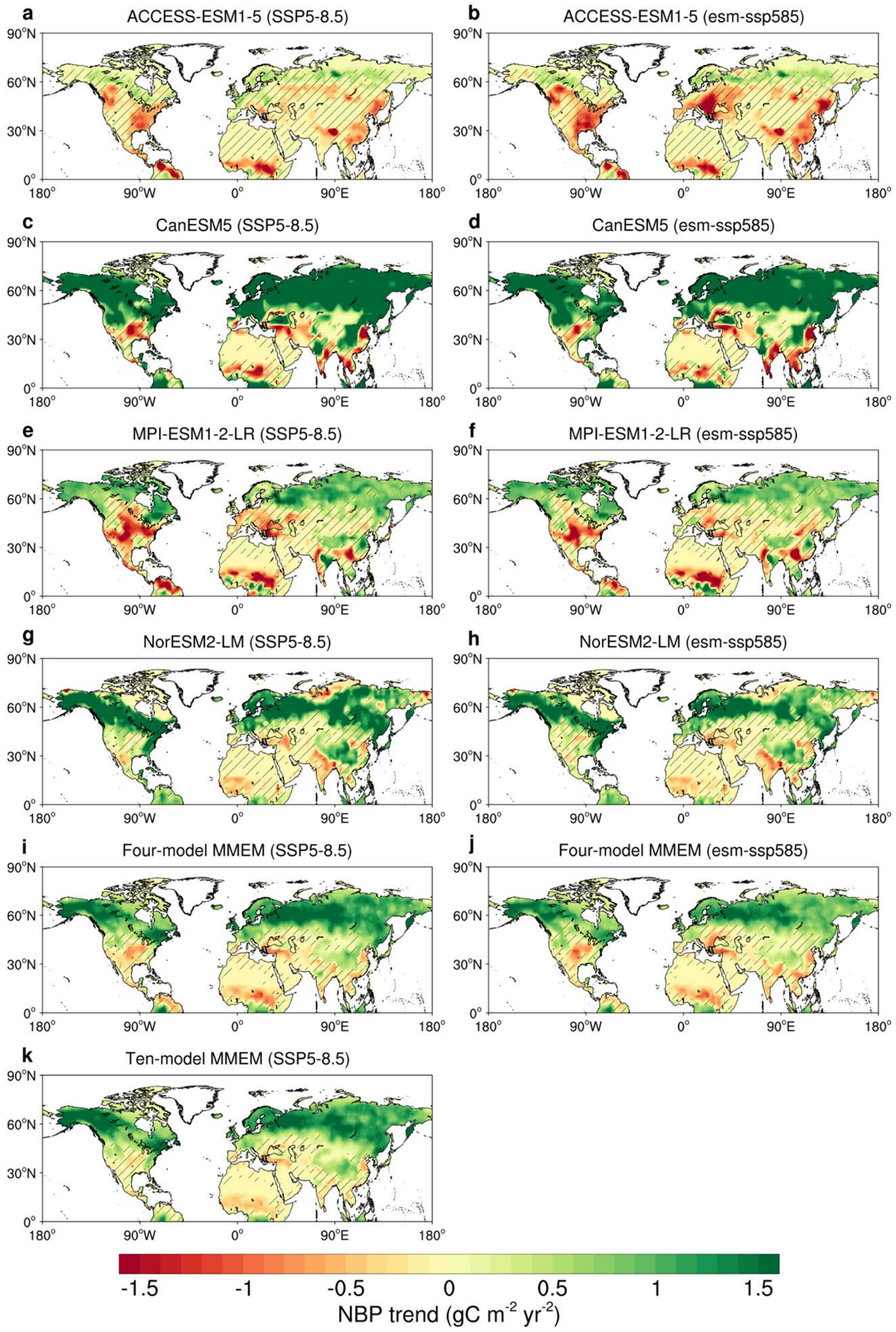

Supplementary Figure 46. Trends in integrated May-August NBP over 2030-2100 across the Northern Hemisphere. Panels show results from ACCESS-ESM1-5 (a, b), CanESM5 (c, d), MPI-ESM1-2-LR (e, f), NorESM2-LM (g, h), and their four-model multi-model ensemble mean (MMEM; i, j). Panel (k) shows the ten-model MMEM NBP trend under SSP5-8.5. The left column shows results under the SSP5-8.5 scenario, and the right column shows results under the esm-ssp585 scenario. The hatched areas indicate that the trends in these regions did not pass the significance test ($p > 0.05$). Note that MPI-ESM1-2-LR provides NBP data only through 2099, so the NBP trend from this model is calculated for 2091-2099.

(2) The projections of trends in CO_2 seasonal metrics under SSP5-8.5 and esm-ssp585

We then examined the spatial patterns of trends in SCA, SZC and AZC across the Northern Hemisphere for 2030-2100 derived from the native CO_2 concentration of the coupled ESMs in esm-ssp585 against those simulated by GEOS-Chem using corresponding SSP5-8.5 fluxes. Despite the differences in atmospheric transport (GEOS-Chem vs. native ESMs), the spatial patterns of resulting seasonality trends are broadly consistent between our results and the fully coupled simulations (Fig.2, Supplementary Fig. 47). This strong agreement confirms that while carbon-climate feedbacks are physically important, they do not fundamentally alter the dominant seasonal trends projected by the SSP concentration-driven scenarios. Therefore, the terrestrial carbon sinks used in our study are sufficient to capture the key drivers of future CO_2 seasonality.

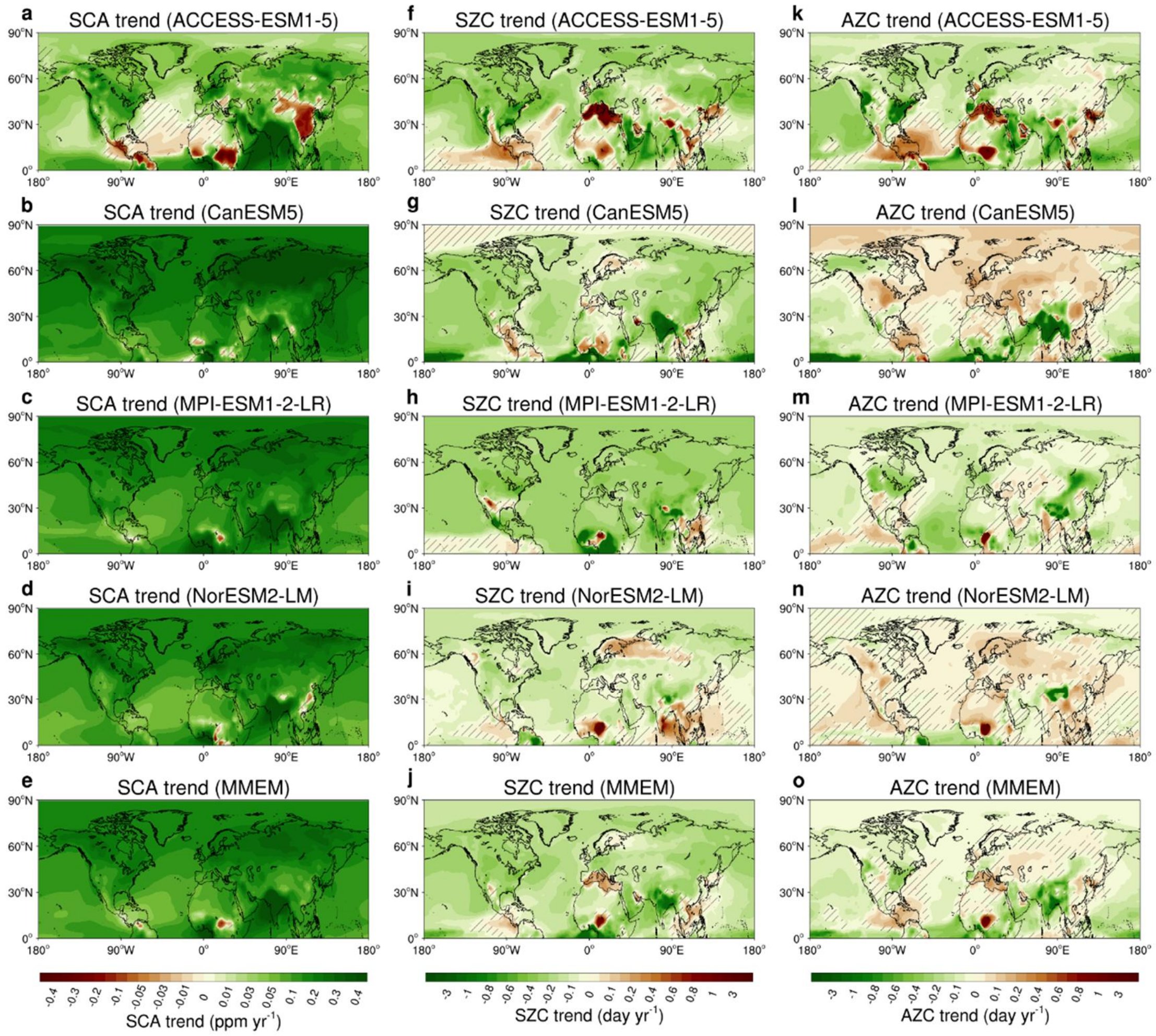

Supplementary Figure 47. Spatial patterns of simulated trends in SCA (**a-e**), SZC (**f-j**), and AZC (**k-o**) over 2030-2100 across the Northern Hemisphere from the esm-ssp585 simulations. Panels show results from ACCESS-ESM1-5 (first row), CanESM5 (second row), MPI-ESM1-2-LR (third row), NorESM2-LM (fourth row), and the ensemble-mean estimates from these four models (bottom row). The hatched areas indicate that the trends in these regions did not pass the significance test ($p > 0.05$). Note that MPI-ESM1-2-LR provides atmospheric CO_2 concentration data only through 2099, so the trends of CO_2 seasonal parameters from this model were calculated for 2030-2099.

(3) Revisions incorporated into the revised manuscript

We have added the following sentences to the Methods section in Lines 427-438.

"We also assessed the impact of carbon-climate feedbacks on the simulation of CO_2 seasonality trends, by performing targeted comparison using the terrestrial carbon fluxes from esm-ssp585

experiment in the Coupled Climate-Carbon Cycle Model Intercomparison Project (C4MIP), which includes such feedbacks[56]. ACCESS-ESM1-5, CanESM5, MPI-ESM1-2-LR, and NorESM2-LM are the models that participated in both ScenarioMIP SSP5-8.5 and C4MIP esm-ssp585 experiments (Supplementary Figs. 45, 46). We examined the spatial patterns of trends in SCA, SZC and AZC from the native CO_2 concentration output of the coupled models. Despite differences in atmospheric transport physics, the seasonality trends remain broadly consistent between these coupled simulations and those simulated by GEOS-Chem using SSP5-8.5 fluxes (Supplementary Fig. 47). This confirms that our primary analysis is sufficient to capture the dominant drivers of future atmospheric CO_2 seasonality."

Reference

O'Neill, B. C. et al. The Scenario Model Intercomparison Project (ScenarioMIP) for CMIP6. *Geosci. Model Dev.* **9**, 3461-3482 (2016)

Jones, C. D. et al. C4MIP - The Coupled Climate-Carbon Cycle Model Intercomparison Project: experimental protocol for CMIP6. *Geosci. Model Dev.* **9**, 2853-2880 (2016).

(3) I understand that you calculated the SCA as the difference between the maximum and minimum CO_2 mole fractions within each calendar year. If so, when you conducted the factorial simulations by isolating the carbon fluxes from land, ocean, and fossil fuel emissions (FFE), the derived SCA in each experiment would represent different physical meanings. In your baseline simulation, for instance, the SCA might be determined by the difference between April and September CO_2 levels, while in the CMIP6_FF experiment, the timing of the maximum and minimum could shift, potentially occurring in different months. I am not sure whether I have understood this correctly — could you please clarify how you defined and compared the SCAs across different factorial simulations?

Response #3:

We thank the reviewer for this important question regarding the physical interpretation of our factorial analysis. The reviewer is correct that if the seasonal timing differed substantially between experiments, comparing their amplitudes (SCA) would be physically ambiguous.

First, we clarify that for station-level comparison between historical simulations and observations, all data were processed to match the temporal frequency of observation records, which was typically weekly. These data were fitted using the curve fitting algorithm CCGCRV and the resulting function was interpolated to a daily resolution to compute the SCA. For historical and future gridded simulations, the CO_2 fields were sampled at a daily resolution, and the curve fitting algorithm and computation of SCA procedures were applied at this daily frequency. As a result, the peak and trough dates defining the SCA have daily resolution.

To address the reviewer's concern regarding whether these dates differ substantially among simulations, we compared the peak and trough dates between factorial simulations (CMIP6_FF, CMIP6_Land and CMIP6_Ocean) and the base simulation (CMIP6_Base). While small differences exist, they are typically less than five days (Response Figs. 1, 2). This stability arises because the seasonality of the fluxes is not removed, but is either held constant or allowed to vary. For example, in CMIP6_FF, the seasonality of land and ocean fluxes are identical to those in CMIP6_Base, while FF emission (including both annual total and seasonality) is fixed at its 2030 state. The peak and trough dates of CO_2 seasonality in the two simulations are very similar, reflecting strong carbon uptake by terrestrial ecosystems during the growing season and carbon release during the non-growing season. Similarly, in CMIP6_Land, the seasonality of the terrestrial carbon sink is fixed at its 2030 state but remains pronounced, yielding a May peak and an October trough comparable to those in CMIP6_Base.

Since the seasonal timing remains stable, the SCA values represent the same physical seasonal window. Therefore, subtracting the SCA of a factorial run from the baseline run effectively isolates the contribution of the specific flux component without being confounded by phase shifts.

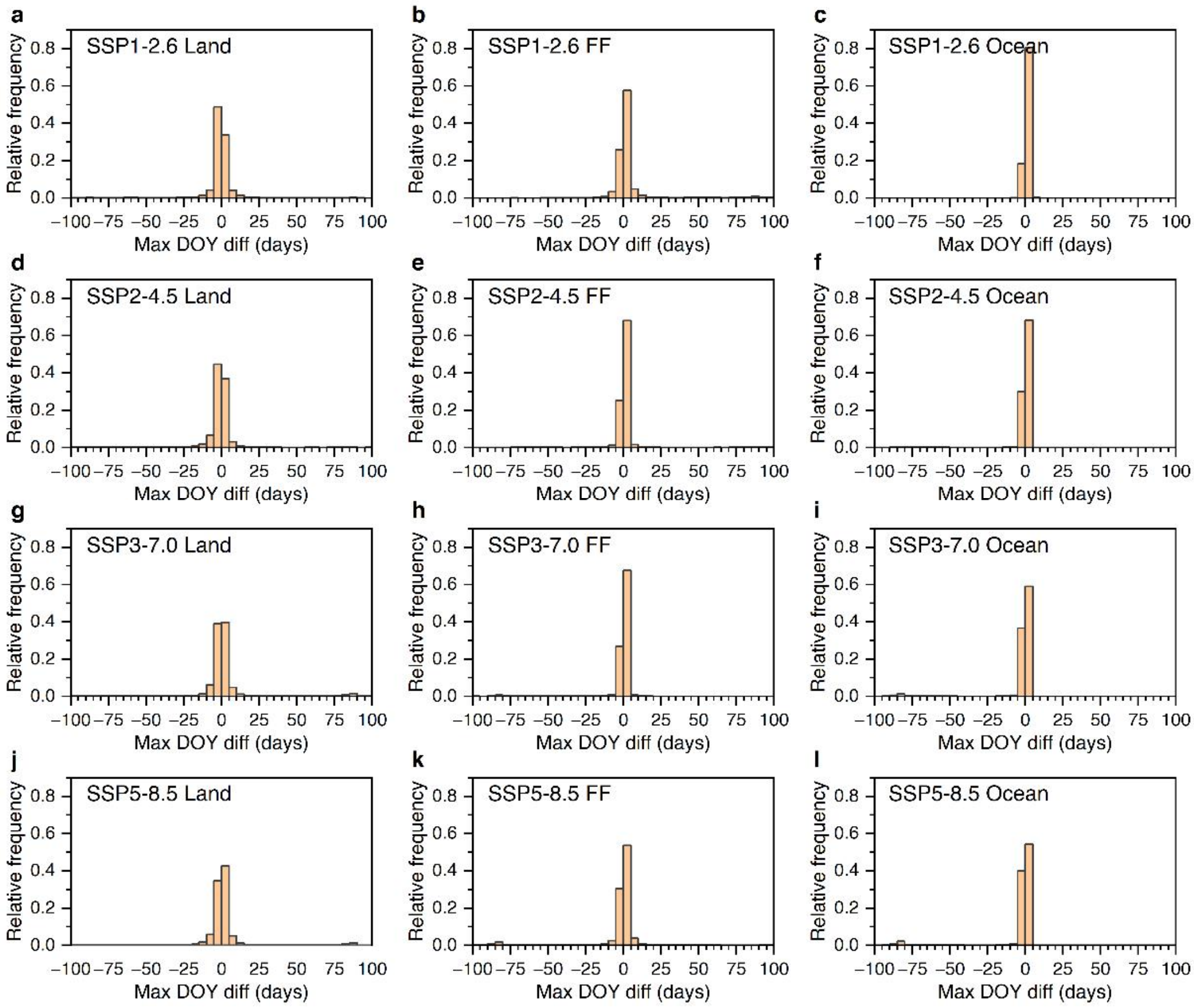

Response Figure 1. Frequency distributions of differences in the dates of maximum detrended CO_2 concentration between the baseline simulation and each factorial simulation under the SSP1-2.6 (**a-c**), SSP2-4.5 (**d-f**), SSP3-7.0 (**g-i**), and SSP5-8.5 (**j-l**) scenarios. The distributions include differences from all Northern Hemisphere grid cells and all years from 2030 to 2100. The first column shows differences between CMIP6_Base and CMIP6_Land, the second between CMIP6_Base and CMIP6_FF, and the third between CMIP6_Base and CMIP6_Ocean.

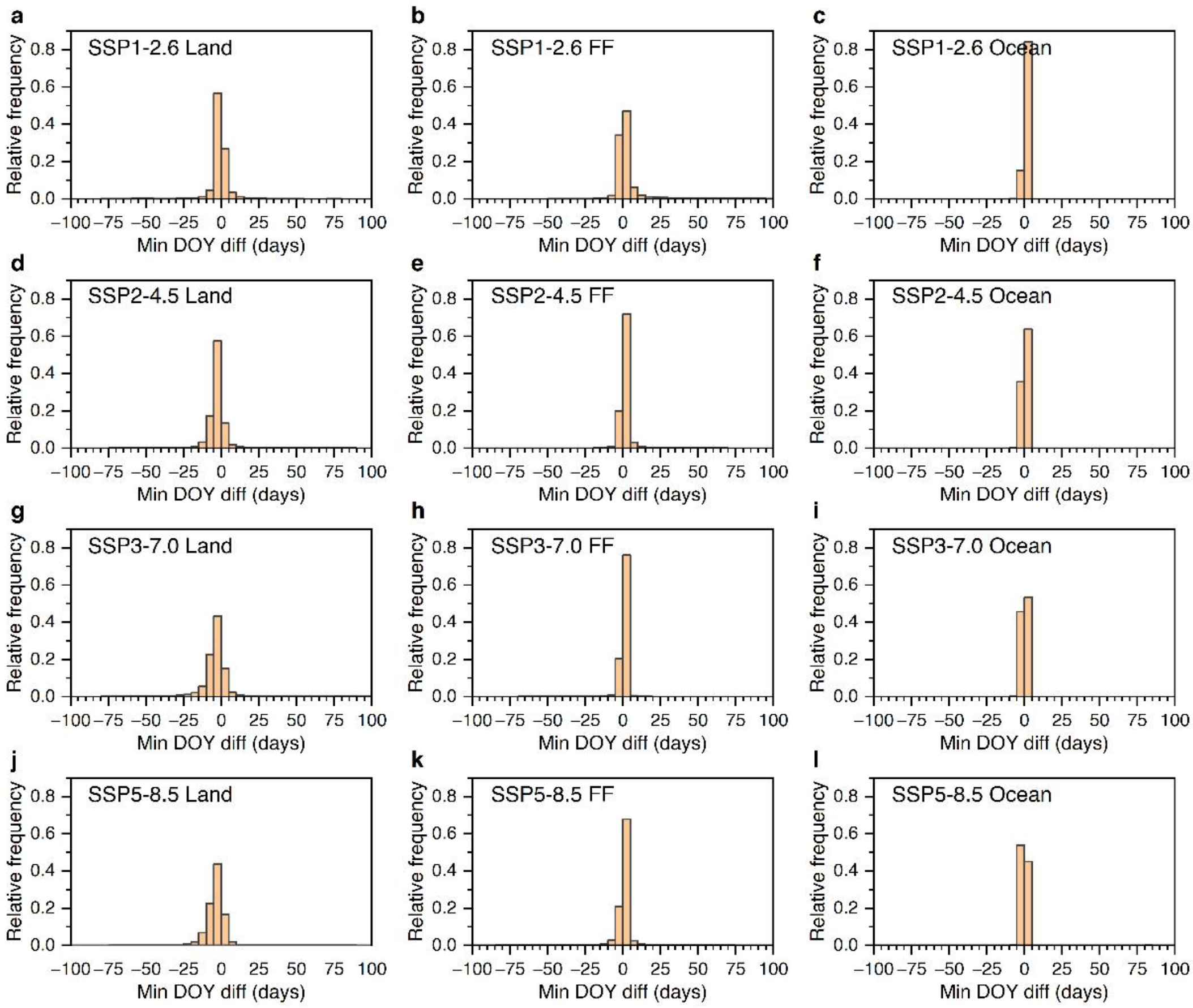

Response Figure 2. Same as Response Fig.1, but showing the frequency distributions of differences in the dates of minimum detrended CO_2 concentration.

We also added the following sentences to the Methods section in Lines 441-444.

"Taking the fossil fuel-driven SCA trend as an example, we subtracted the SCA time series of the CMIP6_FF from the CMIP6_Base. Fossil fuel emissions' contribution to the SCA trend was then defined as the linear trend of this difference time series, estimated using the Theil-Sen method."

(4) You conclude that in SSP1-2.6, the decline in SCA is predominantly driven by fossil fuel emissions rather than land sinks. However, this conclusion critically depends on the accuracy of the seasonal cycle of fossil fuel emissions (FFE). Although you present some results for Europe, China, and the United States, I can confirm that the variation over China shown in Supplementary Figure 10e is incorrect. According to datasets such as MEIC and CDIAC, FFE over China typically peaks in December and reaches a minimum in January or February. Therefore, before drawing this conclusion, you need to first examine and compare the seasonal

variations of FFE in several key regions between the datasets you used and other widely recognized inventories (e.g., MEIC, CDIAC). Otherwise, the validity of this conclusion remains questionable.

Response #4:

We thank the reviewer for this critical comment. We acknowledge that the FFE seasonality for China in the CMIP6 dataset differs from observational inventories such as MEIC, which show a peak in December and a minimum in February. This discrepancy arises because the CMIP6 monthly emission profiles are prescribed using seasonality fractions from the ECLIPSE dataset (Feng et al., 2020), rather than derived from country-specific observational data. The original Supplementary Fig. 10e (now renumbered as Supplementary Fig. 14e, with y-axis units revised to TgC month^{-1}) reflects this CMIP6 standard input.

To address this concern, we first verified the FFE seasonality in our historical simulations, where emissions are taken from MEIC. Supplementary Fig. 17 confirms that China's historical FFE exhibits a minimum in February and a peak in December, consistent with the reviewer's expectation. We also compared this MEIC seasonality to the reference GridFED dataset used by the Global Carbon Project. For large emitters like China, the USA, and Europe, the two inventories show consistent seasonal FFE patterns over the historical period.

We then investigated the impact of using a different FFE seasonality on our future projections. Specifically, we applied the historical (2017-2021 average) monthly emission fractions from MEIC to replace the original seasonality within the CMIP6 scenarios for each model grid cell. The resulting FFE seasonality for China, the USA and Europe is shown in Supplementary Fig. 18. We used this rescaled emission dataset to drive the transport simulations CMIP6_Base and CMIP6_FF under four future scenarios. Terrestrial and ocean CO_2 fluxes were taken from the multi-model mean of ten ESMs, and the meteorology was held constant using MERRA-2 fields for 2021. This setup was chosen to save the computing resources, and is justified by the previous responses, which showed that uncertainties in these components do not alter our conclusion.

The simulated trends in SCA are nearly identical to those produced by the original CMIP6

emissions (Supplementary Fig. 19). More importantly, under SSP1-2.6, the decline in SCA remains predominantly driven by fossil fuel emissions, even with the adjusted seasonality. This robustness arises because the atmospheric CO_2 seasonal amplitude is determined by the integral of fluxes over the accumulation (winter/spring) and drawdown (summer) periods. While the precise timing of the monthly peak shifts between ECLIPSE and MEIC, the cumulative emissions during these seasonal windows remain similar.

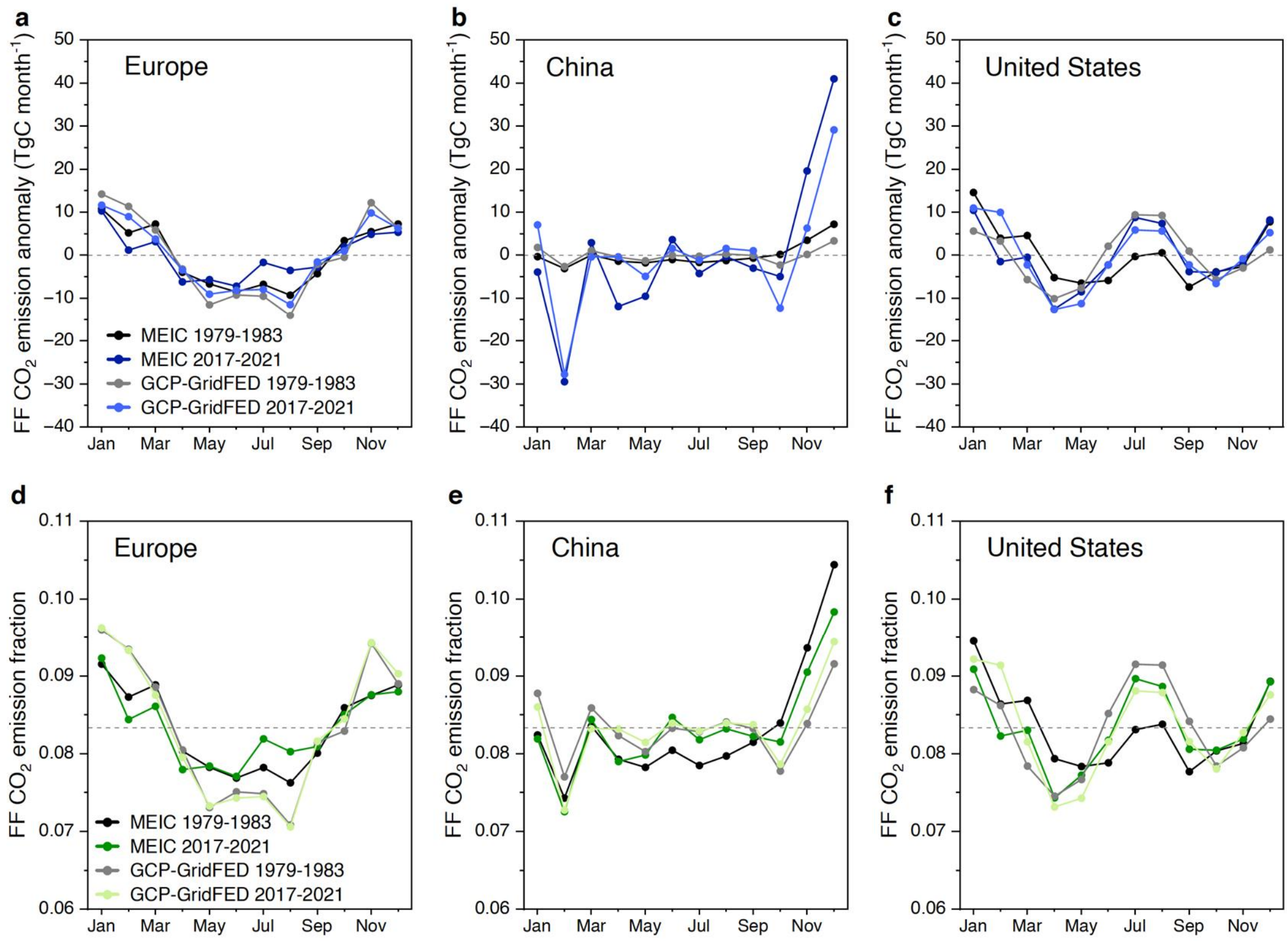

Supplementary Figure 17. Fossil fuel CO_2 emission anomalies (monthly emissions minus the annual-mean monthly emission; **a-c**) and fossil fuel CO_2 emission fractions (monthly emissions divided by the annual total emission; **d-f**) averaged over 1979-1983 and 2017-2021, estimated from the MEIC and GCP-GridFED datasets. Panels show emissions from Europe (left column), China (middle column), and the United States (right column).

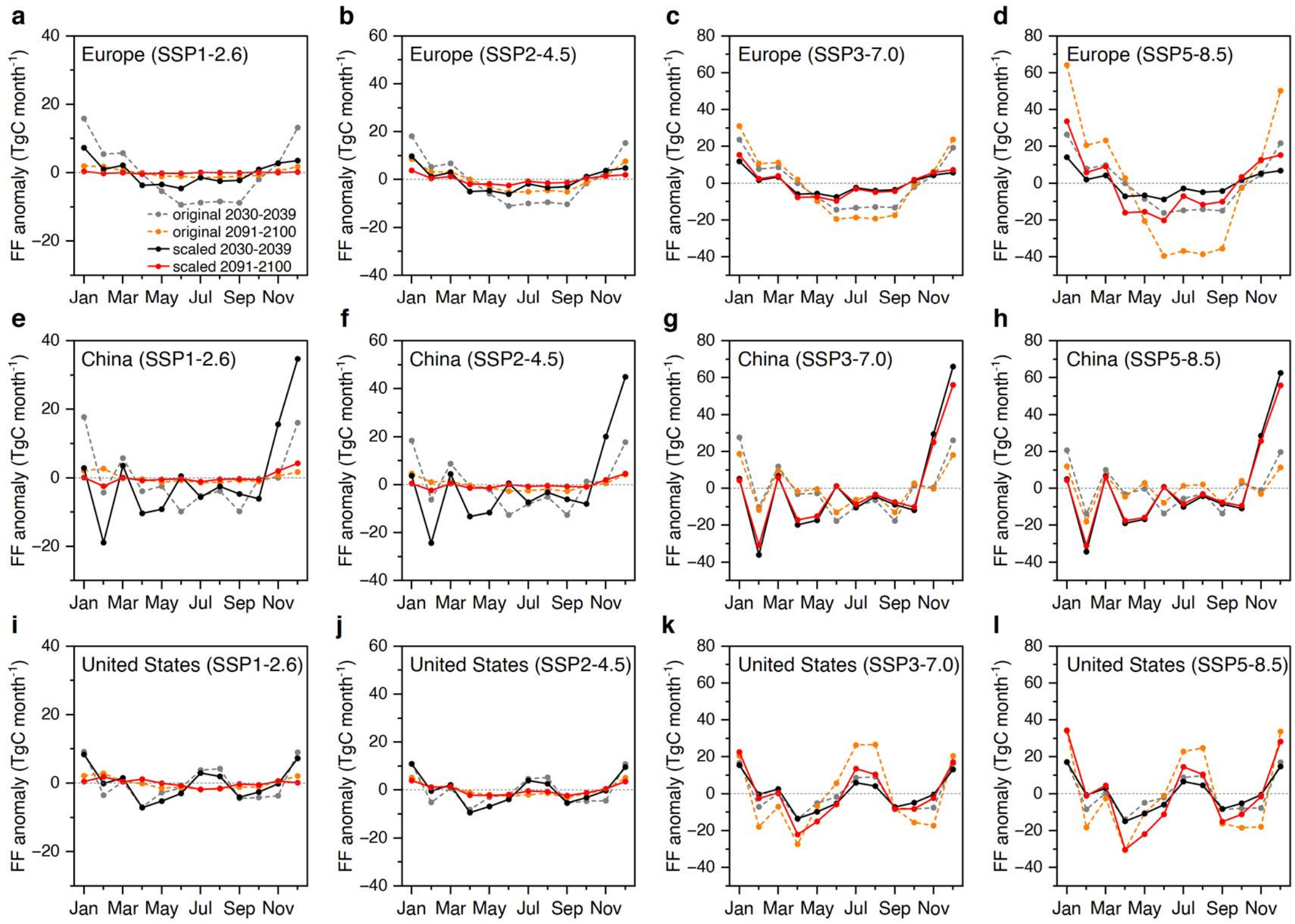

Supplementary Figure 18. Future fossil fuel CO_2 emission anomalies (monthly emissions minus the annual-mean monthly emission) averaged over 2030-2039 and 2091-2100, estimated using (i) the original CMIP6 gridded monthly emissions (original) and (ii) monthly emissions calculated by multiplying the CMIP6 annual total emissions with the 2017-2021 mean monthly fossil fuel CO_2 emission fractions derived from MEIC (scaled). Results are shown for Europe (**a-d**), China (**e-h**), and the United States (**i-l**) under the SSP1-2.6 (first column), SSP2-4.5 (second column), SSP3-7.0 (third column), and SSP5-8.5 (fourth column) scenarios.

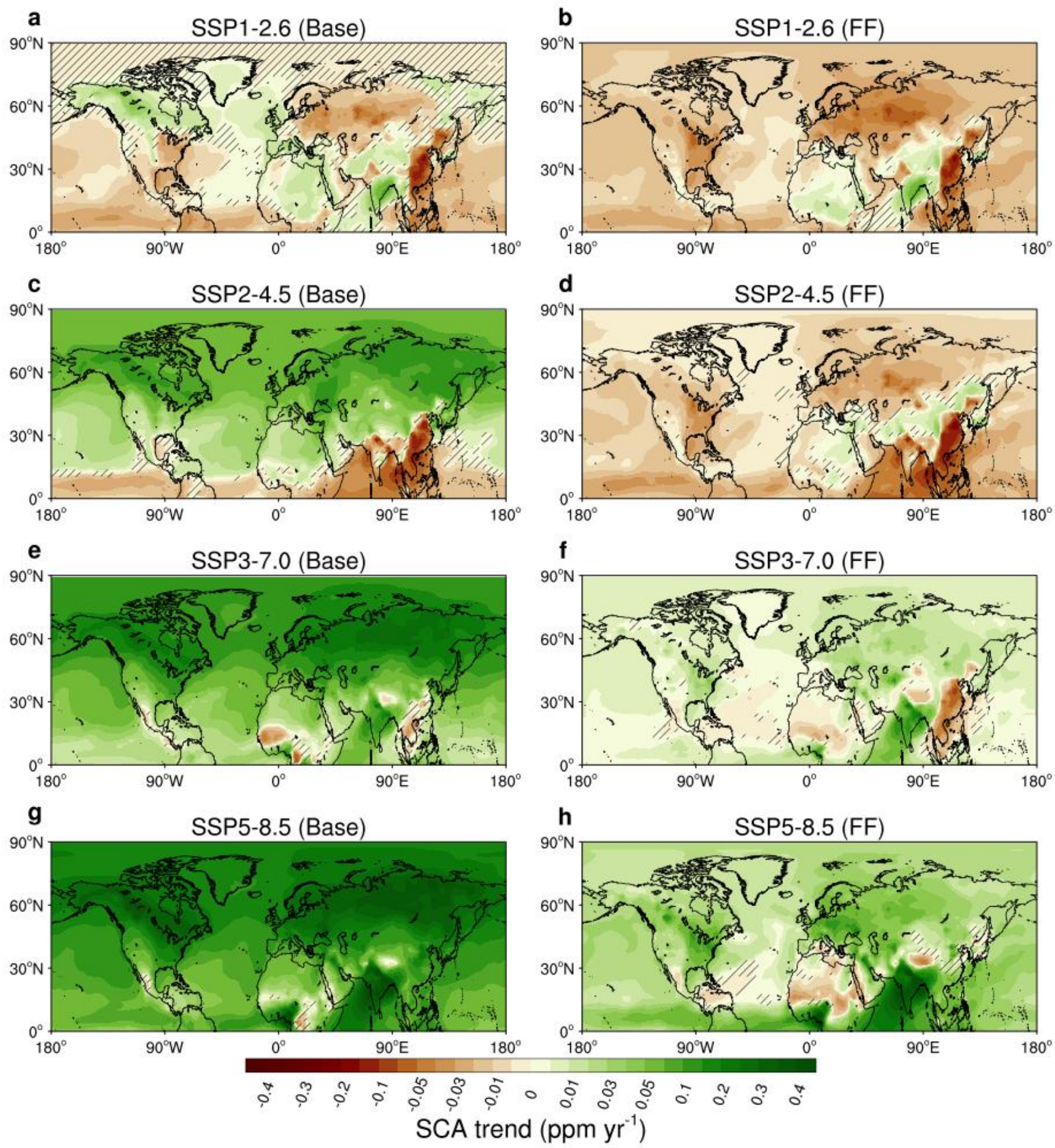

Supplementary Figure 19. Spatial patterns of SCA trends from 2030 to 2100 across the Northern Hemisphere, based on CMIP6_Base simulations (Base), and SCA trends attributable to fossil fuel CO_2 emissions (FF), estimated as the difference between CMIP6_Base and CMIP6_FF. Results are shown for the SSP1-2.6 (**a-b**), SSP2-4.5 (**c-d**), SSP3-7.0 (**e-f**), and SSP5-8.5 (**g-h**) scenarios. Hatched areas denote regions where trends are not statistically significant ($p > 0.05$).

(2) Revisions incorporated into the revised manuscript

a. Methods

We have added the relevant content to the Methods section in Lines 499-507 and 509-516:

"Future fossil fuel CO_2 emissions are provided from 2015 and at decadal intervals from 2020

to 2100, at a spatial resolution of 0.5° × 0.5° and with monthly temporal resolution[22,66]. These projections are generated by downscaling annual, sector-resolved national emissions from Integrated Assessment Models (IAMs) using 2014 country- and sector-specific spatial proxies, primarily sourced from EDGAR v4.2. Monthly variability in these gridded emissions is prescribed using spatially explicit seasonality fractions primarily derived from the ECLIPSE dataset, supplemented by EDGAR v4.3 for international shipping and by Lamarque et al. (2010)[67] for aviation."

"To evaluate the sensitivity of our results to uncertainties in the seasonality of fossil fuel CO_2 emissions, we constructed an alternative emission dataset. Monthly emission fractions averaged over 2017–2021 from the MEIC inventory were applied to the CMIP6 annual total at each grid cell (Supplementary Figs. 17, 18). The resulting emissions were used to drive transport simulations CMIP6_Base and CMIP6_FF under four future scenarios. The results confirm that the projected seasonal cycle metrics are robust to these differences in prescribed emission timing (Supplementary Fig. 19)."

b. Results

We have added the following content to the Results section in Lines 184-188.

"Across the northern temperate regions, decreasing SCA trends under the SSP1-2.6 scenario are mostly driven by fossil fuel CO_2 emissions (Fig. 3b). We confirmed that this conclusion is robust to the choice of emission seasonality (Supplementary Figs. 17-19) by running simulations using an alternative emission dataset with seasonality taken from historical MEIC inventory (see Methods)."

Reference

Feng, L. Y. et al. The generation of gridded emissions data for CMIP6. *Geosci. Model Dev.* **13**, 461-482 (2020).

Lamarque, J. F. et al. Historical (1850-2000) gridded anthropogenic and biomass burning emissions of reactive gases and aerosols: methodology and application. *Atmos. Chem. Phys.* **10**, 7017-7039 (2010).

(5) In SSP2-4.5, fossil fuel emissions (FFE) also decline, but the results do not consistently show that FFE dominates the decrease in SCA. Therefore, the statement in Lines 171–173 — "reduced fossil fuel emissions generally contribute to the decline in SCA" — appears too strong and should be toned down. In addition, the phrase in Lines 288–289 — "broadening from an ecological proxy to a critical metric for tracking global climate policy and its impacts" — seems somewhat exaggerated and would benefit from a more cautious expression.

Response #5:

We thank the reviewer for these suggestions. We have revised the manuscript to address both points with greater precision.

1. For the first point, we have refined the text to specify that this mechanism is primarily characteristic of the strong mitigation scenario SSP1-2.6, where FFE reduce rapidly. We have also added a clearer explanation of the mechanism, that is the synchronization between the seasonality of FFE emissions and atmospheric CO_2. The revised text is shown below in Lines 162-183:

"However, under the SSP1-2.6 scenario, a remarkable shift in the drivers of the SCA trend is observed (Fig. 3b). In boreal region, the contribution of terrestrial ecosystems declines substantially, and fossil fuel CO_2 emissions become the dominant driver of the SCA trend, particularly in Eurasia. The boreal terrestrial ecosystems exhibit a modest enhancement in carbon uptake from May to August (Supplementary Fig. 12), contributing to a slight increase in SCA (Supplementary Fig. 13). By contrast, the decline in fossil fuel CO_2 emissions exerts a much stronger influence, overwhelming the ecosystem-driven signal and propagating its impact into the broader European boreal region through atmospheric transport (Supplementary Figs. 10, 13). The mechanism underlying this shift involves the seasonal profile of fossil fuel emissions. At mid-high latitudes, fossil fuel emissions typically exhibit the seasonal pattern with higher emissions in winter and lower emissions in spring or summer[30-32], which is almost synchronous with the seasonality of atmospheric CO_2 (Supplementary Fig. 14). Consequently, the reduced fossil fuel emissions and dampened seasonality under SSP1-2.6 result in a substantial decrease in the atmospheric CO_2 SCA (Supplementary Figs. 13). At the BRW station,

the contribution of fossil fuel emissions to the variation in the CO_2 seasonal cycle increases substantially, with the fossil fuel-driven decrease in SCA largely offsetting the increase caused by the terrestrial biosphere (Supplementary Figs. 15, 16), resulting in no significant long-term trend in SCA. This implies that it will become more challenging to apply the SCA trend at BRW to track boreal terrestrial ecosystem carbon dynamics under the SSP1-2.6 scenario."

2. We have revised the Discussion section to offer a more cautious perspective. We removed the "critical metric" and instead framed the change as an "evolution" of the signal's utility. We also included the nuance regarding SSP2-4.5, where the fossil fuel signal emerges in post-2060 period to address the phenomenon that the trend is not immediately dominant in intermediate scenarios. The revised paragraph now reads as follows in Lines 281-294:

"As a result, the traditional use of the atmospheric CO_2 seasonal cycle as a proxy for plant activity will likely be compromised under future low-emission scenarios like SSP1-2.6. While this presents a challenge for studying ecosystem dynamics, it also suggests that the signal is evolving. As the biospheric signal weakens, the trends of CO_2 seasonal cycle will become increasingly influenced by fossil fuel emissions. This enhanced influence is not confined to the low-emission pathway like SSP1-2.6. Under the intermediate-emission scenario SSP2-4.5, the contribution from fossil fuel emissions increases after 2060, when emissions peak and subsequently decline sharply (Supplementary Fig. 8), thereby strengthening their imprint on the atmospheric CO_2 seasonal signals (Supplementary Fig. 23). Ultimately, the utility of atmospheric CO_2 seasonal cycle is not diminishing but evolving, transitioning from a proxy dominated by ecosystem fluxes toward an integrated metric capable of tracking anthropogenic emission pathways, particularly when fossil fuel emission reductions outpace changes in terrestrial ecosystem dynamics."

Some minors:

(1) Line 38, can also refers to "Zeng, N., Zhao, F., Collatz, G. J., Kalnay, E., Salawitch, R. J., West, T. O., & Guanter, L. (2014). Agricultural Green Revolution as a driver of increasing atmospheric CO_2 seasonal amplitude. Nature, 515(7527), 394-397. https://www.ncbi.nlm.nih.gov/pubmed/25409829"

Response #10:

We have cited Zeng et al. (2014) in Line 38 following the reviewer's suggestion.

(2) Line 379: CMIP5_FF -> CMIP6_FF

Response #11:

We have changed "CMIP5_FF" to "CMIP6_FF" as suggested in Line 418.

(3) In Supplementary Figure 2, it would be helpful to include some statistical metrics, such as RMSE or correlation coefficients, to quantitatively illustrate the performance of the GEOS-Chem simulations.

Response #12:

We thank the reviewer for this helpful suggestion. To provide a quantitative assessment of the performance of GEOS-Chem simulations, we have calculated the root mean square error (RMSE) and Pearson correlation coefficient between the observed and modelled (HIS_Base) CO_2 seasonal trends. The corresponding values are now included in each panel of Supplementary Fig. 2. The statistics show a high degree of consistency between simulated and observed trends for both SCA and SZC. As noted in the manuscript, the model exhibits larger biases in reproducing the AZC trend. The larger biases in AZC trends are consistent with the well-documented challenge in current ESMs regarding the accurate simulation of autumn phenology and carbon release processes (Piao et al., 2019; Richardson et al., 2012).

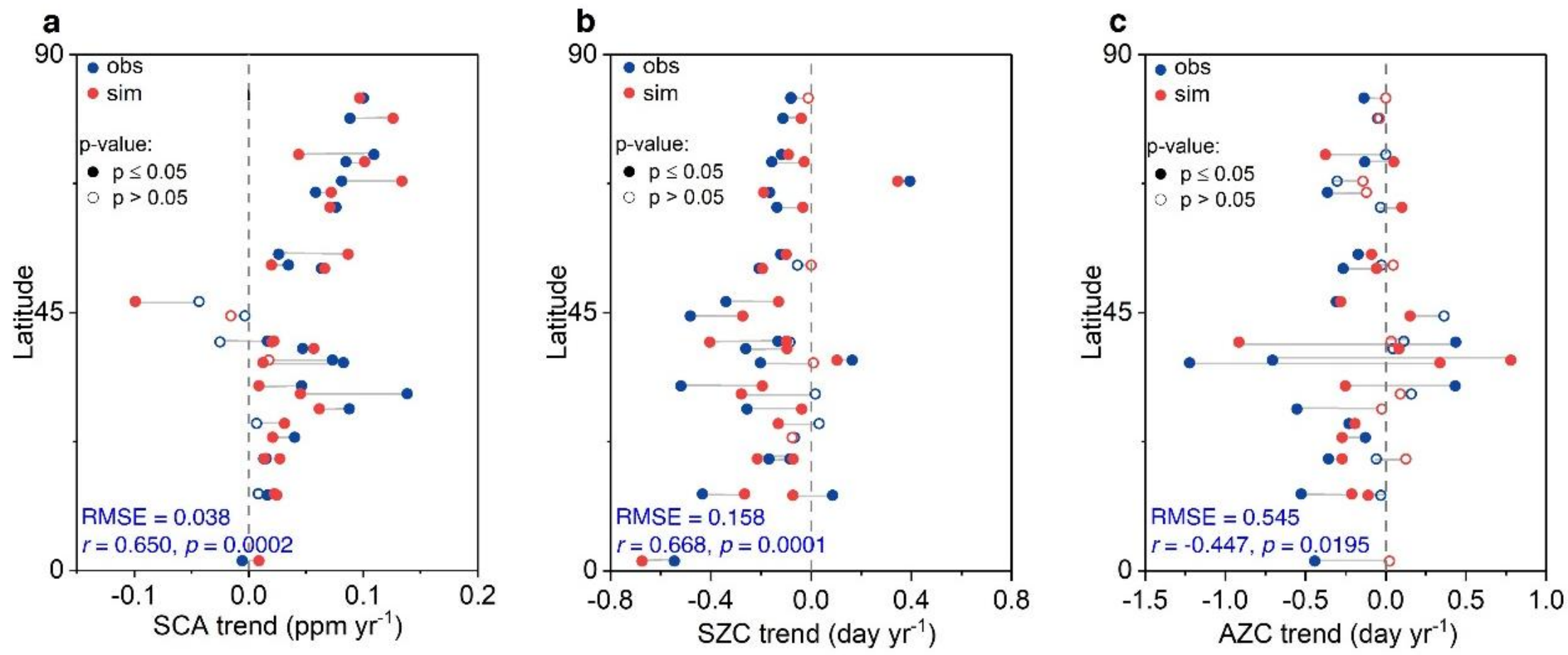

Supplementary Figure 2. Latitudinal distributions of observed and HIS_Base simulated trends

in SCA (a), SZC (b), and AZC (c) at 27 surface stations across the Northern Hemisphere during 1979 – 2021. For all scatters, the solid symbol indicates that it passes significance test ($p \leq 0.05$), while the hollow symbol indicates that it fails the significance test ($p > 0.05$). For each panel, the root mean square error (RMSE) between the observed and simulated values, together with the Pearson correlation coefficient and the corresponding significance test, is reported in the lower-left corner.

Reference

Piao, S. *et al.* Plant phenology and global climate change: Current progresses and challenges. *Glob. Change Biol.* **25**, 1922-1940 (2019).

Richardson, A. D. *et al.* Terrestrial biosphere models need better representation of vegetation phenology: results from the North American Carbon Program Site Synthesis. *Glob. Change Biol.* **18**, 566-584 (2012).

Reviewer #3 (Remarks to the Author):

This manuscript investigates how future emission scenarios affect the amplitude and phase of the atmospheric CO_2 seasonal cycle in the Northern Hemisphere, and identifies the key contributing fluxes (land biosphere, ocean, fossil fuel). The authors use GEOS-Chem simulations driven by state-of-the-art CMIP6 projections, providing valuable insights into a topic relevant to carbon-cycle research and emission monitoring. The main conclusions that terrestrial fluxes dominate seasonal-cycle amplification under high-emission scenarios, whereas reduced fossil fuel seasonality drives the seasonal amplitude decrease under strong mitigation (SSP1-2.6)—are important, novel, and challenge the conventional understanding linking seasonal cycles predominantly to terrestrial biosphere changes. This makes the study potentially significant to the broader atmospheric sciences and climate policy communities.

However, there are several methodological and interpretational issues that must be addressed before the manuscript can be considered suitable for publication. Thus, I recommend major revision, focusing on the following points:

Response:

We thank the reviewer for the encouraging comments and for recognizing the novelty and significance of our work, particularly regarding the mechanisms driving the CO_2 seasonal cycle under mitigation scenarios. We have carefully considered the reviewer's comments and conducted additional simulations (Response Table 1) to investigate the impacts of using different meteorological fields, terrestrial ecosystem CO_2 flux data, and fossil fuel CO_2 emission datasets on the attribution of CO_2 seasonal cycle. All these sensitivity tests show consistent results in terms of the SCA trend in future scenarios, strengthening the reliability and robustness of our conclusions. Below are the detailed point-to-point responses to your concerns and suggestions.

Response Table 1. Sensitivity experiments of future transport simulations under different scenarios driven by alternative meteorological fields, terrestrial CO_2 fluxes, and fossil fuel CO_2 emissions.

Experiment Type	Scenarios	Key inputs	Simulation Configuration	Total number of additional simulations
Meteorological uncertainty	SSP1-2.6, SSP2-4.5, SSP3-7.0, SSP5-8.5	Meteorological fields from 2 ESMs: CanESM5, NorESM2-LM	4 factorial simulations (CMIP6_Base, CMIP6_Land, CMIP6_FF, CMIP6_Ocean)	32 (4×2×4)
Terrestrial CO_2 flux uncertainty	SSP1-2.6, SSP2-4.5, SSP3-7.0, SSP5-8.5	Terrestrial CO_2 fluxes from 4 ESMs: ACCESS-ESM1-5, CanESM5, NorESM2-LM, MPI-ESM1-2-LR	2 factorial simulations (CMIP6_Base, CMIP6_Land)	32 (4×4×2)
Fossil fuel emission uncertainty	SSP1-2.6, SSP2-4.5, SSP3-7.0, SSP5-8.5	Fossil fuel emissions scaled by MEIC seasonal cycle	2 factorial simulations (CMIP6_Base, CMIP6_FF)	8 (4×2)

Major points:

1. Fixed future meteorology: The decision to use fixed 2021 meteorology for future scenario simulations isolates flux-driven changes but ignores potentially important atmospheric responses (e.g., boundary layer height, atmospheric circulation, mixing). This assumption likely influences amplitude and phase results. Please explicitly evaluate this assumption through sensitivity tests (e.g., alternative meteorological years or projected climate-model meteorology) to demonstrate that your findings are robust against this simplification.

Response #1:

We thank the reviewer for raising this critical point regarding the potential influence of future changes in meteorology. We note that Reviewer 2 raised a similar concern.

To address this comprehensively, we conducted a new suite of simulations driven by time-varying CMIP6 meteorological data. A detailed technical description of the methodology, model selection, and interpolation techniques is provided in our Response to Reviewer 2 (**Response #2b**). Below, we summarize these new analyses and key results.

1. Sensitivity tests and methodology: We systematically substituted MERRA-2 fields with CMIP6 counterparts and identified five key meteorological variables that substantially impact

CO_2 transport: surface pressure (ps), planetary boundary layer height (bldep), eastward wind (ua), northward wind (va), and specific humidity (hus). We then selected two representative CMIP6 models (CanESM5 and NorESM2-LM) that provided all necessary variables (Supplementary Tables 4, 5) and ran 32 new simulations (2 meteorological datasets × 4 SSP scenarios × 4 factorial experiments) driven by time-varying meteorological fields during 2030-2100.

2. Key results: We compared the CO_2 seasonal metrics (SCA, SZC, AZC) and their drivers between the constant-meteorology runs and the new time-varying meteorology runs. The trends in CO_2 seasonal metrics driven by time-varying CMIP6 meteorology are largely consistent with our original simulations using constant MERRA-2 fields (see Supplementary Figs. 33-36). Furthermore, the dominance of main drivers of CO_2 seasonality (e.g., fossil fuel CO_2 emissions in SSP1-2.6 and terrestrial ecosystems in SSP5-8.5) also remains unchanged compared with our original simulations (see Supplementary Figs. 37-44).

These results demonstrate that future variations in atmospheric circulation exert a much smaller effect on the projected trends in CO_2 seasonality compared to the magnitude of the flux changes. Consequently, our main conclusion remains robust: fossil fuel CO_2 emissions will dominate SCA trends under SSP1-2.6, whereas increasing land sinks continue to govern CO_2 seasonal variability under high-emission scenarios like SSP5-8.5.

Revisions incorporated into the revised manuscript

a. Methods

We have added a paragraph describing the sensitivity experiments and the use of CanESM5 and NorESM2-LM meteorological fields in Lines 420-426.

"To assess the potential impact of changing atmospheric circulation, we additionally performed a suite of sensitivity simulations driven by time-varying, scenario-specific meteorological fields from the CanESM5 and NorESM2-LM models (Supplementary Text 1). These tests confirm that the projected trends in CO_2 seasonality (Supplementary Figs. 33-36) and their attribution (Supplementary Figs. 37-44) are robust to the choice of meteorological forcing."

b. Supplementary Information

The full text of Supplementary Text 1, describing the sensitivity analysis methodology and results, is provided in Response to Reviewer 2 (**Response #2b**).

2. Representation of fossil fuel emission seasonality: Your key conclusion relies strongly on the projected reduction of fossil fuel emission seasonality derived from IAM scenarios. Future changes in heating methods, renewables penetration, or sectoral shifts may substantially alter these monthly emission patterns. Please provide at least one sensitivity experiment with alternative seasonal distributions (such as fixed 2030 patterns scaled by yearly totals or adjusted sectoral weighting) to confirm the robustness of your main conclusion. Additionally, explicitly quantifying fossil fuel seasonality indices and their correlation with atmospheric CO_2 seasonality would greatly strengthen your attribution.

Response #2:

We thank the reviewer for this insightful comment and agree that changes in the seasonal patterns of fossil fuel emissions (FFE) can substantially affect the trends in CO_2 seasonal metrics. Reviewer 2 raised a similar concern regarding the accuracy of IAM-based seasonality of FFE.

To address this point, we followed the reviewer's suggestion and performed a sensitivity experiment driven by FFE with an alternative seasonality. A detailed technical description is provided in our Response to Reviewer 2 (**Response #4**). Below, we summarize these new analyses and key results:

1. Sensitivity tests and methodology: We clarify that the CMIP6 emissions are derived from IAMs that explicitly simulate future sectoral transitions based on scenario-specific policy assumptions. Therefore, projected shifts in sectoral contributions are inherently captured within the original CMIP6 dataset. However, to rigorously test the robustness of our results against uncertainties in the seasonal distribution of these emissions, we constructed an alternative fossil fuel CO_2 emission dataset. We derived the historical monthly emission fractions from the high-resolution MEIC inventory (averaged over 2017-2021) and applied this fixed seasonality to the annual totals of the future CMIP6 scenarios at each model grid cell. This resulting seasonality

of fossil fuel emissions are quite different from that of the IAM projections especially for China (Supplementary Figs. 17, 18). We ran 8 new simulations (CMIP6_Base and CMIP6_FF under all 4 SSP scenarios) from 2030-2100.

2. The simulated trends in SCA remained consistent between the original CMIP6 simulations and the new MEIC-scaled simulations (Supplementary Fig. 19). By isolating the FFE contribution in the model, we confirmed that the dominance of FFE in driving the SCA decline under SSP1-2.6 is unchanged. The underlying reason it that the cumulative emissions during the accumulation period of atmospheric CO_2 (Nov-May) and drawdown period (May-Aug) remained comparable. Thus, the atmospheric CO_2 amplitude response was largely insensitive to the specific monthly redistribution.

3. To further explicitly quantify the relationship between fossil fuel emissions (FFE) and SCA under SSP1-2.6, we compared the time series of the amplitude of fossil fuel CO_2 emissions (FF amplitude) and the fossil fuel emission-caused SCA (FF caused SCA) under SSP1-2.6 for the period 2030-2100 at the BRW and MLO stations. The FF CO_2 emission amplitude is defined as the difference between the monthly maximum and minimum after removal of a quadratic trend. For BRW, the FF amplitude is derived by aggregating emissions over the boreal region, whereas for MLO it is derived from emissions aggregated over the Northern Temperate region. It can be seen that FF caused SCA is strongly correlated with FF amplitude (Response Fig. 1). A reduction in FF amplitude directly leads to a decrease in FF caused SCA, consistent with our statement in Lines 171-177.

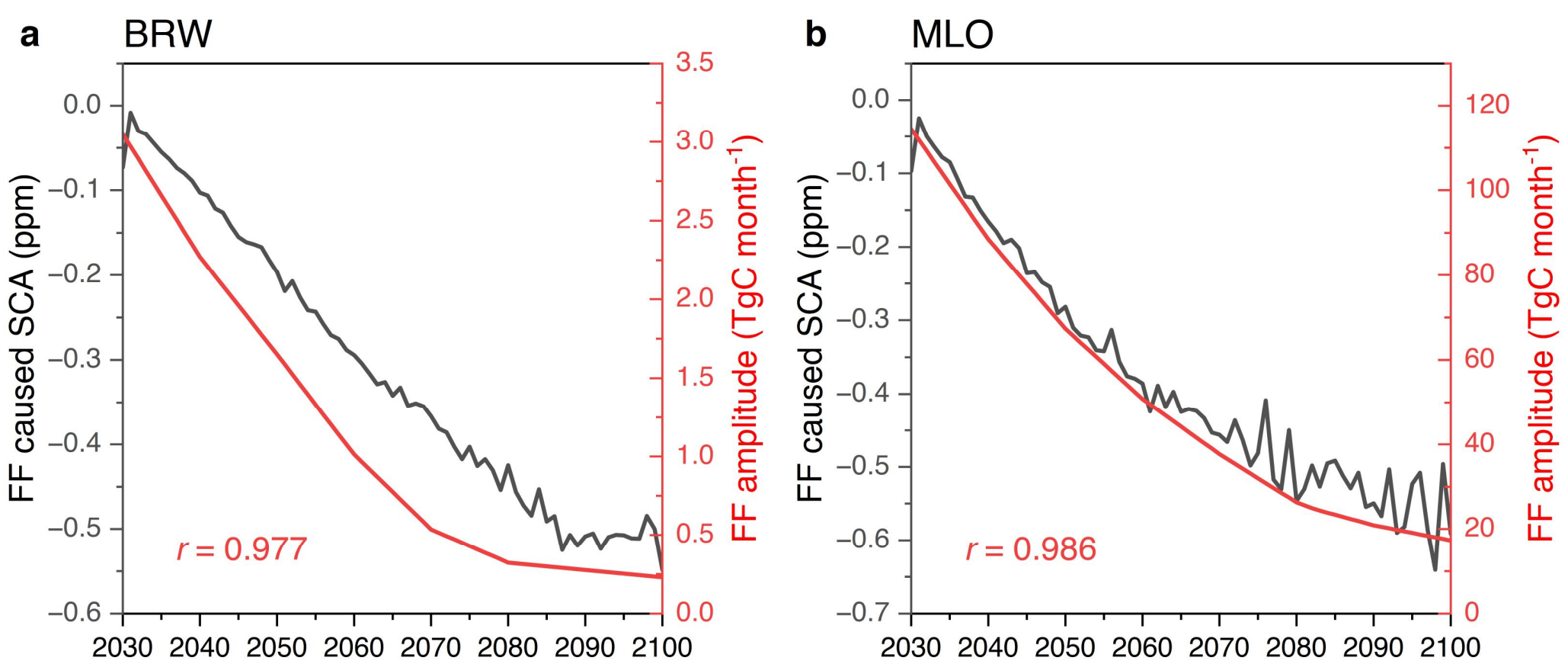

Response Figure 1. Time series of fossil fuel emission-caused SCA (FF caused SCA), and the amplitude of fossil fuel CO_2 emissions (FF amplitude) under SSP1-2.6 during 2030-2100 at the BRW and MLO stations. The fossil fuel CO_2 emission amplitude is defined as the difference between the monthly maximum and minimum after removal of a quadratic trend. For BRW, the FF amplitude is derived by aggregating emissions over the boreal region, whereas for MLO it is derived from emissions aggregated over the Northern Temperate region. Pearson correlations between SCA and FF amplitude (gray), and between FF caused SCA and FF amplitude (red), are shown in the lower-left corner of each panel.

Revisions incorporated into the revised manuscript

a. Methods

We have added the relevant content to the Methods section in Lines 499-507 and 509-516, as outlined below.

"Future fossil fuel CO_2 emissions are provided from 2015 and at decadal intervals from 2020 to 2100, at a spatial resolution of 0.5° × 0.5° and with monthly temporal resolution[22,66]. These projections are generated by downscaling annual, sector-resolved national emissions from Integrated Assessment Models (IAMs) using 2014 country- and sector-specific spatial proxies, primarily sourced from EDGAR v4.2. Monthly variability in these gridded emissions is prescribed using spatially explicit seasonality fractions primarily derived from the ECLIPSE dataset, supplemented by EDGAR v4.3 for international shipping and by Lamarque et al. (2010)[67] for aviation."

"To evaluate the sensitivity of our results to uncertainties in the seasonality of fossil fuel CO_2 emissions, we constructed an alternative emission dataset. Monthly emission fractions averaged over 2017–2021 from the MEIC inventory were applied to the CMIP6 annual total at each grid cell (Supplementary Figs. 17, 18). The resulting emissions were used to drive transport simulations CMIP6_Base and CMIP6_FF under four future scenarios. The results confirm that the projected seasonal cycle metrics are robust to these differences in prescribed emission timing (Supplementary Fig. 19)."

b. Results

We have added the following content to the Results section in Lines 184-188.

"Across the northern temperate regions, decreasing SCA trends under the SSP1-2.6 scenario are mostly driven by fossil fuel CO_2 emissions (Fig. 3b). We confirmed that this conclusion is robust to the choice of emission seasonality (Supplementary Figs. 17-19) by running simulations using an alternative emission dataset with seasonality taken from historical MEIC inventory (see Methods)."

3. Attribution method clarification: There is an inconsistency between methods (describing a vector projection approach) and the caption of Fig. 3 (implying contributions based on absolute trend magnitudes). Please clarify exactly how contributions are attributed and justify the chosen approach. If possible, showing both attribution methods would strengthen confidence in the robustness of conclusions.

Response #3:

We appreciate the reviewer for pointing out this issue. We apologize for the confusion. The original Methods section conflated two distinct attribution approaches. We have now clearly separated them.

1. To quantify the relative importance of each flux component in driving the long-term trends of seasonal metrics (SCA, SZC, AZC), we utilized the factorial simulation approach. We first calculated the linear trend of the SCA, SZC and AZC attributable to each flux component (Land, Ocean or Fossil Fuel) by differencing the baseline and factorial simulations (Lines 439-444). Since these trends can be positive or negative, they cannot be directly plotted on a ternary diagram, which requires non-negative components summing to unity. Therefore, we used the absolute value of each factorial trend normalized by the sum of absolute trends. This provides a measure of relative importance (i.e., which factor is the strongest driver of change), regardless of the direction of that change.

2. We used the vector projection method as a complementary analysis to quantify the contribution of each flux to the overall shape of the seasonal cycle in any given year. This method projects the detrended concentration onto the total seasonal cycle vector. This method is used to track how the dominant driver of the seasonal cycle itself evolves over time (e.g., in

Supplementary Fig. 16), but it is less suitable for attributing specific metric trends compared to the factorial method described above.

Revisions incorporated into the revised manuscript

a. Methods

We have revised the Methods section to clearly separate these two approaches in Lines 441-460.

"Taking the fossil fuel-driven SCA trend as an example, we subtracted the SCA time series of the CMIP6_FF from the CMIP6_Base. Fossil fuel emissions' contribution to the SCA trend was then defined as the linear trend of this difference time series, estimated using the Theil-Sen method. Since these factorial trends can be positive or negative, we quantified their relative importance using a ternary diagram. Because a ternary diagram requires all components to be non-negative and to sum to unity, we used the absolute value of each factorial trend, normalized by the sum of the absolute values of all three factorial trends, to quantify their relative importance (i.e., which factor is the strongest driver of change). As a complementary analysis, we applied a vector projection method[4] to quantify the relative contribution of each flux component to the overall shape of the annual CO_2 seasonal cycle pattern within one year. The contribution fraction of flux *i* is calculated as:

$$C_m = \sum_i C_{mi} \text{ and } x_i = \frac{\sum_m C_{mi} C_m}{\sum_m C_m^2} \quad (2)$$

where C_{mi} is the concentration in month *m* contributed by flux component *i* (where C_{mi} has been detrended and the annual mean removed, i.e. $\sum_m C_{mi} = 0$). C_m is the sum of the concentration from all flux components in month *m* and x_i is the contribution to the seasonal cycle from flux *i*, expressed as a fraction of the total cycle. Summing x_i over all fluxes adds to 1, but individual x_i can be negative. This approach yields an assessment of how the relative influence of each flux component on the CO_2 seasonal cycle within a year evolves over time."

b. Captions

We have revised the caption of Fig. 3 accordingly, as shown below.

“Figure 3. Relative contributions of net terrestrial CO_2 fluxes, fossil fuel CO_2 emissions, and air-sea CO_2 fluxes to trends in atmospheric CO_2 seasonal parameters across the Northern Hemisphere under historical and future scenarios. Relative contributions of three component fluxes to trends in CO_2 seasonal cycle amplitude (SCA; **a-c**), spring zero-crossing date (SZC; **d-f**), and autumn zero-crossing date (AZC; **g-i**) for the historical period (top row), SSP1-2.6 scenario (second row), and SSP5-8.5 scenario (bottom row). The ternary representation of the relative importance of the three component fluxes is based on the normalized fractional trends associated with each flux (see Methods). The locations of the BRW and MLO stations are indicated in each panel.”

We have also clarified in the caption of Supplementary Fig. 16 that the time series of contributions shown there were derived using the vector projection method, as revised below.

“Supplementary Figure 16. Time series of the factorial contributions of fossil fuel CO_2 emissions (FF), net terrestrial CO_2 fluxes (Land), and air-sea CO_2 fluxes (Ocean) to the atmospheric CO_2 seasonal cycle, derived using the vector projection method, at the BRW (**a-d**) and MLO (**e-h**) stations under the SSP1-2.6 (top row), SSP2-4.5 (second row), SSP3-7.0 (third row), and SSP5-8.5 (bottom row) scenarios.”

4. Propagation of land flux uncertainty: You use multi-model means for terrestrial fluxes without explicitly propagating model spread into atmospheric CO_2 metrics. Given significant inter-model differences in future terrestrial fluxes, providing uncertainty ranges for CO_2 seasonal amplitude and phase metrics based on terrestrial flux ensemble spread would enhance the credibility of your analysis.

Response #4:

We thank the reviewer for this helpful comment. Reviewer 2 raised a similar point. To address this, we conducted a new suite of transport simulations driven by individual model fluxes. A detailed technical description is provided in our response to Reviewer 2 (**Response #2c**). Below, we summarize these additional simulations and their results.

1. Methodology and model selection: Running the full 10-model ensemble for all scenarios was computationally demanding, we thus selected four representative ESMs, namely ACCESS-

ESM1-5, CanESM5, NorESM2-LM, and MPI-ESM1-2-LR. These models were chosen because they span the full range of uncertainty in NBP projections (Supplementary Fig. 24). We performed 32 simulations (4 carbon flux datasets × 4 scenarios × 2 factorial simulations, the latter corresponding to CMIP6_Base and CMIP6_Land) to explicitly propagate these flux uncertainties into the atmospheric CO_2 seasonal metrics.

2. Key results: We calculated the inter-model standard deviation for the projected SCA, SZC, and AZC trends (Supplementary Figs. 25-28). Inter-model spread widens under high-emission scenarios (e.g. SSP5-8.5), while the spread is much smaller under SSP1-2.6. We then produced a model agreement map (Supplementary Fig. 32) showing the number of models where the contribution of fossil fuel CO_2 emissions exceeds that of terrestrial fluxes under SSP1-2.6. This result confirms that across the four representative models, fossil fuel emissions remain the dominant driver of SCA decline in the Northern Hemisphere. This general consistency also applies for the dominance of terrestrial fluxes on the trends of seasonal metrics under high-emission scenarios.

Revisions incorporated into the revised manuscript

a. Methods

We have added the relevant content to the Methods section in Lines 517-530.

"For net terrestrial and air-sea CO_2 fluxes, we used the ensemble mean estimates from ten Earth system models (ESMs) participating in the Scenario Model Intercomparison Project (ScenarioMIP)[68] under four scenarios (Supplementary Table 3). The future net terrestrial and ocean CO_2 fluxes for 2030–2100 from different ESMs are provided at a monthly resolution, though their spatial resolution varies. All the data were resampled to the 2°latitude × 2.5°longitude resolution to obtain the ensemble mean fluxes. In addition, to quantify the uncertainty in future simulations of the atmospheric CO_2 seasonal cycle and its attribution arising from uncertainties in projected terrestrial CO_2 fluxes, we conducted transport simulations driven by terrestrial CO_2 fluxes from a representative subset of four ESMs: ACCESS-ESM1-5, CanESM5, NorESM2-LM and MPI-ESM1-2-LR. These models were selected because they collectively span the full range of NBP variability within the 10-model

ensemble (Supplementary Fig. 24). For each model, we performed baseline and LAND factorial simulations across all four future scenarios."

b. Discussion

We have added the following sentences to the Discussion section in Lines 305-316.

"We compared the simulated CO_2 seasonality driven by terrestrial carbon fluxes from four representative ESMs (ACCESS-ESM1-5, CanESM5, NorESM2-LM, and MPI-ESM1-2-LR) (Supplementary Figs. 25-28), selected to span the full range of terrestrial flux variability across the ten ESMs (Supplementary Fig. 24). Despite substantial inter-model differences under high-emission scenarios, all four models consistently produced small SCA trends under SSP1-2.6 (Supplementary Figs. 25, 26). More importantly, these trends are driven by fossil fuel emissions at most regions of the Northern Hemisphere (Supplementary Fig. 29, 32). This finding confirms that under a strong mitigation pathway like SSP1-2.6, the influence of the terrestrial fluxes on atmospheric CO_2 seasonality diminishes with high model agreement, leaving fossil fuel emissions the primary driver of the changing CO_2 seasonal cycle (Supplementary Fig. 29-32)."

5. Historical validation enhancement: You acknowledge weaker historical performance in simulating autumn zero-crossing dates. It would be helpful to provide a more quantitative evaluation across the entire observational network (e.g., RMSE, correlation statistics) and explicitly discuss whether this bias originates from terrestrial flux uncertainty, ocean fluxes, or atmospheric transport.

Response #5:

We thank the reviewer for this helpful suggestion. We agree that providing quantitative evaluation metrics is essential for establishing model credibility. We calculated the root mean square error (RMSE) and Pearson correlation coefficient between the observed CO_2 seasonal trends at the stations and those simulated by the baseline simulation (HIS_Base). The corresponding values are given in the lower-left corner of each panel in Supplementary Fig. 2. The model demonstrates high consistency with observations for SCA and SZC, characterized by high correlations, whereas it exhibits larger biases in reproducing the AZC trend.

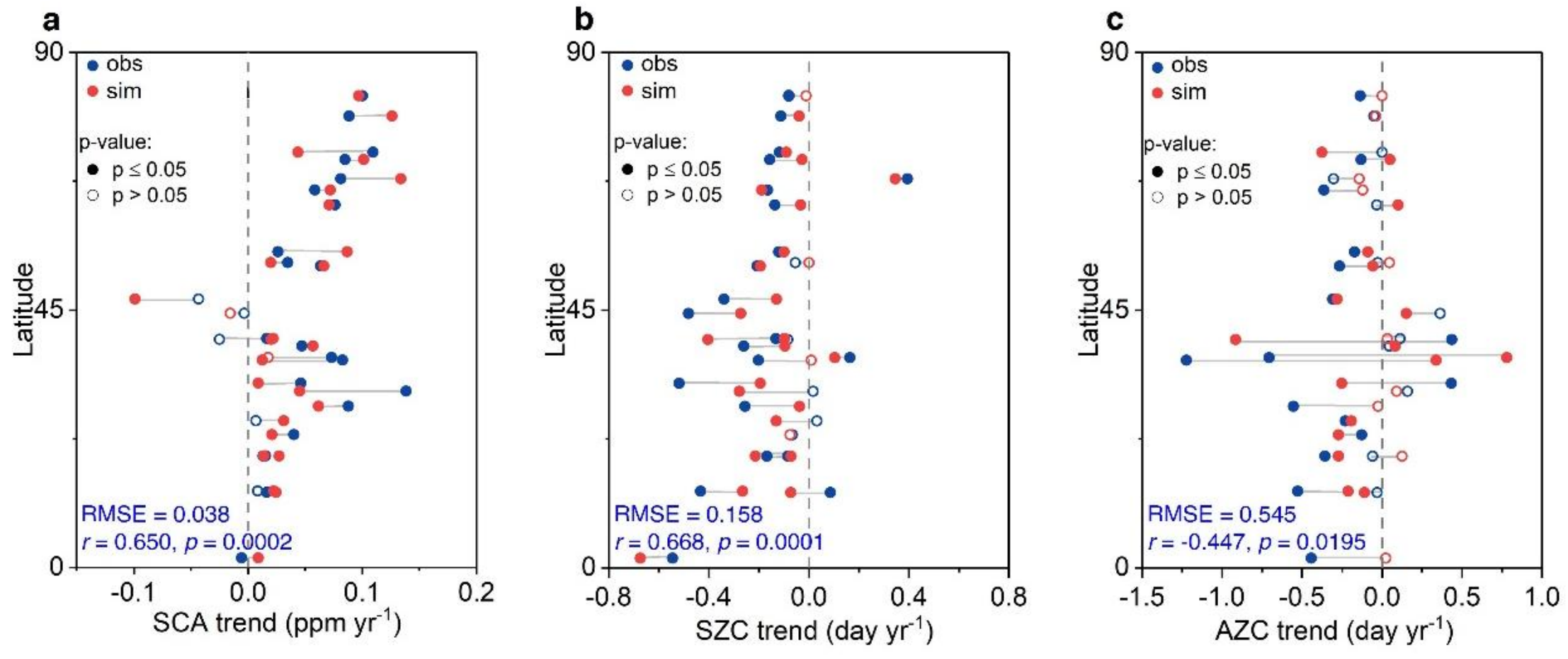

Supplementary Figure 2. Latitudinal distributions of observed and HIS_Base simulated trends in SCA (a), SZC (b), and AZC (c) at 27 surface stations across the Northern Hemisphere during 1979 – 2021. For all scatters, the solid symbol indicates that it passes significance test ($p \leqslant 0.05$), while the hollow symbol indicates that it fails the significance test ($p > 0.05$). For each panel, the root mean square error (RMSE) between the observed and simulated values, together with the Pearson correlation coefficient and the corresponding significance test, is reported in the lower-left corner.

To further investigate the sources of biases in the simulated AZC trends, we conducted a series of sensitivity experiments to quantify the respective roles of terrestrial CO_2 fluxes, ocean CO_2 fluxes, and atmospheric transport. We examined historical trends in global net biome exchange (NBE) simulated by 14 DGVMs from TRENDY v11. The NBE trends show very large inter-model spread, and we therefore selected JULES and LPJ, which produce the smallest and largest trends, respectively, to drive the CO_2 transport simulations (HIS_Base_JULES and HIS_Base_LPJ) (Response Fig. 2). Then, we analyzed ten ocean models from the Global Carbon Budget 2022. Since the spread of the trends in ocean carbon fluxes is much smaller than that for terrestrial fluxes, we selected the model MRI-ESM2-1, which yields the largest trend, to drive a third transport simulation (HIS_Base_MRI-ESM2-1) (Response Fig. 2). We also assessed the influence of atmospheric transport by driving the model with GEOS-FP meteorology (HIS_Base_GEOS-FP) instead of MERRA-2 used in the original baseline simulation (HIS_Base). As GEOS-FP data are available only from April 2012, we compared AZC time series for 2013-2021 from the GEOS-FP-driven simulations with those from

HIS_Base.

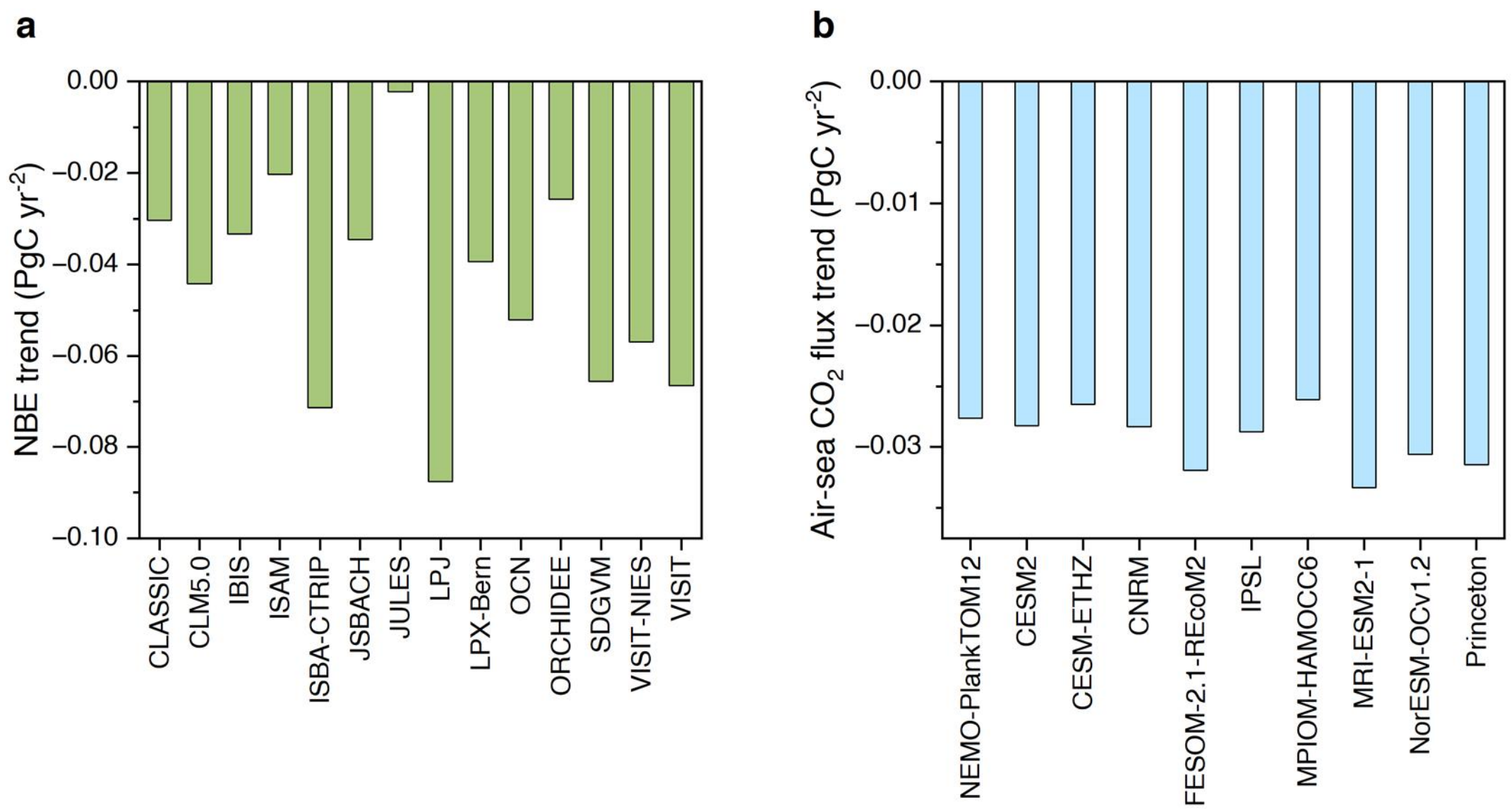

Response Figure 2. Trends in global net biome exchange (NBE) over 1979-2021 estimated by 14 DGVMs from the TRENDY v11 ensemble, and trends in air-sea CO_2 flux over the same period estimated by ten ocean models from the Global Carbon Budget 2022.

The AZC trends simulated by HIS_Base_JULES and HIS_Base_LPJ differ substantially from those of HIS_Base and from each other, indicating a high sensitivity to the representation of terrestrial fluxes (Supplementary Fig. 3). The difficulty of current ESMs in simulating autumn phenology was identified as the key sources of uncertainty (Piao et al., 2019; Richardson et al., 2012). In contrast, the AZC trends from HIS_Base_MRI-ESM2-1 and HIS_Base_GEOS-FP are very close to that from HIS_Base. These results indicate that the biases in simulated AZC trends are primarily attributable to uncertainties in terrestrial CO_2 flux estimates, rather than uncertainties in ocean CO_2 fluxes or atmospheric transport.

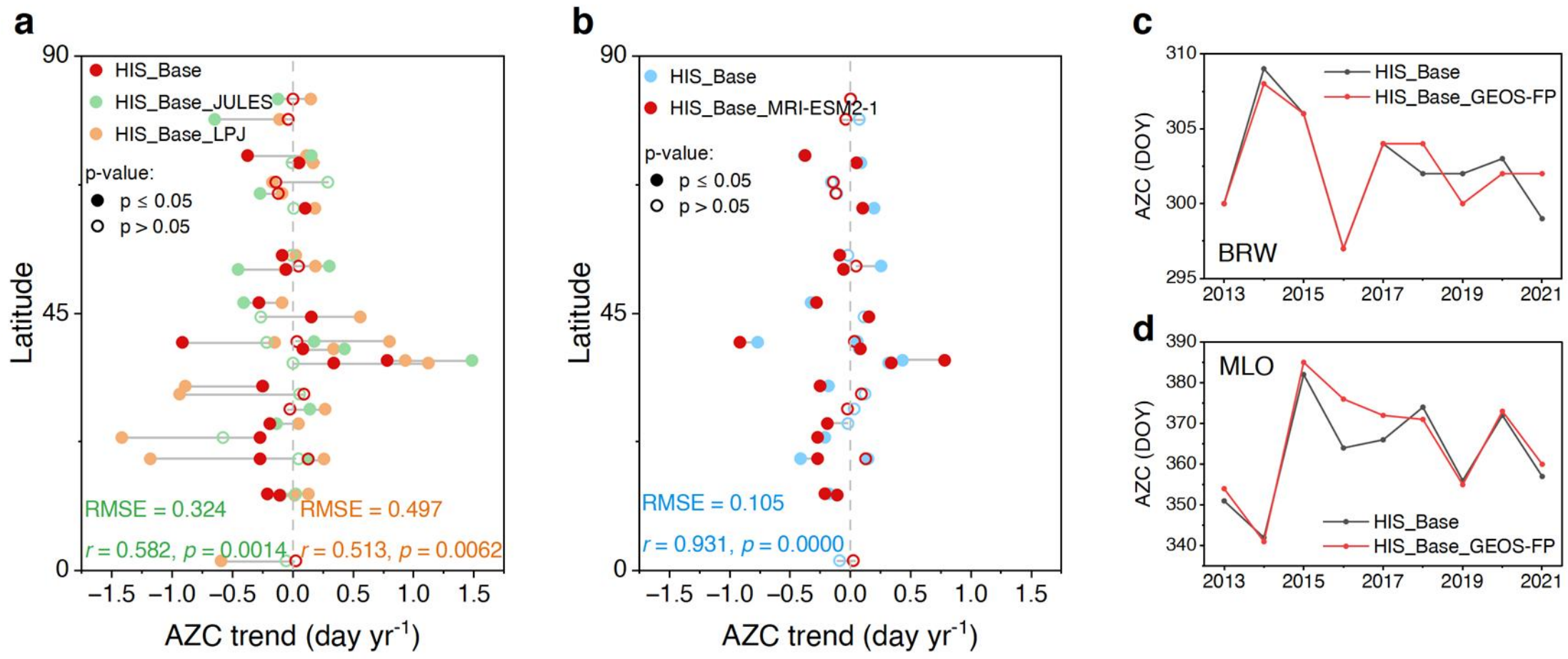

Supplementary Figure 3. Latitudinal distributions of simulated trends in AZC at 27 Northern Hemisphere surface stations during 1979-2021 (**a, b**), and time series of AZC at the BRW and MLO stations for 2013-2021 (**c, d**). Panel (a) shows the AZC trends from the baseline simulation (HIS_Base) and from sensitivity simulations in which NBE was replaced by that derived from the JULES (HIS_Base_JULES) or LPJ (HIS_Base_LPJ) DGVMs. Panel (b) shows the AZC trends from HIS_Base and from a sensitivity simulation in which the air-sea CO_2 flux was replaced by that from the MRI-ESM2-1 ocean model (HIS_Base_MRI-ESM2-1). Panels (c) and (d) show the 2013-2021 AZC time series at BRW and MLO, respectively, from HIS_Base and from a sensitivity simulation driven by GEOS-FP meteorological fields (HIS_Base_GEOS-FP). Because GEOS-FP meteorological fields are available only from April 2012 onward, the time series are shown starting in 2013. For all scatters, the solid symbol indicates that it passes the significance test ($p \leqslant 0.05$), while the hollow symbol indicates that it fails the significance test ($p > 0.05$). For panels (a) and (b), the root mean square error (RMSE) between the AZC trends from the baseline and sensitivity simulations, together with the Pearson correlation coefficients and the corresponding significance tests, are reported at the bottom of the figure.

Reference

Piao, S. et al. Plant phenology and global climate change: Current progresses and challenges. *Glob. Change Biol.* **25**, 1922-1940 (2019).

Richardson, A. D. et al. Terrestrial biosphere models need better representation of vegetation

phenology: results from the North American Carbon Program Site Synthesis. *Glob. Change Biol.* **18**, 566-584 (2012).

Smaller issues:

•Title: Consider explicitly stating "Northern Hemisphere" to clearly define the geographic scope of the study.

Response #7:

Following the reviewer's suggestion, we have revised the title to "Fossil fuel emissions dominate Northern Hemisphere CO_2 seasonal cycle trends under mitigation scenarios".

•Lines 160–161: Briefly clarify in which regions ocean flux contributions might still be relevant (e.g., subpolar North Atlantic or North Pacific).

Response #8:

We thank the reviewer for this comment. Our results demonstrated that while the influence of ocean CO_2 fluxes on the SCA trend remains relatively small under both historical period and future scenarios, their impacts on AZC trend become significant under high-emission scenarios. Specifically, under SSP3-7.0 and SSP5-8.5, ocean fluxes play a dominant role in advancing the AZC in the mid-latitudes of the North Pacific.

We have revised the text in the section "Attributions of trends in spring and autumn zero-crossing dates of atmospheric CO_2" (Lines 239-244) to clarify these regional contributions:

"In contrast, changes in AZC arise from the combined influences of variations in terrestrial ecosystems, fossil fuel emissions, and oceanic processes, with terrestrial ecosystems making the largest contribution overall (Fig. 3i). However, the relative influence of ocean carbon fluxes on the AZC trend intensifies under high-emission scenarios, particularly in the mid-latitudes of the North Pacific, where ocean processes dominate the advancement of AZC."

•Gini coefficient clarification (lines 326–331): Explain briefly that "11/12" is the theoretical maximum for 12 monthly bins to prevent confusion.

Response #9:

We thank the reviewer for raising this point. We have revised the text to explain that when the number of observations in each month is identical, Gini coefficient G reaches its minimum value of 0. G grows with increasing unevenness in the monthly distribution of observations, reaching its maximum when all observations are concentrated in a single month. Mathematically, if all observations occur in only one month, i.e. $x_1, x_2, \ldots, x_{11} = 0$ and $x_{12} = a$, the Gini coefficient is $G = 2 \times \frac{a \times 12}{12 \times a} - \frac{12+1}{12} = \frac{11}{12}$.

We have revised the explanation of Gini coefficient in Lines 345-348 to explicitly clarify its theoretical minimum and maximum values:

"Note that for one-year data aggregated into 12 months, the Gini coefficient ranges from 0 (perfect equality, identical observations every month) to a theoretical maximum of 11/12 (maximum inequality, all observations concentrated in a single month)."

•Line 369: Replace "real revolutions" with "real evolution".

Response #10:

We have revised the wording by replacing "real revolutions" with "real evolution" in Line 404.

•Line 379: "CMIP5_FF" should be corrected to "CMIP6_FF".

Response #11:

We have corrected the typo by changing "CMIP5_FF" to "CMIP6_FF" in Line 418.

•Lines 443–444: Verify that the Zenodo record number provided matches exactly with your submitted checklist.

Response #12:

We have updated the code link to ensure consistency with the submitted checklist. The revised link is: https://zenodo.org/records/17035880.

•Figure 1 (lines 617–631): Clarify explicitly that trends are computed using 11-year running means followed by Theil–Sen slopes, with significance tested using Mann–Kendall tests. Also, explicitly state units: SCA (ppm), SZC and AZC (day of year).

Response #13:

We thank the reviewer for the suggestion. We have revised the caption of Fig. 1 as follows, in Lines 723-739.

"**Figure 1. Variations in the seasonality of atmospheric CO_2 under historical and future scenarios at the Barrow, Alaska (BRW; 71.3° N, 156.6° W) and Mauna Loa, Hawaii (MLO; 19.5° N, 155.6° W) stations.** Detrended seasonal cycles of atmospheric CO_2 simulated for the historical period (1979, 2021) and future projections (2030, 2100) under the SSP1-2.6, SSP2-4.5, SSP3-7.0, and SSP5-8.5 scenarios at BRW (**a**) and MLO (**b**). Given the negligible inter-scenario differences in CO_2 seasonal cycles for 2030 (Supplementary Fig. 7), only the SSP1-2.6 result is shown as representative. Trends and changes in three CO_2 seasonal parameters, including the seasonal cycle amplitude (SCA, ppm; **c, d**), the spring zero-crossing date (SZC, day of year, DOY; **e, f**), and the autumn zero-crossing date (AZC, DOY; **g, h**), are presented at BRW and MLO, respectively. For the historical period, trends and changes in observed and simulated CO_2 seasonal cycle parameters are shown. Linear trends in SCA, SZC, and AZC were estimated from the 11-year running averages of their respective time series using the Theil-Sen method, and their significance was assessed with the Mann-Kendall non-parametric test. Significant trends ($p < 0.05$) are indicated by two dots, and marginally significant trends ($p < 0.1$) by a single dot, both placed above the bars. Values above the bars in (**c**) and (**d**) denote the percentage change in SCA over the period."

•Figure 3 (lines 640–656): Clearly specify the attribution methodology used here and ensure it is consistent with the description provided in Methods. If trends are used in attribution, clarify briefly: "Underlying trends computed as in Fig. 1."

Response #14:

We have provided a detailed description of the methods and data used to generate Fig. 3 in **Response #3**. Please refer to that response for the full details of the revisions made to the Methods section and the caption of Fig. 3.

•Supplementary typo (Supplement lines 39–41): Correct "SSP53-7.0" to "SSP3-7.0".

Response #15:

We have corrected the typo by changing “SSP53-7.0” to “SSP3-7.0” in Line 104 of the Supplementary file.

Reviewer #2 (Remarks to the Author):

General Comments:

The authors have clearly put in a substantial amount of work for this revision, evidenced by the 88 sensitivity simulations and the robust cross-validation using SSA. The transition to time-varying meteorological fields is a major step forward that effectively addresses the previous concerns over transport versus flux forcing. However, from a physical and consistency standpoint, I still find several critical gaps that need to be resolved, particularly regarding the disconnect between the new evidence presented in the supplements and the core narrative in the main text.

Response:

We appreciate the reviewer's positive evaluation of our revisions and the additional constructive comments. To address the reviewer's concerns, we conducted three additional sensitivity experiments focusing on the seasonality of fossil fuel emissions. The results from all these new experiments are consistent with those obtained using the original CMIP6 emissions, thereby reinforcing the robustness of our findings and improving the overall consistency of the manuscript.

1. While the authors revised the magnitude and seasonal profile of Chinese FF emissions in Supplementary Figures 17 and 18 to address previously noted biases (e.g., the Lunar New Year effect), it is not at all clear whether these corrections were integrated into the primary global results in the Main Text. If the core narrative and Figures 2 and 3 still rely on the original, biased CMIP6 inventory, the scientific integrity of the global attribution is compromised. Furthermore, for an assessment of CO_2 concentration responses under net-zero pathways in China, the authors can refer to the recent study by Tan et al. (2025, Science China Earth Sciences, 10.1007/s11430-024-1502-y). This regional simulation provides an ideal simulation for how local source changes impact CO_2 seasonality.

Response #1:

We thank the reviewer for this comment and agree that the representation of emission

seasonality is an important source of uncertainty. We clarify that Figs. 2 and 3 in the main text are based on simulations driven by the original fossil fuel (FF) emission data from CMIP6. Our decision to retain the original CMIP6 emissions in the main text is based on their role as a community-standard, internally consistent framework for representing future emission pathways across regions and sectors. Selectively modifying the seasonality for specific regions (e.g. China) or applying alternative seasonal profiles globally would result in a hybrid dataset that combines heterogeneous sources and assumptions, for which no validated framework currently exists for future projections. Utilizing the standard CMIP6 inventory therefore ensures our baseline results remain internally consistent and fully comparable with the broader CMIP6 literature.

To explicitly assess the impact of this uncertainty, we conducted additional sensitivity experiments using MEIC-scaled seasonal emissions (Supplementary Figs. 17 and 18). We note that the MEIC-derived profiles, while observationally constrained and sector-specific, reflect historical patterns from 2017–2021 and are extrapolated to future scenarios under the assumption of stationary seasonality. They are therefore presented here as a sensitivity test to bound the uncertainty associated with emission seasonality, rather than as a more accurate alternative inventory for future global emissions. In this revision, we further improved upon the previous implementation by applying sector-specific monthly profile from MEIC, rather than a single fixed monthly profile for total emissions (detailed below). Both the earlier MEIC-scaled experiment (Figs. R1, R2, applying a single monthly profile to total emissions) and this updated sector-specific version (now formally included as Supplementary Figs. 20 and 21) consistently show that the key results and conclusions presented in the main text are robust under both the CMIP6-based and MEIC-based seasonal profiles. Therefore, we consider the use of the original CMIP6 dataset in the main analysis to be both methodologically consistent and appropriate for global attribution purposes, while the sensitivity tests consistently confirm that our conclusions remain robust across alternative seasonal emission profiles.

We appreciate the suggestion to consider the study by Tan et al. (2025), which provides valuable insights into regional CO_2 seasonality under emission changes. We have now cited this work in the Introduction (lines 50-53): “Under ambitious climate mitigation scenarios, fossil fuel

emissions are projected to decline substantially, potentially altering their seasonal shapes and contribution to atmospheric CO_2 variability[16,22,23]." and the Discussion (lines 276-278): "These changes in fossil fuel emissions collectively contribute to the decrease of SCA globally, consistent with findings previously reported at the regional scale[23]."

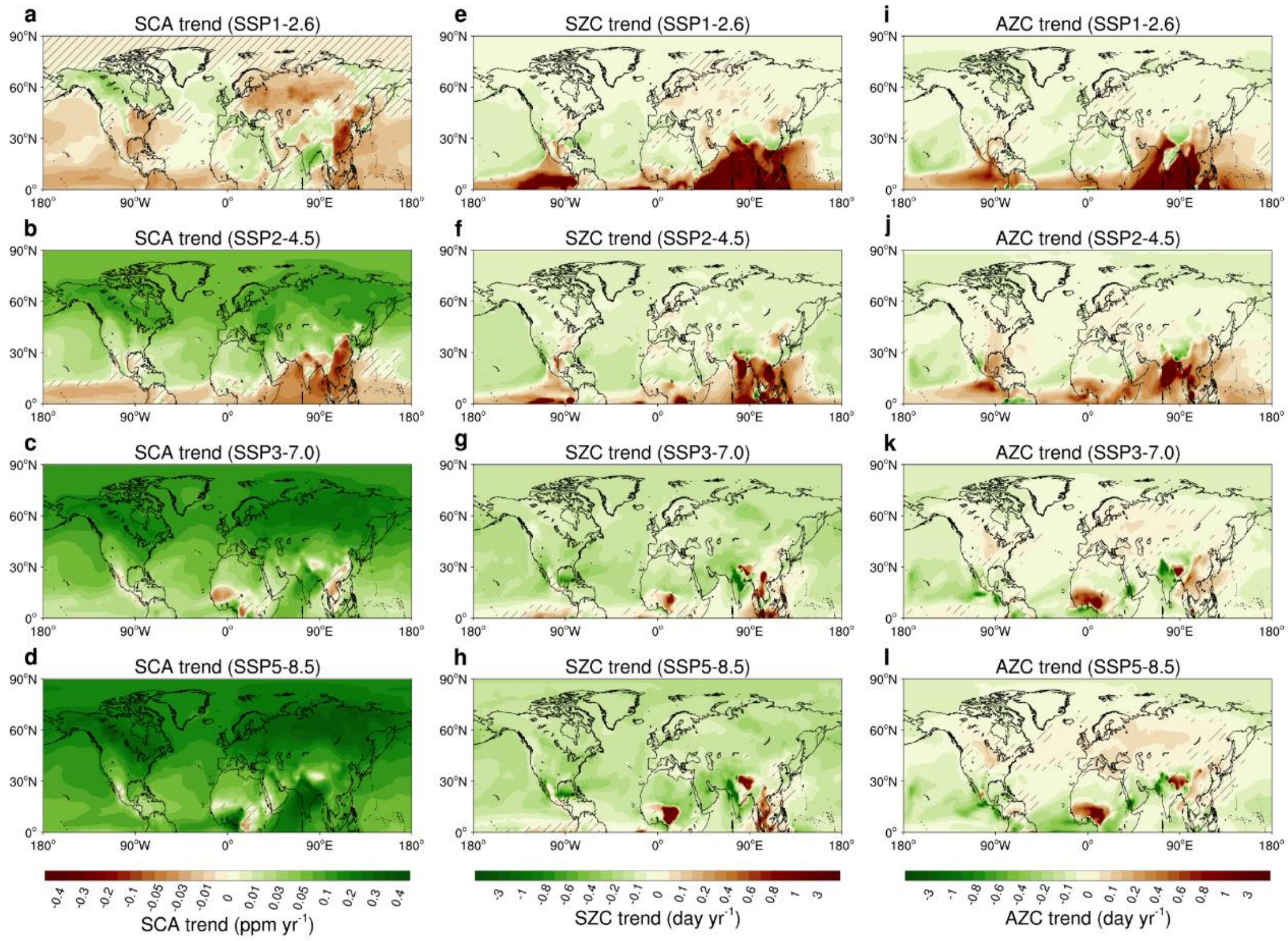

Figure R1. Spatial patterns of simulated trends in atmospheric CO_2 seasonal parameters across the Northern Hemisphere under four future scenarios. Fossil fuel CO_2 emissions in each scenario are derived by scaling CMIP6 monthly total emissions using monthly fractional contributions averaged over 2017-2021 from the MEIC inventory. Simulated trends in seasonal cycle amplitude (SCA; **a-d**), spring zero-crossing date (SZC; **e-h**), and autumn zero-crossing date (AZC; **i-l**) for the SSP1-2.6 (top row), SSP2-4.5 (second row), SSP3-7.0 (third row), and SSP5-8.5 scenarios (bottom row). Hatched areas denote regions where trends are not statistically significant ($p > 0.05$).

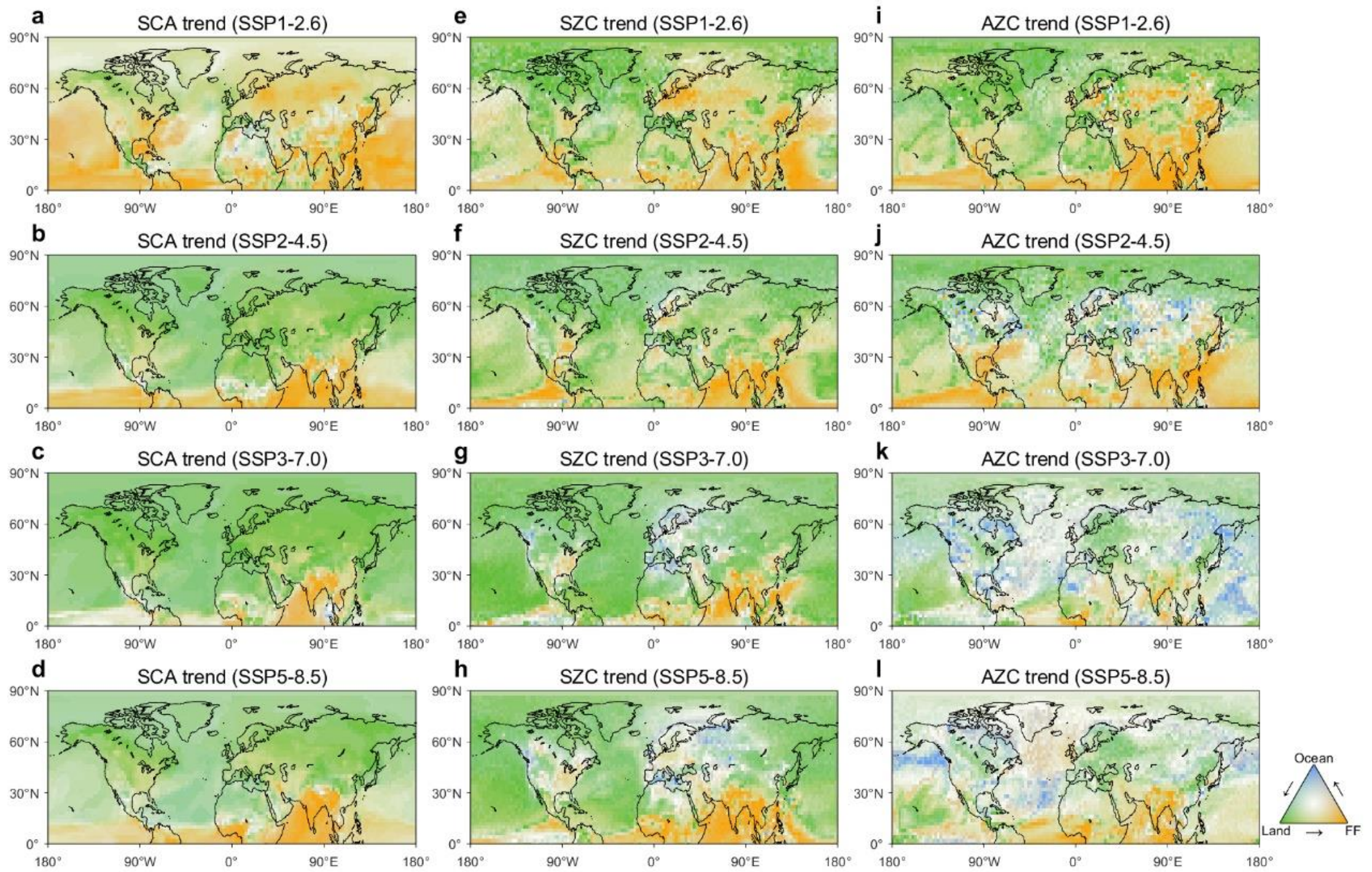

Figure R2. Relative proportions of net terrestrial CO_2 fluxes, fossil fuel CO_2 emissions, and air-sea CO_2 exchange contributing to trends in atmospheric CO_2 seasonal parameters across the Northern Hemisphere from 2030 to 2100 are shown under different future scenarios. Fossil fuel CO_2 emissions in each scenario are derived by scaling CMIP6 monthly total emissions using monthly fractional contributions averaged over 2017-2021 from the MEIC inventory. Panels display the relative proportions of the three component fluxes associated with trends in SCA (**a-d**), SZC (**e-h**), and AZC (**i-l**) for the SSP1-2.6 (top row), SSP2-4.5 (second row), SSP3-7.0 (third row), and SSP5-8.5 (bottom row) scenarios.

2. The study's conclusion that FF emissions dominate future SCA trends hinges on the assumption of a "fixed fractional profile" for emissions through 2100. Although the Discussion briefly acknowledges the "profound decarbonization transition" under SSP1-2.6, the current analysis fails to address the massive uncertainty introduced by the structural change of the emission "shape". In a deep mitigation future, electrification (e.g., heat pumps replacing boilers) and the dominance of intermittent renewables will fundamentally reshape the seasonal phase and timing of residual FF use, rather than merely reducing its absolute magnitude. The authors must significantly deepen the discussion on this point: specifically, how might a shift in the peak-emission month or a change in the winter-to-summer emission ratio in key regions (like

Eurasia) counteract the trend-driving effect of total magnitude reduction? Without a more rigorous exploration of this non-linear uncertainty, the claim that FF magnitude reduction is the sole driver remains overly idealized.

Response #2:

We thank the reviewer for this insightful comment on the potential impact of structural changes in emission seasonality under future mitigation scenarios. We agree that shifts in the seasonal phase and amplitude of fossil fuel (FF) emissions, beyond simple magnitude reduction, represent an important source of uncertainty.

First, we wish to clarify how emission seasonality is represented in CMIP6 and how it evolves under different scenarios. In CMIP6, fossil fuel emissions are first provided by integrated assessment models (IAMs) as annual sectoral emissions, whose relative fractions evolve over time under different scenarios (Supplementary Fig. 18a-d). These annual sectoral emissions are then disaggregated to monthly values using fixed, sector-specific seasonal profiles derived from the ECLIPSE dataset. As a result, while the seasonal profile of individual sectors is fixed, the seasonal cycle of total emissions is time-varying due to shifts in sectoral composition (Supplementary Fig. 18e-l). For example, under SSP1-2.6, substantial reductions in fossil fuel use, widespread deployment of renewable energy, and the deployment of negative-emissions technologies result in marked shifts in the seasonal phase and amplitude of total emissions (Supplementary Fig. 18a, e, i). On the contrary, other scenarios with continued increasing fossil fuel emissions exhibit more stable seasonal patterns.

We acknowledge that this framework assumes that sector-specific seasonal profiles remain unchanged in the future, and therefore does not explicitly capture potential structural shifts such as changes in peak-emission timing or winter-to-summer emission ratios driven by electrification or renewable energy variability. To our knowledge, however, no IAM currently provides monthly emission projections, making this approach the most consistent and tractable method for deriving monthly emissions within a scenario-based framework.

To directly address the reviewer's concern and assess the sensitivity of our conclusions to such structural uncertainties, we conducted three additional sets of experiments. First, we

constructed emissions using MEIC-derived sector-specific monthly fractions (2017-2021 mean), providing an observationally constrained alternative (Supplementary Fig. 19b, f). Second, we designed two idealized experiments to explicitly perturb the seasonal structure of emissions beyond historical patterns. In the CMIP6_uni2100 experiment, emission seasonality evolves toward a uniform distribution by 2100, removing seasonal cycle (Supplementary Fig. 19 c, g). In the CMIP6_rev2100 experiment, the seasonal cycle is progressively reversed relative to the 2030 baseline, representing an extreme shift in phase (Supplementary Fig. 19 d, h). While such a complete phase reversal is not physically expected, this idealized case is designed to provide an upper bound on how much restructuring of emission timing could in principle alter SCA trends, thereby bracketing the realistic range of changes that electrification or renewable penetration could induce.

Despite these substantial perturbations to emission timing, the simulated trends in CO_2 SCA (Supplementary Fig. 20) and the attribution of these trends remain consistent with those derived from the original CMIP6 emissions (Supplementary Fig. 21). This robustness holds across different future scenarios. Even under the extreme CMIP6_uni2100 and CMIP6_rev2100 experiments, both SCA trends and the dominant contribution of FF emissions remain largely unchanged (Supplementary Fig. 22).

These results indicate that, within the range of plausible and even highly idealized changes in emission seasonality, the projected decrease in FF emission magnitude remains the primary driver of future SCA trends under SSP1-2.6 scenario. Structural changes in emission timing, including shifts in seasonal phase or amplitude, exert a secondary influence and do not qualitatively alter our conclusions.

Following the reviewer's suggestion, we have strengthened the manuscript accordingly. Specifically, we now present revised simulations using MEIC-derived sector-specific monthly profiles, replacing the previous approach that applied a single monthly profile to total emissions. We have also expanded the Methods section (lines 521-536) to describe the construction of alternative emission datasets and clarified that these experiments are intended as uncertainty-bounding analyses rather than realistic projections:

“To evaluate the sensitivity of our results to uncertainties in the seasonality of fossil fuel CO_2 emissions, we constructed three alternative emission datasets (Supplementary Fig. 19). In the first case, sector-specific monthly profiles averaged over 2017–2021 from the MEIC global inventory were applied to the CMIP6 annual sector-specific emissions at each grid cell. We further designed two idealized experiments to explicitly perturb the seasonal structure of emissions beyond historically observed patterns. In one experiment, emission seasonality gradually transitions toward a uniform monthly distribution by 2100, thereby eliminating the seasonal cycle. In the other, emission seasonality is progressively reversed relative to the 2030 baseline, representing a pronounced phase shift. These three constructed future fossil fuel CO_2 emission datasets are intended primarily as an uncertainty-bounding analysis rather than as realistic projections of future emission patterns. The resulting emissions were used to drive transport simulations CMIP6_Base and CMIP6_FF under four future scenarios. The results confirm that the projected seasonal cycle metrics are robust to these differences in prescribed emission timing (Supplementary Figs. 20-22).”

In addition, the Discussion (lines 273-280) has been revised to explicitly acknowledge the potential for future structural changes in emission seasonality and to highlight the robustness of our conclusions to these uncertainties.

“The substantial reduction in fossil fuel CO_2 emissions, together with changes in their seasonal distribution, has altered the seasonal magnitude and phase of emissions, resulting in reduced differences between winter and summer emissions (Supplementary Figs. 18, 19). These changes in fossil fuel emissions collectively contribute to the decrease of SCA globally, consistent with findings previously reported at the regional scale[23]. Notably, this pattern remains robust even under scenarios in which the seasonality of fossil fuel emissions is substantially dampened or reversed (Supplementary Fig. 22).”

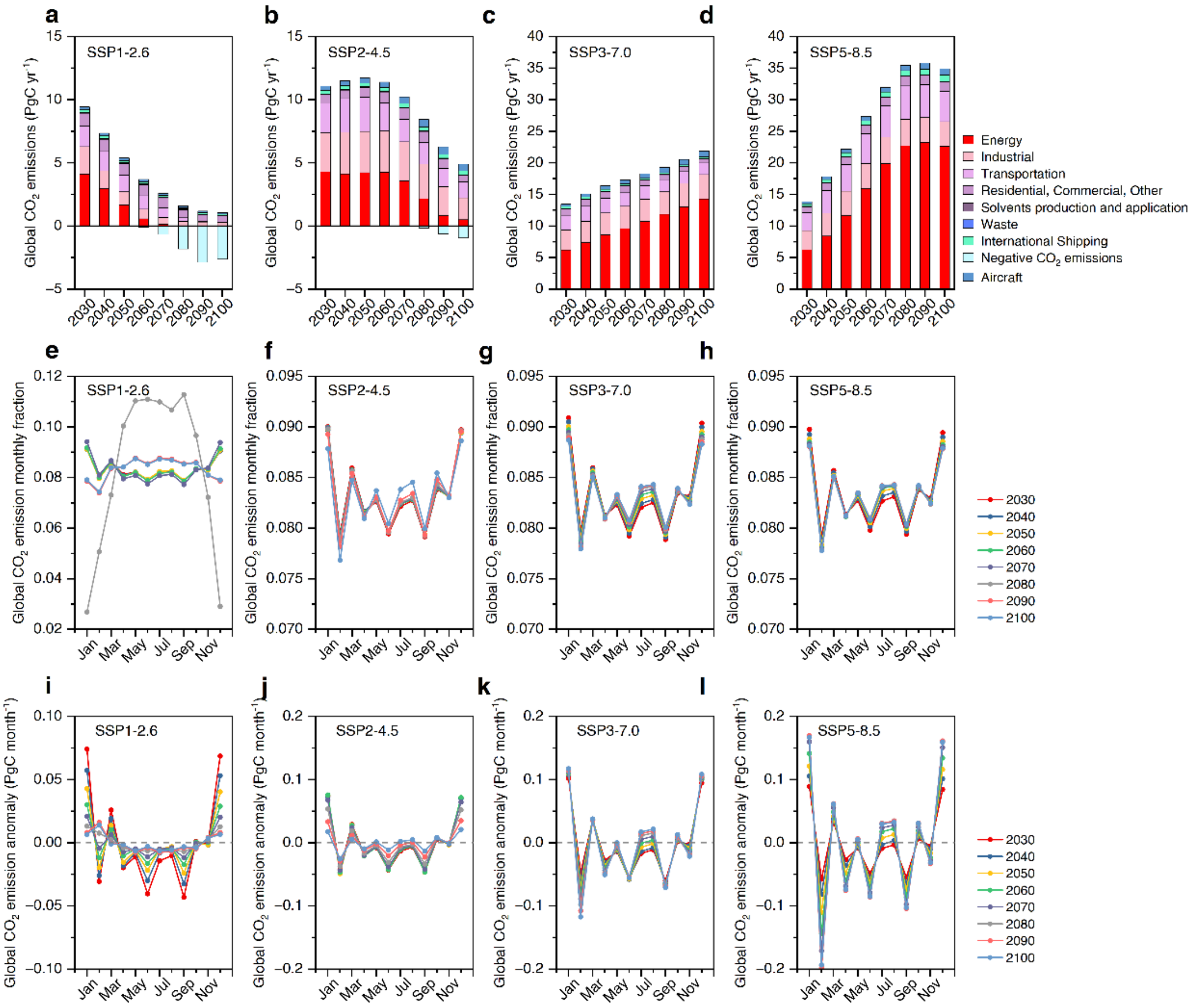

Supplementary Figure 18. Global CO_2 emissions by sector (**a-d**), monthly emission fractions (**e-h**), and monthly emission anomalies (**i-l**) under the SSP1-2.6 (first column), SSP2-4.5 (second column), SSP3-7.0 (third column) and SSP5-8.5 (fourth column) scenarios. All results are derived from the original CMIP6 projections.

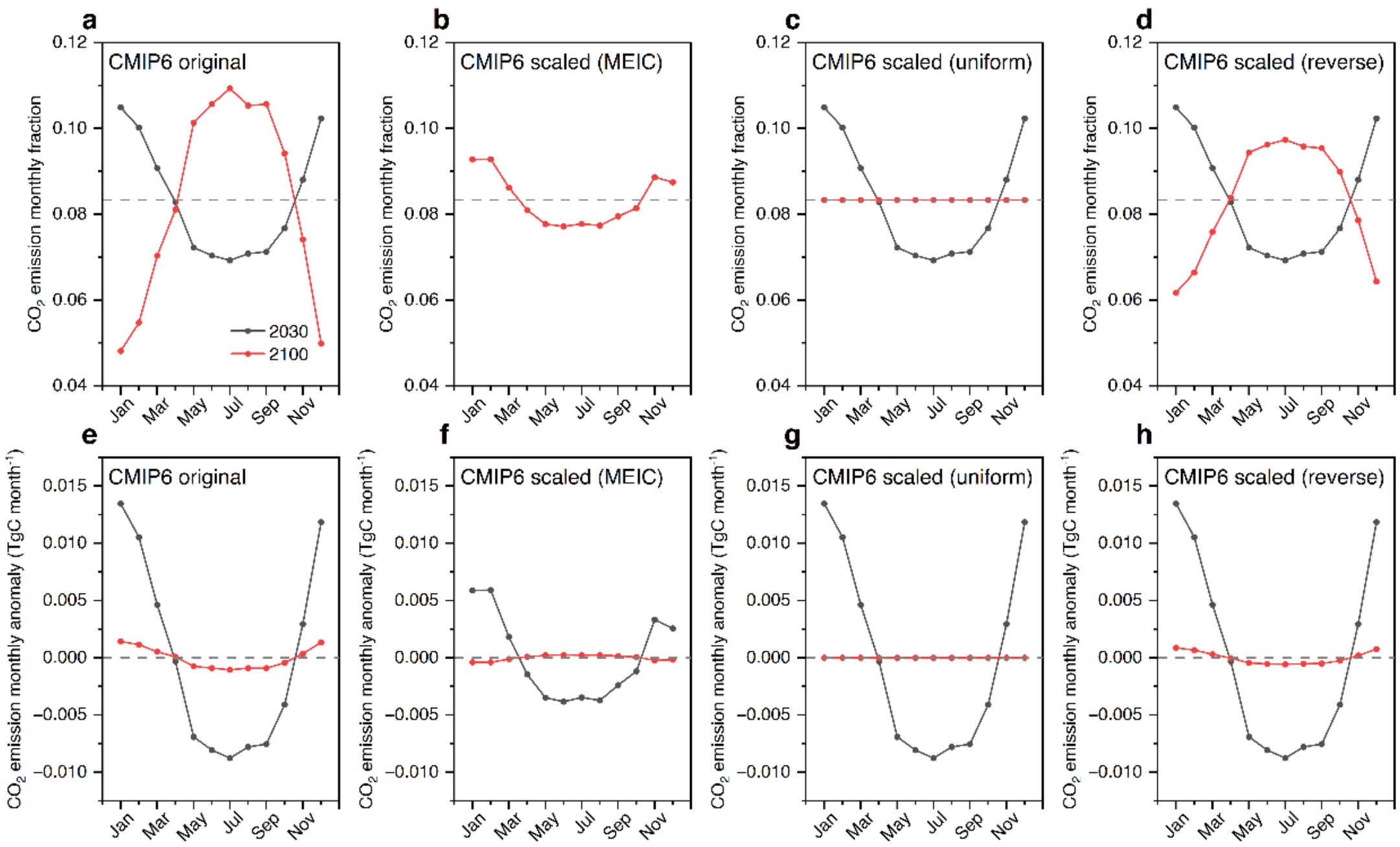

Supplementary Figure 19. Monthly CO_2 emission fractions (**a-d**) and anomalies (**e-h**) at a single grid cell (52.25°N, 8.25°E). Columns show results from the original CMIP6 projections (first column); CMIP6 projections scaled using 2017-2021 mean MEIC sector-specific seasonal fractions (second column); CMIP6 projections with seasonal fractions linearly transitioning from the 2030 distribution to a uniform distribution by 2100 (third column); and CMIP6 projections with seasonal fractions linearly transitioning from the 2030 distribution to a distribution that is mirror-symmetric about the annual mean by 2100 (fourth column).

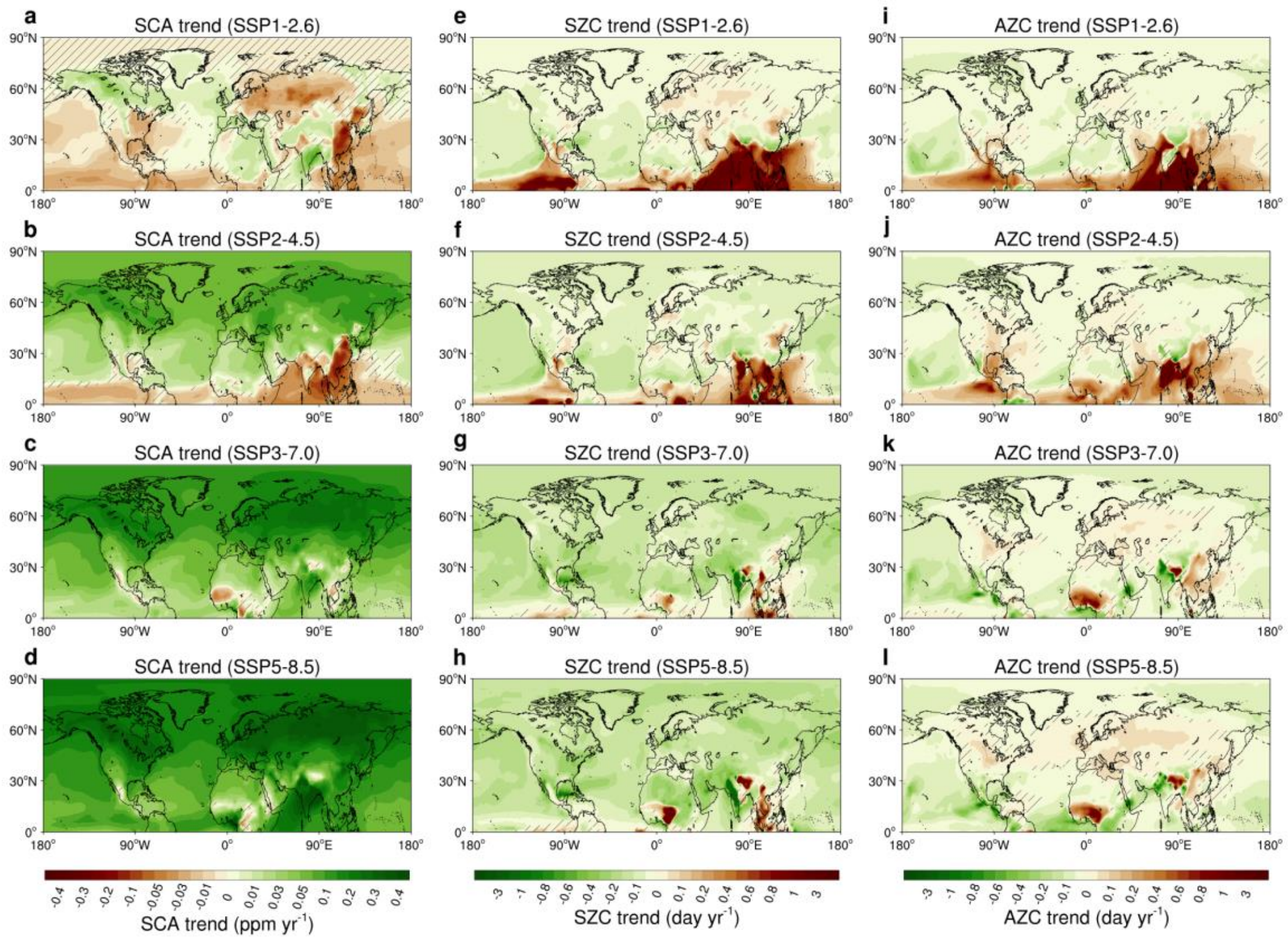

Supplementary Figure 20. Spatial patterns of simulated trends in atmospheric CO_2 seasonal parameters across the Northern Hemisphere under four future scenarios. Fossil fuel CO_2 emissions in each scenario are derived by scaling CMIP6 sectoral monthly emissions using sector-specific monthly fractional contributions averaged over 2017-2021 from the MEIC inventory. Simulated trends in seasonal cycle amplitude (SCA; **a-d**), spring zero-crossing date (SZC; **e-h**), and autumn zero-crossing date (AZC; **i-l**) for the SSP1-2.6 (top row), SSP2-4.5 (second row), SSP3-7.0 (third row), and SSP5-8.5 scenarios (bottom row). Hatched areas denote regions where trends are not statistically significant ($p > 0.05$).

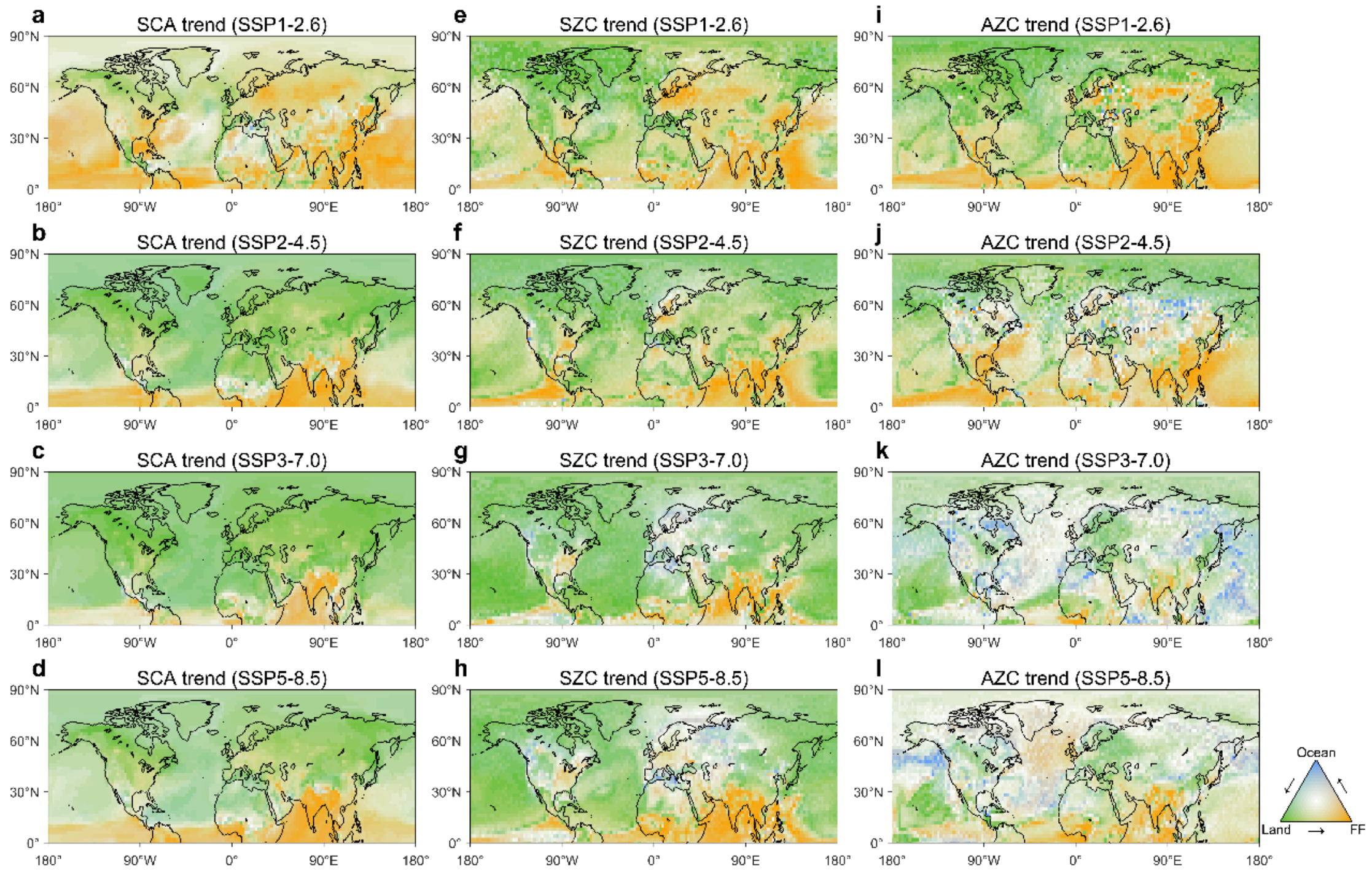

Supplementary Figure 21. Relative proportions of net terrestrial CO_2 fluxes, fossil fuel CO_2 emissions, and air-sea CO_2 exchange contributing to trends in atmospheric CO_2 seasonal parameters across the Northern Hemisphere from 2030 to 2100 are shown under different future scenarios. Fossil fuel CO_2 emissions in each scenario are derived by scaling CMIP6 sectoral monthly emissions using sector-specific monthly fractional contributions averaged over 2017-2021 from the MEIC inventory. Panels display the relative proportions of the three component fluxes associated with trends in SCA (**a-d**), SZC (**e-h**), and AZC (**i-l**) for the SSP1-2.6 (top row), SSP2-4.5 (second row), SSP3-7.0 (third row), and SSP5-8.5 (bottom row) scenarios.

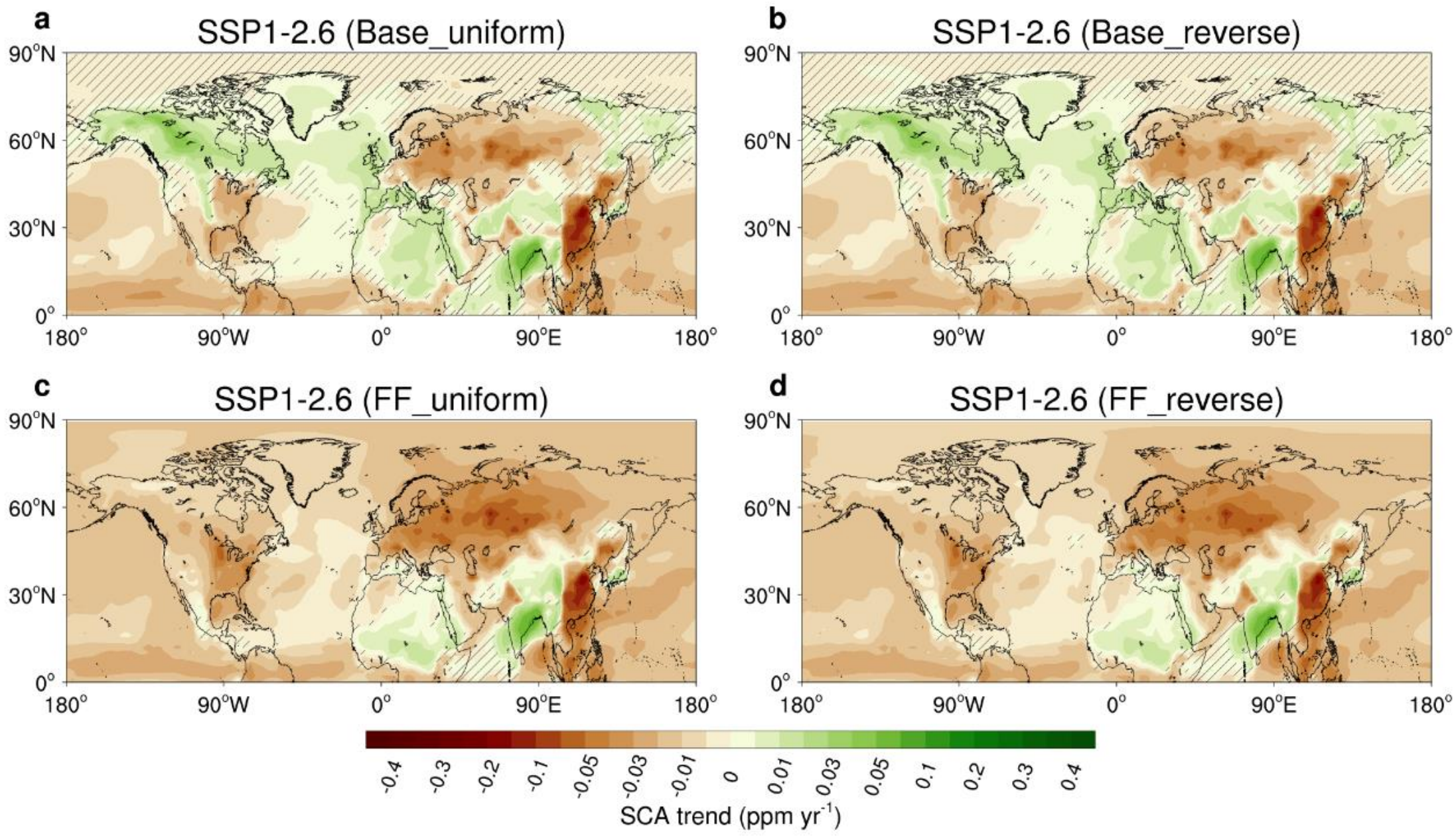

Supplementary Figure 22. Spatial patterns of SCA trends across the Northern Hemisphere during 2030-2100, derived from CMIP6_Base simulations (**a, b**), and the SCA trends attributable to fossil fuel CO_2 emissions (FF), estimated as the difference between CMIP6_Base and CMIP6_FF (**c, d**). In the first column, simulations are based on a rescaled seasonal distribution of CMIP6 FF emissions, in which the monthly fractions are progressively adjusted from 2030 onwards to converge to a uniform distribution by 2100. In the second column, simulations are based on an alternative rescaling of the seasonal emission distribution, where the monthly fractions are gradually modified from 2030 to achieve, by 2100, a reversed (mirror-symmetric with respect to the annual mean) configuration of the 2030 distribution. Hatched areas denote regions where trends are not statistically significant ($p > 0.05$).

3. I noted that Supplementary Figure 17 is missing any citation in the main text. Additionally, given the lower performance in simulating the Autumn Zero-Crossing (AZC) trend compared to SCA (in the validation), the authors should use much more cautious language in the Abstract and Conclusion. Over-stating the confidence in autumn parameters is not advisable when the models themselves still struggle to capture the basic phenological timing of the carbon cycle during the senescence phase.

Response #3:

We thank the reviewer for flagging this. Upon re-checking, we found that Supplementary Fig.

17 was cited in the original submission (line 186) and is now cited at line 188 in the revised manuscript. We also acknowledge the relatively lower performance in simulating the AZC trend compared with SCA, as discussed in the Results section (lines 88-90). We fully agree that the uncertainties in AZC simulations warrant more explicit communication. Accordingly, we have revised the wording throughout the manuscript in two ways: first, we added a clarifying statement to emphasize the greater uncertainty in AZC-related results (lines 90-92); second, we adopted more cautious wording throughout the presentation of the AZC-related findings (lines 140-149):

"Given the limitation in the simulation of AZC, the corresponding trends should be interpreted with caution and are presented here as indicative rather than definitive."

"In contrast, the AZC trend in our simulation shows an opposite pattern across scenarios compared to that of SZC. From SSP1-2.6 to SSP5-8.5, AZC in eastern North America and central-western Europe may gradually shift from advancing to delaying (Fig. 2h, i). However, at both BRW and MLO, AZC is projected to continue to advance under all four scenarios. Under SSP1-2.6, AZC may advance at rates of approximately 0.09 day yr^{-1} at BRW and 0.15 day yr^{-1} at MLO (both $p < 0.05$), while under SSP5-8.5, the corresponding rates are 0.10 day yr^{-1} at both stations (both $p < 0.05$) (Fig. 1g, h). This indicates that, under high-emission scenarios, the AZC trends at BRW and MLO stations may become different from those across the broader boreal and northern temperate regions."

Also, the language in the Abstract and Discussion sections has been further refined (lines 24-28 and lines 252-255):

"Under the high-emission scenario (SSP5-8.5), increasing land sinks are projected to dominate and amplify CO_2 seasonal variability. In contrast, under the low-emission scenario (SSP1-2.6), a widespread decline in CO_2 seasonal amplitude is largely driven by changes in fossil fuel emissions rather than land sinks."

"Our study reveals that under the SSP5-8.5 scenario, terrestrial ecosystems are projected to remain the primary contributor to CO_2 seasonal variations, leading to a larger amplification in SCA and a more substantial advancement in SZC compared to the historical period."

Reviewer #3 (Remarks to the Author):

Recommendation: Minor revision.

The manuscript has improved following this revision. The additional sensitivity analyses on fossil-fuel emission seasonality, time-varying meteorology, and uncertainty propagation from land-model differences provide stronger support for your primary conclusion: under strong mitigation scenarios, fossil-fuel emission patterns (both total emission reductions and smaller winter–summer contrasts) could become the main determinant of future Northern Hemisphere CO_2 seasonal-cycle amplitude trends, rather than terrestrial fluxes.

Response:

We thank the reviewer for the positive assessment of our revisions and for the additional detailed suggestions. In response, we have provided a more comprehensive description and validation of the methods and refined the language to improve clarity and accuracy.

Major points:

1. The revised manuscript clearly states that the ternary plots indicate relative importance based on absolute factorial trends (p.17, lines 439–449), which is appropriate. However, the use of absolute values means the direction (positive or negative contributions) is not explicitly visible. It would be helpful if the Fig. 3 caption or associated text clearly stated that the ternary plots reflect only the magnitude of each factor's influence, while the direction (increase/decrease) can be found separately in the detailed maps (e.g., Supplementary Fig. 13).

Response #1:

We thank the reviewer for this helpful suggestion. We have added clarifying statements in the Methods section (lines 460-462) and in the caption of Fig. 3 (lines 786-790):

"The signed magnitude (increase or decrease) of each factorial trend is provided separately in the corresponding maps (e.g., Supplementary Figs. 13, 23 and 24)."

"The ternary representation of the relative importance of the three component fluxes is based on the normalized absolute factorial trends associated with each flux (see Methods). The

direction (sign) and spatial distribution of each factorial trend are presented separately in the corresponding maps (Supplementary Figs. 13, 23 and 24)."

2. The analysis employs an 11-year running mean before estimating trends and conducting Mann–Kendall significance tests (p.15, lines 378–381; Fig. 1 caption, p.27, lines 735–737). While this smoothing approach is valid, it introduces serial correlation that can affect the accuracy of statistical significance tests. Please briefly clarify whether autocorrelation was explicitly considered or corrected for, or confirm via a simple sensitivity check using unsmoothed annual data. A brief sentence on this would strengthen confidence in the statistical results.

Response #2:

We thank the reviewer for this helpful suggestion. We followed the reviewer's suggestion and performed a sensitivity analysis using the unsmoothed time series of CO_2 seasonal parameters to estimate trends and their statistical significance. The resulting trends in SCA, SZC, and AZC at the BRW and MLO stations using unsmoothed data are shown in Supplementary Fig. 36, and the spatial distributions of these trends under both historical and future scenarios are shown in Supplementary Fig. 37. Overall, the use of an 11-year running mean has only a limited influence on the magnitude of the estimated trends.

Across both the BRW and MLO stations and across Northern Hemisphere spatial patterns, applying a running mean to the CO_2 seasonal parameter time series primarily affects the statistical significance of trends during the historical period, with the effect largely confined to SZC and AZC, and having only a limited influence on trend magnitudes. Owing to the relatively short historical period (1979-2021; 43 years) and the large variability in SZC and AZC (Supplementary Fig. 4), trends estimated from the original time series are less statistically significant than those derived from the smoothed series. By contrast, for the future scenarios, which are the primary focus of this study, the impact of smoothing on both the magnitude and significance of trends in all three parameters is negligible across all three metrics. This reflects the longer time span (2030-2100; 71 years) and stronger underlying signals in the projected changes.

We have added the following content to the Methods section (lines 387-392):

“We additionally performed a sensitivity analysis using the original (unsmoothed) time series of CO_2 seasonal parameters to estimate trends and their statistical significance. Overall, the application of a running mean has little influence on trend estimates, particularly for the long-term future scenarios that are the focus of this study (Supplementary Figs. 36, 37).”

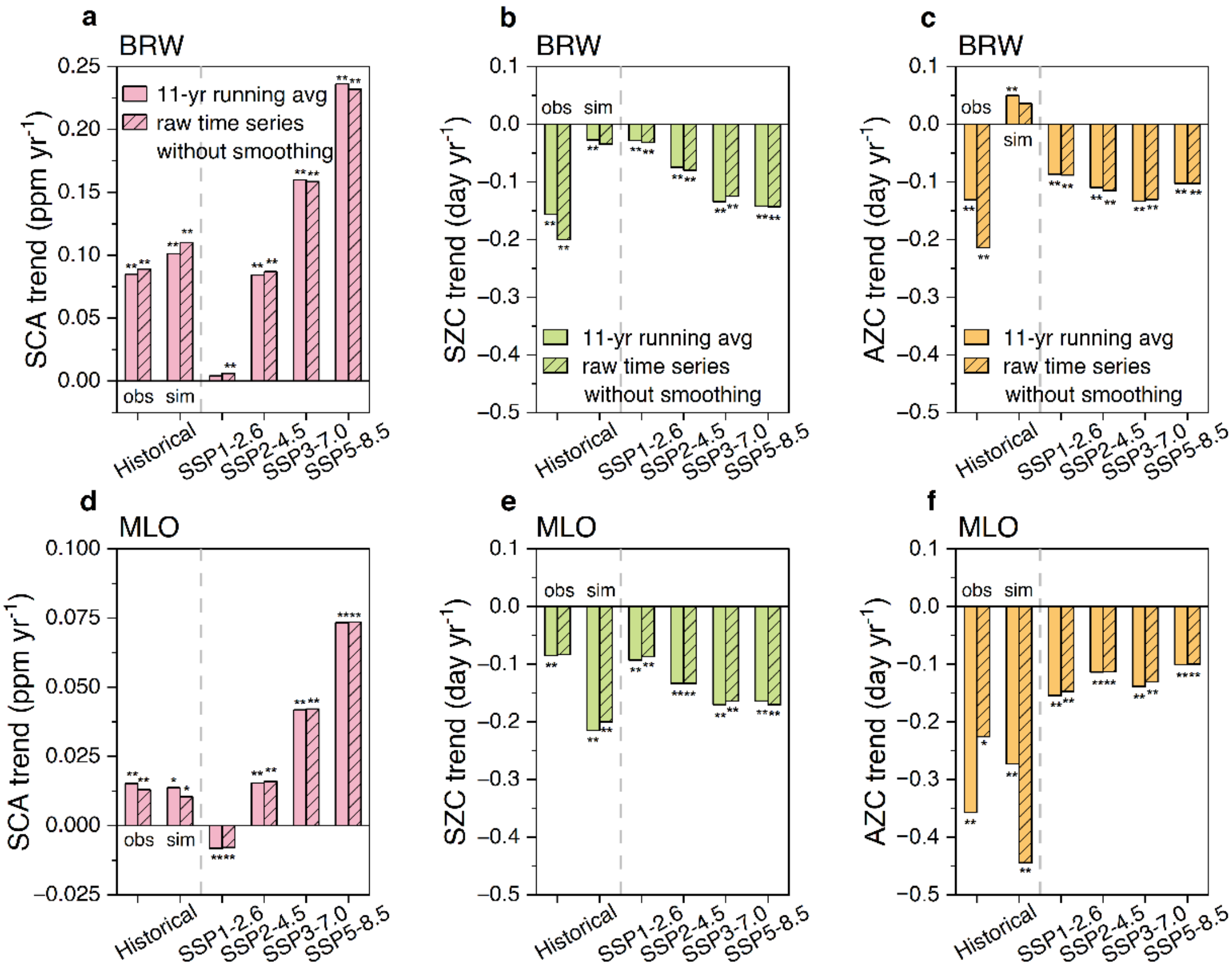

Supplementary Figure 36. Trends in the seasonality of atmospheric CO_2 under the historical period and four future scenarios at Barrow, Alaska (BRW; **a-c**), and Mauna Loa, Hawaii (MLO; **d-f**). “11-yr running avg” denotes trends calculated from 11-year running-mean time series of CO_2 seasonal parameters, whereas “raw time series without smoothing” denotes trends calculated from the original (unsmoothed) time series of the same parameters. The panels display the trends in SCA (left column), SZC (middle column), and AZC (right column). Statistically significant trends ($p < 0.05$) are marked by two dots above the bars, and marginally significant trends ($p < 0.1$) by one dot.

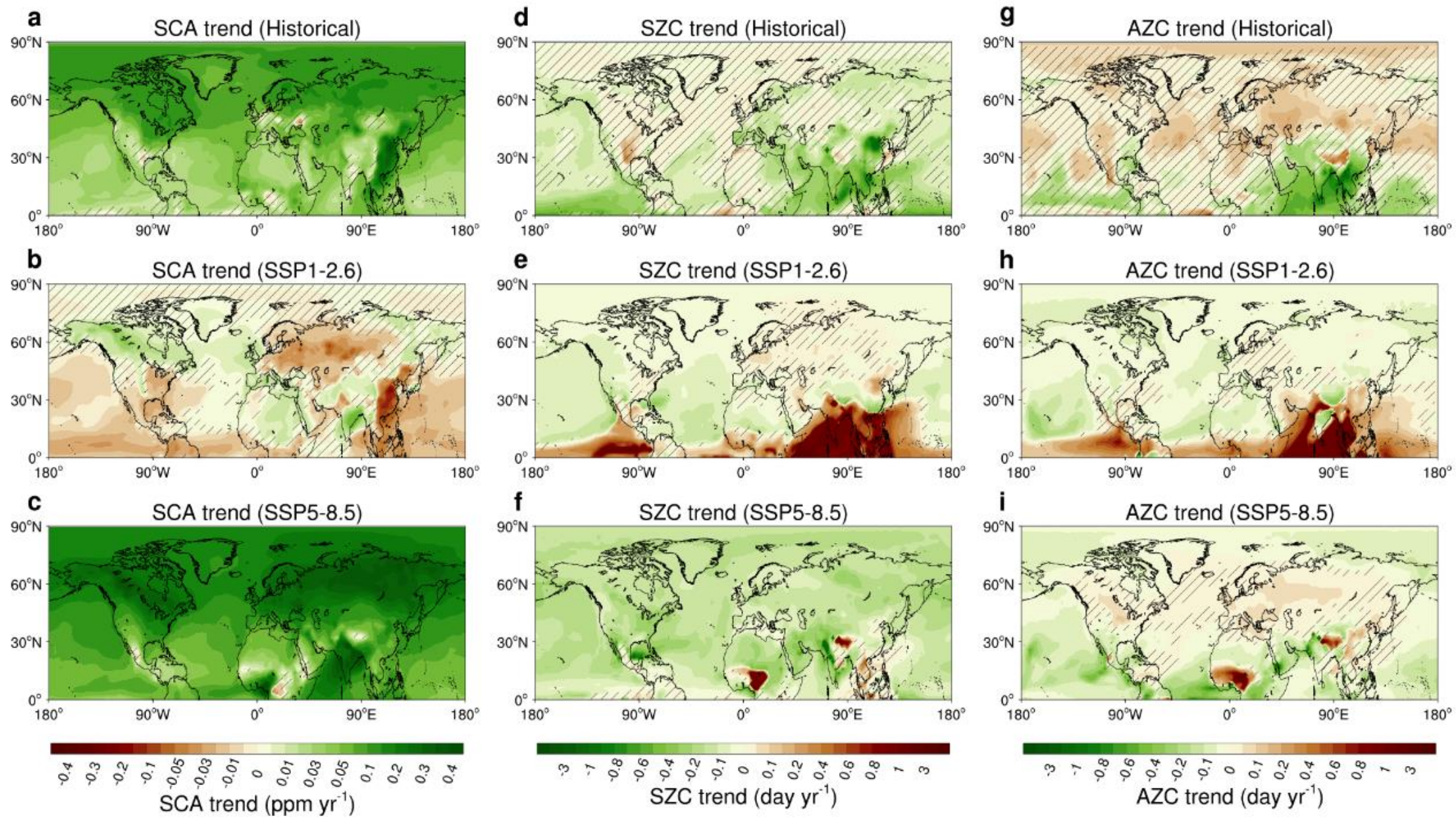

Supplementary Figure 37. Spatial patterns of simulated trends in atmospheric CO_2 seasonal parameters across the Northern Hemisphere under historical and future scenarios. Trends are calculated from the original time series of each seasonal parameter, rather than from 11-year running averages of the respective time series (Fig. 2). Simulated trends in seasonal cycle amplitude (SCA; **a-c**), spring zero-crossing date (SZC; **d-f**), and autumn zero-crossing date (AZC; **g-i**) for the historical period (top row), SSP1-2.6 scenario (second row), and SSP5-8.5 scenario (bottom row). Hatched areas denote regions where trends are not statistically significant ($p > 0.05$).

3. The manuscript argues that opposing regional trends under SSP1-2.6 “compromise” BRW’s representativeness for the northern high-latitude domain (p.6, lines 109–114). Although the argument itself makes sense, “compromise” implies a stronger quantitative basis. It would be beneficial either to provide quantitative support (e.g., temporal correlation between BRW seasonal amplitude and the broader boreal average historically versus SSP1-2.6), or alternatively, slightly adjust wording to indicate “reduces” or “weakens” representativeness, tied explicitly to the spatial pattern shown.

Response #3:

We thank the reviewer for this comment regarding the wording. We have replaced “compromise” with a more cautious formulation. The revised sentence now reads (lines 114-116):

"These contradictory regional effects explain the statistically non-significant trend in SCA at BRW station and imply a reduced representativeness of the station over high latitudes."

4. The sensitivity test applying recent MEIC seasonal distributions (2017–2021) to future CMIP6 emissions totals (p.20, lines 510–516; Results p.8, lines 185–188) is useful. Still, readers may interpret this as a realistic future scenario. Please briefly clarify in the Methods that this sensitivity test is intended primarily as an uncertainty bounding exercise rather than a realistic prediction of future fossil-fuel emission patterns. Also, mention explicitly that the MEIC data used here refers to the global gridded dataset, as many readers associate MEIC specifically with Chinese inventories (p.18, lines 473–478; p.20, lines 510–513).

Response #4:

We thank the reviewer for this valuable comment. We agree that the use of MEIC-derived seasonal distributions could be misinterpreted as representing a realistic future scenario, and we have clarified this point in the revised manuscript. In addition to the previously implemented approach, where CMIP6 total emissions are scaled using a fixed seasonal profile derived from MEIC, we further conducted a sensitivity experiment that applies sector-specific seasonal profiles from MEIC to the corresponding CMIP6 sectoral emissions (see Response #2 to Reviewer 2 for details). These experiments are designed to better capture sectoral differences in emission seasonality and to explore potential variations in the seasonal phase of total emissions.

To avoid misinterpretation, we have revised the Methods section to explicitly state that these constructed emission datasets are intended as uncertainty-bounding sensitivity experiments rather than realistic projections of future fossil fuel emission patterns (lines 521-536).

"To evaluate the sensitivity of our results to uncertainties in the seasonality of fossil fuel CO_2 emissions, we constructed three alternative emission datasets (Supplementary Fig. 19). In the first case, sector-specific monthly profiles averaged over 2017–2021 from the MEIC global inventory were applied to the CMIP6 annual sector-specific emissions at each grid cell. We further designed two idealized experiments to explicitly perturb the seasonal structure of emissions beyond historically observed patterns. In one experiment, emission seasonality

gradually transitions toward a uniform monthly distribution by 2100, thereby eliminating the seasonal cycle. In the other, emission seasonality is progressively reversed relative to the 2030 baseline, representing a pronounced phase shift. These three constructed future fossil fuel CO_2 emission datasets are intended primarily as an uncertainty-bounding analysis rather than as realistic projections of future emission patterns. The resulting emissions were used to drive transport simulations CMIP6_Base and CMIP6_FF under four future scenarios. The results confirm that the projected seasonal cycle metrics are robust to these differences in prescribed emission timing (Supplementary Figs. 20-22)."

We also clarify in the Methods section (lines 486-488) that the MEIC data used here represent a global dataset:

"The global fossil fuel CO_2 emissions from Multi-resolution Emission Inventory model for Climate and air pollution research (MEIC)[63] spanning the period between 1979 and 2021 were used in this study."

Minor editorial issues:

1. The description of future fossil-fuel emissions in CMIP6 scenarios appears twice (p.19, lines 498–501). Combine or delete one instance to remove redundancy.

Response #5:

We thank the reviewer for pointing out this issue. The first sentence has been removed (lines 512-514).

2. Correct "will closely tracks" to "will closely track" (p.9, lines 198–199).

Response #6:

The sentence has been revised to: "This result suggests that the SCA trend at MLO will closely track anthropogenic perturbations rather than terrestrial carbon dynamics under the SSP1-2.6 scenario" (lines 200-202).

3. Add "the" to "In boreal region," making it "In the boreal region," (p.8, line 163).

Response #7:

We have revised the sentence to read: "In the boreal region, the contribution of terrestrial ecosystems declines substantially, and fossil fuel CO_2 emissions become the dominant driver of the SCA trend, particularly in Eurasia" (lines 165-167).

Reviewer #3 (Remarks on code availability):

I did not attempt to run the code, as reproducing the full workflow likely requires substantial external datasets and computational resources beyond the scope of this review.

Response #8:

We thank the reviewer for this remark. The transport simulations were all based on GEOS-Chem model (version 12.9.3), which is publicly available at https://geoschem.github.io/. We also provide the code used to produce the main figures, including scripts for processing the original CO_2 concentration data to derive seasonal parameters and their trends at both site and grid scales, as well as code for quantifying the relative contributions of fossil fuel, terrestrial and oceanic fluxes to CO_2 seasonal trends.
